# Rescuing Alu: Recovery of *New* Inserts Shows LINE-1 Preserves Alu Activity through A-Tail Expansion

**DOI:** 10.1371/journal.pgen.1002842

**Published:** 2012-08-09

**Authors:** Bradley J. Wagstaff, Dale J. Hedges, Rebecca S. Derbes, Rebeca Campos Sanchez, Francesca Chiaromonte, Kateryna D. Makova, Astrid M. Roy-Engel

**Affiliations:** 1 Tulane Cancer Center, Department of Epidemiology, Tulane University, New Orleans, Louisiana, United States of America; 2 Hussman Institute for Human Genomics, Dr. John T. Macdonald Foundation Department of Human Genetics, Miller School of Medicine, University of Miami, Miami, Florida, United States of America; 3 Department of Biology, Center for Medical Genomics, Pennsylvania State University, University Park, Pennsylvania, United States of America; Fred Hutchinson Cancer Research Center, United States of America

## Abstract

Alu elements are trans-mobilized by the autonomous non-LTR retroelement, LINE-1 (L1). Alu-induced insertion mutagenesis contributes to about 0.1% human genetic disease and is responsible for the majority of the documented instances of human retroelement insertion-induced disease. Here we introduce a SINE recovery method that provides a complementary approach for comprehensive analysis of the impact and biological mechanisms of Alu retrotransposition. Using this approach, we recovered 226 *de novo* tagged Alu inserts in HeLa cells. Our analysis reveals that in human cells marked Alu inserts driven by either exogenously supplied full length L1 or ORF2 protein are indistinguishable. Four percent of *de novo* Alu inserts were associated with genomic deletions and rearrangements and lacked the hallmarks of retrotransposition. In contrast to L1 inserts, 5′ truncations of Alu inserts are rare, as most of the recovered inserts (96.5%) are full length. *De novo* Alus show a random pattern of insertion across chromosomes, but further characterization revealed an Alu insertion bias exists favoring insertion near other SINEs, highly conserved elements, with almost 60% landing within genes. *De novo* Alu inserts show no evidence of RNA editing. Priming for reverse transcription rarely occurred within the first 20 bp (most 5′) of the A-tail. The A-tails of recovered inserts show significant expansion, with many at least doubling in length. Sequence manipulation of the construct led to the demonstration that the A-tail expansion likely occurs during insertion due to slippage by the L1 ORF2 protein. We postulate that the A-tail expansion directly impacts Alu evolution by reintroducing new active source elements to counteract the natural loss of active Alus and minimizing Alu extinction.

## Author Summary

SINEs are mobile elements that are found ubiquitously throughout a large diversity of genomes from plants to mammals. The human SINE, Alu, is among the most successful mobile elements, with more than one million copies in the genome. Due to its high activity and ability to insert throughout the genome, Alu retrotransposition is responsible for the majority of diseases reported to be caused by mobile element activity. To further evaluate the genomic impact of SINEs, we recovered and characterized over 200 *de novo* Alu inserts under controlled conditions. Our data reinforce observations on the mutagenic potential of Alu, with newly retrotransposed Alu elements favoring insertion into genic and highly conserved elements. Alu-mediated deletions and rearrangements are infrequent and lack the typical hallmarks of TPRT retrotransposition, suggesting the use of an alternate method for resolving retrotransposition intermediates or an atypical insertion mechanism. Our data also provide novel insights into SINE retrotransposition biology. We found that slippage of L1 ORF2 protein during reverse transcription expands the A-tails of *de novo* insertions. We propose that the L1 ORF2 protein plays a major role in minimizing Alu extinction by reintroducing active Alu elements to counter the natural loss of Alu source elements.

## Introduction

Long INterspersed Element-1 (LINE-1 or L1) and the Short INterspersed Element (SINE) Alu are non-long-terminal-repeat (non-LTR) retroelements that are responsible for approximately one third of the human genome [1]. Due to their ability to randomly insert throughout the genome [2], both L1 and Alu are capable of disrupting critical genes and causing a large diversity of genetic diseases [3]–[6]. The creation of an engineered L1 assay system specifically designed to rescue *de novo* L1 inserts in a culture system demonstrated that L1 insertion contributes significantly to genetic instability through retrotransposition-mediated deletions and rearrangements [7]–[10]. This assay has the added advantage of providing a valuable tool for analyzing aspects of the L1 insertional mechanism under controlled experimental conditions [11]–[13]. Computational analyses further corroborated that both Alu and L1 insertions are associated with genomic loss, rearrangements and structural variation in humans [14]–[16].

Prior to our development of a similar assay system for SINES, there are very few published details of recovered *de novo* SINE insertions in culture. Two previous reports account for a total of 12 fully characterized *de novo* Alu insertion events in culture [17], [18]. One of these approaches utilized an untagged AluSx to transfect cells and the Alu inserts were then detected by “panhandle” PCR amplification using an anchor that is attached to the restriction digested cellular DNA. The researchers evaluated a total of 101 PCR products and found that seven were *bona fide* Alu insertion events [18]. The other five Alu insertion events were recovered using a tagged Alu and inverse PCR approach [17], [18]. An additional published report describes eight inserts from two tagged rodent SINEs [19]. Thus, only 20 *de novo* SINE inserts from cell culture have been characterized prior to the work reported here. Because these data arose from different approaches, using different SINEs, and different cell lines, generalizations from the data become difficult.

New high-throughput approaches have yielded large amounts of data on mobile element insertion, including somatic events observed in cancer samples [20] and brain [21]. However, these approaches are limited by short sequence reads, the inability to sequence through homopolymeric A-tails, and high difficulty of recovery and validation of “singleton” events (very rare events, *i.e.*, somatic insertions) due to the inability to refer back to a reference clone. Although *in silico* and high throughput sequencing analyses provide valuable insights into retroelement activity, a tissue culture assay system provides a controlled genetic environment during retrotransposition that confers the ability to distinguish between retrotransposition-mediated events and those that occur post-insertionally with the added advantage of being able to manipulate SINE sequences for experimental evaluation. Here, we present the adaptation and development of an engineered recovery-construct that allows for the rescue of inserted tagged SINE elements in a tissue culture assay system and provide detailed data from over 200 rescued *de novo* Alu inserts.

## Results

### Creation of the SINE rescue vector

**Figure 1:**
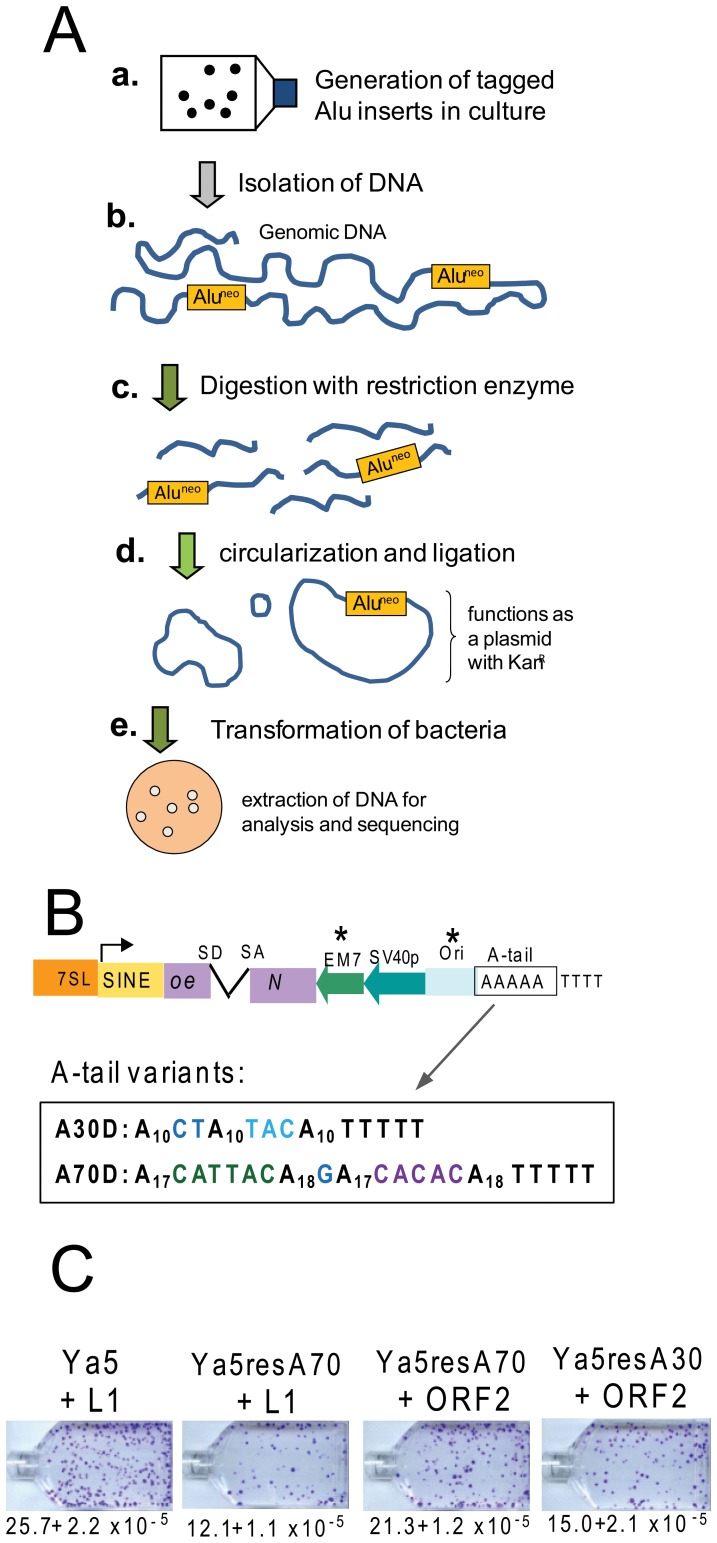
Experimental approach and retrotransposition efficiency of Alu recovery constructs. **A.** Experimental approach for the recovery of SINE inserts. **a.** Cells are transfected with the Alu rescue construct and grown under selection to obtain colonies containing the new Alu insertions. **b.** Genomic DNA is extracted from transfected cells that have undergone selection for SINE inserts (G418^R^ foci). **c.** DNA is digested using a restriction enzyme not present within the expected insert sequence of the tagged Alu (Alu^neo^). **d.** Ligation is performed to favor circularization of the digested DNA. The circularized genomic DNA that contains the tagged Alu insert with the origin of replication and neomycin cassette will function as a plasmid. **e.** DNA is transformed into an *E. coli* strain capable of supporting the replication of the circularized DNA; the neomycin cassette confers resistance to kanamycin. Plasmid DNA is extracted from individual bacterial colonies for analysis and sequencing. **B.** Schematic of the Alu rescue construct. The construct is a modification of an Alu Ya5 tagged with the reporter cassette designed to detect retrotransposition events. The neomycin resistance gene present in the opposite orientation relative to the Alu transcription is disrupted by an inverted intron (neo^TET^). SD and SA indicate the splice donor and splice acceptor sites. Only retrotransposition of the spliced transcript confers G418-resistance to eukaryotic cells, and kanamycin resistance to bacterial cells in the recovery step. “Ori” represents the origin of replication. The critical components introduced to the construct required for the rescue strategy are indicated by asterisks. The sequence composition of the A-tail for the two construct variants is shown. **C.** The Alu rescue constructs show lower retrotransposition efficiency than the parental construct. Retrotransposition efficiencies of the parental pBS-Ya5-*neo^TET^* (Ya5), pBS-Ya5rescue-A70D-SH (Ya5resA70) and pBS-Ya5rescue-A30D (Ya5resA30) driven by either an untagged L1 (pBS-L1PA1_CH_notag) or an ORF2p expression vector (pBudORF2_CH_) were determined in HeLa cells. The mean ± SEM observed neo^R^ colonies (retrotransposition) are indicated below the representative sample for each construct.

Because SINEs are transcribed by RNA polymerase III (pol III), several obstacles introduced by the RNA pol III transcriptional requirements must be overcome to develop experimental methods to investigate the mechanistic aspects of Alu retrotransposition. Due to these constraints, prior methods for the recovery of SINE inserts in culture have been mostly limited to inverse PCR [17], [18]. As an alternate approach, we have developed an Alu recovery system by redesigning the existing Alu-*neo* ^TET^ vector [17], following the strategy used to create the L1 recovery vector [7], [8]. The principle of the method is shown in Figure 1A. We performed extensive modifications and adaptations of the Alu construct [17] (Figure 1B). First, a bacterial promoter (EM7) was inserted upstream of the neo^TET^ cassette to obtain kanamycin resistance in bacterial cells. We then introduced a minimal γ origin of replication (305 bp) of plasmid R6K [22], [23], which was sequence modified to allow RNA polymerase III (pol III) transcription. The R6KγORI was selected due to its smaller size. Specific sections of the R6KγORI were changed by site directed mutagenesis to eliminate runs of four or more thymidine residues that could function as internal RNA pol III terminators (details in Materials and Methods). Finally, in order to analyze A-tail expansion, we substituted the original homopolymeric A-tail with a dA-rich sequence containing non-A disruptions (Figure 1B). As expected, the added sequence length (439 bp) and the variation in A-tail length and composition [24] reduced the retrotransposition efficiency of the Alu rescue construct to close to 50% when directly compared to the parental construct (Figure 1C). The retrotransposition efficiency of the Alu rescue construct increases when using a highly efficient driver vector expressing only L1 ORF2p (Figure 1C). However, the added length to the tagged Alu RNA did not appear to contribute to 5′ truncation of the Alu inserts, as fewer than five percent were truncated (see details below).

### Recovered tagged inserts exhibit hallmark signatures of retrotransposition insertion

**Figure 2:**
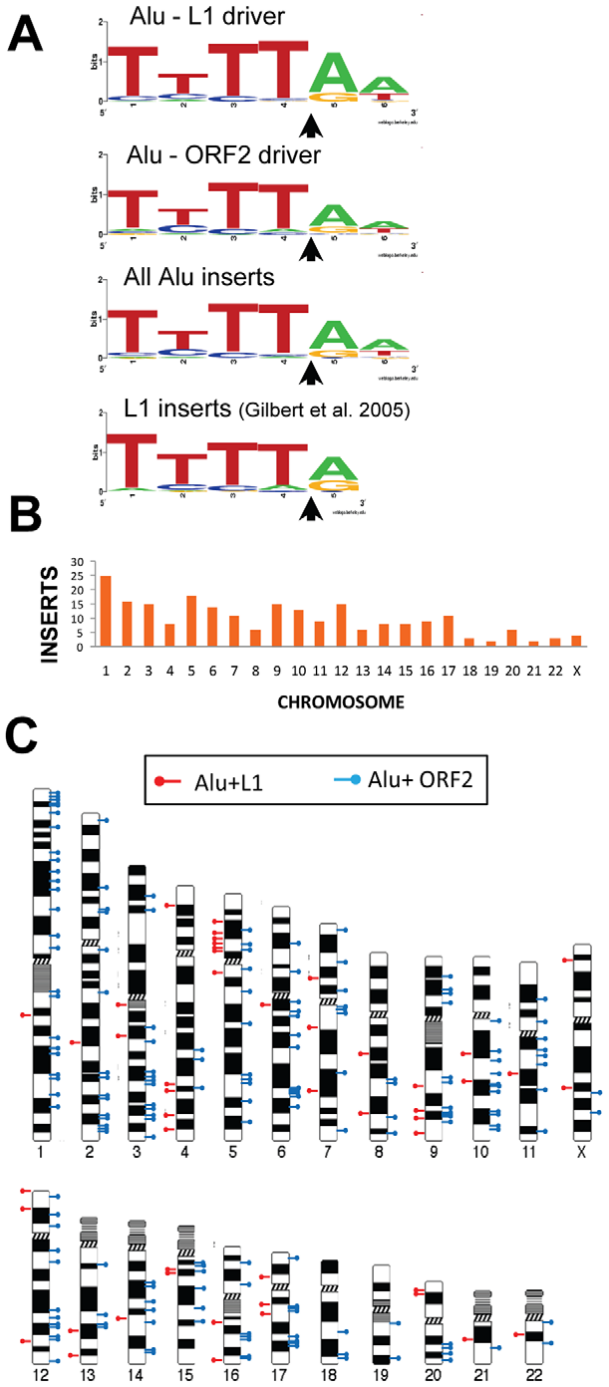
Endonuclease target site and chromosomal distribution of recovered Alu inserts. **A.** The rescued Alu insert target consensus site matches the known L1 endonuclease site. Sequence logos [83] representing genomic pre-integration site consensus sequences of the rescued Alu inserts driven by full-length L1, L1 ORF2 alone, and all Alu inserts combined are shown. Previously published data for *de novo* L1 inserts recovered from culture [9] is shown for comparison. The arrow indicates the endonuclease cleavage site. **B.** Chromosomal distribution of *de novo* Alu inserts. Histogram of the chromosomal distribution of recovered *de novo* Alu inserts is shown. Note that chromosome Y was not included as all data were generated using the female cell line HeLa. **C.** Ideogram shows chromosomal locations of Alu inserts driven by a full-length L1 (red pins) and by ORF2 alone (blue pins).

We recovered a total of 226 Alu inserts from transfected HeLa cells (complete sequence details of the insertions are available in Text S1 and Table S1a). Because transfection of the L1 ORF2 protein alone supports Alu retrotransposition in HeLa [17], [25], we wanted to determine if ORF2-driven Alu inserts differed from those driven by a full length L1. We analyzed *de novo* Alu inserts driven by ORF2 alone (N = 178) or driven by full-length L1 (N = 48) for comparison between the sets. Overall, we found no significant differences between Alu inserts driven by full-length L1 vs. ORF2 alone (Tables 1 and 2 and Figure 2). Therefore, we primarily report the combined observations of all Alu inserts.

**Table 1:** Direct repeat length of recovered tagged Alu inserts.

Length (bp)	Total	ORF2 driver	L1 driver
Mean± S.D.	14.0±3.0	14.0±2.5^a^	14.9±3.3^a^
Median	14	14	15
Range	5–27	6–21	5–27
Number of inserts (N)	192	145	47

a T-test (ORF2 vs. L1) p = 0.078.

**Table 2:** Percent G+C and repeat element content of Alu 20 kb pre-insertion loci.

	20 kb	% Repeat elements				
	% G+C	Alu	L1	L2	MIR	MALR
Alu+L1 driver	40.6	12.5	17.0	3.6	2.8	3.1
Alu+ORF2 driver	42.0	15.0	13.9	3.8	3.1	2.9
Alu−Total	41.7	14.4	14.5	3.8	3.0	2.9
L1^a^	41.0	13.0	13.3	3.7	3.2	3.7
Random (Simulated)	41.0	10.0	16.4	3.2	2.9	3.8
Genome	41.0	10.6	16.9	3.2	2.5	3.6

a data from [36].

We obtained sequences from both 5′ and 3′ genomic flanking sequence of the inserts (Text S1 and Table S1a). Of the fully characterized *de novo* Alu inserts, the vast majority (∼96%) exhibited the hallmark characteristics of retrotransposition: direct repeats flanking the insert, a 3′ oligo dA rich tail and a target site resembling the L1 endonuclease consensus sequence [7], [9], [26]–[28]. Atypical insertions (lacking the retrotransposition hallmarks) were associated with genomic deletions or rearrangements (details below). The observed target consensus site for the inserts (5′-TTTT/AA-3′) is identical to the known preferred L1 endonuclease cleavage site [8] (Figure 2A), suggesting that most Alu inserts in our culture system initiated by the conventional endonuclease-dependent target primed reverse transcription (TPRT) mechanism. The direct repeats ranged from 5–27 bp, with a 14.0±3.0 bp average (Table 1). Eight of the recovered events (3.5%) resulted in an Alu insert with a 5′ truncation. This is less than half of what is observed in the genome (∼10% of Alu elements are 5′ truncated) [1], [29].

As proof of the versatility of the method, we modified our construct to the study of other SINE elements. We recovered seven inserts from two rodent SINEs by substituting the BC1 or B2 sequences for the Alu sequence in the rescue vector [30]–. Sequence analysis revealed that the fully characterized *de novo* inserts (five B2 and one BC1) also contained the endonuclease target site and insertion characteristics of typical L1-mediated retrotransposition (Text S2 and Table S1c)

### Alu retrotransposition-mediated genomic rearrangements associate with atypical insertions

Our analyses of the recovered Alu inserts determined that less than four percent of the inserts (8 of 226; 3.5%) lack the typical characteristics of TPRT-mediated Alu insertions. Six of these insertions (2.7%) contain two features indicating that the insertion likely completed through recombination with an existing Alu present at the genomic site (Text S1 and Table S1a). First, the recovered sequences of these clones contain a chimeric sequence between the genomic and the tagged Alu. Secondly, they lack the characteristic flanking direct repeat. In several cases, the recombination caused a loss or a rearrangement of the genomic sequence (Figure S1). This type of retrotransposition mediated deletion has been previously described for L1 [7]–[9] and Alu [14], [34]. For one particular example, clone 57, the immediate 3′ and 5′ genomic sequences flanking the insert are 99 kb apart in the reference genome assembly. PCR analysis of the transfected and untransfected HeLa DNA confirmed that this genomic rearrangement was not pre-existing in the HeLa cell line, but instead is likely associated with the Alu insertion (Figure S2). Interestingly, clone 57 is the only insert in our data set with no identifiable A-tail. An additional two inserts of the fully characterized Alus (0.8%) also lacked the canonical endonuclease cleavage sites and direct repeats of TPRT insertion (clones 108 and 203), suggesting an endonuclease independent mechanism of insertion [11], [35]. These clones were also associated with potential genomic rearrangements (details in Text S1).

### Genomic distribution of recovered Alu inserts

We used the 5′ flanking genomic sequence from the 226 rescued inserts to determine their genomic location. Alu insertions were recovered from all chromosomes (Figure 2B). The distribution of Alu inserts across chromosomes appears largely random (Figure 2C), in agreement with previous reports of L1 insertions from tissue culture [9]. A previous study showed an enrichment of L1 inserts associated with the c-*myc* gene on chromosome 8 [36]. However, we did not observe Alu insertions associated with the c-*myc* gene.

We analyzed the G+C and repeat element sequence content of the pre-insertion loci in 20 kb intervals of all 226 Alu inserts (Table 2). Relative to the genomic average and modified HeLa karyotype, we find that the overall pattern of Alu pre-insertion sites is consistent with a previous analysis of *de novo* tagged L1 inserts [36]. Pre-insertion sites were Alu rich and L1 poor, although the tagged L1s inserted into comparatively more L1 poor regions (13.3% for L1 inserts from Gasior et al. 2006 compared to 15.5% for Alu inserts in the present study). Alu insertions that were driven by ORF2 alone landed in genomic regions that were more L1 poor than insertions driven by full-length L1 (13.9% compared to 17.0%). However, this observed difference is not statistically significant (two sample, two-tailed t-test, p = 0.172).

### Insertion bias of recovered Alu elements to genes and conserved elements

We next assessed the distribution of recovered inserts relative to annotated genes in the human reference genome. We find that 57.7% of all combined inserts landed in genic sequence compared to 42.3% that were intergenic (Table 3). As previously indicated, there is no significant difference between the genic/intergenic distribution of L1 and ORF2 driven Alu inserts (Pearson *X* ^2^ = 3.41; p = 0.065). Six of the Alu inserts landed within exons, but only two caused disruption of coding sequences, while the other four landed in the 5′ or 3′ untranslated regions (UTRs) of coding exons (Table 3). Just over a third (36.2%) of genic *de novo* Alus inserted in the sense strand, compared to (63.8%) on the opposite strand. This observation is slightly more skewed than the 55% antisense to 45% sense strand intronic distribution of the sequenced human genome [37], [38]. Overall, these data are consistent with an antisense bias (binomial probability, p = 0.002).

**Table 3:** Distribution of recovered tagged Alu inserts.

Location of *de novo* Alu inserts	Genic	Intergenic
*Total (%)*	130 (57.7%)	96 (42.3%)
*Intron (%)*	125 (96.2%)	-
*Exon-coding sequence (CDS) (%)*	2 (1.5%)	-
*Exon- untranslated region (UTR) (%)*	4 (3.0%)	-

Orientation relative to the gene^a^		
*Same (%)*	47 (36.2%)	-
*Opposite (%)*	83 (63.8%)	-

a same vs. opposite: binomial distribution test p = 0.002.

To further analyze Alu insertion preferences, we assessed the *de novo* Alu inserts relative to features that were found to associate with the genome-wide distribution of Alus in a previous evolutionary analysis [39]. In this approach, the 226 *de novo* Alu inserts observed here are localized within a system of 2765 non-overlapping human genome 1 Mb windows as employed in [39] and statistically evaluated for association with previously described genomic features (details in Materials and Methods). Nine genomic features were selected to evaluate genome landscape, recombination and natural selection (details in Table 4 and Table S2). For each feature, we contrasted 203 insert-containing windows and the 2562 insert-free windows, using the Mann-Whitney-Wilcoxon test (see Materials and Methods). After Bonferroni correction for multiple testing, our results (Table 4) indicate that the *de novo* Alus integrated in genomic regions that: (a) are rich in genes and highly conserved elements (suggesting function), (b) have high GC-content, (c) contain a 13-mer associated with recombination hotspots and genome instability (Myers et al. 2008) and (d) are enriched with SINEs, confirming that our observations of the 2-kb flanking regions (Table 2) may extend up to 1 Mb. We repeated the analysis using random subsets of the *de novo* inserts and the results remained consistent (data not shown).

**Table 4:** Genomic features with significant differences in their distributions between Alu insert-containing versus insert-free windows.

Genomic feature	Medians of the Integration windows	Medians of the Non-Integration windows	Absolute difference in medians	Z-value	p-values of the Mann-Whitney-Wilcoxon test
Gene content	0.3027	0.2164	0.0863	5.75	<0.0001^a^
SINE content	687.5	523	164.5	6.14	<0.0001^a^
GC content	0.4125	0.3984	0.0141	4.49	<0.0001^a^
Most conserved element density^b^	785.5	750	35.5	3.01	0.0013^a^
Genome instability 13-mer frequency^c^	55	42	13	5.34	<0.0001^a^

a : Testing the hypothesis that Alu insert-containing windows have a right-tailed distribution compared with insert-free windows.

b : Most conserved elements density as defined in [46].

c : The frequency of the CCNCCNTNNCCNC motif associated with crossover events at recombination hotspots. They cluster at non-allelic homologous recombination (NAHR) breakpoints and mitochondrial deletion hot spots [50].

### *De novo* Alu inserts show no evidence of RNA editing

Some transcripts containing Alu sequences have been reported to be subjected to RNA editing [40]–[43]. However, these examples refer to Alu sequences within RNA pol II generated transcripts. Thus, we evaluated our data for evidence of editing of RNA pol III transcripts. A total of 52,039 bp of *de novo* Alu inserts were analyzed, which excluded the middle A-rich region of the Alu sequence from the analysis. We only found six point mutations (∼0.01%), three clustering within a 20 bp sequence of a single Alu insert. None of the changes reflected the expected sequence changes due to RNA editing and may reflect errors introduced during reverse transcription by the L1 ORF2 or random mutations. Our observations are consistent with previously published data showing no evidence of editing by three APOBECs (3A, 3B or 3G) on the neomycin cassette sequence from inserts of a tagged Alu [44], [45].

### A-tail expansion

An intriguing observation associated with SINE insertion events is the reported increase in A-tail length of new inserts relative to their source element [17]–[19]. We used constructs with non-A disruptions within the A-tail to further investigate the underlying mechanisms of A-tail expansion in recovered *de novo* Alus. We used two constructs containing different A-tails (Figure 1B) to determine if differences in A-tail disruptions or length might differentially affect new insert A-tail sequence. The shorter A-tail construct (A30D) contains three polyA segments of 10 adenosines, separated by two different disruptions (CT and TAC, Figure 1B). The longer A-tail construct (A70D) is more than twice as long as the A30CT tail (82 bp compared to 35 bp) and contains four polyA segments of 17 or 18 adenosines separated by three different disruptions (CATTAC, G, and CACAC, Figure 1B).

**Figure 3:**
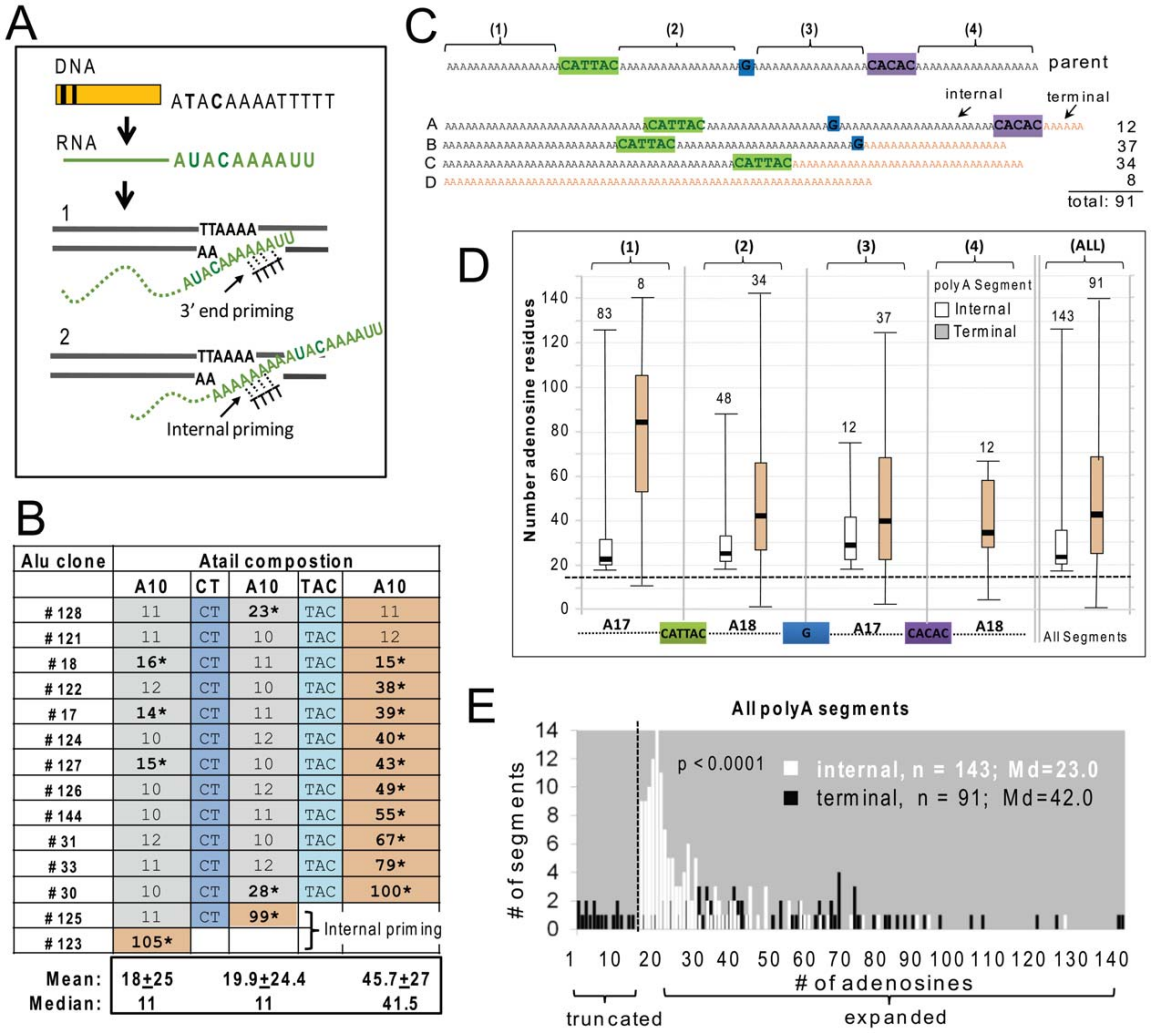
Adenosine expansion occurs throughout the A-tail sequence of Alu inserts with a bias toward the 3′terminal polyA segment. **A.** Schematic of the generation of a typical SINE transcript and details of the insertion step during the target primed reverse transcription (TPRT). SINE transcripts are normally not processed like mRNAs, therefore the RNA sequence (shown in green) directly reflects the parental DNA. The genomic DNA is cleaved at the endonuclease consensus site (5′-TTTT/AA-3′, bottom strand) by ORF2p, exposing thymidines to base pair with the A-tail. During TPRT, priming can occur near the 3′ end of the Alu RNA A-tail (1) or internally (2). The retrotransposed Alu RNA is expected to generate an insert that is either the same size or shorter than the parental A-tail, following end or internal priming, respectively. **B.** A-tail expansion of *de novo* Alu inserts derived from the A30D Alu construct (pBS-Ya5rescue-A30D). The A-tail sequence and composition is shown across the rows for all 14 inserts. The top row shows the sequence composition of the parental A-tail, with three sets of polyA segments containing 10 adenosines separated by two disruptions (CT and TAC, shown in shades of blue). For each of the 14 inserts, the length of the poly A segments and presence/absence of disruptions are shown. Recovered insert polyA segments that are at least 40% longer than the source Alu construct are indicated by asterisks. The polyA segments were classified as internal (gray) or terminal (yellow) relative to the 3′ end of the insert. **C.** Schematic of the construct and representatives of the types of A-tail sequences observed in the *de novo* Alu inserts. Top shows a schematic representation of the parental A-tail in the A70D construct (pBS-Ya5rescue-A70D) that contains four sets (1–4) of polyA segments separated by three disruptions: CATTAC (green), G (blue) and CACAC (purple). Below are four generic representations of the types of inserts observed: Type A contains all polyA segments and non-adenosine disruptions; Type B contains polyA segments 1, 2 and 3 and the first two disruptions; Type C contains polyA segments 1 and 2 and the first disruption; and Type D contains a homopolymeric A-tail. Terminal segments in the insert types are shown in orange. Depending on where priming occurs, polyA segments 1–3 can be internal or terminal (for example segment 2 is internal in a type A but terminal in a type C insert). PolyA segment 4 can only be terminal. Each of the polyA segments was analyzed separately for number of adenosines (panels D and E). However, internal and terminal segments were separated because the initial location of priming for TPRT (as shown in panel A) can uniquely affect the size of terminal segments via polyA shortening. **D.** Evaluation of number of adenosines for each of the four polyA segments (1–4), separated into internal (white) vs. terminal (orange) categories. The schematic of the A-tail of the pBS-Ya5rescue-A70D construct is shown on the bottom with the four polyA segments aligned below the internal vs. terminal distribution pairings for that particular segment. The box-whisker plot for *de novo* Alu insert polyA segment length shows that expansions are not limited to the polyA segment closest to the 3′ end, but occur throughout the A-tail of the Alu insert. The horizontal dashed line indicates the size of the parental segment (17 or 18 As), with polyA sizes above the line representing adenosine expansions and those below the dashed line representing contractions. Numbers above each box-whisker indicate the number of polyA segments recovered for that category. Therefore, the numbers above the terminal segments also represent the number of TPRT priming events for each of the indicated segments. **E.** Histogram distribution of the length of the internal (white) and terminal (black) polyA segments of Alu inserts recovered from HeLa transfected with the Alu construct pBS-Ya5rescue-A70D is shown. The x-axis shows the number of adenosine residues within a segment and the y-axis shows the number of segments. The medians (Md) are indicated. A total of 91 fully characterized Alu inserts were recovered with 91 terminal and 143 internal segments (note that an individual A-tail can have more than one internal segment). A vertical dashed line indicates the boundary separating the polyA segments to the left that are shorter than the parental polyA segments (17 or 18 adenosines) from the ones showing an expansion on the right. Contractions/truncations are only observed in terminal segments (black bars to the left of the dashed line). Statistical difference between internal vs. terminal length distributions is indicated (p<0.0001, Mann-Whitney U test).

We fully analyzed A-tail sequence data from 14 Alu inserts generated from the construct with the short A30D A-tail and 91 inserts from the longer A70D construct (Figure 3). Overall, the *de novo* Alu inserts showed extensive A-tail expansion relative to the parental Alu. A-tail and insert characteristics for the individual inserts are detailed in Table S1b. Because the length of the A-tail at the 3′ end of the recovered inserts can vary depending on where priming occurs within the RNA molecule during TPRT (see Figure 3A), we grouped inserts based on the priming location. Internal priming has previously been observed for L1 inserts [2]. Priming location was inferred by the absence/presence of the non-adenosine disruptions. We define polyA segments of new inserts as “terminal” when the segment is used as the priming location for TPRT. Note that the “terminal” polyA segment of a new insert can be any one of the polyA segments from the reference parental element (shaded orange in Figure 3C) and that internal priming events can generate inserts with shorter individual polyA segments as well as shorter A-tails in general. Figure 3C shows examples of the four types of A-tails generated by construct A70D.

Although the A30D data set is much smaller, many of the observed characteristics were shared between both data sets. Figure 3B shows the A-tail length results for the A30D data set (data for the larger A70D set is provided in the Table S1b). Surprisingly, when the construct with this shorter A-tail was used, all but two Alu inserts (#123 and #125) primed at the most 3′ end polyA segment (Figure 3B). These two Alu30D inserts with A-tails lacking one or both of the non-adenosine nucleotide disruptions were likely the result of internal priming during TPRT (as illustrated in Figure 3A). In contrast, the majority of the priming occurred internally in the A70D dataset, but very few primed in the first or most “internal” polyA or segment #1 (8 out of 91 inserts, Figure 3D). The A30D and A70D data sets are significantly different with respect to having “complete” A-tails (all disruptions and polyA segments present) (Pearson *X* ^2^, p<0.001). It is possible that the added length of the A70D A-tail led to an increased frequency of internal priming by expanding the available area for priming to occur. In both sets, priming seldom occurred at a distance of less than 20–25 bp downstream from where the polyA segment initiates. The A70D data set also has significantly fewer than expected priming events within the most 3′ polyA segment, under the null hypothesis that priming locations are randomly distributed across the A-tail (Chi-square goodness-of-fit, p<0.0001).

We observed significant extension of the polyA segment length in both data sets. Closer inspection of the individual segment sizes revealed that the terminal segments are considerably longer than internal segments. The median terminal segment length (41.5 bp) for the A30D set is about 4 times longer than the median for internal segments (11 bp) (Mann-Whitney U test, p<0.0001). Similar to the A30D data, the 91 A-tails from the A70D data set showed a bias to 3′ end elongation of the inserts when the length of the internal polyA segments is compared to terminal segments (Figures 3D and E). The histogram (Figure 3E) shows the overall size distribution of all four polyA segments, separated into internal (white bars) vs. terminal (black bars) segments. Although both internal and terminal polyA segments increased in length, terminal segments are significantly longer (medians of 23.0 and 42.0, respectively; Mann-Whitney U test, p<0.0001). Almost all of the A70D terminal polyA segments (95.9%) show expansion of four adenosines or more, while just over half of the internal segments exceed this level of expansion (55.6%). Although there is a bias toward larger expansions occurring at terminal segments (gray bars, Figure 3D, Table S1b), all of the internal polyA sections showed at least a minor increase in length relative to parental segments (indicated by the dashed horizontal line, Figure 3D) with medians of 22 or more adenosines per polyA segment. In contrast, shortening only occurs in terminal segments, as we observed 17 inserts with shorter terminal polyA segments than the parental construct (Figure 3E, black bars left of the vertical dashed line and Table S1b). This suggests that the shorter terminal A-stretches may be a result of internal priming within the terminal polyA segments during the initial step of reverse transcription by ORF2p (Figure 3A).

### Expansion of polyA segments is not observed at the RNA level and is not an artifact of the cloning process

To determine if the observed A-tail expansion may have occurred at the RNA level, we generated cDNA clones using 3′ RACE (RT-PCR) from isolated RNA of transiently transfected cells using either construct specific primers or a generic anchored oligo-polydT primer (details in Materials and Methods). Sequence analysis of these clones clearly showed that the insert A-tail elongation could not be explained by RNA transcript variation. We observed only slight transcript sequence differences of 1–3 adenosine losses or gains per polyA segment (Figure S3). Moreover, we observed more than twice as many adenosine losses than gains in the cloned cDNA sequence derived from the transcripts, suggesting that the A-tail variation introduced by transcription or by our recovery and cloning methodology is more likely to lead to shorter A-tails. Analysis of clones recovered from PCR amplification of a DNA template also revealed a similar change in adenosine numbers (Figure S4), possibly indicating that these sequence differences in the cDNA are introduced during the bacterial growth or amplification steps during the RT-PCR steps of the 3′ RACE and are not reflective of the actual RNA sequence.

It is noteworthy that we did not observe the large adenosine amplifications in our analysis of RNA transcripts, making it unlikely that changes in the Alu RNA template are a significant mechanism for the A-tail expansion observed in our recovered clones. During the Alu rescue process, many of the loci containing the Alu inserts were independently recovered multiple times. If expansion of polyA segments is an artifact of the cloning process, we would expect to see segment length variation between independently recovered samples. Instead, we observed minimal sequence variation between the recovered samples derived from the same Alu insert. In eight randomly chosen A-tail examples with a combined 2444 bp, only one sample with a single adenosine insertion was observed (Figure S5). This observation is in stark contrast to the consistent and large A-tail length expansion of the Alu inserts. Our data strongly indicate that the recovery assay is unlikely to contribute to the large A-tail expansions observed.

## Discussion

Our SINE recovery method provides a complementary approach for comprehensive analysis of the impact of Alu on the human genome that can give novel insights into the biological mechanisms governing SINE amplification. In summary, the recovery of *de novo* tagged Alu inserts in HeLa cells revealed that (1) L1 and ORF2 driven Alu inserts are indistinguishable in human cells; (2) Alu insertion mediated deletions and rearrangements lack the hallmarks of retrotransposition, likely due to an alternate mechanism to resolve insertion intermediates; (3) inserts show an apparently random distribution across chromosomes, although a bias exists favoring insertion near other SINEs, highly conserved elements and genes; (4) *de novo* Alu inserts show no evidence of RNA editing; (5) TPRT priming was not observed within the first 20 bp (most 5′) of the A-tail, suggesting the possible interference of bound proteins to the transcript or an unknown spacing requirement needed to engage the RT, RNA and priming sequence; (6) L1 ORF2 protein may show slippage during reverse transcription, leading to the expansion of *de novo* Alu element A-tails; and (7) expansion occurs across the entire length of the A-tail, but with a bias toward the 3′ end.

A major advantage of our approach is the ability to study inserts that have experienced little or no selection and the ability to compare *de novo* inserts to the known reference source element. By comparing inserts from our tissue culture system to genomic Alu inserts, we can better understand how selection has shaped the current distribution of human Alu sequences. Our results indicate that different genomic features might be important for initial Alu integration, as studied here, vs. long-term evolutionary survival of Alu insertions in the genome [39]. In particular, here we show that Alus integrate in genomic regions rich in genes and in sequences categorized as “most conserved” [46], suggesting an integration preference into or near functional elements. The association of Alu integrations with gene-dense regions is intriguing and is consistent with the previously reported enrichment of Alus near housekeeping genes [47], [48]. Although speculative, this integration preference suggests Alu is a highly efficient mutagen of human genes. In addition, targeting gene rich regions may provide fertile ground for added damage due to genomic rearrangements generated during insertion [49]. Interestingly, among these significant features, only enrichment of the genome instability 13-mer [50] was also a significant positive predictor of the distribution of human-specific AluY elements, as identified in an evolutionary analysis [39]. This suggests that, except for this one common predictive feature, there are substantial differences between Alu integration and fixation preferences; while the present analysis largely captures integration, the published Alu distribution properties [39] reflect both integration and fixation. Increased Alu insertion near other SINEs provides a mechanism explaining the clustering of Alus in the human genome [51] and the common occurrence of tandem Alu inserts [52]. Having a higher density of Alu elements may facilitate non-allelic homologous recombination (NAHR), leading to the uneven genetic exchange between alleles that cause both deletions and duplications [49]. The importance of the genome instability 13-mer motif correlating with both Alu distribution and integration is that it highlights a convergence of recombination hotspots and high Alu density regions potentially contributing to Alu-mediated NAHR [49], [53]–[55].

Consistent with the observations obtained from genomic data mining [56], we have found that Alu retrotransposition is associated with genomic deletions and rearrangements. However, the lack of the structural retrotransposition hallmarks suggests that alternate means of resolving retrotransposition intermediates, such as recombination [8], [9], [14], [57] or non-homologous end joining [7], [8], [58] is likely contributing to the Alu-mediated genomic rearrangements/deletions. Overall, our findings validate the tissue culture system as a robust method to study SINE biology.

An important feature of our Alu rescue system is that we were able to directly compare *de novo* Alu insert A-tails to the parental source A-tails with engineered disruptions. This approach allowed us to determine that A-tail elongation occurs during reverse transcription by ORF2p, leading to expansion across the length of the A-tail, but with disproportionate expansion closer to the 3′ end. The portion of the A-tail used for base pairing during TPRT priming was likewise not random, with the majority of priming locations at least 25 or more bases away from the 5′ end of the A-tail. This priming location preference may reflect a physical constraint such as bound proteins that limit where annealing for reverse transcription can occur. Although speculative, a potential protein candidate could be polyA binding protein (PABP), which is known to associate with SINE RNPs [59], [60].

**Figure 4:**
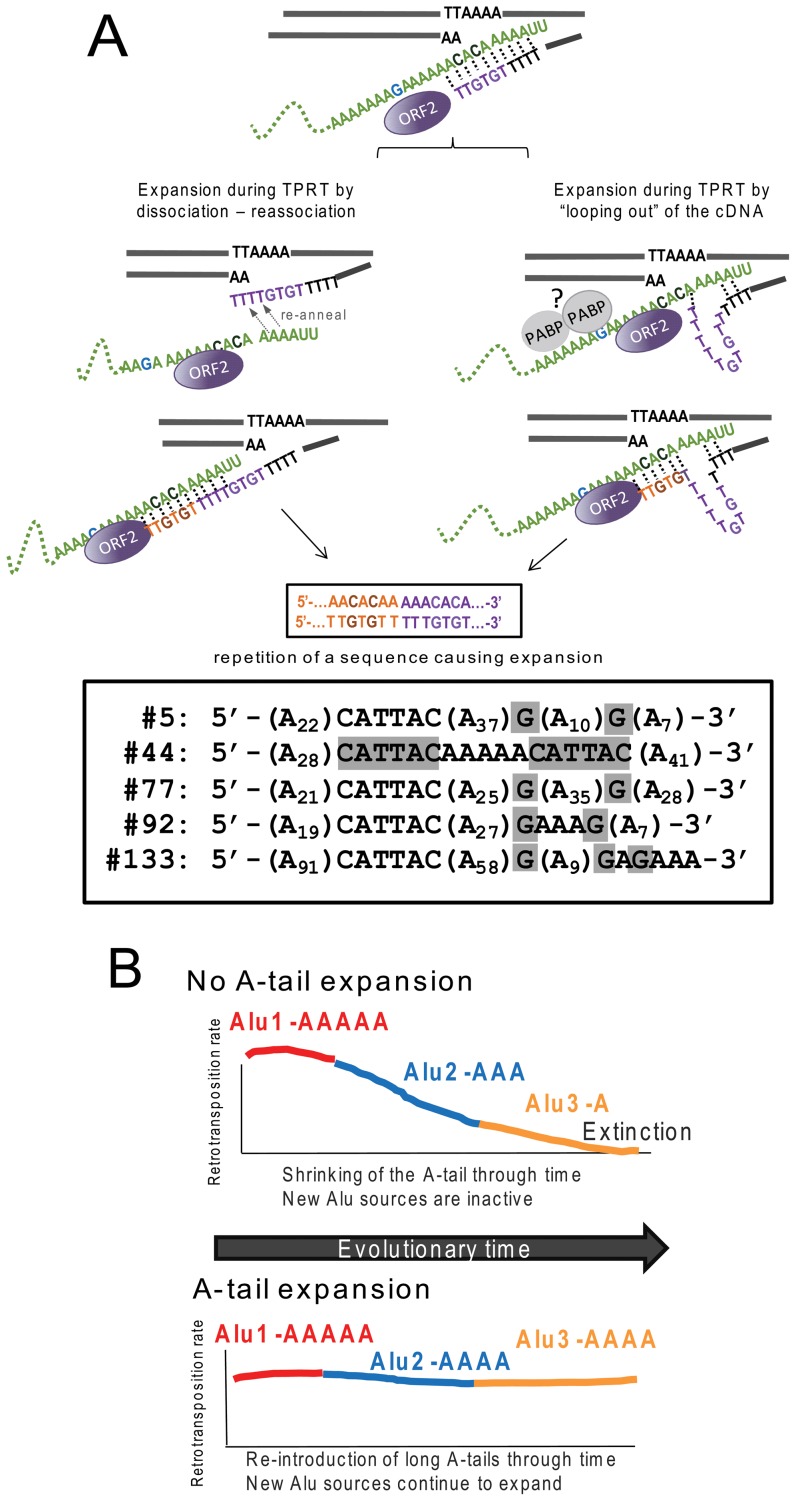
Reverse transcription by L1 ORF2p increases A-tail length of new Alu inserts and helps maintain viable Alu source elements over evolutionary time. **A.** A-tail expansion by the L1 ORF2p endonuclease. We propose a model where expansion of the A-tail occurs early during reverse transcription by the L1 ORF2p due to an unstable interaction between the Alu RNA (green) and the cDNA. A-tail expansion may occur through either “looping out” of the cDNA or rounds of dissociations and re-annealing between the two molecules causing priming and reverse transcription to reinitiate, leading to an increase in size. Note that the “looping out” of the RNA would cause a contraction of the A-tail instead of a lengthening. Although speculative, we propose that the RNA folding may be constricted due to interaction with proteins such as polyA binding protein (PABP, shown as gray circles). The interaction with the potentially bound proteins may also prevent priming from occurring at the most internal adenosines of the A-tail. The nascent cDNA strand (purple) initially only provides a weak interaction allowing for slippage or dissociation to occur. As the cDNA lengthens (orange), the additional hydrogen bonding between molecules eventually stabilizes the process. Depending on where the re-initiation of reverse transcription occurs during slippage, the non-adenosine nucleotides can be duplicated in the cDNA sequence as shown in our model. The bottom panel shows sequences of the A-tail of five recovered Alu clones with duplications of the non-A disruptions (highlighted in gray) that support the model. **B.** L1 ORF2p maintains active source Alu elements across evolutionary time. We present a model where A-tail expansion of new Alu elements plays an important role in replenishing Alu source genes through time. The two panels depict scenarios with (top) and without (bottom) A-tail expansion. Both scenarios begin with an early active source element (Alu1 activity in red). Through time Alu1 (red) gives rise to a new source element Alu2 (blue), which in turn gives rise to another source element Alu3 (yellow) with differing retrotransposition efficiency. Over time, Alu source elements accumulate inactivating mutations; thus the proliferation of Alu in a given population depends on the generation of new source elements. In the scenario with no A-tail expansion (top panel), new Alu source elements will have shorter A-tails (Alu3-A) and lose the ability to support retrotransposition. Without the possibility of expanding A-tails, extinction may occur. In the alternate scenario (bottom panel), new inserts are introduced with an expanded A-tail. The new Alu will become a source element with an expanded A-tail and generate the next subfamily of Alu inserts. The expansion of the Alu A-tail by the L1 ORF2 plays an important role in the continued genesis of new active source Alu elements within a population.

We present a model of slippage by ORF2p during TPRT (Figure 4A) favoring A-tail expansion. We propose that the beginning of TPRT only provides a weak interaction between the Alu transcript and the cleaved DNA strand through limited hydrogen bonding between base pairs. At this early stage, the complex may become dissociated, pausing reverse transcription until the interaction is re-established in a manner somewhat reminiscent of the reiterative synthesis of telomerase during reverse transcription. This is similar to the model proposed for the *I* factor, a non-LTR element in *Drosophila* [61]. In addition, telomerase slippage has been reported in *Saccharomyces* [62]–[64], *T. thermophila* [65] and *Candida albicans* [66]. Previous *in vitro* data also highlighted similarities between the L1 protein and telomerase by demonstrating that L1 ORF2 can initiate reverse transcription on oligonucleotide adapters simulating telomere ends [67]. Our model depicts two non-mutually exclusive mechanisms by which slippage can occur: either (1) complete dissociation occurs followed by re-annealing, or (2) partial dissociation occurs, causing the cDNA strand to “loop out” before base pairing can once again secure the complex. Interestingly, previous observations on the reverse transcription activity of the *Bombyx mori* R2 element demonstrate the incorporation of additional nucleotides that appear to arise from multiple rounds of the reverse transcriptase engaging the 3′ end of full length RNA templates [68]. However, potentially untemplated residues can be incorporated depending on the length and composition of the extreme 3′ end of the RNA. As cDNA length increases, the additional hydrogen bonding between the molecules stabilizes the process and reduces or eliminates slippage. This increased stability with cDNA extension provides a simple explanation for our observation of preferential 3′ A-tail expansion, as the probability of dissociation and expansion diminishes as the nascent cDNA strand grows in length.

In order for our model to favor A-tail expansion over shortening, re-annealing and/or “looping out” must preferentially occur as depicted in Figure 4A to duplicate A-tail nucleotides rather than delete them. Specifically, re-annealing of the cDNA strand must be further 3′ on the Alu RNA strand, with the cDNA strand looping out. We propose that the presence of proteins bound to the Alu RNA could affect re-annealing dynamics. For example, a potential candidate is poly(A) binding protein (PABP, as shown in Figure 4A), which may play an important role in favoring A-tail sequence duplication over deletion, serving as a physical barrier that promotes 3′ re-annealing and/or prevents the Alu RNA from looping out. Because our construct contains non-adenosine residues, sequence duplications can be easily identified. We recovered five Alu inserts with duplicated non-A disruptions in the A-tail sequence (Figure 4A). Duplication of 3′ sequences was previously observed for a recovered L1 sequence [9], indicating that this type of event also occurs during L1 insertion.

Several data support our proposed model. First, no expansion of the polyA segments is observed at the RNA level. Second, A-tail expansion occurs across all polyA segments. These observations are not consistent with RNA polyadenylation or template switching, as these processes would lead exclusively to expansion of the terminal polyA segment. Finally, duplications of the non-A disruptions are a strong indicator of slippage. Although polyadenylation of Alu transcripts and template switching may occur, our data indicate that these types of events are not the main processes contributing to the A-tail expansion of *de novo* Alu inserts in this assay system.

In contrast to L1, A-tail expansion of new Alu inserts has a significant biological impact on the perpetuation of active Alu elements in the human genome (Figure 4B). Although there are over one million Alu elements in the genome, the vast majority are inactive and unable to generate new copies. Several factors, including intrinsic nucleotide composition and adjacent genomic sequences, determine Alu retrotransposition capability [24], [69]. One such requirement for efficient Alu retrotransposition is the presence of an A-tail [70]. Because RNA polymerase III transcribed Alu RNA does not undergo enzymatic polyadenylation like mRNAs, Alu depends on the 3′ encoded polyA sequence to generate A-tail containing Alu transcripts. Previous work has shown that A-tails of individual Alu elements mutate rapidly leading to smaller and more heterogeneous tails [71], [72] and limiting retrotransposition capability [24]. As time progresses, the A-tails of active Alu source elements shrink and degrade, decreasing their ability to support retrotransposition. Therefore, without the reintroduction of new Alu copies with expanded A-tail sequence to counteract the rapid evolutionary loss of homogeneity and length, active Alu copies would be lost, leading to the eventual extinction of Alu. There are precedents for SINE extinction such as in the sigmodontine rodents, where SINE extinction may have preceded LINE extinction [73]. The acquisition of a longer A-tail by new inserts serves an important function in maintaining Alu activity through time and preventing the extinction of Alu or other A-tail dependent SINEs. Additionally, A-tail expansion can explain the appearance of “stealth-driver” Alu elements that have contributed to Alu expansion [74]. Thus, the L1 ORF2 protein is not only essential for Alu retrotransposition, but also plays a critical role in Alu perpetuation by expanding the A-tail of new inserts.

## Materials and Methods

### Plasmids

pBS-L1PA1_CH_notag- contains the fully codon optimized L1_RP_ driven by the CMV promoter and flanked at the 3′end SV40 polyadenylation signal in pBluescript [75].

pBud-ORF2_CH_- contains the fully codon optimized ORF2 from pBS-L1PA1_CH_notag, cloned into the expression vector pBudCE4.1 (Invitrogen), under control of the CMV promoter.

pBS-Ya5rescue-A70Du is derived from *p*AluYa5-*neo* ^TET^ [76] by substituting the 3′ region with a commercially synthesized sequence (Blue Heron biotechnology Inc.) schematic of the plasmid is shown in Figure 1B. The changes to Alu-*neo* ^TET^, include the introduction of a bacterial promoter (EM7, 134 bp) upstream of the *neo* ^TET^ cassette to obtain kanamycin resistance in bacterial cells and the introduction of our modified version of the minimal γ origin of replication (ORI) of plasmid R6K [22], [23]. We selected the R6KγORI for two reasons: first, its small size (305 bp) helps minimize transcript length, and second, it has the fewest poly-T runs of all ORIs evaluated. Two sections of the R6KγORI were changed by site directed mutagenesis to eliminate RNA pol III terminators. The 3′end contains a non-homogeneous 80A-tail, the BC1 unique (“u”) region and a pol III terminator (Figure 2C).

pBS-Ya5rescue-A70D, -the BC1 unique region of the pBS-Ya5rescue was removed by PCR but still contains the A-tail with the three disruptions.

pBS-Ya5rescue-A70D-SH, -the Shine-Dalgarno sequence was modified to remove AT richness that could function as a RNA polymerase III terminator [77] from pBS-Ya5rescue-A70D.

pBS-Ya5rescue-A30D- the A-tail of pBS-Ya5rescue was replaced by 30 adenine run with two disruptions, (details in Figure 2C).

pBS-B2rescue-A70D the 7SL-Alu sequence of pBS-Ya5rescue-AT was replaced by the 7SL-B2 sequence of pB2-*neo* ^TET^ [76], [78].

pCEP-Ya5rescue-AT,-the complete tagged Alu rescue sequence was introduced into the *Sal*I sites of the pCEP4 (InVitrogen) that removes the multicloning site with its promoter and polyadenylation signal, using a PCR approach to add compatible *Sal*I overhangs to the amplicon.

Plasmids were purified by alkaline lysis and twice purified by cesium chloride buoyant density centrifugation. DNA quality was also evaluated by the visual assessment of ethidium bromide stained agarose gel electrophoresed aliquots.

### Site directed mutagenesis

Site directed mutagenesis of the R6Kγori in the pR6Kan plasmid (Epicentre Biotechnologies) was performed using the commercially available Stratagene kit following the manufacturer's recommended protocol. Changes were introduced sequentially using the following primers in independent reactions: 1^st^ site: 5′-AGTTGCTGATTTATATTAAT**A**TTATTGTTCAAACATGAGA-3′ and 2^nd^ site: 5′- AAGCCTTATATATTCTT**V**TT**V**TTCTTATAAAACTTAAAACC-3′ (See Figure 2B). The final sequence of the construct resulted in the first V = G and the second V = C. The nucleotides targeted for mutagenesis are underlined. These primers were specifically designed to eliminate any four contiguous thymidines that may function as RNA polymerase terminators. Individual clones were grown and sequenced to confirm the introduction of the desired nucleotide changes. Because R6Kγori is the only origin of replication of the plasmid used in the mutagenesis, only functional mutations yield bacterial colonies, eliminating the need to verify functionality of our mutated sequences.

### Retrotransposition assay

The basic transient Alu retrotransposition assay was performed as previously described with some minor modifications [17]. HeLa cells (ATCC CCL2) were seeded in T-75 flasks at a density of 1×10^6^ cells/flask. Transient transfections were performed the next day using the Lipofectamine and Plus reagent (InVitrogen) following the manufacturer's protocol using 10 µg of the Alu rescue vector plus 2 µg ORF2 expressing vector or 2 µg of the untagged L1 vector. Following the removal of transfection cocktail, the cells were grown for 24 hr before adding the media containing 400 µg/ml G418 (Fisher Scientific) for selection. To determine evaluate retrotransposition, colonies were stained after 14 days of growth in selection media. To recover Alu inserts, the G418 resistant cells were grown under selection for 14–26 days to produce enough replicated cells for DNA isolation and the Alu insert recovery procedure. Fully confluent flasks of expanded G418 resistant cells were trypsinized and centrifuged in a new tube to be used for DNA extraction.

### Alu insert recovery

DNA extraction was performed using the DNA-Easy kit (Qiagen) following the manufacturer's recommended instructions. We used a modification of a previously described protocol [13]. Briefly, 200 µg of extracted DNA was digested for at least 5 hours at 37°C with 200 U of *Hin*dIII, *Eco*RI, *Spe*I, *BsrG*I, *Nhe*I or *Nde*I followed by heat inactivation of the enzyme by incubating at 65°C for 20 minutes. The digested DNA was diluted to a final volume of 1000 µl containing 1X T4 DNA ligase buffer and 1200 U T4 DNA ligase and incubated overnight at 16°C. After ligation, the sample was concentrated using a Microcon YM-50 filter (Amicon), washed twice with 500 µl distilled water and concentrated to a final volume of approximately 20 µl. The sample was incubated with 50 µl of electrocompetent *E. coli pir*-116 [*F^−^ mcrA Δ(mrr-hsdRMS-mcrBC) φ80dlacZΔM15 ΔlacX74 recA1 endA1 araD139 Δ(ara, leu)7697 galU galK λ- rpsL (Str^R^) nupG pir-116(DHFR)*] TransforMax™ EC110D™ (Epicentre Biotechnologies) in a 0.4 cm cuvette (BioRad) and pulsed using a MicroPulser power source (BioRad) at the manufacturer's preset conditions for bacteria and plated on LB plates containing 50 µg/ml kanamycin. Plasmid DNA was obtained from individual bacterial colonies using the Wizard Plus SV miniprep purification system (Promega). Inserts were initially analyzed by restriction site mapping. Samples were sent for sequencing to either the Translational Genomics Research Institute (TGen), Arizona or to Elim Biopharmaceuticals, Inc, Hayward, California. Lasergene 8, Seqman software was utilized for sequence analysis.

### Analysis of Alu inserts

Genomic location and details are provided in Text S1 and Table S1. The genomic position of each rescued Alu insertion was determined by BLAT (http://genome.ucsc.edu) search using the human genome reference (GRCh37hg19). After manual verification of each insertion position, 20 kb flanking regions (10 kb 5′ and 3′ of the insertion point) were extracted via custom PERL scripts for calculation of GC content and RepeatMasker (V.3.2.8) analysis. The relative abundance of Alu, L1, L2, MIR, and malR elements was tracked for each recovered insertion.

To examine how the genomic regions of the recovered inserts compared to that of Alu elements of various age classes, [1000/100] randomly selected Alu elements from AluJo, AluSx, AluSp, AluYa5, AluYb8, and AluYb9 subfamilies were analyzed in the same fashion as described above. Simulation of random insertion of L1 sequences into a genome possessing a HeLa karyotype was conducted using custom Perl scripting. For the purpose of the simulation, the sequenced nucleotides of the human genome (version hg18) were mapped to a corresponding set of unique consecutive integers. Using published HeLa karyotypic data [79], the mapping process accounted for over and under-represented chromosomal regions of the HeLa karyotype by increasing or decreasing the amount of integer space allocated to the corresponding human regions. Insertion locations were chosen by randomly selecting a genomic nucleotide (via its corresponding integer) from the total mapped set of sequence space using a uniform distribution. The insertion was recorded as occurring between the selected and subsequent genomic base. The sequence flanking the chosen location was subsequently extracted from the human genome and analyzed for repeat content with a local installation of RepeatMasker (default settings).

### Analysis of genome sequence features in Alu integration windows

The non-parametric Mann-Whitney-Wilcoxon test [80] implemented in the coin package version 1.0–18 of R [81] was used to assess whether the distributions of each of the nine genomic features (Table 4) were shifted left or right in the insert-containing (203) versus insert-free (2562) 1-Mb windows (windows were from [39]). From the original 226 Alu *de novo* inserts, nine were not assigned to any window (as some windows were removed due to gaps in the human genome assembly), and 14 windows contained two inserts each, resulting in a total of 203 1-Mb insert-containing windows hosting 217 Alu inserts, and 2562 insert-free windows. The null hypothesis of the test assumes that the distributions in both types of windows are the same, and a shift between the distributions will render a significant p-value (we analyzed all three possible alternative hypotheses; two-, left- and right-sided). For each predictor, we ordered all data ranking them independently of the groupings (insert-containing vs. insert-free) and computed the observed U statistic for the test. Next, we performed 10,000 random permutations of the data; in each, the insert-containing and insert-free labels of the 2765 windows were reshuffled as to produce randomized insert-containing and insert-free groups with the same sizes as the original (*i.e.*, 203 and 2562 windows, respectively), and the test statistics for each predictor were recomputed. Benchmarking the observed U statistics with the null distributions generated by the 10,000 random permutations allowed us to compute the empirical p-values. A Bonferroni correction for multiple testing was then applied to these p-values. Additionally, random subsets of the data (usually including 100 inserts at a time) were analyzed by the same procedure; similar results were obtained (data not shown).

### 3′ RACE of Alu rescue RNA

HeLa cells (4×10^6^/T75 flask) were transiently transfected with 10 µg of pBS-Ya5 rescue-A70Du using the Lipofectamine Plus (InVitrogen) following the manufacturer's protocol. Total RNA was harvested between 24 and 48 h post-transfection using the previously described protocol [82]. For the RT-PCR amplification, cDNA was generated by incubating approximately 1 µg of extracted RNA with the following primers: either unique2: 5′-AGGTTGTGTGTGCCAGTTACCTTGTT-3′, unique4: 5′-GCCAGTTACCTTGTTTTT-3′ (for cells transfected with pBS-Ya5rescue-A70Du) or the anchored oligo dT 5′-GCGAGCACAGAATTAATACGACTCACTATAGGTTTTTTTTTTTT-3′ (for cells transfected with pBS-Ya5rescue-A70D). The unique primers anneal to the unique region of the Alu RNA located between the A-tail and the RNA polymerase III terminator. The RNA-oligo mix was incubated with transcriptor reverse transcriptase (Roche Applied Science) at 65°C for 10 min following the manufacturer's recommended protocol. PCR amplification was performed for the Alu rescue samples with the same primer during cDNA generation or the primer to the anchor: 5′- GCGAGCACAGAATTAATACGACT-3′ and the FAtail230 primer: 5′- CTTATAAAACTTAAAACCTTAGAGGC-3′. PCR amplification was performed for 30 cycles of 20 s at 94°C, 30 s at 58°C and 60 s at 72°C, with a final cycle of 20 min at 72°C. PCR products were excised and extracted from 1% agarose gels using QIAquick gel extraction kit (Qiagen) and cloned for sequence analysis using the TOPO TA cloning kit (Invitrogen).

## Supporting Information

Figure S1 **Alu inserts lacking the characteristic features of retrotransposition are associated with genomic deletions or rearrangements.** A schematic representation of the recovered Alu inserts lacking the characteristic features of retrotransposition is shown. The Alu RNA (yellow) is reverse transcribed by the L1 ORF2p. It is thought that the homology between the Alu sequence of the cDNA helps drive recombination with the genomic Alu element present near the insertion site. The tagged Alu (orange box) that completed insertion by recombining with a genomic Alu (blue box) produces a chimeric Alu with the 5′ region matching the sequence of the genomic Alu and the 3′ region derived from the tagged Alu sequence. The small arrow represents the putative DNA nick of the top strand. No direct repeats (DR) are created by this type of insertion and deletions or rearrangements of the genomic sequence are observed. Six *de novo* Alu inserts (clones indicated) presented these features, representing 2.7% of the recovered Alus.
(PDF)

Figure S2 **PCR analysis of clone 57 pre-insertion site.** The top schematic shows the chromosomal flanks of the clone 57 insert as well as the human genome sequence reference assembly (hg19). The position and orientation of the PCR primers used in this analysis are indicated by arrows and are color coded to show pairings expected to generate amplicons, expected sizes are indicated. We used the following DNA templates for PCR reactions: A) untransfected HeLa DNA, B) Pooled colonies: DNA from transfected HeLa consisting of pooled G418^R^ colonies from which the Alu clone 57 insert was recovered, and C) Alu clone 57 plasmid DNA as a positive control for insert-specific amplicons. Our PCR analysis confirmed that the rearrangement did not pre-exist in the untransfected HeLa cells (no product from primer sets F1 -R1,-R2 or -R3 shown in red). The DNA from the transfected HeLa cells used to rescue clone 57 (pooled colonies) shows the presence of the rearrangement observed in the Alu clone 57 in addition to the intact genomic site. All PCR products were confirmed by sequencing. Our data suggests that the rearrangement observed is likely associated with the Alu insertion. m: 1 kb markers (sizes are indicated on the left). Primer sequences F1: 5′- GAAAACACACCCTATGCTAAATG-3′; R1: 5′-GGCACAAGGAACCAGTGTCATGG-3′; R2: 5′-TATAACTAACTCAGAAGACCAGG-3′; R3: 5′-GGCTTTAACCACTGTGAATCTTGG-3′; GF1: 5′-GAAAACACACCCTATGCTAAATG-3′; GR1: 5′-GTTAGTCATTTTTAACTTCGCG-3′; GF2: 5′-GCATGATGAGCCAGGAGTATGGTG-3′; GR2: 5′-CCACTTTATAACTAACTCAGAAGACC-3′.
(PDF)

Figure S3 **The A-tail expansions present in the** *de novo* **Alu inserts are not observed at the RNA level.** cDNA was generated by 3′RACE (RT-PCR amplification) of RNA from cells transfected with the Alu rescue vector and sequenced. Either an oligo dT primer or a primer annealing to the 3′end of the RNA were used. A sample of the cDNA sequences obtained is shown. The parental sequence of the tagged Alu is shown at the top. Only small expansion/contractions of A-tail sequence were observed (highlighted in gray). There is no evidence that changes at the RNA significantly contributed to the large adenosine expansions observed. Bold underline: inserted adenosine; Dashes: lost adenosines; Dots: identical sequences; Blank spaces were introduced for alignment purposes and the non-adenosine disruptions are shown for easier visual orientation.
(PDF)

Figure S4 **The A-tail expansions present in the** *de novo* **Alu inserts are not introduced by PCR amplification from a DNA template.** DNA from the Alu rescue vector was PCR amplified with primers flanking the A-tail sequence. PCR products from the amplification of the Alu rescue vector were cloned and sequenced. A sample of the sequences obtained is shown. The parental sequence of the tagged Alu is shown at the top. Only small expansion/contractions of A-tail sequence were observed (highlighted in gray). Overall, neither PCR, cloning or the sequencing procedure significantly contributed to the large A expansions observed. Bold underline: inserted adenosine; Dashes: lost adenosines; Dots: identical sequences; Blank spaces were introduced for alignment purposes and the non-adenosine disruptions are shown for easier visual orientation.
(PDF)

Figure S5 **The A-tail expansions present in the** *de novo* **Alu inserts are consistent between independently rescued sequences from the same Alu insert.** Examples of the sequences obtained from the repeated recovery of *de novo* Alu inserts (separate bacterial colonies and separate DNA preparations of the pooled G418^R^ colonies containing the tagged Alu inserts) are shown. The clone # corresponds to the reference name of the specific Alu insert used in Table S1 and Text S1. Names with letter and numbers represent the individual bacterial colony and miniprep sequenced. The top line represents the consensus sequence (cons). Dots: identical sequences. Variation in the length of the A-tail sequence was rarely observed (highlighted in gray), strongly supporting the conclusion that the recovery assay does not contribute to the Atail expansions observed.
(PDF)

Table S1 **** **a.** Genomic location and details of Alu inserts recovered. **b.** A-tail data of Alu inserts recovered. **c.** Genomic location and details of BC1 and B2 inserts recovered.
(XLSX)

Table S2 **Genomic features with non-significant difference in median values between Alu inserts containing versus the other windows.**
(PDF)

Text S1 **Sequences of the pre-insertion and post-insertion genomic sites of the** *de novo* **Alu inserts.**
(PDF)

Text S2 **Sequences of the pre-insertion and post-insertion genomic sites** **of the** *de novo* **BC1 or B2 inserts.**
(PDF)
