# Supplementary figures and images for: Rescuing Alu: Recovery of New Inserts Shows LINE-1 Preserves Alu Activity through A-Tail Expansion
Source: PLoS Genet. 2012 Aug 9;8(8):e1002842. doi: 10.1371/journal.pgen.1002842 (PMC3415434; doi:10.1371/journal.pgen.1002842)

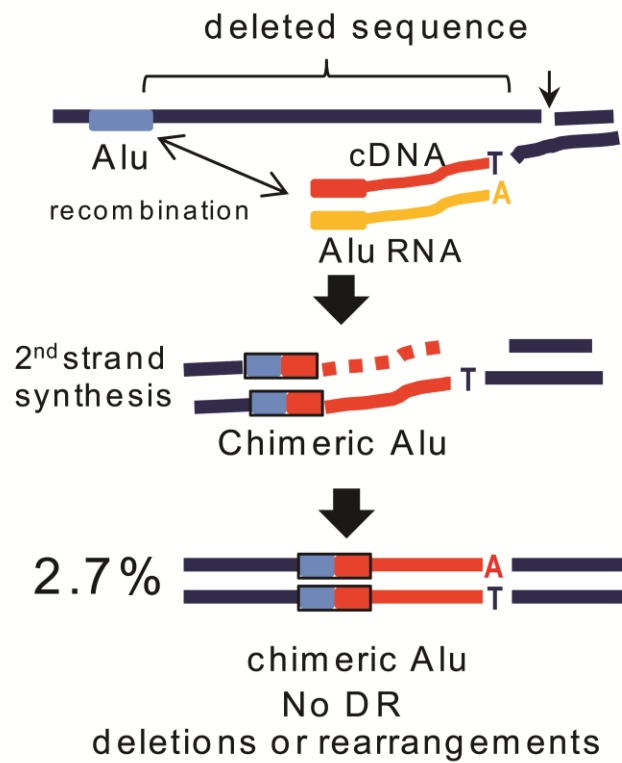

clones #: 57, 152, 185, 200, 239, 250

Supplement: Figure S1 — Alu inserts lacking the characteristic features of retrotransposition are associated with genomic deletions or rearrangements. A schematic representation of the recovered Alu inserts lacking the characteristic features of retrotransposition is shown. The Alu RNA (yellow) is reverse transcribed by the L1 ORF2p. It is thought that the homology between the Alu sequence of the cDNA helps drive recombination with the genomic Alu element present near the insertion site. The tagged Alu (orange box) that completed insertion by recombining with a genomic Alu (blue box) produces a chimeric Alu with the 5′ region matching the sequence of the genomic Alu and the 3′ region derived from the tagged Alu sequence. The small arrow represents the putative DNA nick of the top strand. No direct repeats (DR) are created by this type of insertion and deletions or rearrangements of the genomic sequence are observed. Six de novo Alu inserts (clones indicated) presented these features, representing 2.7% of the recovered Alus. (PDF) [file pgen.1002842.s001.pdf]

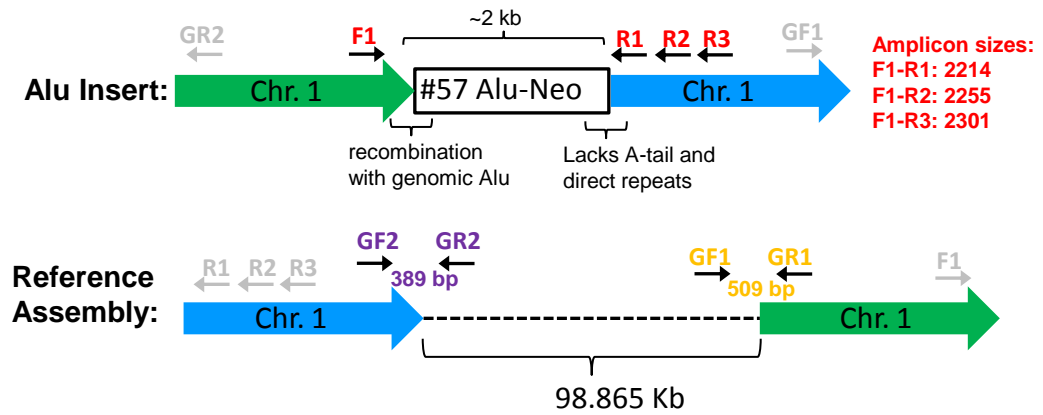

### A) HeLa (pre-transfection)

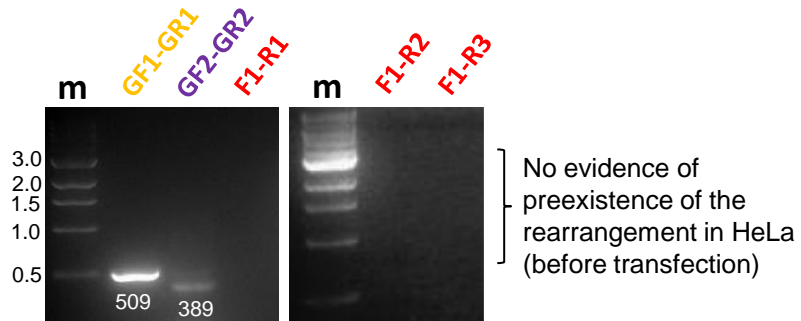

### B) Pooled colonies (post-transfection)

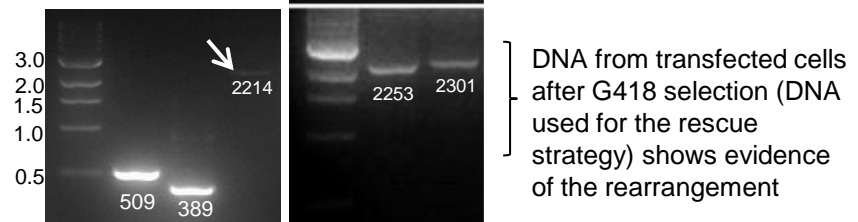

### C) Clone 57

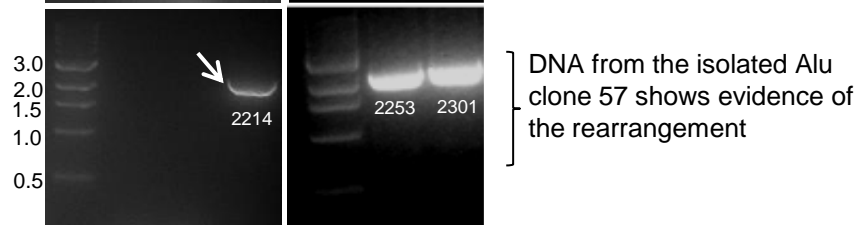

Supplement: Figure S2 — PCR analysis of clone 57 pre-insertion site. The top schematic shows the chromosomal flanks of the clone 57 insert as well as the human genome sequence reference assembly (hg19). The position and orientation of the PCR primers used in this analysis are indicated by arrows and are color coded to show pairings expected to generate amplicons, expected sizes are indicated. We used the following DNA templates for PCR reactions: A) untransfected HeLa DNA, B) Pooled colonies: DNA from transfected HeLa consisting of pooled G418R colonies from which the Alu clone 57 insert was recovered, and C) Alu clone 57 plasmid DNA as a positive control for insert-specific amplicons. Our PCR analysis confirmed that the rearrangement did not pre-exist in the untransfected HeLa cells (no product from primer sets F1 -R1,-R2 or -R3 shown in red). The DNA from the transfected HeLa cells used to rescue clone 57 (pooled colonies) shows the presence of the rearrangement observed in the Alu clone 57 in addition to the intact genomic site. All PCR products were confirmed by sequencing. Our data suggests that the rearrangement observed is likely associated with the Alu insertion. m: 1 kb markers (sizes are indicated on the left). Primer sequences F1: 5′- GAAAACACACCCTATGCTAAATG-3′; R1: 5′-GGCACAAGGAACCAGTGTCATGG-3′; R2: 5′-TATAACTAACTCAGAAGACCAGG-3′; R3: 5′-GGCTTTAACCACTGTGAATCTTGG-3′; GF1: 5′-GAAAACACACCCTATGCTAAATG-3′; GR1: 5′-GTTAGTCATTTTTAACTTCGCG-3′; GF2: 5′-GCATGATGAGCCAGGAGTATGGTG-3′; GR2: 5′-CCACTTTATAACTAACTCAGAAGACC-3′. (PDF) [file pgen.1002842.s002.pdf]
