# Supplementary material for: Rescuing Alu: Recovery of New Inserts Shows LINE-1 Preserves Alu Activity through A-Tail Expansion
Source: PLoS Genet. 2012 Aug 9;8(8):e1002842. doi: 10.1371/journal.pgen.1002842 (PMC3415434; doi:10.1371/journal.pgen.1002842)
Supplement: Figure S3 — The A-tail expansions present in the de novo Alu inserts are not observed at the RNA level. cDNA was generated by 3′RACE (RT-PCR amplification) of RNA from cells transfected with the Alu rescue vector and sequenced. Either an oligo dT primer or a primer annealing to the 3′end of the RNA were used. A sample of the cDNA sequences obtained is shown. The parental sequence of the tagged Alu is shown at the top. Only small expansion/contractions of A-tail sequence were observed (highlighted in gray). There is no evidence that changes at the RNA significantly contributed to the large adenosine expansions observed. Bold underline: inserted adenosine; Dashes: lost adenosines; Dots: identical sequences; Blank spaces were introduced for alignment purposes and the non-adenosine disruptions are shown for easier visual orientation. (PDF) [file pgen.1002842.s003.pdf]

```
DNA:    AAAAAAAAAAAAAAAAA CATTACAAAAAAAAAAAAAAAAAA  GAAAAAAAAAAAAAAAAA CACACAAAAAAAAAAAAAAAAAA
MP#1:   ................. CATTAC..................AAG................- CACAC..............
MP#2:   ................. CATTAC..................  G................- CACAC.................
MP#3:   ................. CATTAC..................  G................- CACAC.................
MP#4:   ................. CATTAC..................AAA................- CACAC..................
MP#5:   ................. CATTAC.................-  G................. CACAC.................
MP#6:   ................. CATTAC..................  G.................ACACGC.................
MP#7:   ................- CATTAC..................  G................. CACAC................
MP#8:   ..............--- CATTAC.................
MP#9:   .................ACATTAC..................
MP#10:  .................ACATTAC...................
MP#11:  ................- CATTAC.................
MP#12:  ...............
```

MP#8–MP#12: Internal priming by the oligo dT used in RT step
