# Supplementary material for: Rescuing Alu: Recovery of New Inserts Shows LINE-1 Preserves Alu Activity through A-Tail Expansion
Source: PLoS Genet. 2012 Aug 9;8(8):e1002842. doi: 10.1371/journal.pgen.1002842 (PMC3415434; doi:10.1371/journal.pgen.1002842)
Supplement: Figure S4 — The A-tail expansions present in the de novo Alu inserts are not introduced by PCR amplification from a DNA template. DNA from the Alu rescue vector was PCR amplified with primers flanking the A-tail sequence. PCR products from the amplification of the Alu rescue vector were cloned and sequenced. A sample of the sequences obtained is shown. The parental sequence of the tagged Alu is shown at the top. Only small expansion/contractions of A-tail sequence were observed (highlighted in gray). Overall, neither PCR, cloning or the sequencing procedure significantly contributed to the large A expansions observed. Bold underline: inserted adenosine; Dashes: lost adenosines; Dots: identical sequences; Blank spaces were introduced for alignment purposes and the non-adenosine disruptions are shown for easier visual orientation. (PDF) [file pgen.1002842.s004.pdf]

```
DNA:   AAAAAAAAAAAAAAAAA CATTACAAAAAAAAAAAAAAAAAA   GAAAAAAAAAAAAAAAAA  CACACAAAAAAAAAAAAAAAAAA
MP#1:  .................ACATTAC..................   G................AAACACAC..................A
MP#2:  ................- CATTAC..................AAAG.................  CACAC..................A
MP#3:  ................. CATTAC..................AA G................-  CACAC..................
MP#4:  .................ACATTAC.................-   G................-  CACAC.................
MP#5:  ................- CATTAC.................-   G.................A CACAC................
MP#6:  .................ACATTAC..................   G.................AACACAC..................A
MP#7:  ................. CATTAC..................   G.................  CACAC................
```
