# Supplementary material for: Rescuing Alu: Recovery of New Inserts Shows LINE-1 Preserves Alu Activity through A-Tail Expansion
Source: PLoS Genet. 2012 Aug 9;8(8):e1002842. doi: 10.1371/journal.pgen.1002842 (PMC3415434; doi:10.1371/journal.pgen.1002842)
Supplement: Figure S5 — The A-tail expansions present in the de novo Alu inserts are consistent between independently rescued sequences from the same Alu insert. Examples of the sequences obtained from the repeated recovery of de novo Alu inserts (separate bacterial colonies and separate DNA preparations of the pooled G418R colonies containing the tagged Alu inserts) are shown. The clone # corresponds to the reference name of the specific Alu insert used in Table S1 and Text S1. Names with letter and numbers represent the individual bacterial colony and miniprep sequenced. The top line represents the consensus sequence (cons). Dots: identical sequences. Variation in the length of the A-tail sequence was rarely observed (highlighted in gray), strongly supporting the conclusion that the recovery assay does not contribute to the Atail expansions observed. (PDF) [file pgen.1002842.s005.pdf]

Clone 23:

cons: AAAAAAAAAAAAAAAAAAAAAAAAAAAAAAAAAAAAAAAAAAAAAAAAAAAAAAAAAAAAAAAAAAAAAAAAAAA
JK25: ....
L2: ....

cons: AAAAAAAAAAAAAAAAAAAAAAAAAAAAAAAAAAAAAA
JK25: ....
L2: ....

Clone 32:

cons: AAAAAAAAAAAAAAAAAAAAA**CATTAC**AAAAAAAAAAAAAAAAAAA**G**AAAAAAAAAAAAAAAAAAAAAAAAAAAAAAAAAAAAAAAAAAA
R1: ....
R2: ....

Clone 54:

cons: AAAAAAAAAAAAAAAAAAA**CATTAC**AAAAAAAAAAAAAAAAAAAAAAAAAAAAAAAA
K23: ....
JK24: ....

Clone 58:

cons: AAAAAAAAAAAAAAAAAAAAAA**CATTAC**AAAAAAAAAAAAAAAAAAAAAAAAAAAAAAAAAAAAAAAAAAAAAAAAAAAAAAAAAAA
K21: ....
K32: ....

Clone 72:

cons: AAAAAAAAAAAAAAAAA**CATTAC**AAAAAAAAAAAAAAAAAAAAA**G**AAAAAAAAAAAAAAAAAAAAAAAAAAAAAAAAAAAAAAAAAA
E10: ....
E43: ....
F25: ....
N6: ....

cons: A-**CACAC**AAAAAAAAAAAAAAAAAAA**C**AAAAAAAAAAAAAAAAAAAAAAAAAAAAAAAAAAAAAA
E10: .A...
E43: .-...
F25: .-...
N6: .-...

Clone 77:

```
cons: AAAAAAAAAAAAAAAAAAAAACATTACAAAAAAAAAAAAAAAAAAAAAAAAAGAAAAAAAAAAAAAAAAAAAAAAAAAAAAAAAAAAAA
 G59: .......................................................................................
G146: .......................................................................................
G147: .......................................................................................
G148: .......................................................................................
G149: .......................................................................................
G185: .......................................................................................

cons: AGAAAAAAAAAAAAAAAAAAAAAA
 G59: ........................
G146: ........................
G147: ........................
G148: ........................
G149: ........................
G185: ........................
```

Clone 80:

```
cons: AAAAAAAAAAAAAAAAAAAAACATTACAAAAAAAAAAAAAAAAAAAAAAAAAAAAAAAAAAAAAAAAAAAAAAAAAAAAAAAAAAAAGAAAAAAA
 D16: ...............................................................................................
 E11: ...............................................................................................
```
