# Supplementary material for: Rescuing Alu: Recovery of New Inserts Shows LINE-1 Preserves Alu Activity through A-Tail Expansion
Source: PLoS Genet. 2012 Aug 9;8(8):e1002842. doi: 10.1371/journal.pgen.1002842 (PMC3415434; doi:10.1371/journal.pgen.1002842)
Supplement: Table S2 — Genomic features with non-significant difference in median values between Alu inserts containing versus the other windows. (PDF) [file pgen.1002842.s007.pdf]

Table S2. Genomic features with non-significant differences in their distributions between Alu insert containing vs. the other windows (203 windows with integrations)

Genomic feature	Medians of the Integration windows	Medians of the Non-Integration windows	Absolute difference in medians	Z-value	p values of MWW test[a]
Decode female [b]	1.5270	1.3264	0.2006	1.97	0.0472
Decode male [b]	0.6204	0.5974	0.023	1.09	0.2742
L1 target [c]	1189.5	1249	59.5	-0.79	0.0107
Recombination hotspots [d]	9	9	0	-2.73	0.4282

*p = 0.0056 is the cut-off of significance after Bonferroni correction.
a: Two-sided hypothesis test.
b: Sites associated with sex-specific recombination in the female/male germline [1].
c: TTTTAA canonical motif characteristic of target primed reverse transcription of retroelements [2,3].
d: computationally predicted hotspots of recombination using linkage disequilibrium among SNPs [4].

Details of the genomic features evaluated in Table 2 and Table S2:

Category	Predictor	Explanation	Calculation	Reference
Genome landscape	L1 target (TTTTAA)	Motif characteristic of target primed reverse transcription	count per window	[2,3]
	SINE count (Alu and Mir)	All SINE elements in the genome	count per window	[5]
	GC content	Reflects local base composition	fraction of G and C bases per window	[5]
Recombination	Recombination hotspots	Computationally predicted hotspots of recombination using linkage disequilibrium among SNPs	count per window	[4]
	Decode female/ Decode male	Sex-specific recombination in female or male germline	rate per window	[1]
	Genome instability 13-mer frequency	Motif associated with recruiting crossover events at recombination hotspots and cluster at NAHR breakpoints	ratio observed/expected frequency per window	[6] [7]
Natural selection	Gene content	Protein coding genes likely reflect local natural selection pressure	fraction of bases per window	[5]
	Most conserved element density	Regions likely to contain functional elements and reflect local natural selection pressure	count per window	[8]

Reference List

1. Kong A, Gudbjartsson DF, Sainz J, Jonsdottir GM, Gudjonsson SA et al. (2002) A high-resolution recombination map of the human genome. Nat Genet 31: 241-247. 10.1038/ng917 [doi];ng917 [pii].

2. Jurka J (1997) Sequence patterns indicate an enzymatic involvement in integration of mammalian retroposons. Proc Natl Acad Sci U S A 94: 1872-1877.

3. Cost GJ, Feng Q, Jacquier A, Boeke JD (2002) Human L1 element target-primed reverse transcription in vitro. Embo J 21: 5899-5910.

4. Myers S, Bottolo L, Freeman C, McVean G, Donnelly P (2005) A fine-scale map of recombination rates and hotspots across the human genome. Science 310: 321-324. 310/5746/321 [pii];10.1126/science.1117196 [doi].

5. Kvikstad EM, Makova KD (2010) The (r)evolution of SINE versus LINE distributions in primate genomes: sex chromosomes are important. Genome Res 20: 600-613. gr.099044.109 [pii];10.1101/gr.099044.109 [doi].

6. Myers S, Freeman C, Auton A, Donnelly P, McVean G (2008) A common sequence motif associated with recombination hot spots and genome instability in humans. Nat Genet 40: 1124-1129. 10.1038/ng.213 [doi].

7. McVean G (2010) What drives recombination hotspots to repeat DNA in humans? Philos Trans R Soc Lond B Biol Sci 365: 1213-1218. 365/1544/1213 [pii];10.1098/rstb.2009.0299 [doi].

8. Siepel A, Bejerano G, Pedersen JS, Hinrichs AS, Hou M et al. (2005) Evolutionarily conserved elements in vertebrate, insect, worm, and yeast genomes. Genome Res 15: 1034-1050. gr.3715005 [pii];10.1101/gr.3715005 [doi].
