# Supplementary material for: Rescuing Alu: Recovery of New Inserts Shows LINE-1 Preserves Alu Activity through A-Tail Expansion
Source: PLoS Genet. 2012 Aug 9;8(8):e1002842. doi: 10.1371/journal.pgen.1002842 (PMC3415434; doi:10.1371/journal.pgen.1002842)
Supplement: Text S1 — Sequences of the pre-insertion and post-insertion genomic sites of the de novo Alu inserts. (PDF) [file pgen.1002842.s008.pdf]

ALU RESCUES

CLONE 1

[P12, P13, Q21, Q30, Q33, Q38, R32, R40, S16, T1, T29] these correspond to the identification labels assigned to the independently generated bacterial colonies that were recovered for *de novo* Alu insert Clone 1. The *de novo* Alu insert Clone 1 was independently recovered 11 times.

Driver: L1
Plasmid: AlurescueA70D $A_{17}CATTACA_{18}GA_{17}CACACA_{18}$ (T)
Chromosome: 5
5' position: 39,107,125
Strand: minus
DR: AAAAACATTCCACA
ENDOsite: TTTT/AA

Empty site:

ATTGGCATGTTATTGGGTATCAAGAAAATTAATGCACAAAACCACTTATTATCATTTGTTATGAAATCCC
AATTATCTTTACAAAGTGTTTAAAGTTTGAACATAGAAAATAATCTCTCTGCTTAATTGTTAACTCAGAA
GACTACATTAGTGAGATGTAAGAATTATTAAATATTCCATTTCCGCTTTGGCTACAATTATGAAGAAGTT
GAAGGTACTTCTTTTAGACCACCAGTAAATAATCCTCCTTCAAAAAATAAAAATAAAAGAAAAAGGAAAA
TCATTCAGGAAGAAATGACCTGTCTAAAAAAACCTAAGGAAGAATAATAATATAAGAAAGGAAATTT↑**AAA**
AACATTCCACA↓AGAAGAAAAATTATTGTTTATACTTCTACTTATGGTTATATCTTATATTCTCTATTCAA
GTGACCTGTCTTTTAAAAAGGCAGTGCTGTCTTACCTCTTGCTAGTGGGTTAAATGTTTTCAAAAATTAT
AGCAGTAGTAGAAGTTTTGTATAAAATTTGTCCTTATTTGTTAATTGTATATAAATGTTAATTATTTGAT
ACGAATGTTATGCATTTAGTATGCACATTGAAGTCTAAACTGTAGAAGAGTCTAAAACAAGTTCTCTTTT

Filled site:

TCATTCAGGAAGAAATGACCTGTCTAAAAAAACCTAAGGAAGAATAATAATATAAGAAAGGAAATTTAAAAA
CATTCCACA**GGCCGGGCGCGGTGGCTCACGCCTGTAATCCCAGCACTTTGGGAGGCCGAGGCGGGC**
GGATCACGAGGTCAGGAGATCGAGACCATCCTGGCTAACACGGTGAAACCCCGTCTCTACTAAAAA
AAAATACAAAAAATTAGCCGGGCGTGGTAGCGGGCGCCTGTAGTCCCAGCTACTCGGGAGGCTGAG
GCAGGGGAATGGCGTGAACCCGGGAGGCGGAGCTTGCAGTGAGCCGAGATCCCGCC…[NEOcasse
tte]*AAAAAAAAAAAAAAAAAAAACATTACAAAAAAAAAAAAAAAAAAAAGAAAAAAAAAAAAAAAAAAAAC*
ACACAAAAAAAAAAAAAAAAAAAAAAAAAAAAAAAAACATTCCACAAGAAGAAAAATTATTGTTTATACTTC
TACTTATGGTTATATCTTATATTCTCTATTCAAGTGACCTGTCTTTTAAAAAGGCAGTGCTGTCTTACCTCT
TGC

No repetitive sequences in the immediate proximity.

CLONE 2

[Green 89, Brown 99, C37, C43, G199, G200, K12, K15, K37, N1, N9, N11,N17, P28, P37, Q15, R29, R49, S28, U5]

Driver: ORF2
Plasmid: AlurescueA70Du $A_{17}CATTACA_{18}GA_{17}CACACA_{18}$ (T)
Chromosome: 9
5' position: 97,894,942
Strand: plus
DR: AAAAGTGAACGGGTT
ENDOsite: TTTT/GA

Empty site:

TGAAAAGTTTCAGTCTGTACAGTAAATGACAATCTAGGGCTAATACCTCACTTGATGTAGGAGCTACCAA
CCGTGGCAGACAGCATGACTCGACAGCAGTCCAGTCCACCGCACGGAAGCCAAAGGGGAAGCCTC↑**AAAAG**
TGAACGGGTT↓TTGAAAACTTTTCTTTTATTTTAGATGTTAAATACATACATAGTCATTTGGAACTCAGGT
AATAAGAGACTAATCCCTCATTATTCATGGTCTTAATGGAAAATATCAGACCTGCGAGTCCAAGAGGCCA
AAGAACATTTGGGAAGGTCATTTCACTTTGTACAACCTGTACCACACTTTTATCTTTTGTTTTTGTTACA
TTTTGGTAACTATAGAATTTCCAGGACTCATTCTGCATAGTCCCAAGAGAGAGAAATATAATTAACAGTG

Filled site:

TGAAAAGTTTCAGTCTGTACAGTAAATGACAATCTAGGGCTAATACCTCACTTGATGTAGGAGCTACCAA
CCGTGGCAGACAGCATGACTCGACAGCAGTCCAGTCCACCGCACGGAAGCCAAAGGGGAAGCCTCAAAAG
TGAACGGGTT**GGCCGGGCGCGGTGGCTCACGCCTGTAATCCCAGCACTTTGGGAGGCCGAGGCGGGC**
GGATCACGAGGTCAGGAGATCGAGACCATCCTGGCTAACACGGTGAAACCCCGTCTCTACTAAAAA
AAAATACAAAAAATTAGCCGGGCGTGGTAGCGGGCGCCTGTAGTCCCAGCTACTCGGGAGGCTGAG
GCAGGGGAATGGCGTGAACCCGGGAGGCGGAGCTTGCAGTGAGCCGAGATCCCGCC… **[NEOcasse**
tte]***AAAAAAAAAAAAAAAAAAACATTACAAAAAAAAAAAAAAAAAAAAAAAAAAAAAAAAAAATA***
AAAAAAAAAAAATAAAAAAAAAAAAAAAAAAAAAAAAAAAAAAAAAAAGTGAACGGGTTTGAAAACT
TTTCTTTTATTTTAGATGTTAAATACATACATAGTCATTTGGAACTCAGGTAATAAGAGACTAATCCCTCAT
TATTCATGGTCTTAATGGAAAATATCAGACCTGCGAGTCCAAGAGGCCA

No repetitive sequences in the immediate proximity.

CLONE 3

[P34, Q20, S3, S17, S18, T7]

Driver: L1
Plasmid: AlurescueA70D $A_{17}CATTACA_{18}GA_{17}CACACA_{18}$ (T)
Chromosome: 17
5’ position: 19,921,248
Strand: plus
DR: AAAAACTTGGTGATTCT
ENDOsite: TTTT/AT

Empty site:

AGTCAGACCAAGAACCCACCAATTCCGGACACAAAAGGTGTTCCCACCATTCCTGTCCTCTTACTTCTCTCG
CCTTACAAGTAGGTAACCATTGTTGTTAGTTACTTATGTACCTTCCAAAGTTTCTTCATGCAAATACAAGCA
AATATGAATCAGATTCTCCATTTCTCCTTTTTTGCACAAATAGTAGCATTATTCTCTATCTGGTCTTCATCC
TGTGTTTTTATTTCTTGGTATATCACAAAGATCGTCACATACCAACTGCTTGCTCTTTATAATGTCCCCTTA
GGTCTTCCCAAGAATATCTAATAATTTCTTCTATGTGCTCTAGTAATTATCTTTGCTTGGGTTTGTGTTCTG
TTGATTCAGTGTCCCTGTTTCATCATACTGAAT↑**AAAAACTTGGTGATTCT**↓TTATGTGTTCATCTTCGAAG
TTTGAGAATTCCTTTTTTCCTGTCTTACTAGGCGTAGCGTGCCTCCTGTGATTGTTGAAGAGCTGTGGTGTG
CTGCTGAGTGGCGTGTGTATTCCATGTGAGGGGAAGGGTCCAACAGTCCTGGTCATTCAGACTGCAGTTCCC
CAGGTAATCACCTTGTCAGGTTCTACCTCTGCTGTTCTATCTGCTGTTACAAGTCTGTTCCTGGAAACCTGT

Filled site:

TGTCCCCTTAGGTCTTCCCAAGAATATCTAATAATTTCTTCTATGTGCTCTAGTAATTATCTTTGCTTGGGT
TTGTGTTCTGTTGATTCAGTGTCCCTGTTTCATCATACTGAATAAAAACTTGGTGATTCT**GGCCGGGCGCG**
GTGGCTCACGCCTGTAATCCCAGCACTTTGGGAGGCCGAGGCGGGCGGATCACGAGGTCAGGAGAT
CGAGACCATCCTGGCTAACACGGTGAAACCCCGTCTCTACTAAAAAAAAATACAAAAAATTAGCCG
GGCGTGGTAGCGGGCGCCTGTAGTCCCAGCTACTCGGGAGGCTGAGGCAGGGGAATGGCGTGAACC
CGGGAGGCGGAGCTTGCAGTGAGCCGAGATCCCGCC…[NEOcassette]*AAAAAAAAAAAAAAAA*
AAAAAAAAAAAAAAAAAAAAAAAAAAAAAAAAACATTACAAAAAACTTGGTGATTCTTTATGTGTTC
ATCTTCGAAGTTTGAGAATTCCTTTTTTCCTGTCTTACTAGGCGTAGCGTGCCTCCTGTGATTGTTGAAGAG
CTGTGGTGTGCTGCTGAGTGGCGTGTGTATTCCATGTGAGGGGAAGGGTCCAACAGTCCTGGTCATTCAGAC

LTR and L1 at 5’.

CLONE 4

[R37, R43, S13, U10]

Driver: L1
Plasmid: AlurescueA70D $A_{17}CATTACA_{18}GA_{17}CACACA_{18}$ (T)
Chromosome: 20
5’ position: 7,130,649
Strand: minus
DR: AGAAAATCCACTCACTT
ENDOsite: TTCT/AA

Empty site:

AAGAAATTTCCACTCTAACACTCATTTTCTGCCACCATAATTTTTCCAGGGTCAGATGCTTAAGGCAATGTT
CTAAGTTCATAATTAAGTTTAGTTGCATGCACAATTTGGGTAGTTAAGTAATTACATGATTTGCACATGCAA
ATACTGCAATCATTGGCACATAAAAATAACGTCCTTTAAACATCTGGGCCTGATGGAGGACAAATGGTCAGG
AAAATCAGAATTTCTGTGGTTCCCCAAAGGGATCACTATAAACAGGAAACACTACAATCTGTTCTCAGACAT
TT↑**AGAAAATCCACTCACTT**↓CCCAAAGGGATCACTATAAACAGGAAACACTACAATCTGTTCTCAGACATT
TAGAAAATCCACTCACTTTAACCTAGAGAAAAGAAGCAACATGGAGGCATTCTAACTTCGACTTCTCCTCTC
CAAAACTATCCAGGTACCGAATAAGCTATTTGTAACCATGAAATGTCTGAACACTAATCAGAACATTTGAGT
GTAAAATACAGTCCTACCACTGAAGAGGTTGAAAAGTTTTGAACAAATCACTTAATCCCTGAGTCTCCTAAT
ATTAATCTCTAAAATAGGAATAATAACAATATCAACACTATAGGATTGAAAAGAGAAATAAATGAGATAAAT

Filled site:

AAGAAATTTCCACTCTAACACTCATTTTCTGCCACCATAATTTTTCCAGGGTCAGATGCTTAAGGCAATGTT
CTAAGTTCATAATTAAGTTTAGTTGCATGCACAATTTGGGTAGTTAAGTAATTACATGATTTGCACATGCAA
ATACTGCAATCATTGGCACATAAAAATAACGTCCTTTAAACATCTGGGCCTGATGGAGGACAAATGGTCAGG
AAAATCAGAATTTCTGTGGTTCCCCAAAGGGATCACTATAAACAGGAAACACTACAATCTGTTCTCAGACAT
TTAGAAAATCCACTCACTT**GGCCGGGCGCGGTGGCTCACGCCTGTAATCCCAGCACTTTGGGAGGCC**
GAGGCGGGCGGATCACGAGGTCAGGAGATCGAGACCATCCTGGCTAACACGGTGAAACCCCGTCTC
TACTAAAAAAAAATACAAAAAATTAGCCGGGCGTGGTAGCGGGCGCCTGTGGTCCCAGCTACTCGG
GAGGCTGAGGCAGGGGAATGGCGTGAACCCGGGAGGCGGAGCTTGCAGTGAGCCGAGATCCCGCCA
CTGCA…[NEOcassette]*AAAAAAAAAAAAAAAAAAAAAAAAAAAAAAAAAAAAAACATTACAAA*
AAAAAAAAAAAAAAAAAAAGAAAAAAAAAAAAAAAAAAAAAAAAAAAAAGAAAATCCACTCACTTCC
CAAAGGGATCACTATAAACAGGAAACACTACAATCTGTTCTCAGACATTTAGAAAATCCACTCACTTTAACC
TAGAGAAAAGAAGCAACATGGAGGCATTCTAACTTCGACTTCTCCTCTCCAAAACTATCCAGGTACCGAATA
AGCTATTTGTAACCATGAAATGTCTGAACAC

A MIR at the 3’.

CLONE 5

[P4, P21, P26, P19, P18 Q3, Q7]

Driver: ORF2
Plasmid: AlurescueA70D $A_{17}CATTACA_{18}GA_{17}CACACA_{18}$ (T)
Chromosome: 12
5' position: 90,412,031
Strand: minus
DR: AAAACTAACAGGAGG
ENDOsite: TTTT/AA

Empty site:

GCAGTAGATACTAGTAGAATACTCACGTGACTTTAATGACTACCTAAGGTCTATGCCACCTAAGATGCATCA
TGACACAAACATAAGGGGTGGGAGAGAGATATAATTATAATCTTCAGGTGTTTAGATGCATTTTCTTGTGTG
CAGCTGCTATTATCTCTACTTATCATTGATGTGTCATTTTATATTCTGGCAGCAATGATTATTCCTAATTAT
ATATGGGAAAATAAATTGTAATATGTATTTAATACAAACACTCTGTCATTTGGGTAGTTATGTCAAACGGTT
AATCATGTTT↑**AAAACTAACAGGAGG**↓ACAGATTAGGGGGCAAAAATATGTTATGAAAAGACAACCCTCTTT
TTCCATAGTCTTGATTTAAAATTTAACCTAAAGGTTAGAAAAATGGTTTCAAGACGCATATTAGTATTTTTA
CCAAAGAACAGTTTCTCCTTGGAACATAAAGAATTCTTTTGAATCAGAATATCTCTGCAAACAACAGTCTCT
TCCTTGTGAGACTCGTCTCTCATGTTCAACCCCTTACTTCTGCCCCATGGTAGGACAAGGCAAAAGAATCCA

Filled site:

GCAGTAGATACTAGTAGAATACTCACGTGACTTTAATGACTACCTAAGGTCTATGCCACCTAAGATGCATCA
TGACACAAACATAAGGGGTGGGAGAGAGATATAATTATAATCTTCAGGTGTTTAGATGCATTTTCTTGTGTG
CAGCTGCTATTATCTCTACTTATCATTGATGTGTCATTTTATATTCTGGCAGCAATGATTATTCCTAATTAT
ATATGGGAAAATAAATTGTAATATGTATTTAATACAAACACTCTGTCATTTGGGTAGTTATGTCAAACGGTT
AATCATGTTTAAAACTAACAGGAGG**GGCCGGGCGCGGTGGCTCACGCCTGTAATCCCAGCACTTTGGG**
AGGCCGAGGCGGGCGGATCACGAGGTCAGGAGATCGAGACCATCCTGGCTAACACGGTGAAACCCC
GTCTCTACTAAAAAAAATACAAAAAAATTAGCCGGGCGTGGTAGCGGGCGCCTGTGGTCCCAGCTA
CTCGGGAGGCTGAGGCAGGGGAATGGCGTGAACCCGGGAGGCGGAGCTTGCAGTGAGCCGAGATCC
CGCCACTGCA…[NEOcassette]*AAAAAAAAAAAAAAAAAAAAAACATTACAAAAAAAAAAAAAA*
AAAAAAAAAAAAAAAAAAAAAAAGAAAAAAAAAAGAAAAAAACTAACAGGAGGACAGATTAGGGGGCA
AAAATATGTTATGAAAAGACAACCCTCTTTTTCCATAGTCTTGATTTAAAATTTAACCTAAAGGTTAGAAAA
ATGGTTTCAAGACGCATATTAGTATTTTTACCAAAGAACAGTTTCTCCTTGGAACATAAAGAATTCTTTTGA
ATCAGAATATCTCTGCAAACAACAGTCTCT

No repetitive sequences in the immediate proximity.

CLONE 6

[M18 ,M24, M52, M68, R5, R27b, S19, S25]

Driver: L1
Plasmid: AlurescueA70D   A$_{17}$CATTACA$_{18}$GA$_{17}$CACACA$_{18}$ (T)
Chromosome: 5
5' position: 45,784,963
Strand: minus
DR: AATTGAATGCAGAT
ENDOsite: AATT/AA

Empty site:

TTCACAGAAGCAAGAAAATGAAAATATATTTGCTTGCTTAAAGTGAAATACCCCTAGATATGTTACAGGTAG
TTAGACATGTATGAGCAGGACAGAAGAAGACTCTCCCCCACCTACTAGGAATGTTGGGTGATGGCTTGGCAA
TTACCACATTGCCTCTCTAAATGTGATAAGTTGGCAGCTAGCACCAGGGAGAGGCTATTTCTTGATGGTCCA
CACCAGTTGCACTAAAGTGTT↑**AATTGAATGCAGAT**↓ATGGTCCACACCAGTTGCACTAAAGTGTTAATTGA
ATGCAGATGCCAGGGAGAAGCAACTTCCTGGGTATGTGCATTAAGAGACAAAATGGTGGAATATGACCTTCC
AGGGGTACTTCACCAGGAAAGGGAGGAAAAGCCTCAGATGGGCGTGCCTACAACTTCCTAAACACACTGTGC
CTGCTCATCTCCTAAGGGTAAGGAGGGAACTGCACATGTGAGCAGCTCACCCTAAGGGAAGAATCATGGGAA

Filled site:

TTCACAGAAGCAAGAAAATGAAAATATATTTGCTTGCTTAAAGTGAAATACCCCTAGATATGTTACAGGTAG
TTAGACATGTATGAGCAGGACAGAAGAAGACTCTCCCCCACCTACTAGGAATGTTGGGTGATGGCTTGGCAA
TTACCACATTGCCTCTCTAAATGTGATAAGTTGGCAGCTAGCACCAGGGAGAGGCTATTTCTTGATGGTCCA
CACCAGTTGCACTAAAGTGTTAATTGAATGCAGAT**GGCCGGGCGCGGTGGCTCACGCCTGTAATCCCAG
CACTTTGGGAGGCCGAGGCGGGCGGATCACGAGGTCAGGAGATCGAGACCATCCTGGCTAACACGG
TGAAACCCCGTCTCTACTAAAAAAAAAATACAAAAAATTAGCCGGGCGTGGTAGCGGGCGCCTGTGG
TCCCAGCTACTCGGGAGGCTGAGGCAGGGGAATGGCGTGAACCCGGGAGGCGGAGCTTGCAGTGAG
CCGAGATCCCGCCACTGCA…[NEOcassette]*AAAAAAAAAAAAAAAAAAAAAAAAAAAAACATT
ACAAAAAAAAAAAAAAAAAAAAAAAAAAAAAAAAAAAAAAAAAAAAAAAAAAAAAAAAAAAAAAAA
AAAAAAAAAAAAAAAAAAAAAAAAAAAAAA***TTGAATGCAGATATGGTCCACACCAGTTGCACTAAAGTG
TTAATTGAATGCAGATGCCAGGGAGAAGCAACTTCCTGGGTATGTGCATTAAGAGACAAAATGGTGGAATAT
GACCTTCCAGGGGTACTTCACCAGGAAAGGGAGGAAAAGCCTCAGATGGGCGTGCCTACAACTTCCTAAACA
CACTGTGC

LTRs (ERVs).

CLONE 8

[P15, Q41]

Driver: ORF2
Plasmid: AlurescueA70D $A_{17}CATTACA_{18}GA_{17}CACACA_{18}$ (T)
Chromosome: 6
5’ position: 75,863,870
Strand: minus
DR: AAGAGTAATATA
ENDOsite: TCTT/AA

Empty site:

ACAGGCTATATATATACTTTAACATTTTAGGGCTGGTACCACCTATAAAGTAAATGTTTTTGGAATGTTTGA
TGGAGGAGAAAGCTCACCACTTGTTGGACAAGAAATGACAACCCTTTCCGACACAACTGTTATGCCAATTTT
ATCTTCTGGTAAGAAATTGAGTTACATTCCTTGAGGAAAACAACTTAGGAAACTATTTCAAATGTGAACTGT
TAGGGTATCATACAATGGAAAACAATGATACAAATTTTACACTAAAATTTATTTCAATAATTTACATACTTA
GCAAAAAAATTCTTCAAACATCCAGTCAAGCATGCTTTTTGACAGTATTTTTTTATACTTCTTTTATCAGCA
TTGCATTATTTCATACTTAAATTATTACATAAATCTATTTTT↑**AAGAGTAATATA**↓TAATTATTAATGTATT
AATAATTATTAGTGATGTAATTAATGTAGTAATTTTAAATTATGTAATAACGCATAATTTAAAATTGTGCAT
TAAATTTATTAATGCATTTAAATATTAAATTTAAAATTATGCATTATTGCATAATTAAGCTCTATTATTTAA
AGAGTTTTTACTAAATGTAGATATTGTACCGTTTTCTATTTTTAATGACTAAGATGCAGTTTTCTTTTGTTA

Filled site:

ACAGGCTATATATATACTTTAACATTTTAGGGCTGGTACCACCTATAAAGTAAATGTTTTTGGAATGTTTGA
TGGAGGAGAAAGCTCACCACTTGTTGGACAAGAAATGACAACCCTTTCCGACACAACTGTTATGCCAATTTT
ATCTTCTGGTAAGAAATTGAGTTACATTCCTTGAGGAAAACAACTTAGGAAACTATTTCAAATGTGAACTGT
TAGGGTATCATACAATGGAAAACAATGATACAAATTTTACACTAAAATTTATTTCAATAATTTACATACTTA
GCAAAAAAATTCTTCAAACATCCAGTCAAGCATGCTTTTTGACAGTATTTTTTTATACTTCTTTTATCAGCA
TTGCATTATTTCATACTTAAATTATTACATAAATCTATTTTTAAGAGTAATATA**GGCCGGGCGCGGTGGCT**
CACGCCTGTAATCCCAGCACTTTGGGAGGCCGAGGCGGGCGGATCACGAGGTCAGGAGATCGAGAC
CATCCTGGCTAACACGGTGAAACCCCGTCTCTACTAAAAAAAAAAATACAAAAAATTAGCCGGGCGT
GGTAGCGGGCGCCTGTAGTCCCAGCTACTCGGGAGGCTGAGGCAGGGGAATGGCGTGAACCCGGGA
GGCGGAGCTTGCAGTGAGCCGAGATCCCGCCACTGCACTCC…[NEOcassette]*AAAAAAAAAAA*
AAAAAAAAAACATTACAAAAAAAAAAAAAAAAAAAAAAAAAAAAAAAAAAAAAAAAAAAAAAAAAAG
AAAAAAAAAAAAAAAAAAAAAAAAAAAAAAAAAAAAGAGTAATATATAATTATTAATGTATTAATAATT
ATTAGTGATGTAATTAATGTAGTAATTTTAAATTATGTAATAACGCATAATTTAAAATTGTGCATTAAATTT
ATTAATGCATTTAAATATTAAATTTAAAATTATGCATTATTGCATAATTAAGCTCTATTATTTAA

No repetitive sequences in the immediate proximity.

CLONE 9

[N4, N7, P7, R3]

Driver: L1
Plasmid: AlurescueA70D $A_{17}CATTACA_{18}GA_{17}CACACA_{18}$ (T)
Chromosome: 17
5' position: 38,298,267
Strand: minus
DR: AAAATGATGGATGC
ENDOsite: TTTT/AT

Empty site:

AGTACCCAGCTGTAACACCCTACTTAGCCCTATCCCAGTGCTATCCCCTATCAGCAAGTGGTACAATTGC
TTGAATGTTTGTCTGACTTCCTTATTGAGTTGTAAGATCTCTAAAGATAGGAATTTTGTTATAGTGTTCA
CTATTCCATCCTCAAGGGCTTAACAGATTCTAAAATGTAGTAACAGTTCTAAAAATGTATAGACAAAATC
TTTGATCCACCATCCAATTCCCCCTATATATAATCCCATTACCAGAACTCAGGAGTCCACTCAACAACCC
AGGAAACAAACACTTACTCTGATGGCATCTCTAGTAGTTTATCCAAAAGACAGGAAACTTTCTCCTAAAA
ACAGAAAAATCCCCACCTAT↑**AAAATGATGGATGC**↓TCTTGGCACAAAACATTCCGAATCCTACCCTTTCAA
AGTCTTCAGATAGATTTGGAAAAAAATAACCAATTGGCTGGCTTCTCAACCTACACTGCATAAAACAAAC
TGTCAAACATCTTTCAGAGGACTTCAAGATATCCTCACTCTCTTATTTAGAACCTCATATTCTTCAGACA
GGAAGAGAAGCCAACTAACAGTATAACACCTGTGGCTTGAGACGGCGTGCCCCAAGTTTTCCCTAGGTCT

Filled site:

TAGACAAAATCTTTGATCCACCATCCAATTCCCCCTATATATAATCCCATTACCAGAACTCAGGAGTCCACT
CAACAACCCAGGAAACAAACACTTACTCTGATGGCATCTCTAGTAGTTTATCCAAAAGACAGGAAACTTTCT
CCTAAAAACAGAAAAATCCCCACCTATAAAATGATGGATGC**GGCCGGGCGCGGTGGCTCACGCCTGTAA**
TCCCAGCACTTTGGGAGGCCGAGGCGGGCGGATCACGAGGTCAGGAGATCGAGACCATCCTGGCTA
ACACGGTGAAACCCCGTCTCTACTAAAAAAAAAAATACAAAAAATTAGCCGGGCGTGGTAGCGGGCG
CCTGTAGTCCCAGCTACTCGGGAGGCTGAGGCAGGGGAATGGCGTGAACCCGGGAGGCGGAGCTTG
CAGTGAGCCGAGATCCCGCCACTGCACTCC… [NEOcassette] ***AAAAAAAAAAAAAAAAAAAAAA***
AAAAAAAAAAAAAAACATTACAAAAAAAAAAAAAAAAAAAAAAAAAAAAAAATGATGGATGCTCTT
GGCACAAAACATTCCGAATCCTACCCTTTCAAAGTCTTCAGATAGATTTGGAAAAAAATAACCAATTGGCTG
GCTTCTCAACCTACACTGCATAAAACAAACTGTCAAACATCTTTCAGAGGACTTCAAGATATCCTCACTCTC
TTATTTAGAACCTCATATTCTTCAGACA

LINE2 at 5'.

CLONE 10

[R26]

Driver: ORF2
Plasmid: AlurescueA70D $A_{17}CATTACA_{18}GA_{17}CACACA_{18}$ (T)
Chromosome: 14
5’ position: 78,080,203
Strand: plus
DR: AAAAATGAAAACAAC
ENDOsite: TTTT/AA

Empty site:

TTCAAGCACAGAAACATACTCTTGAGAGTGTAAAGTGATCCTAGACGTTTCTGACTTCTCTCCTGTTCATTA
AAAGACATGGTGGGAGATGTGAGGAAAAGAATTTACATCTCAACAATGTTATACTCTAAATTCATATTTTAG
TAAAAGCATGACACACAGCTGTATGCGTATTTTTGATGGCACAGTTGGCAACATTTCCCCCCCAGAAAGAGG
GGAAAGATAGGTAGAGGTTGGAGCACAAGCACATCGCTTTAATT↑**AAAAATGAAAACAAC**↓TTTTAAAAAAT
GTTAAGATATTGTATTGCTCTCATGTAGTTACCAAGATCTCTCTTTTGTATAAGAAAGAAAAATATTCACAG
ACAATATTCCTCCATCTGAGAGTGTAGAAAGCTCCAGATCTGAAGTCTGTATTAAAAATACTGGTGAGAAGT
AGTTTTCTAGAAGGGATACTGCAGCCCTCACAGCATTCCAACAGACCGTTCTTCCAAATGCCACCAATGTGC

Filled site:

TTCAAGCACAGAAACATACTCTTGAGAGTGTAAAGTGATCCTAGACGTTTCTGACTTCTCTCCTGTTCATTA
AAAGACATGGTGGGAGATGTGAGGAAAAGAATTTACATCTCAACAATGTTATACTCTAAATTCATATTTTAG
TAAAAGCATGACACACAGCTGTATGCGTATTTTTGATGGCACAGTTGGCAACATTTCCCCCCCAGAAAGAGG
GGAAAGATAGGTAGAGGTTGGAGCACAAGCACATCGCTTTAATTAAAAATGAAAACAAC**GGCCGGGCGCGG**
TGGCTCACGCCTGTAATCCCAGCACTTTGGGAGGCCGAGGCGGGCGGATCACGAGGTCAGGAGATC
GAGACCATCCTGGCTAACACGGTGAAACCCCGTCTCTACTAAAAAAAAATACAAAAAATTAGCCGG
GCGTGGTAGCGGGCGCCTGTAGTCCCAGCTACTCGGGAGGCTGAGGCAGGGGAATGGCGTGAACCC
GGGAGGCGGAGCTTGCAGTGAGCCGAGATCCCGCCACTGCACTCC…[NEOcassette]*AAAAAAA*
AAAAAAAAAAAACATTACAAAAAAAAAAAAAAAAAAAGAAAAAAAAAAAAAAAAAAAAAAAAAAAA
AAAAAAAAAAAAAAAACACACAAAATGAAAACAACTTTTAAAAAAATGTTAAGATATTGTATTGCTCTCATG
TAGTTACCAAGATCTCTCTTTTGTATAAGAAAGAAAAATATTCACAGACAATATTCCTCCATCTGAGAGTGT
AGAAAGCTCCAGATCTGAAGTCTGTATT

No repetitive sequences in the immediate proximity.

CLONE 14

[O32]

Driver: ORF2
Plasmid: AlurescueA70D $A_{17}CATTACA_{18}GA_{17}CACACA_{18}$ (T)
Chromosome: 12
5' position: 54,194,943
Strand: plus
DR: GAAAAGATGT
ENDOsite: TTTC/AT

Empty site:

CACAAGCACACAGTATATTTAAGTATGGCATTAGAAAACTATGTCAGTCAGTAAGACAATGCCCTGTGAGTG
GTAACCACAATTAAGTGCTATTGGAATTCTGGGAAGGGAGGGAGAATTGTGAGGGACTGGCTAGAGAAAACT
TCATAGAAGTGGTAGGATTTTAAGAAAAATTTTGAGAATAGACAGAACATTCTACATTAATGAAATGACTCA
TAGTTATGGAAGCAAGAAAGCAAAAGGTATTCTACACACACTAAACAGAGCTTTCTCTAAACTTTTTGAAGT
CCAGGATGATATATATTCACATTTGATTTCTTAGCATACCGAATATATAGTAGCTAGATGTTCAATGTATAT
TTGTTCAATGAATGAAACAATGAATGACCCATTTATCTGGCACAGAGGGTTCATGTGAGGGCATTTTGAAAG
AGATGAATAGAACTGTAGGCTCTAGAAGAGGGGAT↑**GAAAAGATGT**↓GTAAAAACTGCTTAAATGAGATCTT
TCTTGAGTCAGTGGGCAGATAACTTAGAAAAGAGATGAGAGGCAGGGAGAACTTTTAGGATGCTTATGTAGA
AGCTTAGATGATAAATGCTAAAAGACTAAACTAGGACCATGGCATGCAAATAGAAATGCATGGATGGAAGTA
CCAACATTGTGAAAATGTGATGAACAAAACTTGGTTACTAATGGACTTTGGTGGCGTAGCGGTGGGGAGGGT

Filled site:

CACAAGCACACAGTATATTTAAGTATGGCATTAGAAAACTATGTCAGTCAGTAAGACAATGCCCTGTGAGTG
GTAACCACAATTAAGTGCTATTGGAATTCTGGGAAGGGAGGGAGAATTGTGAGGGACTGGCTAGAGAAAACT
TCATAGAAGTGGTAGGATTTTAAGAAAAATTTTGAGAATAGACAGAACATTCTACATTAATGAAATGACTCA
TAGTTATGGAAGCAAGAAAGCAAAAGGTATTCTACACACACTAAACAGAGCTTTCTCTAAACTTTTTGAAGT
CCAGGATGATATATATTCACATTTGATTTCTTAGCATACCGAATATATAGTAGCTAGATGTTCAATGTATAT
TTGTTCAATGAATGAAACAATGAATGACCCATTTATCTGGCACAGAGGGTTCATGTGAGGGCATTTTGAAAG
AGATGAATAGAACTGTAGGCTCTAGAAGAGGGGATGAAAAGATGT**GCCGGGCGCGGTGGCTCACGCCTGT**
AATCCCAGCACTTTGGGAGGCCGAGGCGGGCGGATCACGAGGTCAGGAGATCGAGACCATCCTGGC
TAACACGGTGAAACCCCGTCTCTACTAAAAAAAAAATACAAAAAATTAGCCGGGCGTGGTAGCGGG
CGCCTGTAGTCCCAGCTACTCGGGAGGCTGAGGCAGGGGAATGGCGTGAACCCGGGAGGCGGAGCT
TGCAGTGAGCC…[NEOcassette]*AAAAAAAAAAAAAAAAACATTACAAAAAAAAAAAAAAAAAA*
AAAAAAAAAAAAAGAAAAAAAAAAAAAAAAAAAAAAAAAAAAAAAAAAAAAAAAAAAAAAAAAAAAAA
AAAAAAAAAAAAAAAAAAAAAAAAAAAAAAAGAAAAGATGTGTAAAAACTGCTTAAATGAGATCTTTCT
TGAGTCAGTGGGCAGATAACTTAGAAAAGAGATGAGAGGCAGGGAGAACTTTTAGGATGCTTATGTAGAAGC
TTAGATGATAAATGCTAAAAGACTAAACTAGGACCATGGCATGC

LINE1 at 5'.

CLONE 15

[O34]

Driver: ORF2
Plasmid: AlurescueA70D $A_{17}CATTACA_{18}GA_{17}CACACA_{18}$ (T)
Chromosome: 3
5' position: 149,112,532
Strand: plus
DR: AAAAATTTTGAACCTTG
ENDOsite: TTTT/AA

Empty site:

AGACTGCCAAAGGGGTTCATGACACAAAATAAATTAAAAATTTACAGCATCGTGTATATAATAGCAAATGAT
CAAAGCAACCAAAAGTGGAAACTGACCAAAAACCATTTGTACCTTGCTATAGTGAATACTCTGCAGCTGAAA
AAAAAAAGGAATGAAGAATCTGCCTAATAAAATGAAACAATATCCAAGATATTTTACAGAGAGTAAAGCAGG
ATACAAAATAATTTATGTAGCAAGCTGCTACTTGTTTAAAAAAAAATGAAAGAGAGAGTGAATATATACAAAT
GTCTGTTTGCTTGTATGTGCATAAAATAGCTCTGGAGGACACACAGTAAACTGATAATATTGGTGGCTTCTG
GGGATGAGAACTGGGTGGTTTGGGGCTGGAGTGTTATGGAGACTTTTTCCTGATAAAACCTACAGGACCTTT
↑**AAAAATTTTGAACCTTG**↓CAAAAGTATAATCTATTCAAAAATTAAATTTAAAAAAGGGAACACTTAAGAAC
TCCTTACTGGAGTCTGTACAGGAGATTCATACATAGCCCCAAGGCAACACCTGGTCGAGTCCTGTCTCTCCT
CCTCTGTGGAGCCTCAGCCTGCTCTGCAGCAAGCCTTGTAGCTTCTATGCCCTCAATCTCCTATTCTGAACC

Filled site:

ATACAAAATAATTTATGTAGCAAGCTGCTACTTGTTTAAAAAAAAATGAAAGAGAGAGTGAATATATACAAAT
GTCTGTTTGCTTGTATGTGCATAAAATAGCTCTGGAGGACACACAGTAAACTGATAATATTGGTGGCTTCTG
GGGATGAGAACTGGGTGGTTTGGGGCTGGAGTGTTATGGAGACTTTTTCCTGATAAAACCTACAGGACCTTT
<u>AAAAATTTTGAACCTTG</u>**GGCCGGGCGCGGTGGCTCACGCCTGTAATCCCAGCACTTTGGGAGGCCGA
GGCGGGCGGATCACGAGGTCAGGAGATCGAGACCATCCTGGCTAACACGGTGAAACCCCGTCTCTA
CTAAAAAAAAATACAAAAAATTAGCCGGGCGTGGTAGCGGGCGCCTGTAGTCCCAGCTACTCGGGA
GGCTGAGGCAGGGGAATGGCGTGAACCCGGGAGGCGGAGCTTGCAGTGAGCCGAGATCCCGCCACT
GCACTCC…[NEOcassette]*AAAAAAAAAAAAAAAAAAAAAAAAAAAAAAACATTACAAAAAAAAAAA
AAAAAAAAGAAAAAAAAAAAAAAAAAAAAAAAAAAAAAAAAAAAAAAAAAAAAAAAAAAAAAAAA
AAAAAAAAAAA<u>AAAAA</u>*** <u>TTTTGAACCTTG</u>CAAAAGTATAATCTATTCAAAAATTAAATTTAAAAAAGGGAACA
CTTAAGAACTCCTTACTGGAGTCTGTACAGGAGATTCATACATAGCCCCAAGGCAACACCTGGTCGAGTCCT
GTCTCTCCTCCTCTGTGGAGCCTCAGCCTGCTCT

LINE1 at 5'.

CLONE 17

[U23]

Driver: L1
Plasmid: Alurescue A30D $A_{10}CTA_{10}TACA_{10}$
Chromosome: 10
5' position: 91,344,768
Strand: plus
DR: AAAAAATATAAATTTTT
ENDOsite: TTTT/AG

Empty site:

AATTTATTGGACACCTACTTATATATATTAAATACATTCACATATGTAAATGCTTAGAACTATGTCTGGTAC
ATGTGTGCTAAGTGTGTGCCAGGGTTGTTAGTATTATAACTATTTCATCTTTATCTTTGTCATGGCAAAACA
CTGACTATTATTTCACATATGAGAAATGGATAAAGTAAATTACCCTTGCAGGGTCACAGGTCTTGTACCAGG
GCTCAATACCAAATCTGTCTGACTTTAAAGCTATATTTCATGAGAGAAAACCCTAAACTTCCCTCAGTAGCT
CTTGCAAAGGCACTTAAATTAGTCCAAGCCTTACTTTGCCCATCTGTAAAATAAAAGACCATACGTCTATGA
AAATGAAAGGAACT↑**AAAAAATATAAATTTTT**↓AGAGAACTATGTGTTAGTTTGTTCAGGCTGCTATATATA
ACAAAGTACCCTAGACTGGGTGGCTTATAAACAATAGAAATTTGTTTCTCACAGTTCTAGA

Filled site:

AATTTATTGGACACCTACTTATATATATTAAATACATTCACATATGTAAATGCTTAGAACTATGTCTGGTAC
ATGTGTGCTAAGTGTGTGCCAGGGTTGTTAGTATTATAACTATTTCATCTTTATCTTTGTCATGGCAAAACA
CTGACTATTATTTCACATATGAGAAATGGAAGGTAATAAAGTAAATTACCCTTGCAGGGTCACAGGTCTTGT
ACCAGGGCTCAATACCAAATCTGTCTGACTTTAAAGCTATATTTCATGAGAGAAAACCCTAAACTTCCCTCA
GTAGCTCTTGCAAAGGCACTTAAATTAGTCCAAGCCTTACTTTGCCCATCTGTAAAATAAAAGACCATACGT
CTATGAAAATGAAAGGAACTAAAAAATATAAATTTTT**GGCCGGGCGCGGTGGCTCACGCCTGTAATCCCAGC**
ACTTTGGGAGGCCGAGGCGGGCGGATCACGAGGTCAGGAGATCGAGACCATCCTGGCTAACACGGTGAAACC
CCGTCTCTACTAAAAAAAAATACAAAAAATTAGCCGGGCGTGGTAGCGGGCGCCTGTAGTCCCAGCTACTCG
GGAGGCTGAGGCAGGGGAATGGCGTGAACCCGGGAGGCGGAGCTTGCAGTGAGCCGAGATCCCGCC... **[NEOc**
assette] ***AAAAAAAAAAAAAAACTAAAAAAAAAAAAATACAAAAAAAAAAATTTAAAAAAAAAAAAAAAAA***
AAAAAAAAAAAAAAAAAAATATAAATTTTTAGAGAACTATGTGTTAGTTTGTTCAGGCTGCTATATATAACAA
AGTACCCTAGACTGGGTGGCTTATAAACAATAGAAATTTGTTTCTCACAGTTCTAGA

MIRs at the 5'; LTR at the 3'.

CLONE 18

[U22]

Driver: L1
Plasmid: Alurescue A30D $A_{10}CTA_{10}TACA_{10}$
Chromosome: 7
5' position: 80,207,048
Strand: minus
DR: AAGAATATTCTTT
ENDOsite: TCTT/AA

Empty site:

TGCAACAAATATTAGTGAGCAGAACATTAATGTCTTATAAAGGAACTTCTTCATGGACACTATTAATCATAT
TAATTGTCCTGCCTTTGAAATTTGGGTTCTTCTACACTAAATAAAATTTGGATAACTTAATTCAGGAGAAAC
CACTAAAAATGGCTAGAAGAATAGAAGTATTAGAAAAGTAAACACACAGTTGGAAAAATTAATATGTATGTA
GTACATAAGTGGATATGGTTGGGTGGTGACCATCACTGAAGGTATGGTTTGTTAATTTAAAATACAAATTGA
TTTCACTTGTATAAACATTACTGAATAATATAAGGCATTAAGCATGGTTTTAGAATTATCTT↑**AAGAATATT**
CTTT↓AATACAAAGTAAATCATCTGCCTTAACAAATCATCATGATTCAACAGGCAATTAGAAAGATTGGAAG
ACCATTCTAGGCAGAGGACACTACAAGGGTTAA

Filled site:

TGCAACAAATATTAGTGAGCAGAACATTAATGTCTTATAAAGGAACTTCTTCATGGACACTATTAATCATAT
TAATTGTCCTGCCTTTGAAATTTGGGTTCTTCTACACTAAATAAAATTTGGATAACTTAATTCAGGAGAAAC
CACTAAAAATGGCTAGAAGAATAGAAGTATTAGAAAAGTAAACACACAGTTGGAAAAATTAATATGTATGTA
GTACATAAGTGGATATGGTTGGGTGGTGACCATCACTGAAGGTATGGTTTGTTAATTTAAAATACAAATTGA
TTTCACTTGTATAAACATTACTGAATAATATAAGGCATTAAGCATGGTTTTAGAATTATCTTAAGAATATTC
TTT**GCCGGGCGCGGTGGCTCACGCCTGTAATCCCAGCACTTTGGGAGGCCGAGGCGGGCGGATCACGAGGTC**
AGGAGATCGAGACCATCCTGGCTAACACGGTGAAACCCCGTCTCTACTAAAAAAAAATACAAAAAATTAGCC
GGGCGTGGTAGCGGGCGCCTGTAGTCCCAGCTACTCGGGAGGCTGAGGCAGGGGAATGGCGTGAACCCGGGA
GGCGGAGCTTGCAGTGAGCC... [NEOcassette] ***AAAAAAAAAAAAAAAACTAAAAAAAAAAAAAATACAAA***
AAAAAAAAAAAAAAGAATATTCTTTAATACAAAGTAAATCATCTGCCTTAACAAATCATCATGATTCAACAG
GCAATTAGAAAGATTGGAAGACCATTCTAGGCAGAGGACACTACAAGGGTTAA

No repetitive sequences in the immediate proximity.

CLONE 20

[Q34]

Driver: L1
Plasmid: AlurescueA70D $A_{17}CATTACA_{18}GA_{17}CACACA_{18}$ (T)
Chromosome: 13
5' position: 110,579,080
Strand: plus
DR: AAAGACTGAAGAAATA
ENDOsite: CTTT/GA

Empty site:

TATTTCTAGCAAAAGTCTACCTCATATTAATCAAAAATAAATTCTCCTCCAGATTAAGATGACTGGCCTACA
GAAATAGGAAAGCACACCTCGGTGAGGCAGTTAGAGAGTGGATGATTGACTGATAAACACCAGAAACTTGGC
TAAGCATTGTGGATGTATTAATCCAAAAATTTTAGGGACCTTAAAATTAAAACAAACAGAATGGTGTACAAA
TATTGTACTCTAGTTAGTGAGTTTTTTTTTCCACGGGAATGTGAGTTAGCAATTCTGAAACTAGTTTTTGTT
C↑**AAAGACTGAAGAAATA**↓AAGAAATATGTATGTAAGTAATGACAGCCAGGACTCTGTCAGAGAAATGAGTT
GCCAACGAAAAAAGGGGGAATGCTCAAATGAACCCCATCAAGTATCA

Filled site:

TATTTCTAGCAAAAGTCTACCTCATATTAATCAAAAATAAATTCTCCTCCAGATTAAGATGACTGGCCTACA
GAAATAGGAAAGCACACCTCGGTGAGGCAGTTAGAGAGTGGATGATTGACTGATAAACACCAGAAACTTGGC
TAAGCATTGTGGATGTATTAATCCAAAAATTTTAGGGACCTTAAAATTAAAACAAACAGAATGGTGTACAAA
TATTGTACTCTAGTTAGTGAGTTTTTTTTTCCACGGGAATGTGAGTTAGCAATTCTGAAACTAGTTTTTGTT
CAAAGACTGAAGAAATA**GGCCGGGCGCGGTGGCTCACGCCTGTAATCCCAGCACTTTGGGAGGCCGAGGCGG**
GCGGATCACGAGGTCAGGAGATCGAGACCATCCTGGCTAACACGGTGAAACCCCGTCTCTACTAAAAAAAAA
AATACAAAAAATTAGCCGGGCGTGGTAGCGGGCGCCTGTAGTCCCAGCTACTCGGGAGGCTGAGGCAGGGGA
ATGGCGTGAACCCGGGAGGCGGAGCTTGCAGTGAGCC… [NEOcassette] ***AAAAAAAAAAAAAAAAA***
CATTACAAAAAAAAAAAAAAAAAAAAAAAAAAGGAAAAAAAAAAAAAAAAAAAAAAAAAG
ACTGAAGAAATAAAGAAATATGTATGTAAGTAATGACAGCCAGGACTCTGTCAGAGAAATGAGTTGCCAACG
AAAAAAGGGGGAATGCTCAAATGAACCCCATCAAGTATCA

LINE1 flanks insertion site.

CLONE 22

[Q39a]

Driver: L1
Plasmid: AlurescueA70D A$_{17}$CATTACA$_{18}$GA$_{17}$CACACA$_{18}$ (T)
Chromosome: 12
5’ position: 20,053,995
Strand: plus
DR: AGAAATAAGTTTTTAT
ENDOsite: TTCT/AC

Empty site:

TATTCTTGACTTTAGAACTTTTGTGTATATTTTAGAATCAGCTTGTCAAACTCTATAAAATGACTATTGAAA
TTTTGTTTGCAATTGAATTGAATCCATTCAGCTGGGAAGAATTGACATCGCTATAATAGGGAGCTATCAACC
CCTGAACATGTTTTTAATTTCCATTTATTAGGTCCTTTTTACTGTTTCTCAATTAGTTGCATAAATTTCTCT
ATAGAAGTCTTATATATATGCTCATTGATTTATTTTTAGGTACTTTTTATTTTTGATACTATTGTAAATAGT
ACCACTTTTAATTTTATTTTATAATTGTTTGT↑**AGAAATAAGTTTTTAT**↓ATTGATTTTTGTATCCAGGAAC
AATGCTAAGTTTTCCTCATTAAAAAACTATTTGTAGGTTTTTTTGGATTTTATATAAATGCAGTTATTTCCT
CTGTAA

Filled site:

TATTCTTGACTTTAGAACTTTTGTGTATATTTTAGAATCAGCTTGTCAAACTCTATAAAATGACTATTGAAA
TTTTGTTTGCAATTGAATTGAATCCATTCAGCTGGGAAGAATTGACATCGCTATAATAGGGAGCTATCAACC
CCTGAACATGTTTTTAATTTCCATTTATTAGGTCCTTTTTACTGTTTCTCAATTAGTTGCATAAATTTCTCT
ATAGAAGTCTTATATATATGCTCATTGATTTATTTTTAGGTACTTTTTATTTTTGATACTATTGTAAATAGT
ACCACTTTTAATTTTATTTTATAATTGTTTGTAGAAATAAGTTTTTAT**GGCCGGGCGCGGTGGCTCACGCCT**
GTAATCCCAGCACTTTGGGAGGCCGAGGCGGGCGGATCACGAGGTCAGGAGATCGAGACCATCCTGGCTAAC
ACGGTGAAACCCCGTCTCTACTAAAAAAAAAATACAAAAAATTAGCCGGGCGTGGTAGCGGGCGCCTGTAGT
CCCAGCTACTCGGGAGGCTGAGGCAGGGGAATGGCGTGAACCCGGGAGGCGGAGCTTGCAGTGAGCCGAGAT
CCCGCCACTGCACTCCAGC…[NEOcassette]***AAAAAAAAAAAAAAAAAAAAAAAAAAAAAAAAAAAAA***
AAAAAAAAAAAAAAAAAAAAAAAAAAAAAAAAAACATTACAAAAAAAAAAAAAAAAAAAAAGAAATAAGTT
TTTATATTGATTTTTGTATCCAGGAACAATGCTAAGTTTTCCTCATTAAAAAACTATTTGTAGGTTTTTTTG
GATTTTATATAAATGCAGTTATTTCCTCTGTAA

LINE1 flanks insertion site.

CLONE 23

[JK25, L2, N14, S14]

Driver: ORF2
Plasmid: AlurescueA70D $A_{17}CATTACA_{18}GA_{17}CACACA_{18}$ (T)
Chromosome: 5
5’ position: 95,622,168
Strand: plus
DR: AAAAGAATCAATAG
ENDOsite: TTTT/AA

Empty site:

ATGAAATTCTCTATGTTAACAGTAGTAACAGTTCATTATCTCTCATTCTAAGATGCTTTAAATACTCTAGTT
GCCATAAAGGTAGCTGACCTATAGAAATATTTCCCAAGCCTATTAGCGGAGACAAGATATTACTTTTCTTGT
CAATGAAAGAAGAGGTCGATGGATGGAAGCTGCAAACAATTATTTTAGAGAAATCTTCCTTCACTTGCTCCC
TGGACTCAAAGAACAAATGTT↑**AAAAGAATCAATAG**↓AATGTTTTATAAGAGAATGAGCCGTTAAAGGGAGG
GGCAAGATATGGACCGTGTTGTGACTTGGAAACACCATGCTTTGCACCAGTAACCAATATTGTTA

Filled site:

ATGAAATTCTCTATGTTAACAGTAGTAACAGTTCATTATCTCTCATTCTAAGATGCTTTAAATACTCTAGTT
GCCATAAAGGTAGCTGACCTATAGAAATATTTCCCAAGCCTATTAGCGGAGACAAGATATTACTTTTCTTGT
CAATGAAAGAAGAGGTCGATGGATGGAAGCTGCAAACAATTATTTTAGAGAAATCTTCCTTCACTTGCTCCC
TGGACTCAAAGAACAAATGTTAAAAGAATCAATAG**TGGCCGGGCGCGGTGGCTCACGCCTGTAATCCCAGCA**
CTTTGGGAGGCCGAGGCGGGCGGATCACGAGGTCAGGAGATCGAGACCATCCTGGCTAACACGGTGAAACCC
CGTCTCTACTAAAAAAAAATACAAAAAATTAGCCGGGCGTGGTAGCGGGCGCCTGTAGTCCCAGCTACTCGG
GAGGCTGAGGCAGGGGAATGGCGTGAACCCGGGAGGCGGAGCTTGCAGTGAGCCGAGATCCCGCCACTGCAC
TCCAGC… [NEOcassette] ***AAAAAAAAAAAAAAAAAAAAAAAAAAAAAAAAAAAAAAAAAAAAAAAAAAA***
AAAAAAAAAAAAAAAAAAAAAAAAAAAAAAAAAAAAAAAAAAAAAAAAAAAAAAAAAAAAAAAAAAAAAAAA
AAAAAAAGAATCAATAGAATGTTTTATAAGAGAATGAGCCGTTAAAGGGAGGGGCAAGATATGGACCGTGTT
GTGACTTGGAAACACCATGCTTTGCACCAGTAACCAATATTGTTA

An extra T added immediately 5’ of Alu.

No repetitive sequences in the immediate proximity.

CLONE 25

[Q1]

Driver: L1
Plasmid: AlurescueA70D $A_{17}CATTACA_{18}GA_{17}CACACA_{18}$ (T)
Chromosome: 12
5’ position: 1,485,432
Strand: minus
DR: AAGAAAGACATATT
ENDOsite: TCTT/AA

Empty site:

GTGGGGAAGACCCGGTGATGTAAAGCCCCTGTGCCCTGCGCACGACAGTCAAGTGGCACGTGGTGGCCATTA
TTGTCACTGCTCTATTTTCACAGAATTCTGAAAAGTTATACTAGTCGGGGATACCCAGAACGGGAGGTAACA
TGTGGAAAATCACAGAACTCGAAGGCGAGTGAGATGGGATGTAGCTTGGGAGTTGACATTTTCCTAATGTTG
AGGACTTCAGCCAGACGGGCATTTAAATGTTCTTCAGTGACGAAAATGTAAGATTT↑**AAGAAAGACATATT**↓
AGAATTTAGAATTATTTTTTAAAAATTCTATATGAGAGTCATATCATTAAAAAGAACAGGTGGAAAAAAGAA
AGGATAAAAAGTACCTCTACCTCTTTTT

Filled site:

GTGGGGAAGACCCGGTGATGTAAAGCCCCTGTGCCCTGCGCACGACAGTCAAGTGGCACGTGGTGGCCATTA
TTGTCACTGCTCTATTTTCACAGAATTCTGAAAAGTTATACTAGTCGGGGATACCCAGAACGGGAGGTAACA
TGTGGAAAATCACAGAACTCGAAGGCGAGTGAGATGGGATGTAGCTTGGGAGTTGACATTTTCCTAATGTTG
AGGACTTCAGCCAGACGGGCATTTAAATGTTCTTCAGTGACGAAAATGTAAGATTTAAGAAAGACATATT**GG**
CCGGGCGCGGTGGCTCACGCCTGTAATCCCAGCACTTTGGGAGGCCGAGGCGGGCGGATCACGAGGTCAGGA
GATCGAGACCATCCTGGCTAACACGGTGAAACCCCGTCTCTACTAAAAAAAAAATACAAAAAATTAGCCGGGC
GTGGTAGCGGGCGCCTGTAGTCCCAGCTACTCGGGAGGCTGAGGCAGGGGAATGGCGTGAACCCGGGAGGCG
GAGCTTGCAGTGAGCCGAGATCCC... [NEOcassette] ***AAAAAAAAAAAAAAAAAAAAACATTACAAAAAA***
AAAAAAAAAAAAAAAAAAAAAGAAAAAAAAAAGAAAGACATATTAGAATTTAGAATTATTTTTTAAAAATTC
TATATGAGAGTCATATCATTAAAAAGAACAGGTGGAAAAAAGAAAGGATAAAAAGTACCTCTACCTCTTTTT

No repetitive sequences in the immediate proximity.

CLONE 26

[Q44]

Driver: L1
Plasmid: AlurescueA70D $A_{17}CATTACA_{18}GA_{17}CACACA_{18}$ (T)
Chromosome: 10
5' position: 69,896,024
Strand: minus
DR: AAAGAAAAGGACC
ENDOsite: CTTT/AA

Empty site:

CCTCCATCCATGTTAGGGAATGCTTTCATAGAAATGCCCTGACCGGTGGCATGTTTTTAGAACGCCAAATCC
TCATGACACTTACAGAATGGCAGTAGGTGCCAGGCATGATTCATGGCTCAATAGAAATGAGTCAATGATTGG
GATAAATAGGGCAGGTCCAGATGGTT↑**AAAGAAAAGGACC**↓CACCTTTCACGCACAAGGATGGCTTGCCAGA
TCTGCTCATTTGGCTAGCTAGCAAGCCTGGGAAGAAAGAAGGAAGACACTGAAGAGACACATTTTTCAG

Filled site:

CCTCCATCCATGTTAGGGAATGCTTTCATAGAAATGCCCTGACCGGTGGCATGTTTTTAGAACGCCAAATCC
TCATGACACTTACAGAATGGCAGTAGGTGCCAGGCATGATTCATGGCTCAATAGAAATGAGTCAATGATTGG
GATAAATAGGGCAGGTCCAGATGGTTAAAGAAAAGGACC**GGCCGGGCGCGGTGGCTCACGCCTGTAATCCCA**
GCACTTTGGGAGGCCGAGGCGGGCGGATCACGAGGTCAGGAGATCGAGACCATCCTGGCTAACACGGTGAAA
CCCCGTCTCTACTAAAAAAAAAATACAAAAAATTAGCCGGGCGTGGTAGCGGGCGCCTGTAGTCCCAGCTACT
CGGGAGGCTGAGGCAGGGGAATGGCGTGAACCCGGGAGGCGGAGCTTGCAGTGAGCCGAGATCCCGCCACTG
CACTCCAGCCT…[NEOcassette]*AAAAAAAAAAAAAAAAAAAAAAACATTACAAAAAAAAAAAAAAAA*
AAAAAAAAAAAAAAAAAAAAAAAAAAAAAAAAAAAAAAAAAAGAAAAGGACCCACCTTTCACGCACAAGGATG
GCTTGCCAGATCTGCTCATTTGGCTAGCTAGCAAGCCTGGGAAGAAAGAAGGAAGACACTGAAGAGACACAT
TTTTCAG

No repetitive sequences in the immediate proximity.

CLONE 27

[P29]

Driver: ORF2
Plasmid: AlurescueA70D $A_{17}CATTACA_{18}GA_{17}CACACA_{18}$ (T)
Chromosome: 9
5' position: 17,024,668
Strand: minus
DR: AAAAGTTAACCT
ENDOsite: TTTT/AT

Empty site:

CTAAGTTAATTTTCATAAATTTCATAAAGTAAGCCCACTTGGCACCCAGATCAAGAAATGGAGCATGACAGC
CCCCAGAACCTCCATTTGTGCCCCTGCTAGTTTTTATCCCCTCCCAGAAAAAAAGTCCTATCCTGACTCCTA
ACACCACAGGTTAATTTCATTTTTTTAATAAATTGAATTATACAGTATTTTTTCTTTTGGGTCTGGCTTCTT
TTACTCAACATTATGATTAT↑**AAAAGTTAACCT**↓GCCATGTGTAAAGAATTAGCCATGTTATTGTTAGTGTT
GTATAGTATTGTAATATATGACTAGACTACAGTTCATTCTACTGTTGAGGTAAATTTGAGTA

Filled site:

CTAAGTTAATTTTCATAAATTTCATAAAGTAAGCCCACTTGGCACCCAGATCAAGAAATGGAGCATGACAGC
CCCCAGAACCTCCATTTGTGCCCCTGCTAGTTTTTATCCCCTCCCAGAAAAAAAGTCCTATCCTGACTCCTA
ACACCACAGGTTAATTTCATTTTTTTAATAAATTGAATTATACAGTATTTTTTCTTTTGGGTCTGGCTTCTT
TTACTCAACATTATGATTATAAAAGTTAACCT**GGCCGGGCGCGGTGGCTCACGCCTGTAATCCCAGCACTTT**
GGGAGGCCGAGGCGGGCGGATCACGAGGTCAGGAGATCGAGACCATCCTGGCTAACACGGTGAAACCCCGTC
TCTACTAAAAAAAAATACAAAAAATTAGCCGGGCGTGGTAGCGGGCGCCTGTAGTCCCAGCTACTCGGGAGG
CTGAGGCAGGGGAATGGCGTGAACCCGGGAGGCGGAGCTTGCAGTGAGCCGAGATCCCGCCA… [NEOcass
ette]***AAAAAAAAAAAAAAAAAAAAAAAAAAAAAAAAACATTACAAAAAAAAAAAAAAAAAAAAAAAAAAAA***
AAAGAAAAAAAAAAAAAAAAAAAAAAAAACACACAAAAAAAAAAAAAAAAAAAAAAAAAAAAAAAAAAAAAA
AAAAAAAAAAAAAAAAAAAAAAAAAAAAAAAGTTAACCTGCCATGTGTAAAGAATTAGCCATGTTATTGTTA
GTGTTGTATAGTATTGTAATATATGACTAGACTACAGTTCATTCTACTGTTGAGGTAAATTTGAGTA

LINE1 flanks insertion site.

CLONE 28

[R4]

Driver: ORF2
Plasmid: AlurescueA70D   A$_{17}$CATTACA$_{18}$GA$_{17}$CACACA$_{18}$ (T)
Chromosome: 16
5' position: 69,500,842
Strand: plus
DR: AAGAATTTATT
ENDOsite: TCTT/AC

Empty site:

ATGCCAGTCTTCAAATGATTGTTTTTCTTGGAAATAGCTAAACTGTTGGGGAAAATTAAACACTGAGATTGA
AGTTTAGTGACTGTGTTAATAAAACAAGGGAAATAAACCGTTTTTATAATTACCTTTGAAACATACTGATAG
CCATCTAACATTACTAACATATCAGATTTCTGTTGAGAGATTGTTCATTAGGGCTCGGCTATTTCACTGTCA
CAGGAACTGAGGATTACTAACAGGAAAGGATAACCCCATTCTTGTTACCAAAGTTTGAATTTAATGTTTAAT
CTTTGATACTAGT↑**AAGAATTTATT**↓ATCACAGCACTGTCCCTGATGCTGAATAGTGGGTAGGTGCTTGACA
AATCCTGGTTGATTCGTGAAAGCCTGAGGTAGAAGACTGTCCAATTATGTAAAT

Filled site:

ATGCCAGTCTTCAAATGATTGTTTTTCTTGGAAATAGCTAAACTGTTGGGGAAAATTAAACACTGAGATTGA
AGTTTAGTGACTGTGTTAATAAAACAAGGGAAATAAACCGTTTTTATAATTACCTTTGAAACATACTGATAG
CCATCTAACATTACTAACATATCAGATTTCTGTTGAGAGATTGTTCATTAGGGCTCGGCTATTTCACTGTCA
CAGGAACTGAGGATTACTAACAGGAAAGGATAACCCCATTCTTGTTACCAAAGTTTGAATTTAATGTTTAAT
CTTTGATACTAGTAAGAATTTATT**GGCCGGGCGCGGTGGCTCACGCCTGTAATCCCAGCACTTTGGGAGGCC**
GAGGCGGGCGGATCACGAGGTCAGGAGATCGAGACCATCCTGGCTAACACGGTGAAACCCCGTCTCTACTAA
AAAAAAAAAATACAAAAAATTAGCCGGGCGTGGTAGCGGGCGCCTGTAGTCCCAGCTACTCGGGAGGCTGAG
GCAGGGGAATGGCGTGAACCCGGGAGGCGGAGCTTGCAGTGAGCCGAGATCCCGCCACT…[NEOcassett
e]*AAAAAAAAAAAAAAAAAAAAAAAACATTACAAAAAAAAAAAAAAAAAAAAAAAAAAAAAAAAAAAAAA*
AAAAAAAAAAAAAAAAAAAAAAAAAAAAAAAAAAAAAAAGAATTTATTATCACAGCACTGTCCCTGATGCT
GAATAGTGGGTAGGTGCTTGACAAATCCTGGTTGATTCGTGAAAGCCTGAGGTAGAAGACTGTCCAATTATG
TAAAT

No repetitive sequences in the immediate proximity.

CLONE 29

[Q45]

Driver: L1
Plasmid: AlurescueA70D $A_{17}CATTACA_{18}GA_{17}CACACA_{18}$ (T)
Chromosome: 5
5' position: 59,814,549
Strand: plus
DR: AAGAAAGATACTGCC
ENDOsite: TCTT/GA

Empty site:

AAGCAACCAGGAAAGAAGAGGATACTGAATTGTCAGCTTGACAAAATGGGACTCTATACAGTTTCCATTTGC
CTTTAGTTAGAACTACAAGAGTTCATACACACACTCAAGGTAAACAGATGCGTGAACTCCCACAGCCTTTAC
CTGACCACTTGTGTATGGTATTTGTGGAAATGCCTGGTGTTATTTTTTTTTCTTC↑**AAGAAAGATACTGCC**↓
TTTGAGGCTTCCAAACTAGTCTTCATAATGTTGAAAGTTGCTAGGGGCTCATCAGGACACTAATGCACAACC
CAAGAAACTATCATCAGATGACTAAATT

Filled site:

AAGCAACCAGGAAAGAAGAGGATACTGAATTGTCAGCTTGACAAAATGGGACTCTATACAGTTTCCATTTGC
CTTTAGTTAGAACTACAAGAGTTCATACACACACTCAAGGTAAACAGATGCGTGAACTCCCACAGCCTTTAC
CTGACCACTTGTGTATGGTATTTGTGGAAATGCCTGGTGTTATTTTTTTTTCTTC<u>AAGAAAGATACTGCC</u>**GG**
CCGGGCGCGGTGGCTCACGCCTGTAATCCCAGCACTTTGGGAGGCCGAGGCGGGCGGATCACGAGGTCAGGA
GATCGAGACCATCCTGGCTAACACGGTGAAACCCCGTCTCTACTAAAAAAAAATACAAAAAATTAGCCGGGC
GTGGTAGCGGGCGCCTGTAGTCCCAGCTACTCGGGAGGCTGAGGCAGGGGAATGGCGTGAACCCGGGAGGCG
GAGCTTGCAGTGAGCCGAGATCCCGCCACTGCACTCCAGCCT... [NEOcassette] ***AAAAAAAAAAAAA***
AAAAAAAAAACATTACAAAAAAAAAAAAAAAAAAAAAAAAAAAAAAAAAAAAAAAAAAAAAAAAAAAAAAAA
AAAAAAAAAAAAAAAAAAAAAA<u>***AA***GAAAGATACTGCC</u>TTTGAGGCTTCCAAACTAGTCTTCATAATGTTGAA
AGTTGCTAGGGGCTCATCAGGACACTAATGCACAACCCAAGAAACTATCATCAGATGACTAAATT

No repetitive sequences in the immediate proximity.

CLONE 30

[U13, U18, U25]

Driver: L1
Plasmid: Alurescue A30D $A_{10}CTA_{10}TACA_{10}$
Chromosome: 9
5' position: 98430829
Strand: plus
DR: AAAAAATGCAATCCT
ENDOsite: TTTT/AA

Empty site:

ACTCGGACATGAAACACACTTAGCAAATTCTATTTAATTGAGTAATGAACAGATATTGAGCATCCTCCGGCA
GTGAAATCTGAAATAATGGCATTGAGAAAACAAATGTATGAAAATGGACATTCCCCACTGAACACTGCAATG
ATATCATTAATGAAATTGATATATTGGGTGTTGCCAAGTCACTCAAGTGGCTGTTAATAAATACTAGAGCTT
TTACGAGAATACAACTTCTTT↑**AAAAAATGCAATCCT**↓AAAATCAAGTCCCCAAATTTTAACATAAATGTAG
TCGGAAGGTTTCTTTATGAATTAAGAAGTAAAGTGTTAAATAGTATAAAATATAACAAAAGGCAGA

Filled site:

ACTCGGACATGAAACACACTTAGCAAATTCTATTTAATTGAGTAATGAACAGATATTGAGCATCCTCCGGCA
GTGAAATCTGAAATAATGGCATTGAGAAAACAAATGTATGAAAATGGACATTCCCCACTGAACACTGCAATG
ATATCATTAATGAAATTGATATATTGGGTGTTGCCAAGTCACTCAAGTGGCTGTTAATAAATACTAGAGCTT
TTACGAGAATACAACTTCTTT<u>AAAAAATGCAATCCT</u>**GGCCGGGCGCGGTGGCTCACGCCTGTAATCCCAGCA**
CTTTGGGAGGCCGAGGCGGGCGGATCACGAGGTCAGGAGATCGAGACCATCCTGGCTAACACGGTGAAACCC
CGTCTCTACTAAAAAAAAATACAAAAAATTAGCCGGGCGTGGTAGCGGGCGCCTGTAGTCCCAGCTACTCGG
GAGGCTGAGGCAGGGGAATGGCGTGAACCCGGGAGGCGGAGCTTGCAGTGAGCCGAGATCCCGCCACTGCAC
TCCAGCCT…[NEOcassette]***AAAAAAAAAACTAAAAAAAAAAAAAAAAAAAAAAAAAAAAAAAAATACA***
AAAAAAACAAAAAAAAAAAAAAAACAAAAAAAAAAAAAAAAAAAAAAAAAAAAAAAAAAAAAAAAAAAAAAAA
AAAAAAAAAAAAAAAAAAAAAAAAAAAAAAAAAAAA<u>AAAAAA</u><u>TGCAATCCT</u>AAAATCAAGTCCCCAAATTTT
AACATAAATGTAGTCGGAAGGTTTCTTTATGAATTAAGAAGTAAAGTGTTAAATAGTATAAAATATAACAAA
AGGCAGA

No repetitive sequences in the immediate proximity.

CLONE 31

[U14]

Driver: L1
Plasmid: Alurescue A30D $A_{10}CTA_{10}TACA_{10}$
Chromosome: 15
5' position: 36,732,143
Strand: plus
DR: AAAATTTTATTTT
ENDOsite: TTTT/AT

Empty site:

TATGGTATTATTTCAAATAACCATTTTGTTGACTGAATATTTTCACTTATTAAACCAATAATTATTAAAAGA
TCTGAGATTGCCCAGGAGAATGAAGATCCTTCATCACATAATGTAAGCTAAGCAGTCAGTTATTAGAGTCAT
TTACAATATTAATAGCCCTGAGCAGAAGTACAAACCATAGGTCTGTATACTCTGCTGCCGAATTTGCCAGTT
ACAAACTGGGGTTCTATTGCTTGACTCCATCTTTGCCATATGTTCTGTTATTGGAAATTCCAT↑**AAAATTTT**
ATTTT↓GCTATCTTTTATGAAGCATAGACCAAAGAGCTTAAAGATATTTCATCAACCTAGAAAAAAATGCAA
TACTGTGAGAATACGTAGACACATGTATGTGTGT

Filled site:

TATGGTATTATTTCAAATAACCATTTTGTTGACTGAATATTTTCACTTATTAAACCAATAATTATTAAAAGA
TCTGAGATTGCCCAGGAGAATGAAGATCCTTCATCACATAATGTAAGCTAAGCAGTCAGTTATTAGAGTCAT
TTACAATATTAATAGCCCTGAGCAGAAGTACAAACCATAGGTCTGTATACTCTGCTGCCGAATTTGCCAGTT
ACAAACTGGGGTTCTATTGCTTGACTCCATCTTTGCCATATGTTCTGTTATTGGAAATTCCATAAAATTTTA
TTTT**GGCCGGGCGCGGTGGCTCACGCCTGTAATCCCAGCACTTTGGGAGGCCGAGGCGGGCGGATCACGAGG**
TCAGGAGATCGAGACCATCCTGGCTAACACGGTGAAACCCCGTCTCTACTAAAAAAAAAATACAAAAAATTA
GCCGGGCGTGGTAGCGGGCGCCTGTAGTCCCAGCTACTCGGGAGGCTGAGGCAGGGGAATGGCGTGAACCCG
GGAGGCGGAGCTTGCAGTGAGCCGAGATCCCGCCACT…[NEOcassette]***AAAAAAAAAAAACTAAAA***
AAAAAATACAAAAAAAAAAAAAACAAAAAAAAAAAAAAAAAAAAAAAAAAAAAAAAAAAAAAAAAAAAAAAA
AAAAAAAAAAAAAAATTTTATTTTGCTATCTTTTATGAAGCATAGACCAAAGAGCTTAAAGATATTTCATCA
ACCTAGAAAAAAATGCAATACTGTGAGAATACGTAGACACATGTATGTGTGT

No repetitive sequences in the immediate proximity.

CLONE 32

[R1, R2, R6]

Driver: ORF2
Plasmid: AlurescueA70D $A_{17}CATTACA_{18}GA_{17}CACACA_{18}$ (T)
Chromosome: 8
5' position: 141,765,251
Strand: plus
DR: AAGAGAAAATTAG
ENDOsite: TCTT/AA

Empty site:

GACCAGAACGTGTGCAAAGGGTTTTTTCTAAAAACTAAAAAGTTACTAATTGGTAATTACTCAAAATAGATT
TTTAAAATCTTCGTAAAATACAGGCAATATTTAGGGAAAAGAGAAGTTTTTCATTGTTCAATGAAAATACCA
CCTTGTGGCCACTATGCCTAGACAAGGGATGCAAGATTAGGCTCTGAATTAATAACAGCTAGTAGACGTTTA
AAATATCTTAACAAATAAATGTAGAAAAAAGCTATTCTAGGATGAATT↑**AAGAGAAAATTAG**↓ATTTAATAA
CGGATTTTAAGGCTGCGAATAATAATGAGAGAATGTGCCAGTGATCAGCAGGTGCTTGAAGGAAAGAAAAAA
CAAGCCTGATAATCTTGCC

Filled site:

GACCAGAACGTGTGCAAAGGGTTTTTTCTAAAAACTAAAAAGTTACTAATTGGTAATTACTCAAAATAGATT
TTTAAAATCTTCGTAAAATACAGGCAATATTTAGGGAAAAGAGAAGTTTTTCATTGTTCAATGAAAATACCA
CCTTGTGGCCACTATGCCTAGACAAGGGATGCAAGATTAGGCTCTGAATTAATAACAGCTAGTAGACGTTTA
AAATATCTTAACAAATAAATGTAGAAAAAAGCTATTCTAGGATGAATTAAGAGAAAATTAG**GGCCGGGCGCG**
GTGGCTCACGCCTGTAATCCCAGCACTTTGGGAGGCCGAGGCGGGCGGATCACGAGGTCAGGAGATCGAGAC
CATCCTGGCTAACACGGTGAAACCCCGTCTCTACTAAAAAAAAAATACAAAAAATTAGCCGGGCGTGGTAGCG
GGCGCCTGTAGTCCCAGCTACTCGGGAGGCTGAGGCAGGGGAATGGCGTGAACCCGGGAGGCGGAGCTTGCA
GTGAGCCGAGATCCCGCC... [NEOcassette] ***AAAAAAAAAAAAAAAAAAAAAAAACATTACAAAAAAAAA***
AAAAAAAAAAAAGAAAAAAAAAAAAAAAAAAAAAAAAAAAAAAAAAAAAAAAAAAAAAAAAAGAGAAAATTAGA
TTTAATAACGGATTTTAAGGCTGCGAATAATAATGAGAGAATGTGCCAGTGATCAGCAGGTGCTTGAAGGAA
AGAAAAAACAAGCCTGATAATCTTGCC

No repetitive sequences in the immediate proximity.

CLONE 33

[U7, U8]

Driver: L1
Plasmid: Alurescue A30D $A_{10}CTA_{10}TACA_{10}$
Chromosome: 9
5' position: 124,049,036
Strand: minus
DR: AAAAAATACCTACTGTA
ENDOsite: TTTT/AA

Empty site:

TCTTGTTGAATCCTCCCCAAAACCCATGGCTGGCATTGTCTCCTTTTATAGACGGAGGAACAAAAGCTCAGA
GAGGTTCAGCCCAAGGTTACACCATTAGGAATTCAGGGCTGTCTGACTCTGAAGTCCGAAGTCTGACTCTCA
CTTCTACACACCTTT↑**AAAAAATACCTACTGTA**↓CACAGTGGCATGAAGATCAGCTCTCATCAGACAGGTAA
GGAGAATGATGGCCACAGTGGCTGTTCAATCACAAATTGTCTCCTTTCTTTCTCTGACTTCC

Filled site:

TCTTGTTGAATCCTCCCCAAAACCCATGGCTGGCATTGTCTCCTTTTATAGACGGAGGAACAAAAGCTCAGA
GAGGTTCAGCCCAAGGTTACACCATTAGGAATTCAGGGCTGTCTGACTCTGAAGTCCGAAGTCTGACTCTCA
CTTCTACACACCTTTAAAAAATACCTACTGTA**GGCCGGGCGCGGTGGCTCACGCCTGTAATCCCAGCACTTT**
GGGAGGCCGAGGCGGGCGGATCACGAGGTCAGGAGATCGAGACCATCCTGGCTAACACGGTGAAACCCCGTC
TCTACTAAAAAAAAAATACAAAAAATTAGCCGGGCGTGGTAGCGGGCGCCTGTAGTCCCAGCTACTCGGGAGG
CTGAGGCAGGGGAATGGCGTGAACCCGGGAGGCGGAGCT... [NEOcassette] ***AAAAAAAAAAAACTAAA***
AAAAAAAAATACAAAAAAAAAAAAAAAAAAAAAAAAAAAAAAAAAAAAAAAAAAAAAAAAAAAAA
AAAAAAAAAAAAAAAAAAAAAAAAAAAAAAATACCTACTGTACACAGTGGCATGAAGATCAGCTCTCATC
AGACAGGTAAGGAGAATGATGGCCACAGTGGCTGTTCAATCACAAATTGTCTCCTTTCTTTCTCTGACTTCC

MIR at the 5'.

CLONE 35
[Q2]

Driver: L1
Plasmid: AlurescueA70D $A_{17}CATTACA_{18}GA_{17}CACACA_{18}$ (T)
Chromosome: 9
5’ position: 129,888,976
Strand: minus
DR: AAAAATAAACCA
ENDOsite: TTTT/AT

Empty site:

CTGCTAGAACACAACCCAACATTCTTTAGAGGAAGACAAGAAAATACAGAATGTCTTTCATGTATCATCCAC
AATGTCCAGTATACAATAAAAAATTATAGACATGTGAAGAAGCAGGACAATATGACCCATAATCAAGAAAAA
AAAGCAGTAAAATCAAACTCACTAATGAGCCAGATGTTGGAATTAGTAGGCAAGGATTTTTAAATGGCTATT
ATAAACATGCACAGGATTTTGAGAACATGAAAGATAGAATGAATCAGTGGATATGGAATCCCAGAAATGGGA
ATTAT↑**AAAAATAAACCA**↓AGTGAAAATTCTAGAACCAAACATTGTAGTATCTGACATAAAATACTTTAGAC
GAAATTAACAGCCGATTAAATGCTGTAGACAAAAGGTTAGCAAATGT

Filled site:

CTGCTAGAACACAACCCAACATTCTTTAGAGGAAGACAAGAAAATACAGAATGTCTTTCATGTATCATCCAC
AATGTCCAGTATACAATAAAAAATTATAGACATGTGAAGAAGCAGGACAATATGACCCATAATCAAGAAAAA
AAAGCAGTAAAATCAAACTCACTAATGAGCCAGATGTTGGAATTAGTAGGCAAGGATTTTTAAATGGCTATT
ATAAACATGCACAGGATTTTGAGAACATGAAAGATAGAATGAATCAGTGGATATGGAATCCCAGAAATGGGA
ATTATAAAAATAAACCA**GGCCGGGCGCGGTGGCTCACGCCTGTAATCCCAGCACTTTGGGAGGCCGAGGCGG
GCGGATCACGAGGTCAGGAGATCGAGACCATCCTGGCTAACACGGTGAAACCCCGTCTCTACTAAAAAAAAA
ATACAAAAAATTAGCCGGGCGTGGTAGCGGGCGCCTGTAGTCCCAGCTACTCGGGAGGCTGAGGCAGGGGAA
TGGCGTGAACCCGGGAGGCGGAGCTTGCAGTGAGCCGAGATCCCGCCACTGCACTCCA… [NEOcassette
]** ***AAAAAAAAAAAAAAAAAAAAAAAACATTACAAAAAAAAAAAAAAAAAAAAAAAAAAAAAAAAAAAAAAAAAAAAA
AAAAAAAAAAAAAAAAAAAAAAAAAAAAAAAAAAAAAAAAAAAAAAAAAAAAAAAAGGAAAAAAAAAAAAAA
AAAAAAAAAAAAAAAA***TAAACCAAGTGAAAATTCTAGAACCAAACATTGTAGTATCTGACATAAAATACTTT
AGACGAAATTAACAGCCGATTAAATGCTGTAGACAAAAGGTTAGCAAATGT

LINE1 flanks insertion site.

CLONE 36

[N34, N50]

Driver: L1
Plasmid: AlurescueA70D   A$_{17}$CATTACA$_{18}$GA$_{17}$CACACA$_{18}$ (T)
Chromosome: 6
5' position: 68,059,409
Strand: plus
DR: AAAGTATTTTATT
ENDOsite: CTTT/GA

Empty site:

ATCAAAACAAACTTAGGATTAGAAGGATCATAAGTATAACCCAATATGGCATTTCCTGTACTCTCATAGAGA
TATCATTAGGTAAAAAGATTTATGTTGCATCTAAGAACACAAAGCAGATGACAGGTGCTGCCAATAACTGAT
ATGAGCATATTCAGTTAGCAAGCCAGGACTCAGAAACAGCTTTTCCAGTTATCCAGTCTCTGTTCCACAGAT
AAATAGAATAATAGATTAATTCTATCAATTTTTTTCCCACACCCCAGTGATTTGTGGAGAAAATTAAAGCAT
TTC↑**AAAGTATTTTATT**↓AAATTCAAATATTATCAGGAAAGTTAAAAGTTTGTTGTATCAATACTAATATGT
AAGATAATTTTCTTATACTATAAAAAAGATACATATTGGAAAGGTA

Filled site:

ATCAAAACAAACTTAGGATTAGAAGGATCATAAGTATAACCCAATATGGCATTTCCTGTACTCTCATAGAGA
TATCATTAGGTAAAAAGATTTATGTTGCATCTAAGAACACAAAGCAGATGACAGGTGCTGCCAATAACTGAT
ATGAGCATATTCAGTTAGCAAGCCAGGACTCAGAAACAGCTTTTCCAGTTATCCAGTCTCTGTTCCACAGAT
AAATAGAATAATAGATTAATTCTATCAATTTTTTTCCCACACCCCAGTGATTTGTGGAGAAAATTAAAGCAT
TTCAAAGTATTTTATT**GGCCGGGCGCGGTGGCTCACGCCTGTAATCCCAGCACTTTGGGAGGCCGAGGCGGG**
CGGATCACGAGGTCAGGAGATCGAGACCATCCTGGCTAACACGGTGAAACCCCGTCTCTACTAAAAAAAAAT
ACAAAAAATTAGCCGGGCGTGGTAGCGGGCGCCTGTAGTCCCAGCTACTCGGGAGGCTGAGGCAGGGGAATG
GCGTGAACCCGGGAGGCGGAGCT… [NEOcassette] ***AAAAAAAAAAAAAAAAAAAAAAAACATTACA***
AAAAAAAAAAAAAAAAAGAAAAAAAAAAAAAAAAAAAAAAAAAAAAAAAAAAAAAAAAAAAGTATTTTA
TTAAATTCAAATATTATCAGGAAAGTTAAAAGTTTGTTGTATCAATACTAATATGTAAGATAATTTTCTTAT
ACTATAAAAAAGATACATATTGGAAAGGTA

No repetitive sequences in the immediate proximity.

CLONE 38

[N28]

Driver: L1
Plasmid: AlurescueA70D $A_{17}CATTACA_{18}GA_{17}CACACA_{18}$ (T)
Chromosome: 4
5' position: 151,379,841
Strand: minus
DR: AAAACAAGAAAGAGC
ENDOsite: TTTT/AT

Empty site:

ACTTATGGTCTATATGCTCAGTCAGCTCTTCAGTGTCTCATTCTTAAAAATGGAGGTGCAGCCAAGTATTAG
CAAACATTTAAAGACAACCTCTAATGTGAAAGATGACAAAGACCAAAGCAAATAGAAAATCAAAACAAAACC
AGAAGAAACAGAGGCAATTTATGAAACAGAAGTGTTTTTTTAAAAAAAAAAAAACTAGAATAAATATTTTCA
GATAAAGGGAAACTATTGCATTAATAGAACAAGAAAATTATGCTATGGAAAGTGAATAATCAT↑**AAAACAAG
AAAGAGC**↓AGTATAAAAATTAATAGAAGAGTTATAAATGCAGATATCTTCTAAAAGGAATATCAACATGATG
TGGAGAAAACAGCCTGGAAAGATAAAATTAGACCAG

Filled site:

ACTTATGGTCTATATGCTCAGTCAGCTCTTCAGTGTCTCATTCTTAAAAATGGAGGTGCAGCCAAGTATTAG
CAAACATTTAAAGACAACCTCTAATGTGAAAGATGACAAAGACCAAAGCAAATAGAAAATCAAAACAAAACC
AGAAGAAACAGAGGCAATTTATGAAACAGAAGTGTTTTTTTAAAAAAAAAAAAACTAGAATAAATATTTTCA
GATAAAGGGAAACTATTGCATTAATAGAACAAGAAAATTATGCTATGGAAAGTGAATAATCATAAAACAAGA
AAGAGCT**GGCCGGGCGCGGTGGCTCACGCCTGTAATCCCAGCACTTTGGGAGGCCGAGGCGGGCGGATCACG
AGGTCAGGAGATCGAGACCATCCTGGCTAACACGGTGAAACCCCGTCTCTACTAAAAAAAAATACAAAAAAT
TAGCCGGGCGTGGTAGCGGGCGCCTGTAGTCCCAGCTACTCGGGAGGCTGAGGCAGGGGAATGGCGTGAACC
CGGGAGGCGGAGCTTGCAGTGAGCCGAGATCCC… [NEOcassette]** ***AAAAAAAAAAAAAAAAAAAAAA
AAAAAAAAAAAAAAAAAAAAAAAAAAAAAAAAACATTACAAAAAAAAAAAAAAAAAAAAAAAAAAAA
AAAAAAAAAAAA***CAAGAAAGAGCAGTATAAAAATTAATAGAAGAGTTATAAATGCAGATATCTTCTAAAAG
GAATATCAACATGATGTGGAGAAAACAGCCTGGAAAGATAAAATTAGACCAG

LINE1 flanks insertion site.

An extra T added immediately 5' of Alu.

CLONE 39

[N31, N37,N51]

Driver: L1
Plasmid: AlurescueA70D $A_{17}CATTACA_{18}GA_{17}CACACA_{18}$ (T)
Chromosome: 5
5’ position: 21,542,539
Strand: plus
DR: AAGATATATGGATTTA
ENDOsite: TCTT/AA

Empty site:

ATCCAAATAGATAAAGTTTCAGGCCATTCTTCGAAAGCATATTTCCCATAAACCACATCTAAAACTAATTAT
TTTGAAATGTTAATTATCCCAATAAATGAAAGTTAAGTGGTTTATAATATCATTATATTCCTCCTACAAACG
TGTTTTAAGCAATAAGGAAAACAATGTACTATATGAGATTTTTTTCAAAAACAGAATGCACATTTTGAATTT
ACACAAACTTATACTGAGGAAAGTGTGACAAAATATTTTAAATAATTATTTTAATTTGTCATTCAAGTTTTC
ATTTT↑**AAGATATATGGATTTA**↓GAATTTATTTTATTTTATTTTTTAGCATTCATCGGTACATTTTATGTAT
TTTTTTAACCTTTAAGTTCAGGGTTACATGTGCAGGTTTGTTATATAGGTA

Filled site:

ATCCAAATAGATAAAGTTTCAGGCCATTCTTCGAAAGCATATTTCCCATAAACCACATCTAAAACTAATTAT
TTTGAAATGTTAATTATCCCAATAAATGAAAGTTAAGTGGTTTATAATATCATTATATTCCTCCTACAAACG
TGTTTTAAGCAATAAGGAAAACAATGTACTATATGAGATTTTTTTCAAAAACAGAATGCACATTTTGAATTT
ACACAAACTTATACTGAGGAAAGTGTGACAAAATATTTTAAATAATTATTTTAATTTGTCATTCAAGTTTTC
ATTTTAAGATATATGGATTTA**GGCCGGGCGCGGTGGCTCACGCCTGTAATCCCAGCACTTTGGGAGGCCGAG**
GCGGGCGGATCACGAGGTCAGGAGATCGAGACCATCCTGGCTAACACGGTGAAACCCCGTCTCTACTAAAAA
AAAAATACAAAAAATTAGCCGGGCGTGGTAGCGGGCGCCTGTAGTCCCAGCTACTCGGGAGGCTGAGGCAGG
GGAATGGCGTGAACCCGGGAGGCGGAGCTTGCAGTGAGCCGAGATCCCGCC… [NEOcassette]*AAAAA*
AAAAAAAAAAAAAAAAAAAAAAAAAAAAAAAAAAAAAAAAACATTACAAAAAAAAAAAAAAAAAAAAAAAAA
AAGATATATGGATTTAGAATTTATTTTATTTTATTTTTTAGCATTCATCGGTACATTTTATGTATTTTTTTA
ACCTTTAAGTTCAGGGTTACATGTGCAGGTTTGTTATATAGGTA

LINE1 fragments both 5’ and 3’ of insertion site.

CLONE 42

[I27]

Driver: L1
Plasmid: AlurescueA70D $A_{17}CATTACA_{18}GA_{17}CACACA_{18}$ (T)
Chromosome: 4
5' position: 184,194,385
Strand: minus
DR: AAAAAAAAAA
ENDOsite: TTTT/GA

Empty site:

CGTCTCTACTAAAAATACAAAATTAGCTGGGCATGGTGGTGCATGCCTGTAATCCCAGCTACTCGGGAGGCT
GAGGCAGGAGAATCACTTGAACCTGGGAGGGAGAGGTTGTGGTTAGCCGAGATTATGCCATTGTACTCCAGC
CTGGGCAATAAGAAGAAAACTCCGTCTCAAAAGAAAATAATGATAATAAATTAAAAATAAATAAATAACATC
GTTTAAAAATAGACAAAGAATGTGAAAAGACATTTCTC↑**AAAAAAAAAA**↓GATATACTAATGGCCAATAAGG
CTGTGATGCTCACCATCATTAGTCATTAGGGAAATGTCAATCAAAGCCACAGTGAAAACACCACTTCACACC
CACAAGAA

Filled site:

CGTCTCTACTAAAAATACAAAATTAGCTGGGCATGGTGGTGCATGCCTGTAATCCCAGCTACTCGGGAGGCT
GAGGCAGGAGAATCACTTGAACCTGGGAGGGAGAGGTTGTGGTTAGCCGAGATTATGCCATTGTACTCCAGC
CTGGGCAATAAGAAGAAAACTCCGTCTCAAAAGAAAATAATGATAATAAATTAAAAATAAATAAATAACATC
GTTTAAAAATAGACAAAGAATGTGAAAAGACATTTCTCAAAAAAAAAA**GGCCGGGCGCGGTGGCTCACGCCT**
GTAATCCCAGCACTTTGGGAGGCCGAGGCGGGCGGATCACGAGGTCAGGAGATCGAGACCATCCTGGCTAAC
ACGGTGAAACCCCGTCTCTACTAAAAAAAAAAAATACAAAAAATTAGCCGGGCGTGGTAGCGGGCGCCTGTAG
TCCCAGCTACTCGGGAGGCTGAGGCAGGGGAATGGCGTGAACCCGGGAGGCGGAGCTTGCAGTGAGCCGAGA
TCCCGCCACTGCACTCC... [NEOcassette] ***AAAAAAAAAAAAAAAAACATTACAAAAAAAAAAAAAAAAA***
AAGAAAAAAAAAAAAAAAAAAAAAAAAAAAAAAAAAAAAAAAAAAAAAAAAAAAAAAAAAAAAAAAAAAAAGAT
ATACTAATGGCCAATAAGGCTGTGATGCTCACCATCATTAGTCATTAGGGAAATGTCAATCAAAGCCACAGT
GAAAACACCACTTCACACCCACAAGAA

Alu 5' and LINE1 flanks insertion site.

CLONE 44

[I28]

Driver: L1
Plasmid: AlurescueA70D $A_{17}CATTACA_{18}GA_{17}CACACA_{18}$ (T)
Chromosome: 5
5' position: 33,309,606
Strand: plus
DR: AAAACCATCCA
ENDOsite: TTTT/AA

Empty site:

CCCAACATAACTCGTTAACCATTAAACTGTTCCTCTAATATCTCTTCAGGGGCCAAGTCCAGGCTGCAAGCA
ATGTAAATCCGTTCTACTGGAAAGAGTTGTATACAACAGAATGAAAACTGGCTCAAGGTCAAGAAAAAAAAGA
ACAGAAAACAAGGCTACAAAAGACAGAGTGATTACATTCATACATCATTTTTGCAGTTCTTTTCTGACTTTC
CTCTCAAAATTACAAAGAAGCATACTTT↑**AAAACCATCCA**↓AGTCCATGGTTTCCTTTATTCTTTCATTTAG
AATCCTAGCTCTACACATTCAGAAACTATGACATGATTTTAAAATGCTACATGAATATATATATATGCA

Filled site:

CCCAACATAACTCGTTAACCATTAAACTGTTCCTCTAATATCTCTTCAGGGGCCAAGTCCAGGCTGCAAGCA
ATGTAAATCCGTTCTACTGGAAAGAGTTGTATACAACAGAATGAAAACTGGCTCAAGGTCAAGAAAAAAAAGA
ACAGAAAACAAGGCTACAAAAGACAGAGTGATTACATTCATACATCATTTTTGCAGTTCTTTTCTGACTTTC
CTCTCAAAATTACAAAGAAGCATACTTTAAAACCATCCA**GGCCGGGCGCGGTGGCTCACGCCTGTAATCCCA**
GCACTTTGGAGGCCGAGGCGGGCGGATCACGAGGTCAGGAGATCGAGACCATCCTGGCTAACACGGTGAAA
CCCCGTCTCTACTAAAAAAAAAATACAAAAAAATTAGCCGGGCGTGGTAGCGGGCGCCTGTAGTCCCAGCTACT
CGGGAGGCTGAGGCAGGGGAATGGCGTGAACCCGGGAGGCGGAGCTTGCAGTGAGCCGAGATCCCGCCAC… [
NEOcassette]***AAAAAAAAAAAAAAAAAAAAAAAAAAAAAAACATTACAAAAAACATTACAAAAAAAAAA***
AAAAAAAAAAAAAAAAAAAAAAAAAAAAAAAAAAACCATCCAAGTCCATGGTTTCCTTTATTCTTTCATTTA
GAATCCTAGCTCTACACATTCAGAAACTATGACATGATTTTAAAATGCTACATGAATATATATATATGCA

No repetitive sequences in the immediate proximity.

CLONE 45

[I18]

Driver: L1
Plasmid: AlurescueA70D $A_{17}CATTACA_{18}GA_{17}CACACA_{18}$ (T)
Chromosome: 21
5’ position: 28,745,144
Strand: minus
DR: AAAATCTCAGTTTC
ENDOsite: TTTT/AA

Empty site:

TTCCAAAAATTCAAAATAGATAATGATGTCTCCTTGCTTAGAACCTTCAACAGCTTTCCATTTCGCTTATAA
TATACACCTCTTACAATGACCAGTCTCTACAGGATCTGGCCCTACCTGCCTGTCTGATTACACATTATACCA
CTTTTACTTTGCTCACTGCAGCCACATTGACCTTTTTCCAGGTTATTGAGTGTATCAGGGTGGCTTTGCAAT
GCTTGATTTCTCTTGTCATCTCCACACGGGTAATTCCATGATACAATT↑**AAAATCTCAGTTTC**↓AACTTAAA
TCCCTCTTACAGCCAGCTTTATTTACATAGTTTGGAGGTGCCCAATGATTACTTTTTGGTTAAATTACCCTC
TTTCATTTTTATCATAATAT

Filled site:

TTCCAAAAATTCAAAATAGATAATGATGTCTCCTTGCTTAGAACCTTCAACAGCTTTCCATTTCGCTTATAA
TATACACCTCTTACAATGACCAGTCTCTACAGGATCTGGCCCTACCTGCCTGTCTGATTACACATTATACCA
CTTTTACTTTGCTCACTGCAGCCACATTGACCTTTTTCCAGGTTATTGAGTGTATCAGGGTGGCTTTGCAAT
GCTTGATTTCTCTTGTCATCTCCACACGGGTAATTCCATGATACAATTAAAATCTCAGTTTC**GGCCGGGCGC**
GGTGGCTCACGCCTGTAATCCCAGCACTTTGGGAGGCCGAGGCGGGCGGATCACGAGGTCAGGAGATCGAGA
CCATCCTGGCTAACACGGTGAAACCCCGTCTCTACTAAAAAAAAAATACAAAAAATTAGCCGGGCGTGGTAG
CGGGCGCCTGTAGTCCCAGCTACTCGGGAGGCTGAGGCAGGGGAATGGCGTGAACCCGGGAGGCGGAGCTTG
CAGTGAGCCGAGATCCCGCCACTGCACTCCAGC…[NEOcassette]*AAAAAAAAAAAAAAAAAAACATTA*
CAAAAAAAAAAAAAAAAAAAAAAAAGAAAAAAAAAAAAAAAAAAAAAAAAAAAAAAAAAAAAAAAAAAAAAAAA
AAAAAAAAAAAAAAAAAAAAAAAAAAAAAAAAAAAAAAAAAAAAAAAAAAAAATCTCAGTTTCAACTTAAAT
CCCTCTTACAGCCAGCTTTATTTACATAGTTTGGAGGTGCCCAATGATTACTTTTTGGTTAAATTACCCTCT
TTCATTTTTATCATAATAT

LINE1 at the 5’.

CLONE 46

[I7, I20]

Driver: L1
Plasmid: AlurescueA70D $A_{17}CATTACA_{18}GA_{17}CACACA_{18}$ (T)
Chromosome: 20
5' position: 8,779,579
Strand: plus
DR: GAAAAATTAGATTCT
ENDOsite: TTTC/AA

Empty site:

GACACATAAATCAACAGATCAGCAACAATGCCTGAGTATTACACAATGACTGCTGACTCCCCCATCAGCCCT
CCAATTCTCAGAAGAATCTCCATGGAACCCACAATAGCTGGAAACATACAGAAAGGGAACTCTGGGAAATAT
AGTTTGGCTGAGACAGTTGGCACAATAAGAAGCATCACACTGTTAAGTAAAAATCAGAAAATGTTTTATTTC
AAAAATACTCTGTGTCTCTT↑**GAAAAATTAGATTCT**↓AAGATTATAATAGAAACATGCAAAGAATTTCTAAA
GCTAAGAAGAAGCTGTCCCTTAGGGCTGCATGGTACTCCCTAGGCTGTTTCAAAGATTGCTATTG

Filled site:

GACACATAAATCAACAGATCAGCAACAATGCCTGAGTATTACACAATGACTGCTGACTCCCCCATCAGCCCT
CCAATTCTCAGAAGAATCTCCATGGAACCCACAATAGCTGGAAACATACAGAAAGGGAACTCTGGGAAATAT
AGTTTGGCTGAGACAGTTGGCACAATAAGAAGCATCACACTGTTAAGTAAAAATCAGAAAATGTTTTATTTC
AAAAATACTCTGTGTCTCTTGAAAAATTAGATTCT↓…**[NEOcassette]*AAAAAAAAAAAAAAAAAA***
CATTACAAAAAAAAAAAAAAAAAAAAAAAAAAAAAAAAAAAAAAAAAAAGAAAAATTAGATTCTAAGA
TTATAATAGAAACATGCAAAGAATTTCTAAAGCTAAGAAGAAGCTGTCCCTTAGGGCTGCATGGTACTCCCT
AGGCTGTTTCAAAGATTGCTATTG

LTR at the 5'.

CLONE 47

[I3]

Driver: L1
Plasmid: AlurescueA70D $A_{17}CATTACA_{18}GA_{17}CACACA_{18}$ (T)
Chromosome: 2
5’ position: 170,561,870
Strand: plus
DR: AAGAGATAATT
ENDOsite: TCTT/AA

Empty site:

TTTGTGTCAATTTAAAAAAAATTTTAAAGGAAACACTAGAACTGAAAATTAGCATGCTTGAAATTAGGAGAC
TGATGGATAGAATTGACAGAACAGGCATAGCTGAAGAAAGAATTGGTGAATGAACACAAATTAGAAAAAAAA
ATCCAGAATGATGCAGAGAGACAAAATAATAAAAATACAAAAGTTGAGACATAGATTTAGTAAAAGATCTAA
CAGAGGAAATTTATGTCCCAAGAGAGGAGAGAGAAAATGAAACATAAGCAaTAGTTT↑**AAGAGATAATT**↓AT
TGGCTGGGCGTGGTGGCTCATGCCTGTAATCTCAGCACTTTGGGAGGCCTAGGCAGGTGGATCACAAGGTCA
GGAGATCGAGACCATCCTGGCTAACA

Filled site:

TTTGTGTCAATTTAAAAAAAATTTTAAAGGAAACACTAGAACTGAAAATTAGCATGCTTGAAATTAGGAGAC
TGATGGATAGAATTGACAGAACAGGCATAGCTGAAGAAAGAATTGGTGAATGAACACAAATTAGAAAAAAAA
ATCCAGAATGATGCAGAGAGACAAAATAATAAAAATACAAAAGTTGAGACATAGATTTAGTAAAAGATCTAA
CAGAGGAAATTTATGTCCCAAGAGAGGAGAGAGAAAATGAAACATAAGCAaTAGTTTAAGAGATAATT**GGCC**
GGGCGCGGTGGCTCACGCCTGTAATCCCAGCACTTTGGGAGGCCGAGGCGGGCGGATCACGAGGTCAGGAGA
TCGAGACCATCCTGGCTAACACGGTGAAACCCCGTCTCTACTAAAAAAAAATACAAAAAATTAGCCGGGCGT
GGTAGCGGGCGCCTGTAGTCCCAGCTACTCGGGAGGCTGAGGCAGGGGAATGGCGTGAACCCGGGAGGCGGA
GCTTGCAGTGAGCCGAGATCCCGCCACTGCACTCC… **[NEOcassette]** ***AAAAAAAAAAAAAAAAAAAAAA***
AAAAAAAAAACATTACAAAAAAAAAAAAAAAAAAAAAAAAAAAAAAAAAAAAAAAAAAAAAAAAAAAAAAAAAAA
AAAAGAAAAAAAAAAAAAAAAAAAAAAAAAAAAAAACACACAAAAAAAAAAAATTAAAAAAAAAAAAAAAAA
AAAAAAAAAAAAAAAAAAAAAAAAAAAGAGATAATTATTGGCTGGGCGTGGTGGCTCATGCCTGTAATCTCA
GCACTTTGGGAGGCCTAGGCAGGTGGATCACAAGGTCAGGAGATCGAGACCATCCTGGCTAACA

LINE1 at the 5; Alu at the 3’.

CLONE 48

[I5, I21, I22, L6]

Driver: L1
Plasmid: AlurescueA70D $A_{17}CATTACA_{18}GA_{17}CACACA_{18}$ (T)
Chromosome: 4
5' position: 15,638,359
Strand: minus
DR: AGAAATGTAATTGTA
ENDOsite: TTCT/AT

Empty site:

ATACTTGTTGATTTAAAGCTGTATAACCTGTTTTGTTCCTTTTTCAAGTGATGCTGCTTAATGCTACAAAGT
GAGTATTGTTTTTTCTGTGATGCCTTTGTAAGAGGAAGTTAATTCTGTGTTCATTATTGTAGCTCCCACTGA
TGAAGTATGATTTGGGCCATGATAGTTTAACCCTACTGGTTCTTCACAAGTTTAACATCCCCATGTTACCTC
TAATACATGTTATTTCATTTTCACATCTTGCAAGCAGGAAGGAATTGACTTCAGGCACTGGATTTTGTTTAT
↑**AGAAATGTAATTGTA**↓ATTTCTTATTGCATATGCTCTTTAGCCTTTACTGGTAAAGTTTTTCTTTTTTATT
TCAGAAGCAGAAGTGTCAGAACACTCCACAGGTATAACCCATCTT

Filled site:

ATACTTGTTGATTTAAAGCTGTATAACCTGTTTTGTTCCTTTTTCAAGTGATGCTGCTTAATGCTACAAAGT
GAGTATTGTTTTTTCTGTGATGCCTTTGTAAGAGGAAGTTAATTCTGTGTTCATTATTGTAGCTCCCACTGA
TGAAGTATGATTTGGGCCATGATAGTTTAACCCTACTGGTTCTTCACAAGTTTAACATCCCCATGTTACCTC
TAATACATGTTATTTCATTTTCACATCTTGCAAGCAGGAAGGAATTGACTTCAGGCACTGGATTTTGTTTAT
AGAAATGTAATTGTA**GGCCGGGCGCGGTGGCTCACGCCTGTAATCCCAGCACTTTGGGAGGCCGAGGCGGGC**
GGATCACGAGGTCAGGAGATCGAGACCATCCTGGCTAACACGGTGAAACCCCGTCTCTACTAAAAAAAAATA
CAAAAAATTAGCCGGGCGTGGTAGCGGGCGCCTGTAGTCCCAGCTACTCGGGAGGCTGAGGCAGGGGAATGG
CGTGAACCCGGGAGGCGGAGCTTGCAGTGAGCCGAGATCCCGCCACTGCACTCCAGCCT… [NEOcassett
e]*AAAAAAAAAAAAAAAAAAAAAAAAAAAAAAAAAAAAAAAAAAACATTACAAAAAAAAAAAAAAAAAAA*
AAAAAAAAAAAAAAAAAAAAAAAAAAAAAAAAAAAAAAAAAAAAAAAAAAAAAAAAAAAAGAAATGTAATTG
TAATTTCTTATTGCATATGCTCTTTAGCCTTTACTGGTAAAGTTTTTCTTTTTTATTTCAGAAGCAGAAGTG
TCAGAACACTCCACAGGTATAACCCATCTT

No repetitive sequences in the immediate proximity.

CLONE 54

[K23, JK24]

Driver: ORF2
Plasmid: AlurescueA70D $A_{17}CATTACA_{18}GA_{17}CACACA_{18}$ (T)
Chromosome: 6
5' position: 136,981,148
Strand: minus
DR: AAAAATTGTAGATT
ENDOsite: TTTT/AT

Empty site:

TAATTTTTGTGTCTAAAGTTATTATAGCATTTTATTCTTGCGAAATATATATACTGTATGCATTTATACTTC
AACTGATAGAATTTCAGCGTAAAAAAGGTAAGATTTTCCTTTTAAGTTATTTAAGAATCCATGTTGTAAAAA
GACAAAGAAGAAGGTTACATAACAATGTTTGTATTATGACCTTTTAATTTATGCATATACTGACATATAT↑**A**
AAAATTGTAGATT↓TATGTACATTAAAGTGACCATAGCAGTTATCCTTGGGTTTTCAAATTAAAGGTTAATT
TTCACTTTCTTCTGTGTACCTTTCTGGTTAGTCTGACTTACT

Filled site:

TAATTTTTGTGTCTAAAGTTATTATAGCATTTTATTCTTGCGAAATATATATACTGTATGCATTTATACTTC
AACTGATAGAATTTCAGCGTAAAAAAGGTAAGATTTTCCTTTTAAGTTATTTAAGAATCCATGTTGTAAAAA
GACAAAGAAGAAGGTTACATAACAATGTTTGTATTATGACCTTTTAATTTATGCATATACTGACATATATAA
AAATTGTAGATT**GGCCGGGCGCGGTGGCTCACGCCTGTAATCCCAGCACTTTGGGAGGCCGAGGCGGGCGGA**
TCACGAGGTCAGGAGATCGAGACCATCCTGGCTAACACGGTGAAACCCCGTCTCTACTAAAAAAAAAAATACA
AAAAATTAGCCGGGCGTGGTAGCGGGCGCCTGTAGTCCCAGCTACTCGGGAGGCTGAGGCAGGGGAATGGCG
TGAACCCGGGAGGCGGAGCTTGCAGTGAGCCGAGATCCCGCCACTGCACTCC… [NEOcassette]*AAAA*
AAAAAAAAAAAAAAAACATTACAAAAAAAAAAAAAAAAAAAAAAAAAAAAAAAAAAAATTGTAGATTTATGT
ACATTAAAGTGACCATAGCAGTTATCCTTGGGTTTTCAAATTAAAGGTTAATTTTCACTTTCTTCTGTGTAC
CTTTCTGGTTAGTCTGACTTACT

No repetitive sequences in the immediate proximity.

CLONE 55

[K33]

Driver: ORF2
Plasmid: AlurescueA70D $A_{17}CATTACA_{18}GA_{17}CACACA_{18}$ (T)
Chromosome: 6
5' position: 136,558,667
Strand: plus
DR: AAAAATTAAAAATGTG
ENDOsite: TTTT/AA

Empty site:

CTCCCAAGTTGATAAGCCCACCTCACACAAAGAACATGCGATCAGCTTTTCCTTGCCTCATTTTAAATAAGA
ATCACCAGATGCTTACCCAAGGCTACAAAACTAAAGATAGAGATCAAAGCTAAAAGGAAAAAAGAAACTCAC
AAAAAAATACAGTAATTCAAGAACAGAGGAAACTTAAAACAAACCTACACTTTTAATACTGTCAGTGGGATAA
GACCAACAAAACCAAAAAGATTTTAACAAGAAGCATTTGAAGAAAAAGAAACAGCTCTT↑**AAAAATTAAAAA
TGTG**↓GGCCAGGTGTGGTGGCTCACACCTGTAATCCCAGCACTTTGGGAGGCCGAGGCCGGCAGATCACAAG
GTCAGGAGTTCATGACCAGCCTGGCCAACATGG

Filled site:

CTCCCAAGTTGATAAGCCCACCTCACACAAAGAACATGCGATCAGCTTTTCCTTGCCTCATTTTAAATAAGA
ATCACCAGATGCTTACCCAAGGCTACAAAACTAAAGATAGAGATCAAAGCTAAAAGGAAAAAAGAAACTCAC
AAAAAAATACAGTAATTCAAGAACAGAGGAAACTTAAAACAAACCTACACTTTTAATACTGTCAGTGGGATAA
GACCAACAAAACCAAAAAGATTTTAACAAGAAGCATTTGAAGAAAAAGAAACAGCTCTTAAAAATTAAAAAT
GTG**GGCCGGGCGCGGTGGCTCACGCCTGTAATCCCAGCACTTCGGGAGGCCGAGGCGGGCGGATCACGAGGT
CAGGAGATCGAGACCATCCTGGCTAACACGGTGAAACCCCGTCTCTACTAAAAAAAAATACAAAAAATTAGC
CGGGCGTGGTAGCGGGCGCCTGTAGTCCCAGCTACTCGGGAGGCTGAGGCAGGGGAATGGCGTGAACCCGGG
AGGCGGAGCTTGCAGTGAGCCGAGATCCCGCCAC... [NEOcassette]** ***AAAAAAAAAAAAAAAAAAACAT
TACAAAAAAAAAAAAAAAAAAAAAAGAAAAAAAAAAAAAAAAAAAAAAA***TTAAAAATGTGGGCCAGGT
GTGGTGGCTCACACCTGTAATCCCAGCACTTTGGGAGGCCGAGGCCGGCAGATCACAAGGTCAGGAGTTCAT
GACCAGCCTGGCCAACATGG

LINE1 at the 5'; Alu at the 3'.

CLONE 56

[K3, K20]

Driver: ORF2
Plasmid: AlurescueA70D $A_{17}CATTACA_{18}GA_{17}CACACA_{18}$ (T)
Chromosome: 2
5' position: 234,312,608
Strand: minus
DR: AAAATTTAATGTGTG
ENDOsite: TTTT/AC

Empty site:

AAAAAACGATCATTACAGAGACTCATGATATGGGGAATCTTATGATAAAACAGCAAATAAAGCCAAAATACA
AAATTATACATGTATATAACACATGTAAATTATACATGTATATAACACATGTAAACACAAAATTATACATGT
ATATAACACATGTAAAATGTAATTTTGTAACACAAAACACAAAATTTAACTTTGTAACACAAAACACAAAAT
TATACATGTATATAACACATGT↑**AAAATTTAATGTGTG**↓CCTATTAAAAAGTCTGAAGGGAGGAAGGGCGCA
GTGGCTCACGCCTGTAATCCCAGCACTTTGGGAATCCAAGGTGGGCGGATCACGAGGTCAGGAGTTC

Filled site:

AAAAAACGATCATTACAGAGACTCATGATATGGGGAATCTTATGATAAAACAGCAAATAAAGCCAAAATACA
AAATTATACATGTATATAACACATGTAAATTATACATGTATATAACACATGTAAACACAAAATTATACATGT
ATATAACACATGTAAAATGTAATTTTGTAACACAAAACACAAAATTTAACTTTGTAACACAAAACACAAAAT
TATACATGTATATAACACATGTAAAATTTAATGTGTG**GGCCGGGCGCGGTGGCTCACGCCTGTAATCCCAGC**
ACTTTGGGAGGCCGAGGCGGGCGGATCACGAGGTCAGGAGATCGAGACCATCCTGGCTAACACGGTGAAACC
CCGTCTCTACTAAAAAAAAAAATACAAAAAATTAGCCGGGCGTGGTAGCGGGCGCCTGTAGTCCCAGCTACTC
GGGAGGCTGAGGCAGGGGAATGGCGTGAACCCGGGAGGCGGAGCTTGCAGTGAGCCGAGATCCCGCCACTGC
AC…[NEOcassette]*AAAAAAAAAAAAAAAAAAAAAAAAAAAAAAAAAACATTACAAAAAAAAAAAAAA*
AAAAAAAAAAAAAAAAAAAAAAAAAAAAAAAAAAAAAAAAAAAAAAAAAAAAAAAAAAAATTTAATGTGTGC
CTATTAAAAAGTCTGAAGGGAGGAAGGGCGCAGTGGCTCACGCCTGTAATCCCAGCACTTTGGGAATCCAAG
GTGGGCGGATCACGAGGTCAGGAGTTC

Alu at the 3'.

CLONE 57

[JK27]

Driver: ORF2
Plasmid: AlurescueA70D $A_{17}CATTACA_{18}GA_{17}CACACA_{18}$ (T)
Chromosome: 1
5' position: 41,408,564; 3' position: 41,507,429
Strand: minus
DR: none
ENDOsite: unknown

Empty site:

AGAACTAGAAATAAATGTTTGCTACAACAAGCCATTGAAGTACTGGGGTTGTTTTTTTCCTGTAGGAAAGGA
TGAAAACACACCCTATGCTAAATGGGATAGGACAGTGATGAATAGACCTTGGTATGCTCATGTATGGTACAC
AATAGACAGCAATCAAGATGAATGAACTGCAGGGACATGCCTCAGATATGAATTAATCTTGCCAATCTAATA
GTAAGTGAGAAAAGTAGATCTCAAAAGATTATAGGCTGGGCACAGTGGCTCACGCCTGTAATCCCAGCACTT
TGGGAGGC↓TGAGGTGGGCGGATCACAAGGTCAGGAGCTCGAGATCAGCCTGGACAACATAGTGAAACCCCG
T

Filled site:

AGAACTAGAAATAAATGTTTGCTACAACAAGCCATTGAAGTACTGGGGTTGTTTTTTTCCTGTAGGAAAGGA
TGAAAACACACCCTATGCTAAATGGGATAGGACAGTGATGAATAGACCTTGGTATGCTCATGTATGGTACAC
AATAGACAGCAATCAAGATGAATGAACTGCAGGGACATGCCTCAGATATGAATTAATCTTGCCAATCTAATA
GTAAGTGAGAAAAGTAGATCTCAAAAGATTATAGGCTGGGCACA**GTGGCTCACGCCTGTAATCCCAGCACTT**
TGGGAGGCCGAGGCGGGCGGATCACGAGGTCAGGAGATCGAGACCATCCTGGCTAACACGGTGAAACCCCGT
CTCTACTAAAAAAAAAATACAAAAAATTAGCCGGGCGTGGTAGCGGGCGCCTGTAGTCCCAGCTACTCGGGA
GGCTGAGGCAGGGGAATGGCGTGAACCCGGGAGGCGGAGCTTGCAGTGAGCCGAGATCCCGCCACTGCACTC
CAGC…[NEOcassette][NoAtail]AACAGTATTAAGCAGGAGATTGTATTGATTTCACTGGTC
CCAGTGACTATTAAGTTTAGCTGTAATTGAAATCTACCCAGCATTAAACTCTGAGTCTGCTGTTTT
GGGATTCTCAGGTTCCTCTCTGTTAGAGGAAGTGGTTCATTGCCCATGACACTGGTTCCTTGTGCC
TTTCTTTCCAGTTGTCATCCTGGTCTTCTG

LINE1 5'; Alu flanking insertion site.

Inserted by recombination with a pre-existing truncated Alu: missing between 10-47 bp of 5' Alu sequence.

Pre-insertion site sequence is identical to the Alu rescue vector across this range, making the ENDO cleavage site uncertain.

Post-insertion genomic flanking site map to locations of chr. 1 sequence in the human reference genome that are separated by 98,865 bases (detailed analysis shown in supplemental Figure S1).

CLONE 58

[K21, K32]

Driver: ORF2
Plasmid: AlurescueA70D $A_{17}CATTACA_{18}GA_{17}CACACA_{18}$ (T)
Chromosome: 1
5' position: 109,491,736
Strand: plus
DR: AAAAGTTTAATATC
ENDOsite: TTTT/AT

Empty site:

AAGTGAAGCAATTTTTTTTTCCTTTTCTTTTTCTTGTGGCTTCCTCAAATCATGGGAGTGAAGCAAATTTTA
TCCAAGCGCACCTTCTCAACCACTCAGGTGAGACAGTATTCGTACTTAATAAGAATCACAAGCAGGTGGGTA
TAGATCTTATTGGGCTATATCTGGATCTCAGTGAACAAACTCTCCCAGTCTCTCAAGCTGGGACTCATTCAC
TCTAGCTCTTCCCTATCCCTTATCCCCCCTACTTCCCTAAGCCATCAACATTTGCCTATTCTACCTCAT↑**AA**
AAGTTTAATATC↓TGCCTCTTATCTTCAACCCATCACTGCCTCATTTCAGACCTTCAGCATTGCTGGACCTC
TGCAGCCTCCACTGATCTCACTGACACCCCCCTTCTCCATT

Filled site:

AAGTGAAGCAATTTTTTTTTCCTTTTCTTTTTCTTGTGGCTTCCTCAAATCATGGGAGTGAAGCAAATTTTA
TCCAAGCGCACCTTCTCAACCACTCAGGTGAGACAGTATTCGTACTTAATAAGAATCACAAGCAGGTGGGTA
TAGATCTTATTGGGCTATATCTGGATCTCAGTGAACAAACTCTCCCAGTCTCTCAAGCTGGGACTCATTCAC
TCTAGCTCTTCCCTATCCCTTATCCCCCCTACTTCCCTAAGCCATCAACATTTGCCTATTCTACCTCAT<u>AAA</u>
<u>AGTTTAATATC</u>**GGCCGGGCGCGGTGGCTCACGCCTGTAATCCCAGCACTTTGGGAGGCCGAGGCGGGCGGAT**
CACGAGGTCAGGAGATCGAGACCATCCTGGCTAACACGGTGAAACCCCGTCTCTACTAAAAAAAAATACAAA
AAATTAGCCGGGCGTGGTAGCGGGCGCCTGTAGTCCCAGCTACTCGGGAGGCTGAGGCAGGGGAATGGCGTG
AACCCGGGAGGCGGAGCTTGCAGTGAGCCGAGATCCCGCCACTGCACTCCAGCCT…[NEOcassette]*AA*
AAAAAAAAAAAAAAAAAACATTACAAAAAAAAAAAAAAAAAAAAAAAAAAAAAAAAAAAA
AAAAAAAAAAAAA<u>AAAA</u><u>GTTTAATATC</u>TGCCTCTTATCTTCAACCCATCACTGCCTCATTTCAGACCTT
CAGCATTGCTGGACCTCTGCAGCCTCCACTGATCTCACTGACACCCCCCTTCTCCATT

No repetitive sequences in the immediate proximity.

CLONE 59

[K30]

Driver: ORF2
Plasmid: AlurescueA70D $A_{17}CATTACA_{18}GA_{17}CACACA_{18}$ (T)
Chromosome: 4
5' position: 121,674,454
Strand: minus
DR: AATAGTATGATTTA
ENDOsite: TATT/AT

Empty site:

AGCTATAAAATAAAAATTTTAGAGAAAACACATAATAATAATATTCTTCCCGGTATAATAATACTGTAGCCT
TCCACTTCAGTTCTGCAGCACGTAAAAGCTATCCTCTGTGGTCCCCTTAAGAAAATACTCTTTTTGTCTTTG
TGGTGAAGAGAGGGAGCTTCTCCACTTATTAAAGCAAATCTATAGCAGGTTTCTCACTCTAGTTTGTAGTAT
CATATTCATTCATATTATATGAATATTAGGAATTGGAGTACTTCCTAGATATAAAAGTACTTCATAAACCAT
AAAGGATTAGACAGATATTCCTGTTTAT↑**AATAGTATGATTTA**↓AATGTACAATATCCAAAATATTTCACAA
TGAGTATTATGAAAGAGGTTTAAATTAGCTTGAGATTAGGAGGTTATTTTACATGTGAGCAGTTGTCACAGA

Filled site:

AGCTATAAAATAAAAATTTTAGAGAAAACACATAATAATAATATTCTTCCCGGTATAATAATACTGTAGCCT
TCCACTTCAGTTCTGCAGCACGTAAAAGCTATCCTCTGTGGTCCCCTTAAGAAAATACTCTTTTTGTCTTTG
TGGTGAAGAGAGGGAGCTTCTCCACTTATTAAAGCAAATCTATAGCAGGTTTCTCACTCTAGTTTGTAGTAT
CATATTCATTCATATTATATGAATATTAGGAATTGGAGTACTTCCTAGATATAAAAGTACTTCATAAACCAT
AAAGGATTAGACAGATATTCCTGTTTATAATAGTATGATTTA**GGCCGGGCGCGGTGGCTCACGCCTGTAATC**
CCAGCACTTTGGGAGGCCGAGGCGGGCGGATCACGAGGTCAGGAGATCGAGACCATCCTGGCTAACACGGTG
AAACCCCGTCTCTACTAAAAAAAAATACAAAAAATTAGCCGGGCGTGGTAGCGGGCGCCTGTAGTCCCAGCT
ACTCGGGAGGCTGAGGCAGGGGAATGGCGTGAACCCGGGAGGCGGAGCTTGCAGTGAGCCGAGATCCCGCCA
CTGCACTCCAGC... [NEOcassette] ***AAAAAAAAAAAAAAAAAAAAAAAAAAAAAAAAAAAAAAAAAAAAA***
AAAAAAAAAAAAAAAAAAAAAAAAAAAAAAAAAAAAAAAAACATTACAAAAAAAAAAAAAAAAAAAAAAAAA
AAAAAAAAAAAAAAAATAGTATGATTTAAATGTACAATATCCAAAATATTTCACAATGAGTATTATGAAAGA
GGTTTAAATTAGCTTGAGATTAGGAGGTTATTTTACATGTGAGCAGTTGTCACAGA

No repetitive sequences in the immediate proximity.

CLONE 60

[JK6, K45]

Driver: ORF2
Plasmid: AlurescueA70D $A_{17}CATTACA_{18}GA_{17}CACACA_{18}$ (T)
Chromosome: 2
5’ position: 228,108,922
Strand: plus
DR: AACAATGAAATA
ENDOsite: TGTT/AA

Empty site:

GTAGTTGTGTCATTCATTTTTATCAGACCTCAGCAGCTGTGAGGCTAATTAGGACTTTTGCACAGACTTTAA
TGTGCACAAGAATCACTAAAGAATCATGTTAAACATGCAGATTCTCGGTCAATAGTCTGGGAGGTGAGGCAG
ATCTAAGATTCTACATTTCTGATAAGCTTCCAGGTAATGCTAATGCTGCAGGCCCCAAACCCCACTTTGAGT
AGCAAGGGTTTGAATGAATTTTTGTCTTTTCTGATAAACAATTATCAAGTTCCAATGAAATAAGCAAGCAAT
TGAATCTTATT↑**AACAATGAAATA**↓ATCAGAAGGGCAAAACAGCTTGGCATGTGACTGAGACTGGGTTTGAT
GTATGGGTTTCTTAGTGCCAAATAATTTTCAGAGTGTTTACTTTTTCTTTTTT

Filled site:

GTAGTTGTGTCATTCATTTTTATCAGACCTCAGCAGCTGTGAGGCTAATTAGGACTTTTGCACAGACTTTAA
TGTGCACAAGAATCACTAAAGAATCATGTTAAACATGCAGATTCTCGGTCAATAGTCTGGGAGGTGAGGCAG
ATCTAAGATTCTACATTTCTGATAAGCTTCCAGGTAATGCTAATGCTGCAGGCCCCAAACCCCACTTTGAGT
AGCAAGGGTTTGAATGAATTTTTGTCTTTTCTGATAAACAATTATCAAGTTCCAATGAAATAAGCAAGCAAT
TGAATCTTATTAACAATGAAATA**GGCCGGGCGCGGTGGCTCACGCCTGTAATCCCAGCACTTTGGGAG**
GCCGAGGCGGGCGGATCACGAGGTCAGGAGATCGAGACCATCCTGGCTAACACGGTGAAACCCCGT
CTCTACTAAAAAAAAATACAAAAAATTAGCCGGGCGTGGTAGCGGGCGCCTGTAGTCCCAGCTACT
CGGGAGGCTGAGGCAGGGGAATGGCGTGAACCCGGGAGGCGGAGCTTGCAGTGAGCCGAGATCCCG
CCACTGCACTCCAGC… [NEOcassette] ***AAAAAAAAAAAAAAAAAAAAAAAAAAAAAAAAAAAAC***
ATTACAAAAAAAAAAAAAAAAAAAAAAAAAAAAAAACAATGAAATAATCAGAAGGGCAAAACAGCTT
GGCATGTGACTGAGACTGGGTTTGATGTATGGGTTTCTTAGTGCCAAATAATTTTCAGAGTGTTTACTTTTT
CTTTTTT

hAT-Charlie DNA element at the 5’.

CLONE 61

[JK30]

Driver: ORF2
Plasmid: AlurescueA70D $A_{17}CATTACA_{18}GA_{17}CACACA_{18}$ (T)
Chromosome: 14
5' position: 50,000,464
Strand: plus
DR: AAAAGCTGAAAGA
ENDOsite: TTTT/AA

Empty site:

CATTCTCCTCCCTCCCTCCACCTCCTACCTCTTCCTCTCACTGGCTGAACCCAATGGGATGCCAGAAAATGG
CCATGGATGCAGTACATTCCATACAACATAGTGTAAATATGTTTTCAAATCGGAGGTTTGCTGATATAACTG
GAAAATCCAAGAAAAAAAATCAGATACAGTACACGTCTCCAAGTGTTTTCAAATAGGGAAATAATCATATTA
AAATGTAGATTCCTGGCTTCTCTT↑**AAAAGCTGAAAGA**↓TCCCCCAGCTGCGATGGCTCATGCCTGTAATCC
CAGCACTTCGGAAGGCCGAGGCGGGTGGATCACTTGAGGTCAGGAGTTTGAGACAAGCCTGGCCAACATGGT
GAAACCCCATCTCTACTAAAAATACAAAAATTAGCTGGGCGTGGTGGCAGGCACCTGTAGTCCCAGCTACTA
GTGGGGCTGACGCAGGATAATCA

Filled site:

CATTCTCCTCCCTCCCTCCACCTCCTACCTCTTCCTCTCACTGGCTGAACCCAATGGGATGCCAGAAAATGG
CCATGGATGCAGTACATTCCATACAACATAGTGTAAATATGTTTTCAAATCGGAGGTTTGCTGATATAACTG
GAAAATCCAAGAAAAAAAATCAGATACAGTACACGTCTCCAAGTGTTTTCAAATAGGGAAATAATCATATTA
AAATGTAGATTCCTGGCTTCTCTTAAAAGCTGAAAGA**GGCCGGGCGCGGTGGCTCACGCCTGTAATCCCAGC**
ACTTTGGGAGGCCGAGGCGGGCGGATCACGAGGTCAGGAGATCGAGACCATCCTGGCTAACACGGTGAAACC
CCGTCTCTACTAAAAAAAAAAATACAAAAAATTAGCCGGGCGTGGTAGCGGGCGCCTGTAGTCCCAGCTACTC
GGGAGGCTGAGGCAGGGGAATGGCGTGAACCCGGGAGGCGGAGCTTGCAGTGAGCCGAGATCCCGCCACTGC
ACTCCAGCCT... [NEOcassette] ***AAAAAAAAAAAAAAAAAAAAAAAAACATTACAAAAAAAAAAAAAAAA***
AAAAAAAAAAAAAAAAAAAAAAAAAAAAAAAAAAAAAAAAAAAAAAAGCTGAAAGATCCCCCAGCTGCGATG
GCTCATGCCTGTAATCCCAGCACTTCGGAAGGCCGAGGCGGGTGGATCACTTGAGGTCAGGAGTTTGAGACA
AGCCTGGCCAAC

LTR at the 5'; hAT-Charlie DNA Element flanks the insertion site; Alu at the 3'.

CLONE 62

[JK11]

Driver: ORF2
Plasmid: AlurescueA70D $A_{17}CATTACA_{18}GA_{17}CACACA_{18}$ (T)
Chromosome: 15
5' position: 33,056,819
Strand: plus
DR: AAAAATGTTAGCGGC
ENDOsite: TTTT/GA

Empty site:

CTCAGACTCTAGAATTGTTAGACAATGCATTTCTGTTTATAAATTGCCCATTCTCAGGTATTCTGTTACAGG
AACACAAAACGTACTAAAACAGATGTTAGTTGGACCATCAGCTTCATAGACTATTTAAAGACTAGGCAAAGA
TGTATACTCATTAGCTAAGCAAGGGAGGGTAAATCTTGGTTAGACCAGAGATGAGATTCTTCCTATGGTGTG
CTGGTATTTTTTCCTGGGTCCTTATGACATCCAGGCCTCTACCCTCACATAGGTGTTGTAATTCAGAACACA
AACCTAATAATGTTTCTC↑**AAAAATGTTAGCGGC**↓CTTTGGATTCTTCTTTTCAAAGAATAGTTCTAGAATA
TTTCTCCATGATAGAGTATTTTGAAGGAGAGAGCGGTCTAATTTGATGAATGAATCCATCAGT

Filled site:

CTCAGACTCTAGAATTGTTAGACAATGCATTTCTGTTTATAAATTGCCCATTCTCAGGTATTCTGTTACAGG
AACACAAAACGTACTAAAACAGATGTTAGTTGGACCATCAGCTTCATAGACTATTTAAAGACTAGGCAAAGA
TGTATACTCATTAGCTAAGCAAGGGAGGGTAAATCTTGGTTAGACCAGAGATGAGATTCTTCCTATGGTGTG
CTGGTATTTTTTCCTGGGTCCTTATGACATCCAGGCCTCTACCCTCACATAGGTGTTGTAATTCAGAACACA
AACCTAATAATGTTTCTCAAAAATGTTAGCGGC**GGCCGGGCGCGGTGGCTCACGCCTGTAATCCCAGCACTT
TGGGAGGCCGAGGCGGGCGGATCACGAGGTCAGGAGATCGAGACCATCCTGGCTAACACGGTGAAACCCCGT
CTCTACTAAAAAAAAATACAAAAAATTAGCCGGGCGTGGTAGCGGGCGCCTGTAGTCCCAGCTACTCGGGAG
GCTGAGGCAGGGGAATGGCGTGAACCCGGGAGGCGGAGCTTGCAGTGAGCCGAGATCCCGCCACTGCACTCC
AGCCT... [NEOcassette]** ***AAAAAAAAAAAAAAAAAAAAAACATTACAAAAA***TGTTAGCGGCCTTTGGA
TTCTTCTTTTCAAAGAATAGTTCTAGAATATTTCTCCATGATAGAGTATTTTGAAGGAGAGAGCGGTCTAAT
TTGATGAATGAATCCATCAGT

LTR at the 5'.

CLONE 63

[K4, K11, K19, K24]

Driver: ORF2
Plasmid: AlurescueA70D $A_{17}CATTACA_{18}GA_{17}CACACA_{18}$ (T)
Chromosome: 16
5’ position: 86,633,892
Strand: minus
DR: TAAAGGAAATGAGG
ENDOsite: TTTA/AT

Empty site:

AGTCAAAGAAGACAAGAAAAGTTCCTATAATCCAAGAACATGCATCTCCAGAATGAAAGGGGTACTCGTGTC
CCAGCACAGGAGAAAACACCACAGCAAACAAGTCAAACATTAACAAAAAAAAAAAAAAAAAAAAAAAAAGAGG
ATCCCTGTTGCTACATCCCCTACTTCCATCATACATTGCTCTCAAGACAACCACTTTAATTCTCTTAGCTCT
CTCTTCTAATATGTGTGTATTACTGTTTCTTGATTCATCAGTTTCAGGACTTAACTACTGACTTTTAT↑**TAA**
AGGAAATGAG↓GATTTTAGTTCATGTATCCACCTCTCTTCCCCACACTCTCCTAATAGAATTATATTGTAAT
TTTCCATAAAATTGGCATTCAGTATTTTCCTTCTTATGA

Filled site:

AGTCAAAGAAGACAAGAAAAGTTCCTATAATCCAAGAACATGCATCTCCAGAATGAAAGGGGTACTCGTGTC
CCAGCACAGGAGAAAACACCACAGCAAACAAGTCAAACATTAACAAAAAAAAAAAAAAAAAAAAAAAAAGAGG
ATCCCTGTTGCTACATCCCCTACTTCCATCATACATTGCTCTCAAGACAACCACTTTAATTCTCTTAGCTCT
CTCTTCTAATATGTGTGTATTACTGTTTCTTGATTCATCAGTTTCAGGACTTAACTACTGACTTTTATTAAA
GGAAATGAG**GGCCGGGCGCGGTGGCTCACGCCTGTAATCCCAGCACTTTGGGAGGCCGAGGCGGGCGGATCA**
CGAGGTCAGGAGATCGAGACCATCCTGGCTAACACGGTGAAACCCCGTCTCTACTAAAAAAAAAAATACAAAA
AATTAGCCGGGCGTGGTAGCGGGCGCCTGTAGTCCCAGCTACTCGGGAGGCTGAGGCAGGGGAATGGCGTGA
ACCCGGGAGGCGGAGCTTGCAGTGAGCCGAGATCCCGCCACTGCACTCCAGC... [NEOcassette]*AAAA*
AAAAAAAAAAAAACATTACAAAAAAAAAAAAAAAAAAAAAAAAAGAAAAAAAAAAAAAAAAAAAAAAAAAAA
AAAAAAAAAAAAAAAAAAAAAAAAAAAAAAAAAAAAAAAACAAAAAATAAAGGAAATGAGGATTTTAGTTCAT
GTATCCACCTCTCTTCCCCACACTCTCCTAATAGAATTATATTGTAATTTTCCATAAAATTGGCATTCAGTA
TTTTCCTTCTTATGA

LINE1 at the 5’ and flanking insertion site.

CLONE 64

[G34, G152, G154, G160, G165, G168, G176, G178, L26]

Driver: ORF2
Plasmid: AlurescueA70Du $A_{17}CATTACA_{18}GA_{17}CACACA_{18}$ (T)
Chromosome: 11
5' position: 130,084,684
Strand: plus
DR: AGAAGTTCGAGACC
ENDOsite: TTCT/AG

Empty site:

GCCAGCACTGTGCTCTATCATATCCATCTATATCACGCATATATACATCTATCTATCACATACATATCAGCA
CTGTGCTCTATCATACACATCCACACCGTGTACTATATATCAGTAGCATACTGTCACCAGCATTTCATAAAG
AAAGGAGATCTTAACATGCTAACTGCAGGTAGGTCATACTGTTGGTATAAAAGACAGTCTTGTATTGCCAGG
CACGGTGGCTCATGCCTGTAATACCAGCACTCTGCGAGGCCGAGGCAGGTGGATTGTTTGAGTCT↑**AGAAGT**
TCGAGACC↓AGACCTGGGCAACATAGACATTTTTACCTGTCCGGTAAAAATGCAAAAAATTAGCCAGGCATG
GTGGCGGGCTCCTGTAATCCCAGCCACTTGGGAGACT

Filled site:

GCCAGCACTGTGCTCTATCATATCCATCTATATCACGCATATATACATCTATCTATCACATACATATCAGCA
CTGTGCTCTATCATACACATCCACACCGTGTACTATATATCAGTAgCATACTGTCACCAGCATTTCATAAAG
AAAGGAGATCTTAACATGCTAACTGCAGGTAGGTCATACTGTTGGTATAAAAGACAGTCTTGTATTGCCAGG
CACGGTGGCTCATGCCTGTAATACCAGCACTCTGCGAGGCCGAGGCAGGTGGATTGTTTGAGTCTAGAAGTT
CGAGACC**GGCCGGGCGCGGTGGCTCACGCCTGTAATCCCAGCACTTTGGGAGGCCGAGGCGGGCGGATCACG**
AGGTCAGGAGATCGAGACCATCCTGGCTAACACGGTGAAACCCCGTCTCTACTAAAAAAAAATACAAAAAAT
TAGCCGGGCGTGGTAGCGGGCGCCTGTAGTCCCAGCTACTCGGGAGGCTGAGGCAGGGGAATGGCGTGAACC
CGGGAGGCGGAGCTTGCAGTGAGCCGAGATCCCGCCACTGCACTCCAGCCT… [NEOcassette]*AAAAA*
AAAAAAAAAAAAAAAAAAAAAAAAAAAAAAAAAAAAACATTACAAAAAAAAAAAAAAAAAAAAAAAAAAA
AAAAAAAAAAAAAAAAAAAAAAAAAAAAAAAAAAAAAAAAAAAAAAAAAAAGAAGTTCGAGACCAGACC
TGGGCAACATAGACATTTTTACCTGTCCGGTAAAAATGCAAAAAATTAGCCAGGCATGGTGGCGGGCTCCTG
TAATCCCAGCCACTTGGGAGACT

hAT-Charlie DNA element at the 5; Alu flanking insertion site.

CLONE 65

[L35, L36]

Driver: L1
Plasmid: AlurescueA70D $A_{17}CATTACA_{18}GA_{17}CACACA_{18}$ (T)
Chromosome: X
5' position: 115,456,032
Strand: minus
DR: GAAAAGGTGATATTT
ENDOsite: TTTC/AA

Empty site:

TGTACAGTAAATGCTTACCAAGGCCCTAAATCTTGACAGATCTTGAGCCTCTGGCTGTGTCTATGCTTGCCA
AATCTCAATATTTGAAAATATCGAAGTATGCAAATATTTTCCAATATTCCCTCAAATGCTTAGTGTAGATAC
AGAACTCGTACTTTTACTGAGCACCAGAACCACAGGCTGAGTCAGACTGGGAATACAAAAGAAATT↑**GAAAA
GGTGATATTT**↓GAGGAACTCAAAAGGTACCACACTGAACCTAGGCCGATGCTCTGCTTTCCATCCCTACATG
ACTGCCTCATCATAAGTAGCATCCCACAGCTGCTCTTCT

Filled site:

TGTACAGTAAATGCTTACCAAGGCCCTAAATCTTGACAGATCTTGAGCCTCTGGCTGTGTCTATGCTTGCCA
AATCTCAATATTTGAAAATATCGAAGTATGCAAATATTTTCCAATATTCCCTCAAATGCTTAGTGTAGATAC
AGAACTCGTACTTTTACTGAGCACCAGAACCACAGGCTGAGTCAGACTGGGAATACAAAAGAAATTGAAAAG
GTGATATTT**GGCCGGGCGCGGTGGCTCACGCCTGTAATCCCAGCACTTTGGGAGGCCGAGGCGGGCGGATCA
CGAGGTCAGGAGATCGAGACCATCCTGGCTAACACGGTGAAACCCCGTCTCTACTAAAAAAAAATACAAAAA
ATTAGCCGGGCGTGGTAGCGGGCGCCTGTAGTCCCAGCTACTCGGGAGGCTGAGGCAGGGGAATGGCGTGAA
CCCGGGAGGCGGAGCTTGCAGTGAGCCGAGATCCCGCC... [NEOcassette]** ***AAAAAAAAAAAAAAAAAA
AAAACATTACAAAAAAAAAAAAAAAAAAAAAAAAAAAAAAAAAAAAAAAAAGAAAAAAAAAAAAAAAAA***GAAA
AGGTGATATTTGAGGAACTCAAAAGGTACCACACTGAACCTAGGCCGATGCTCTGCTTTCCATCCCTACATG
ACTGCCTCATCATAAGTAGCATCCCACAGCTGCTCTTCT

No repetitive sequences in the immediate proximity.

CLONE 67

[M34]

Driver: L1
Plasmid: AlurescueA70D $A_{17}CATTACA_{18}GA_{17}CACACA_{18}$ (T)
Chromosome: 13
5' position: 89,566,510
Strand: minus
DR: AAAAAATTTCT
ENDOsite: TTTT/AA

Empty site:

TGTTGAAGAGACTATTCTATTTCCATTGTAAGTTATTAGTGCCCTTGTCAAAGATTAGTTAACCATATATAC
ATGAGTTTATTTCTGGGCTTTTTATTCTGTTTCATTGGTCTATATGTCTGTTTTCATGCCAGACTGTACTGT
TTTGGTTACTATAGCTTTGTAATGTAATTAGAAATCAAGAAGTATAATTCCTCCAGCTTTGTTATTTTTTAA
CAGATGGCTTTAGGTATTTGAGATATTTTGTGGATCCATATAAATTTCATGGTTTT↑**AAAAAATTTCT**↓AAT
GAAAAATGCCTTTGAAATTTTAATAGGGGTTGCATTGGATCTGTAGTTTGCTTTGTGTAGTACGGACATTTT
ACCAATATTGACTCTTCCAATCTAA

Filled site:

TGTTGAAGAGACTATTCTATTTCCATTGTAAGTTATTAGTGCCCTTGTCAAAGATTAGTTAACCATATATAC
ATGAGTTTATTTCTGGGCTTTTTATTCTGTTTCATTGGTCTATATGTCTGTTTTCATGCCAGACTGTACTGT
TTTGGTTACTATAGCTTTGTAATGTAATTAGAAATCAAGAAGTATAATTCCTCCAGCTTTGTTATTTTTTAA
CAGATGGCTTTAGGTATTTGAGATATTTTGTGGATCCATATAAATTTCATGGTTTTAAAAAATTTCT**GGCCG**
GGCGCGGTGGCTCACGCCTGTAATCCCAGCACTTTGGGAGGCCGAGGCGGGCGGATCACGAGGTCAGGAGAT
CGAGACCATCCTGGCTAACACGGTGAAACCCCGTCTCTACTAAAAAAAAATACAAAAAATTAGCCGGGCGTG
GTAGCGGGCGCCTGTAGTCCCAGCTACTCGGGAGGCTGAGGCAGGGGAATGGCGTGAACCCGGGAGGCGGAG
CTTGCAGTGAGCCGAGATCCCGCCACTGCACTCCAGC… [NEOcassette] ***AAAAAAAAAAAAAAAAAA***
AACATTACAAAAAAAAAAAAAAAAAAAAAAAAAAAAAAAAGAAAAAAAAAAAAAAAAAAAAAAAAAA
AAAAAAAAAAAAAAATTTCTAATGAAAAATGCCTTTGAAATTTTAATAGGGGTTGCATTGGATCTGTAGTT
TGCTTTGTGTAGTACGGACATTTTACCAATATTGACTCTTCCAATCTAA

LINE1 flanking the insertion site.

CLONE 68

[M8]

Driver: L1
Plasmid: AlurescueA70D $A_{17}CATTACA_{18}GA_{17}CACACA_{18}$ (T)
Chromosome: 5
5' position: 36,073,451
Strand: minus
DR: CAAAAGAAGGTTGA
ENDOsite: TTTG/GA

Empty site:

CAATTCAAAAAGTCAGAATGTTTCATTATCTCCAAAGAATTGCACTAGCTCCCTAGCAATGAATCCTAACCA
GATTGAAATGTCTGAAAAGATAGACACAGAATTCAGAATCTGGATAGGAAAGTTCAACAAGATC↑**CAAAAGA**
AGGTTGA↓AATTCAATCCATGGAAGCCAGAAAACTGACCCAATATATGAAAGACGTCATAGCTAGAAGAAAG
AACAAAATTGAACTTCTGGAATAGAGAAAACCACTA

Filled site:

CAATTCAAAAAGTCAGAATGTTTCATTATCTCCAAAGAATTGCACTAGCTCCCTAGCAATGAATCCTAACCA
GATTGAAATGTCTGAAAAGATAGACACAGAATTCAGAATCTGGATAGGAAAGTTCAACAAGATCCAAAAGAA
GGTTGA**GGCCGGGCGCGGTGGCTCACGCCTGTAATCCCAGCACTTTGGGAGGCCGAGGCGGGCGGATCACGA**
GGTCAGGAGATCGAGACCATCCTGGCTAACACGGTGAAACCCCGTCTCTACTAAAAAAAAATACAAAAAATT
AGCCGGGCGTGGTGGCGGGCGCCTGTGGTCCCGGCTACTCGGGAGGCTGAGGCAGGGGAATGGCGTGAACCC
GGGAGGCGGAGCTTGCAGTGAGCCGAGATCCCGCCACTGCACTCCAGC…[NEOcassette]*AAAAAAAA*
AAAAAAAAACATTACAAAAAAAAAAAAAAAAAAAAAAAAAAAAAAGAAAAAAAAAAAAAAAAAAAAAAAAAA
AAAAAAAAAAAAAAAAAAAAAAAAAAAAAAAAAAAAAAAAAAAAAAAAAAAAAAAAACAAAAGAAGGTTGAA
ATTCAATCCATGGAAGCCAGAAAACTGACCCAATATATGAAAGACGTCATAGCTAGAAGAAAGAACAAAATT
GAACTTCTGGAATAGAGAAAACCACTA

LINE1 flanking the insertion site.

CLONE 72

[E10, E43, F25, N6]

Driver: ORF2
Plasmid: AlurescueA70Du $A_{17}CATTACA_{18}GA_{17}CACACA_{18}$ (T)
Chromosome: 16
5' position: 14,536,134
Strand: plus
DR: AAGAACAAGTATGTG
ENDOsite: TCTT/GT

Empty site:

TTTGAACAATGTCTCTATTTACAGGACAAAAAATAACCGGGAATAGTCAATGTATCTCCTTTGGGCATGCCT
CTGTAAACACCATCTAACTAAGGTGCTGATGGAACCTGCTGCTAGGGACCAGCCAGCAGTATTTTCACAATG
GTCAGCCCAGGCAGCATCAGACCATCCATGCGGCCCTGAGATTCTCCAGAGAAGTGAGGGGGTGGGCAATTT
CTCCAGGTCCCAGGAACACATCAAC↑**AAGAACAAGTATGTG**↓ACAGAAATCCTTCAATTAAACATGACCTGG
GAAAACAGCCACACTTCCTGAAGACATTTTATTCCAGCATTAATGGCATGCAGAGTAGTTTTCCTTTGCG

Filled site:

TTTGAACAATGTCTCTATTTACAGGACAAAAAATAACCGGGAATAGTCAATGTATCTCCTTTGGGCATGCCT
CTGTAAACACCATCTAACTAAGGTGCTGATGGAACCTGCTGCTAGGGACCAGCCAGCAGTATTTTCACAATG
GTCAGCCCAGGCAGCATCAGACCATCCATGCGGCCCTGAGATTCTCCAGAGAAGTGAGGGGGTGGGCAATTT
CTCCAGGTCCCAGGAACACATCAAC<u>AAGAACAAGTATGTG</u>**GGCCGGGCGCGGTGGCTCACGCCTGTAATCCC**
AGCACTTTGGGAGGCCGAGGCGGGCGGATCACGAGGTCAGGAGATCGAGACCATCCTGGCTAACACGGTGAA
ACCCCGTCTCTACTAAAAAAAAAATACAAAAAATTAGCCGGGCGTGGTAGCGGGCGCCTGTAGTCCCAGCTAC
TCGGGAGGCTGAGGCAGGGGAATGGCGTGAACCCGGGAGGCGGAGCTTGCAGTGAGCCGAGATCCCGCCACT
GCACTCCA… [NEOcassette] ***AAAAAAAAAAAAAAAAAAACATTACAAAAAAAAAAAAAAAAAAAAAAAA***
GAAAAAAAAAAAAAAAAAAAAAAAAAAAAAAAAAAAAAAAAAAAAAAAACACACAAAAAAAAAAAAAAAAAA
AAACAAAAAAAAAAAAAAAAAAAAAAAAAAAAAAAAAAAAAAAAA<u>***A***GAACAAGTATGTG</u>ACAGAAATCCTTC
AATTAAACATGACCTGGGAAAACAGCCACACTTCCTGAAGACATTTTATTCCAGCATTAATGGCATGCAGAG
TAGTTTTCCTTTGCG

No repetitive sequences in the immediate proximity.

CLONE 73

[H1, H12, H15, H16, H17, H21, H39, H60]

Driver: L1
Plasmid: AlurescueA70Du $A_{17}CATTACA_{18}GA_{17}CACACA_{18}$ (T)
Chromosome: 8
5' position: 81,155,536
Strand: plus
DR: AGAAATTCAGATGGTAG
ENDOsite: TTCT/AT

Empty site:

ACAGCCTTGAAATCAAGATTCCCAACTTATTTTCTATTCCATTTAAAAACGATTTGATTCTTTCCCTGGCTG
TCTTGGGTCACACTGATCTGATTTCACCCCTGTCTGTGTCACCTCATAATTTATGATAAGAAGGAAACTAGT
AGAAGTGTTTCCATTTTTAACAGGCTGGGGACAACTATTTGCCTGAGCTGAAATTAACATCACTTCATTAGA
AATACAGTTTTAAAATCACAGTGAAAGGCAAGAGCAAAGACAAGATTTTAAAAATATGCCTTCCCTAT↑**AGA**
AATTCAGATGGTAG↓GTTGGGCGCCGTGGCTCACACCTGTAATCCCAGCACTTTGGGAAGCCGAAGTAGGCA
GATCACAAGGTCAGGAGTTCGAGACCAGTCTGGCCAACATGGT

Filled site:

ACAGCCTTGAAATCAAGATTCCCAACTTATTTTCTATTCCATTTAAAAACGATTTGATTCTTTCCCTGGCTG
TCTTGGGTCACACTGATCTGATTTCACCCCTGTCTGTGTCACCTCATAATTTATGATAAGAAGGAAACTAGT
AGAAGTGTTTCCATTTTTAACAGGCTGGGGACAACTATTTGCCTGAGCTGAAATTAACATCACTTCATTAGA
AATACAGTTTTAAAATCACAGTGAAAGGCAAGAGCAAAGACAAGATTTTAAAAATATGCCTTCCCTATAGAA
ATTCAGATGGTAG**GGCCGGGCGCGGTGGCTCACGCCTGTAATCCCAGCACTTTGGGAGGCCGAGGCGGGCGG**
ATCACGAGGTCAGGAGATCGAGACCATCCTGGCTAACACGGTGAAACCCCGTCTCTACTAAAAAAAAAAAAA
ATACAAAAAATTAGCCGGGCGTGGTAGCGGGCGCCTGTAGTCCCAGCTACTCGGGAGGCTGAGGCAGGGGAA
TGGCGTGAACCCGGGAGGCGGAGCTTGCAGTGAGCCGAGATCCCGCCACTGCACTCCAGCCTG… **[NEOcas**
sette] ***AAAAAAAAAAAAAAAAAAACATTACA***GAAATTCAGATGGTAGGTTGGGCGCCGTGGCTCACAC
CTGTAATCCCAGCACTTTGGGAAGCCGAAGTAGGCAGATCACAAGGTCAGGAGTTCGAGACCAGTCTGGCCA
ACATGGT

Alu at the 3'.

CLONE 74

[H7, H31, H40, H49, H52, H57]

Driver: L1
Plasmid: AlurescueA70Du $A_{17}CATTACA_{18}GA_{17}CACACA_{18}$ (T)
Chromosome: 11
5' position: 86,515,625
Strand: minus
DR: AAGAAAAAAACAAACTGC
ENDOsite: TCTT/AT

Empty site:

ATTTTCAATGTGCATGAAGAACATGGCAGTTACAATATCCTCTTTCATGAGCCACAGCCTTACTCCAACCTC
TCAATATAGAGGATCAAATGTATTCTTTGGCAAAATGATGAATGATTATGTCTCAAGAAACTTTGGGATCAG
TTATGAATCACAGAATCCTGTTAGAGAATGCTAAAGAGTCTACTGCATGATAGAATATTACTAATTCAGAGT
TCTTACAACTTGAATTCTGTTCATTTAGCCAGGAGTGAAGGAGAAGCCAGCCATCTAT↑**AAGAAAAAAAACAA**
ACTGC↓TAACCCAACAGGACAGTGTGAAGCTTTAACTTTGAAGAATAAATTTTTCAGAGACTTAATAAGTCC
TAATTTCTATTAGTTTGCAACTCTGCTTCCAAGT

Filled site:

ATTTTCAATGTGCATGAAGAACATGGCAGTTACAATATCCTCTTTCATGAGCCACAGCCTTACTCCAACCTC
TCAATATAGAGGATCAAATGTATTCTTTGGCAAAATGATGAATGATTATGTCTCAAGAAACTTTGGGATCAG
TTATGAATCACAGAATCCTGTTAGAGAATGCTAAAGAGTCTACTGCATGATAGAATATTACTAATTCAGAGT
TCTTACAACTTGAATTCTGTTCATTTAGCCAGGAGTGAAGGAGAAGCCAGCCATCTATAAGAAAAAAAACAAA
CTGC**AGGCCGGGCGCGGTGGCTCACGCCTGTAATCCCAGCACTTTGGGAGGCCGAGGCGGGCGGATCACGAG**
GTCAGGAGATCGAGACCATCCTGGCTAACACGGTGAAACCCCGTCTCTACTAAAAAAAAATACAAAAAATTA
GCCGGGCGTGGTAGCGGGCGCCTGTAGTCCCAGCTACTCGGGAGGCTGAGGCAGGGGAATGGCGTGAACCCG
GGAGGCGGAGCTTGCAGTGAGCCGAGATCCCGCCACTGCACTCCAGC... [NEOcassette] ***AAAAAAAAA***
AAAAAAAAAAAAAAAAAAAAAAAAAAAAAAAAAAAAAAAAAAAAAAAAAAAAAAAAAAAAAAAAAAAAAAAA
AAAAAAAAAAAAGAAAAAAAACAAACTGCTAACCCAACAGGACAGTGTGAAGCTTTAACTTTGAAGAATAAA
TTTTTCAGAGACTTAATAAGTCCTAATTTCTATTAGTTTGCAACTCTGCTTCCAAGT

No repetitive sequences in the immediate proximity.

An extra A added immediately 5' of Alu.

CLONE 76

[G150]

Driver: ORF2
Plasmid: AlurescueA70Du $A_{17}CATTACA_{18}GA_{17}CACACA_{18}$ (T)
Chromosome: 9
5' position: 97,694,639
Strand: plus
DR: none
ENDOsite: unknown

Empty site:

CCTGTCTTCAGGGAACTGAGAGCATTTAGTTGCATTAAAACAAATAAAAAAACAAACAAACAAAAAACCTGC
AGGTATCTTGGGCTCTGGAAGGACATACAGGTCAGGGTGAAAAGTATGCTTGGTCCAAGCATCTTTTTTCTT
TTAACACATGAAGTATGTGTCTTATTATTTTAAAAGACATTGAGAAAAAGGAACATTCAACATTCTCTTGGT
TATAGATACTGGAGTATTAACAAAGTGAAAAGAGAAAAACTAAAATTTTGTTTGGCTCTGACTTACTAGTTT
TAGTAATTTATAAGAACAGCTTTCCATTTGTGCCTTTCATACATCCACTGAGTCATGAGGTTTCATCAAATG
T

Filled site:

CCTGTCTTCAGGGAACTGAGAGCATTTAGTTGCATTAAAACAAATAAAAAAACAAACAAACAAAAAACCTGC
AGGTATCTTGGGCTCTGGAAGGACATACAGGTCAGGGTGAAAAGTATGCTTGGTCCAAGCATCTTTTTTCTT
TTAACACATGAAGTATGTGTCTTATTATTTTAAAAGACATTGAGAAAAAGGAACATTCAACATTCTCTTGGT
TATAGATACTGGAGTATTAACAAAGTGAAAAGAGAAAA**ACTAAAAAAAAATACAAAAAATTAGCCGGGCGTG**
GTAGCGGGCGCCTGTAGTCCCAGCTACTCGGGAGGCTGAGGCAGGGGAATGGCGTGAACCCGGGAGGCGGAG
CTTGCAGTGAGCCGAGATCCCGCCACTGCACTCCAGCCTGGGCGACAGAGCGAG…[NEOcassette]*AAA*
AAAAAAAAAAAAAAAAAAAAAACATTACAAAAAAAAAAAAAAAAAAAAAAAAAAAAAAAAGAAAAAAAA
AAAAAAAAAAAAAACACACAAAAAAAAAAAAAAAAAAAAAAAAAAAAAAAAAAAAAAAAAAAAAAAAA
***AAAAAAAAAAAAAAAAAAAA*NGGGGGNNTTNNN…[unknown 3' sequence]**

No repetitive sequences in the immediate proximity.

Alu insert truncation missing between 115-122 bp of the 5' sequence.

These seven nucleotides are identical between the Alu vector and insertion sequence.

We were unable to sequence past the A-tail and primers designed from the 3' genomic sequence did not work on the recovered insert plasmid.

CLONE 77

[G50,G59,G146,G147,G148,G149, G185]

Driver: ORF2
Plasmid: AlurescueA70Du   A$_{17}$CATTACA$_{18}$GA$_{17}$CACACA$_{18}$ (T)
Chromosome: 11
5’ position: 75,775,172
Strand: plus
DR: TAGGAATGTT
ENDOsite: CCTA/AA

Empty site:

CCCTTTAAAATGGGTATTTTTCAAGATTGATAGATCTATAAATACATGTACTTTTGTAAAGTAAGATGAATG
CATGATAGTTATAAGCTATTATACTATTCAACTTCAAGTGACCTAAGAATTTGAAAGCAATATAAAATGTAA
ATTCTGTCCTAATATTCGATCTGTCTTTGCATTGCCTTATCCGAACCATACCTTGTGGCTTTTTGTATAAAT
AAGTCCCACTGAAGTCATAATAAAGTCAAATGTTGTGAAATCAATTTTT↑**TAGGAATGTT**↓GAAGCTTGGTT
TTCAATGTATGCTGATCTCAATTTACTCTTCTAACATACGAAACAGTTTCTAGATTTATACCTTGAAAATAG
AGATAGCTATGGGTTTG

Filled site:

CCCTTTAAAATGGGTATTTTTCAAGATTGATAGATCTATAAATACATGTACTTTTGTAAAGTAAGATGAATG
CATGATAGTTATAAGCTATTATACTATTCAACTTCAAGTGACCTAAGAATTTGAAAGCAATATAAAATGTAA
ATTCTGTCCTAATATTCGATCTGTCTTTGCATTGCCTTATCCGAACCATACCTTGTGGCTTTTTGTATAAAT
AAGTCCCACTGAAGTCATAATAAAGTCAAATGTTGTGAAATCAATTTTTTAGGAATGTT**GGCCGGGCGCGGT**
GGCTCACGCCTGTAATCCCAGCACTTTGGGAGGCCGAGGCGGGCGGATCACGAGGTCAGGAGATCGAGACCA
TCCTGGCTAACACGGTGAAACCCCGTCTCTACTAAAAAAAAATACAAAAAATTAGCCGGGCGTGGTAGCGGG
CGCCTGTAGTCCCAGCTACTCGGGAGGCTGAGGCAGGGGAATGGCGTGAACCCGGGAGGCGGAGCTTGCAGT
GAGCCGAGATCCCGCCACTGCACTCCAGCCTG... [NEOcassette] ***AAAAAAAAAAAAAAAAAAAAAAACA***
TTACAAAAAAAAAAAAAAAAAAAAAAAAAAAAGAAAAAAAAAAAAAAAAAAAAAAAAAAAAAAAAAAAAAAAG
AAAAAAAAAAAAAAAAAAAAAAAAATAAAAATAGGAATGTTGAAGCTTGGTTTTCAATGTATGCTGATCTCA
ATTTACTCTTCTAACATACGAAACAGTTTCTAGATTTATACCTTGAAAATAGAGATAGCTATGGGTTTG

No repetitive sequences in the immediate proximity.

CLONE 78

[E4, F39]

Driver: ORF2
Plasmid: AlurescueA70Du $A_{17}CATTACA_{18}GA_{17}CACACA_{18}$ (T)
Chromosome: 9
5' position: 117,610,791
Strand: minus
DR: AGAATCTG
ENDOsite: TTCT/AA

Empty site:

TCTAAAAGAAACTCAAATATATCATGCTTCCTCAAATTTGCTGGAGAAATATTTATAAGTTTACATTCATTA
ATAAAGTGCTGTGAAATTTCTTCAGTCTCTGTATTTACTCAAACCCTTAGAGTTTCTTCATCTTGCTTAAAT
TTTTTCCTGGATGAAGGCATGCTGCAGAGCCTGGTTGATGCTAATTCATTTAGCTAGGCCCAACATT↑**AGAA
TCTG**↓GTCCAACAAGTCCTTTAGCTAGTATACTTTTTTACACTGGTCTTCAGGAAGTGTCTGGCACCCAATC
AAGTTGGCCAATAGGTCCTTAGTTGGATTAATT

Filled site:

TCTAAAAGAAACTCAAATATATCATGCTTCCTCAAATTTGCTGGAGAAATATTTATAAGTTTACATTCATTA
ATAAAGTGCTGTGAAATTTCTTCAGTCTCTGTATTTACTCAAACCCTTAGAGTTTCTTCATCTTGCTTAAAT
TTTTTCCTGGATGAAGGCATGCTGCAGAGCCTGGTTGATGCTAATTCATTTAGCTAGGCCCAACATTAGAAT
CTG**GGCCGGGCGCGGTGGCTCACGCCTGTAATCCCAGCACTTTGGGAGGCCGAGGCGGGCGGATCACGAGGT
CAGGAGATCGAGACCATCCTGGCTAACACGGTGAAACCCCGTCTCTACTAAAAAAAAATACAAAAAATTAGC
CGGGCGTGGTAGCGGGCGCCTGTAGTCCCAGCTACTCGGGAGGCTGAGGCAGGGGAATGGCGTGAACCCGGG
AGGCGGAGCTTGCAGTGAGCCGAGATCCCGCCACTGCACTCCAGCCTG... [NEOcassette]** ***AAAAAAAA
AAAAAAAAAAAAAAAAAAAACATTACAAAAAAAAAAAAAAAAAAAAGAAAAAAAAAAAAA
AAAAAAAAAAAAAAAAAAACACACAAAAAAAAAAAAAAAAAAAAAAA***GAATCTGGTCCAA
CAAGTCCTTTAGCTAGTATACTTTTTTACACTGGTCTTCAGGAAGTGTCTGGCACCCAATCAAGTTGGCCAA
TAGGTCCTTAGTTGGATTAATT

No repetitive sequences in the immediate proximity.

CLONE 79

[F1, F2, F5, F12,F15]

Driver: ORF2
Plasmid: AlurescueA70Du $A_{17}CATTACA_{18}GA_{17}CACACA_{18}$ (T)
Chromosome: 11
5' position: 64,887,860
Strand: plus
DR: AGAAGATGAAAAAG
ENDOsite: TTCT/AA

Empty site:

CTTTTGGGAACACTGAAACCTCACTATATCCAATCACAGATGTGATTAGGTTACCAAGTCCGGCCAGGCAGT
CACAGAGGTACAAACGAGGCCATGCTCCCAACATAATATTTACTTCTAAAACCAAGGCAGACCAGATTCCTG
CCCTGGACTCTAAGGCCACAGCTGGTACCAAGTTCAGGGAACGGCCAGGTTCACCTTCTATACAAACCTCTT
CCCCCACCCATCCCTTCTATTGCTGCCTCCATAACAAACCTCTCACCACAGTGATTCCTGCTACCTTCTCTG
TTT↑**AGAAGATGAAAAAG**↓AATGACCTGGGGGGACTGATCAAGTTACTTAGGATAGGGAATAACCCCAATTT
TACATCTGAAGCCCCCCAGGATCTGTAGCTGTTTCTTCACAGGAAAT

Filled site:

CTTTTGGGAACACTGAAACCTCACTATATCCAATCACAGATGTGATTAGGTTACCAAGTCCGGCCAGGCAGT
CACAGAGGTACAAACGAGGCCATGCTCCCAACATAATATTTACTTCTAAAACCAAGGCAGACCAGATTCCTG
CCCTGGACTCTAAGGCCACAGCTGGTACCAAGTTCAGGGAACGGCCAGGTTCACCTTCTATACAAACCTCTT
CCCCCACCCATCCCTTCTATTGCTGCCTCCATAACAAACCTCTCACCACAGTGATTCCTGCTACCTTCTCTG
TTT<u>AGAAGATGAAAAAG</u>**GGCCGGGCGCGGTGGCTCACGCCTGTAATCCCAGCACTTTGGGAGGCCGAGGCGG**
GCGGATCACGAGGTCAGGAGATCGAGACCATCCTGGCTAACACGGTGAAACCCCGTCTCTACTAAAAAAAAA
TACAAAAAATTAGCCGGGCGTGGTAGCGGGCGCCTGTAGTCCCAGCTACTCGGGAGGCTGAGGCAGGGGAAT
GGCGTGAACCCGGGAGGCGGAGCTTGCAGTGAGCCGAGATCCCGCCACTGCACTCCAGC… [NEOcassett
e] ***AAAAAAAAAAAAAAAAAAACATTACAAAAAAAAAAAAAAAAAAAAAAAAAAAAAAAAA***
AAAAAAAAAAAAAAAAAAAAAA<u>A</u><u>GAAGATGAAAAAG</u>AATGACCTGGGGGGACTGATCAAGTTACTTAG
GATAGGGAATAACCCCAATTTTACATCTGAAGCCCCCCAGGATCTGTAGCTGTTTCTTCACAGGAAAT

No repetitive sequences in the immediate proximity.

CLONE 80

[D16, D17, D35, D42, D123, D137, D174, E11]

Driver: ORF2
Plasmid: AlurescueA70Du $A_{17}CATTACA_{18}GA_{17}CACACA_{18}$ (T)
Chromosome: 12
5' position: 46,713,555
Strand: plus
DR: AAAATTTATGTATTA
ENDOsite: TTTT/AA

Empty site:

TGTGGTATATCCATATAATTAAATATATTTATATATAAATATGTGCATATTTATATTGAGTATATTAAACTC
TGTGAATATACTAAAATCCACAGAATTGTACACTTTATAAGTATGAATTTTTATGGTATATGAATTATATCT
AAATGAAGCTGTTAAAAAGAAAAAGAAAAACACAACTGGCTTCTGGCAGAACAATTCTAATTTTTTATCATA
TAGATATTTAATTTCACCATAGAAGAGTTATAGCATTTTAATAAATGATCCTGAGGTTATTTATT↑**AAAATT**
TATGTATTA↓AAATAACCCTGGGGTTATTTATTAAAATTGCATAAGGCTGAGGCAGGAGAATGGAGTGAACC
CGGGAGGCGGAGCTTATAGTGAGCTGATTTCGCCTCAC

Filled site:

TGTGGTATATCCATATAATTAAATATATTTATATATAAATATGTGCATATTTATATTGAGTATATTAAACTC
TGTGAATATACTAAAATCCACAGAATTGTACACTTTATAAGTATGAATTTTTATGGTATATGAATTATATCT
AAATGAAGCTGTTAAAAAGAAAAAGAAAAACACAACTGGCTTCTGGCAGAACAATTCTAATTTTTTATCATA
TAGATATTTAATTTCACCATAGAAGAGTTATAGCATTTTAATAAATGATCCTGAGGTTATTTATTAAAATTT
ATGTATTA**ACAAAAAAATTAGCCGGGCGTGGTAGCGGGCGCCTGTAGTCCCAGCTACTCGGGAGGCTGAGGCA**
GGGGAATGGCGTGAACCCGGGAGGCGGAGCTTGCAGTGAGCCGAGATCCCGCCACTGC…[NEOcassette
]*AAAAAAAAAAAAAAAAAAAAACATTACAAAAAAAAAAAAAAAAAAAAAAAAAAAAAAAAAAAAAAAA*
AAAAAAAAAAAAAAAAAAAAAAGAAAAAAATTTATGTATTAAAATAACCCTGGGGTTATTTATTAAAATT
GCATAAGGCTGAGGCAGGAGAATGGAGTGAACCCGGGAGGCGGAGCTTATAGTGAGCTGATTTCGCCTCAC

L1 at the 5'; Alu at the 3'.

Alu insert truncation missing 129 bp of the 5' sequence.

CLONE 81

[Brown Alu rescue MP#52, 56, 72, C47, E12, colored 6]

Driver: ORF2
Plasmid: AlurescueA70Du $A_{17}CATTACA_{18}GA_{17}CACACA_{18}$ (T)
Chromosome: 16
5' position: 66,895,293
Strand: plus
DR: AAGACATGGTT
ENDOsite: TCTT/AC

Empty site:

AGAAGGAAGGAAGGAAGCAAGGAAGGAAGGAAGGAAGGACGGACGGAAGGAAGGACGGACAGAAGGAA
GGAGAAAGAAAAAAAGAAAGAAGGAAGGAAGGAAGGAAGGAAAGAAAGAAAGAAAGGAAAGAAAGAAAGAAA
GAAAGAAAGAAAGAAAGAAAGAAAGAAAGAAAGAAAGAGAAAGAAAGAAGAACTCCCCGCTCCAAATAACTC
CCTGGCAGCTTGTCATAGTGCCTGAAATCCTGCCATTGGATGTGCACTGTGCACAGGT↑**AAGACATGGTT**↓G
CCTGATGAATGCAAGTGCCGCTGCGAAGGCCACAGTCAGTAACACCTTGTTGGGATGAAAAGGAGGATGTTA
TCGCCTCCTTTTCAGTCTGCTGTGTGA

Filled site:

AGAAGGAAGGAAGGAAGCAAGGAAGGAAGGAAGGAAGGACGGACGGAAGGAAGGACGGACAGAAGGAA
GGAGAAAGAAAAAAAGAAAGAAGGAAGGAAGGAAGGAAGGAAAGAAAGAAAGAAAGGAAAGAAAGAAAGAAA
GAAAGAAAGAAAGAAAGAAAGAAAGAAAGAAAGAAAGAGAAAGAAAGAAGAACTCCCCGCTCCAAATAACTC
CCTGGCAGCTTGTCATAGTGCCTGAAATCCTGCCATTGGATGTGCACTGTGCACAGGTAAGACATGGTT**GGC**
CGGGCGCGGTGGCTCACGCCTGTAATCCCAGCACTTTGGGAGGCCGAGGCGGGCGGATCACGAGGTCAGGAG
ATCGAGACCATCCCGGCTAAAACGGTGAAACCCCGTCTCTACTAAAAATACAAAAAATTAGCCGGGCGTAGT
GGCGGGCGCCTGTAGTCCCAGCTACTTGGGAGGCTGAGGCAGGAGAATGGCGTGAACCCGGGAGGCGGAGCT
TGCAGTGAGCCGAGATCCCGCCACTGCACTCCAG... [NEOcassette] ***AAAAAAAAAAAAAAAAAACATT***
ACAAAAAAAAAAAAAAAAAAAAAAAAAAAAAAAAAAAAAAAAAAAAAAAAAAAAAAAAAAAAAAAAAAAAAAAA
AAAAAAAAAAAAAAAAAAAAAAAAAAAAAAAAAAAAAAAAAAAAAGACATGGTTGCCTGATGAATGCAAGTG
CCGCTGCGAAGGCCACAGTCAGTAACACCTTGTTGGGATGAAAAGGAGGATGTTATCGCCTCCTTTTCAGTC
TGCTGTGTGA

No repetitive sequences in the immediate proximity.

CLONE 82

[C40, C49, E7]

Driver: ORF2
Plasmid: AlurescueA70Du $A_{17}CATTACA_{18}GA_{17}CACACA_{18}$ (T)
Chromosome: 10
5’ position: 94,664,520
Strand: minus
DR: AAAACTTCCTACTCT
ENDOsite: TTTT/AG

Empty site:

ATAAATGCTGCTGGAACAACTGGACATTCACATGCAAAAAAGTGAATAAATAAATGTAAACAGTGACCTTCA
CTTTTTATAAAAATTAATTCAAAATAGATCATAGACCTAAATGTAAAACACAAAACTCTAAGACTCTAGAAG
ATAACACAAGGGAAAATTTAGATGACCTTGGGTTTGGCAATTAGTTTTTAGAAACACCAAAGCACAATGCAT
GAGGGAAAGAAAAAAAGGTAAACTGGACTTTTCCAATTTAATTTCATTAAAACT↑**AAAACTTCCTACTCT**↓T
TAAAAACACTGTTAAGAAAATGAAAAAACAAGCCACAGACTGGGAGAAAATATGTTCGAAACACAAATTGAA
TCAAGGACTTGAATCCAAAATATAAAA

Filled site:

ATAAATGCTGCTGGAACAACTGGACATTCACATGCAAAAAAGTGAATAAATAAATGTAAACAGTGACCTTCA
CTTTTTATAAAAATTAATTCAAAATAGATCATAGACCTAAATGTAAAACACAAAACTCTAAGACTCTAGAAG
ATAACACAAGGGAAAATTTAGATGACCTTGGGTTTGGCAATTAGTTTTTAGAAACACCAAAGCACAATGCAT
GAGGGAAAGAAAAAAAGGTAAACTGGACTTTTCCAATTTAATTTCATTAAAACTAAAACTTCCTACTCT**GGC**
CGGGCGCGGTGGCTCACGCCTGTAATCCCAGCACTTTGGGAGGCCGAGGCGGGCGGATCACGAGGTCAGGAG
ATCGAGACCATCCCGGCTAAAACGGTGAAACCCCGTCTCTACTAAAAATACAAAAAATTAGCCGGGCGTAGT
GGCGGGCGCCTGTAGTCCCAGCTACTTGGGAGGCTGAGGCAGGAGAATGGCGTGAACCCGGGAGGCGGAGCT
TGCAGTGAGCCGAGATCCCGCCACTGCACTCC… [NEOcassette]*AAAAAAAAAAAAAAAAACATTAC*
AAAAAAAAAAAAAAAAAAAAAAAAAAAAAAAAAAGAAAAAAAAAAAAAAAAAAAAAAAAAAAAAAAAAAAAAA
AAAAAAAAAAAAAAAAAAAAAAAAAAAAAAAAAAAAAAAAACTTCCTACTCTTTAAAAACACTGTTAAGAAA
ATGAAAAAACAAGCCACAGACTGGGAGAAAATATGTTCGAAACACAAATTGAATCAAGGACTTGAATCCAAA
ATATAAAA

LINE1 flanking the insertion site.

CLONE 83

[D4, D41, D43, D44, D46, D62, D68, D84, D176]

Driver: ORF2
Plasmid: AlurescueA70Du $A_{17}CATTACA_{18}GA_{17}CACACA_{18}$ (T)
Chromosome: 17
5' position: 8,072,661
Strand: minus
DR: GAGAAATGCAGAT
ENDOsite: TCTC/AT

Empty site:

AAAACTATGCATAGTACAAAAAATTATAAGCAAAAGTTAAAGAATAGATGATAATCTAGGGGGAATATTTTA
AATTCATATTGCAGAGAAAGGACTAATTTTGACATATATATATATCTACAAATTTACAAGATAAATACCAAT
AACCCAATAAAACTGTACAAAACATTTCTACAGAAATTTCACTGGTAAGGAAATACAAAAAACTCTCAACTA
TATGAAAAGATGCCTAACCTCATTCATCAAT↑**GAGAAATGCAGAT**↓CATGTTTCAACTTAGCATATTTGTAA
TCACCAAAAAGTTTCATAGAGTGTTGGAGGGGGTGTGGGCAAACAGGCGTTGCCTATTTGTTGGTAGGAGTA
AA

Filled site:

AAAACTATGCATAGTACAAAAAATTATAAGCAAAAGTTAAAGAATAGATGATAATCTAGGGGGAATATTTTA
AATTCATATTGCAGAGAAAGGACTAATTTTGACATATATATATATCTACAAATTTACAAGATAAATACCAAT
AACCCAATAAAACTGTACAAAACATTTCTACAGAAATTTCACTGGTAAGGAAATACAAAAAACTCTCAACTA
TATGAAAAGATGCCTAACCTCATTCATCAATGAGAAATGCAGAT**GGCCGGGCGCGGTGGCTCACGCCTGTAA**
TCCCAGCACTTTGGGAGGCCGAGGCGGGCGGATCACGAGGTCAGGAGATCGAGACCATCCTGGCTAACACGG
TGAAACCCCGTCTCTACTAAAAAAAAAATACAAAAAATTAGCCGGGCGTGGTAGCGGGCGCCTGTAGTCCCAG
CTACTCGGGAGGCTGAGGCAGGGGAATGGCGTGAACCCGGGAGGCGGAGCTTGCAGTGAGCCGAGATCCCGC
CACTGCACTCCAGCCT…[NEOcassette]*AAAAAAAAAAAAAAAAAAAACATTACAAAAAAAAAAAA*
AAAAAAAAAAAAAAAAAAAAAAAAAGAGAAATGCAGATCATGTTTCAACTTAGCATATTTGTAATCACC
AAAAAGTTTCATAGAGTGTTGGAGGGGGTGTGGGCAAACAGGCGTTGCCTATTTGTTGGTAGGAGTAAA

LINE1 flanking the insertion site.

CLONE 84

[D10, D24, D33, D53, D55, D75, D76, D89, D111, D116, D126, D136]

Driver: ORF2
Plasmid: AlurescueA70Du $A_{17}CATTACA_{18}GA_{17}CACACA_{18}$ (T)
Chromosome: 9
5' position: 33,346,926
Strand: minus
DR: AGAAACTGG
ENDOsite: TTCT/GA

Empty site:

TGAGACTAACAATTCTTAAGTAATAAAATCATGAACCTCTGCCTGGAACAATGAGCACTTGAGAGAGAAAAC
TTACCTCATGCTTGCCCATGCACCACTTCTGAGTTAGGAAAGTGCAAGGGGGACACTTCTCCTCACTATGAC
AAGAATGATATACTGCAGAAAGAGAAAGGTAACTTTAGGAATACTTTCTAAATAAAGCCATGTAAACCATTA
TACATCATATAAGTGGCATACGGCATGAAACTTTTAAATTC↑**AGAAACTGG**↓GAGTACTTTTGAAAAACGGT
CACAGGAAAGTAAAAAGTACCTTGCTTTATGTTAAAGTGTTGAAGGACAAGCATGAGTGAGAAAAAGAAACC
CAACCAAG

Filled site:

TGAGACTAACAATTCTTAAGTAATAAAATCATGAACCTCTGCCTGGAACAATGAGCACTTGAGAGAGAAAAC
TTACCTCATGCTTGCCCATGCACCACTTCTGAGTTAGGAAAGTGCAAGGGGGACACTTCTCCTCACTATGAC
AAGAATGATATACTGCAGAAAGAGAAAGGTAACTTTAGGAATACTTTCTAAATAAAGCCATGTAAACCATTA
TACATCATATAAGTGGCATACGGCATGAAACTTTTAAATTCAGAAACTGG**GGCCGGGCGCGGTGGCTCACGC**
CTGTAATCCCAGCACTTTGGGAGGCCGAGGCGGGCGGATCACGAGGTCAGGAGATCGAGACCATCCTGGCTA
ACACGGTGAAACCCCGTCTCTACTAAAAAAAAATACAAAAAATTAGCCGGGCGTGGTAGCGGGCGCCTGTAG
TCCCAGCTACTCGGGAGGCTGAGGCAGGGGAATGGCGTGAACCCGGGAGGCGGAGCTTGCAGTGAGCCGAGA
TCCCGCCACTGCACTCCAGCCTG... [NEOcassette] ***AAAAAAAAAAAAAAAAAAAAAAAACATTACA***G
AAACTGGGAGTACTTTTGAAAAACGGTCACAGGAAAGTAAAAAGTACCTTGCTTTATGTTAAAGTGTTGAAG
GACAAGCATGAGTGAGAAAAAGAAACCCAACCAAG

No repetitive sequences in the immediate proximity.

CLONE 85
[C4]

Driver: ORF2
Plasmid: AlurescueA70Du $A_{17}CATTACA_{18}GA_{17}CACACA_{18}$ (T)
Chromosome: 3
5' position: 147,900,227
Strand: minus
DR: AGAAATTAGTTTTG
ENDOsite: TTCT/AT

Empty site:

AGTTATAAGTTCTTCTTAAGAACTTCTTAAATCCTTTCTTAAGTTCTTCTACTTTTAGATATTTTATTGTTG
AAATACAATAAAGAAAACTAAAAACAGCCATATGTTGCTCAATTTGTTCAATTTATTTTCAATTGAGATTTT
TCTTGATCTAAAATGGTTTCTAT↑**AGAAATTAGTTTTG**↓ATTTTATTTTACAAGAATCTCCTTCATAGTCAT
ATGAAATTTTTAACTAATGTTCTTGCAACGGAATGTCACTTTTGTTGCATATTTTACAAGTAGGTTT

Filled site:

AGTTATAAGTTCTTCTTAAGAACTTCTTAAATCCTTTCTTAAGTTCTTCTACTTTTAGATATTTTATTGTTG
AAATACAATAAAGAAAACTAAAAACAGCCATATGTTGCTCAATTTGTTCAATTTATTTTCAATTGAGATTTT
TCTTGATCTAAAATGGTTTCTATAGAAATTAGTTTTG**GGCCGGGCGCGGTGGCTCACGCCTGTAATCCCAGC**
ACTTTGGGAGGCCGAGGCGGGCGGATCACGAGGTCAGGAGATCGAGACCATCCCGGCTAAAACGGTGAAACC
CCGTCTCTACTAAAAATACAAAAAATTAGCCGGGCGTAGTGGCGGGCGCCTGTAGTCCCAGCTACTTGGGAG
GCTGAGGCAGGAGAATGGCGTGAACCCGGGAGGCGGAGCTTGCAGTGAGCCGAGATCCCGCCACTGCACTCC
AG… [NEOcassette] ***AAAAAAAAAAAAAAAAAAAAAAAAAAAAAAAAAAAAAAAAAAAAAAAAAAAAAAA***
AAAAAAAAAAAAAACATTACAAAAAAAAAAAAAAAAAAAAAAAAAAAAAAAAAAAAAAAAAAAAAAAAAAAA
AAAAAAAAAAAAAAGAAATTAGTTTTGATTTTATTTTACAAGAATCTCCTTCATAGTCATATGAAATTTTTA
ACTAATGTTCTTGCAACGGAATGTCACTTTTGTTGCATATTTTACAAGTAGGTTT

DNA element flanking the insertion site.

CLONE 87

[C16]

Driver: ORF2
Plasmid: AlurescueA70Du $A_{17}CATTACA_{18}GA_{17}CACACA_{18}$ (T)
Chromosome: 12
5' position: 95,168,036
Strand: plus
DR: TAAAATCTAGTCT
ENDOsite: TTTA/AA

Empty site:

GAATTCAGAGACAGCTTAGGTGGGTTGTTCTGGTTCAGGATCACTCAGATTTGCAGTTCAGATGCCAATCAG
GGCTGTGTTCATCTGAAGGCTTGACTGGGGCTGCACAATCTACTTCCAAGATACCCCACTCACATAGCTGTT
GGCAGAAAGCCTCAGTTTCCTTGCCATGTGGGCCTCTCCATAGGGTTGAGTGTGAAGCCGTATGGCAGGTGG
CTTCCCCAAGAGCTAGTAATCTAAGAGAGGGCAAAATAGAAGCCACAATGCCTTT↑**TAAAATCTAGTCT**↓CC
AAAGTCTAGCAATATCACTCCTTTAACTTTCTATTAATTAGAAATGAGTCTTCAAGTACAGCCCATGTTAAG
GGGAAGAGAATTAGGCTCCATCTTTC

Filled site:

GAATTCAGAGACAGCTTAGGTGGGTTGTTCTGGTTCAGGATCACTCAGATTTGCAGTTCAGATGCCAATCAG
GGCTGTGTTCATCTGAAGGCTTGACTGGGGCTGCACAATCTACTTCCAAGATACCCCACTCACATAGCTGTT
GGCAGAAAGCCTCAGTTTCCTTGCCATGTGGGCCTCTCCATAGGGTTGAGTGTGAAGCCGTATGGCAGGTGG
CTTCCCCAAGAGCTAGTAATCTAAGAGAGGGCAAAATAGAAGCCACAATGCCTTTTAAAATCTAGTCT**GGCC**
GGGCGCGGTGGCTCACGCCTGTAATCCCAGCACTTTGGGAGGCCGAGGCGGGCGGATCACGAGGTCAGGAGA
TCGAGACCATCCCGGCTAAAACGGTGAAACCCCGTCTCTACTAAAAATACAAAAAATTAGCCGGGCGTAGTG
GCGGGCGCCTGTAGTCCCAGCTACTTGGGAGGCTGAGGCAGGAGAATGGCGTGAACCCGGGAGGCGGAGCTT
GCAGTGAGCCGAGATCCCGCCACTGCACTCC... [NEOcassette] ***AAAAAAAAAAAAAAAAAAAAAAAAAA***
AAAAAAAAAAAAAAAAAAAAAAAAAACATTACAAAAAAAAAAAAAAAAAAAAAAAAAAAAAAAAAAAAAAAAA
AAAAAAAAAAAAAAAAAAAAAAAAAAAAAAAAAGTAAAATCTAGTCTCCAAAGTCTAGCAATATCACTCCTT
TAACTTTCTATTAATTAGAAATGAGTCTTCAAGTACAGCCCATGTTAAGGGGAAGAGAATTAGGCTCCATCT
TTC

LTR flanking the insertion site.

CLONE 88

[Brown Alu rescue MP#69 ,55, 59, 105, 87, 86,67, C1, C38, C51, colored 1]

Driver: ORF2
Plasmid: AlurescueA70Du $A_{17}CATTACA_{18}GA_{17}CACACA_{18}$ (T)
Chromosome: 9
5' position: 126,744,038
Strand: minus
DR: AAACAATAACTTCT
ENDOsite: GTTT/AT

Empty site:

GTTCAGTAAGTTGCCTGACACATGGGGAGCACACAAATAAAGATTAAAAAAATAAAGATTAAAAAATGTAGC
ATGGTTTTTTGTTTTTGTTTTTTTAGTCTAGGAAGAATTTTTATTAGAAGTAGGAAGAAATGTCAGGTTCTT
TTATTGTGGGGGAAACACACATAATATGGAACTAACTGGCTTAACCATTTTTTAAATGTACATTTCAGGGAC
GTTAAGTACATTCATATGTGCAACCATCACCACCATCCATCTCTAAAACTTTTTCATCTTCCCTACTGAACC
TCTAAACCCAAT↑**AAACAATAACTTCT**↓TGCTCCTCCCTTTCCTCTAGTCTATGTCAACCACCATTCTTTCT
TCTGTTTCTATGAATTTGGCTATTCTAGGTATCTCACATAAGTGAAATCATACAGT

Filled site:

GTTCAGTAAGTTGCCTGACACATGGGGAGCACACAAATAAAGATTAAAAAAATAAAGATTAAAAAATGTAGC
ATGGTTTTTTGTTTTTGTTTTTTTAGTCTAGGAAGAATTTTTATTAGAAGTAGGAAGAAATGTCAGGTTCTT
TTATTGTGGGGGAAACACACATAATATGGAACTAACTGGCTTAACCATTTTTTAAATGTACATTTCAGGGAC
GTTAAGTACATTCATATGTGCAACCATCACCACCATCCATCTCTAAAACTTTTTCATCTTCCCTACTGAACC
TCTAAACCCAATAAACAATAACTTCT**GGCCGGGCGCGGTGGCTCACGCCTGTAATCCCAGCACTTTGGGAGG**
CCGAGGCGGGCGGATCACGAGGTCAGGAGATCGAGACCATCCCGGCTAAAACGGTGAAACCCCGTCTCTACT
AAAAATACAAAAAATTAGCCGGGCGTAGTGGCGGGCGCCTGTAGTCCCAGCTACTTGGGAGGCTGAGGCAGG
AGAATGGCGTGAACCCGGGAGGCGGAGCTTGCAGTGAGCCGAGATCCCGCCACTGCACTCCAG… [NEOcas
sette]*AAAAAAAAAAAAAAAAAAAAAAAAAAAAAAAAAAAAAAAAAAAAAAAAAAAAAAAAAAAAAAAAA*
AAAAAAAAAAAAAAAAAAAAAAAAAAAAAAAAAAAAAAAAAAAAAAACAATAACTTCTTGCTCCTCCCTTTCC
TCTAGTCTATGTCAACCACCATTCTTTCTTCTGTTTCTATGAATTTGGCTATTCTAGGTATCTCACATAAGT
GAAATCATACAGT

LINE-1 flanking the insertion site.

CLONE 89

[Green Alu rescue MP#9,10,11,12,59,C10, C12, colored 15]

Driver: ORF2
Plasmid: AlurescueA70Du $A_{17}CATTACA_{18}GA_{17}CACACA_{18}$ (T)
Chromosome: 18
5' position: 72,573,745
Strand: minus
DR: AAAAATATTTTTTA
ENDOsite: TTTT/AT

Empty site:

GAAGATTTTAATAACAAATTTAAACAATTTTAGAGAACTTTTAAAGAAAATTCCTAAAGTTCTAAGTCTTAT
CCAGTGGGTATCAAAGAAAATACTATTCACAATTAATATCTAATGAAATATTAATCTTTAAATCATGGAAAG
ATATTCTATTCTCACTAAGCACCTATGAAAACACTGAATTAATCTGTAACTCTATGTGACTTGAAAGTCATA
AATTCAGAATTAAAAACTTCAATTTATCCTCAAGAGTAGGATTTAATTTACAGATTCATAGTTAAATATCCA
TATTCACAAAATCATTACCATTTACAGTAAGGAAATAATGAAAT↑**AAAAATATTTTTTA**↓AATGAAAAACAT
GAAAGGGTATTGAATATAACACGAATATAAAAGTCAATGAAATAACTAAATATATATAGCAGTACAAACATC
CTAAGAATTCAAAGAA

Filled site:

GAAGATTTTAATAACAAATTTAAACAATTTTAGAGAACTTTTAAAGAAAATTCCTAAAGTTCTAAGTCTTAT
CCAGTGGGTATCAAAGAAAATACTATTCACAATTAATATCTAATGAAATATTAATCTTTAAATCATGGAAAG
ATATTCTATTCTCACTAAGCACCTATGAAAACACTGAATTAATCTGTAACTCTATGTGACTTGAAAGTCATA
AATTCAGAATTAAAAACTTCAATTTATCCTCAAGAGTAGGATTTAATTTACAGATTCATAGTTAAATATCCA
TATTCACAAAATCATTACCATTTACAGTAAGGAAATAATGAAATAAAAATATTTTTTA**GGCCGGGCGCGGTG
GCTCACGCCTGTAATCCCAGCACTTTGGGAGGCCGAGGCGGGCGGATCACGAGGTCAGGAGATCGAGACCAT
CCCGGCTAAAACGGTGAAACCCCGTCTCTACTAAAAATACAAAAAATTAGCCGGGCGTAGTGGCGGGCGCCT
GTAGTCCCAGCTACTTGGGAGGCTGAGGCAGGAGAATGGCGTGAACCCGGGAGGCGGAGCTTGCAGTGAGCC
GAGATCCCGCCACTGCACTCCAGC... [NEOcassette]** ***AAAAAAAAAAAAAAAAAAAAACATTACAAA
AAAAAAAAAAAAAAAAAAGAAAAAAAAAAAAAAAAAAAAAAAAAAAAAAAAAAAAA***TATTTTTTAAATGA
AAAACATGAAAGGGTATTGAATATAACACGAATATAAAAGTCAATGAAATAACTAAATATATATAGCAGTAC
AAACATCCTAAGAATTCAAAGAA

No repetitive sequences in the immediate proximity.

CLONE 91

[Brown Alu rescue MP#54, 78, 91, C9, colored 17]

Driver: ORF2
Plasmid: AlurescueA70Du $A_{17}CATTACA_{18}GA_{17}CACACA_{18}$ (T)
Chromosome: 10
5' position: 69,240,667
Strand: plus
DR: AAAAAGTTAATTCACC
ENDOsite: TTTT/GA

Empty site:

TGTTCCTGCTGCAGATAACAAGCCAGAGCCTGTCCCTTTGTTTCCTGTAAGGAATGCTTTTAGCTAATCTAT
AACCTATAGAAACAATGCTTATCACTGGCTTATTGTCAGTAAATAGGTGGGTCAAACTCTGTTTGAGGCTCT
CAGCTCTGAAGGCTGTTAGCTCCCTGATTCCCACTTTGTACTCTATTTCTGTGTCTTTGTCTTAATTCCTCT
AACGCCGCTGGGTTGGGGTCTCCATGACCAAGCTGGTCTTGGCAACGATACTAGCAAACTGAATCCAGTAGC
ACATC↑**AAAAAGTTAATTCACC**↓ATAATCATGTAAGCTTTATTCTAGGGACGCAACGTTGGTTCAACATACA
CAAAAAAATAAAACTAGGCCTAGTTTGTTAGGAGTTTTTATCATGAAGCA

Filled site:

TGTTCCTGCTGCAGATAACAAGCCAGAGCCTGTCCCTTTGTTTCCTGTAAGGAATGCTTTTAGCTAATCTAT
AACCTATAGAAACAATGCTTATCACTGGCTTATTGTCAGTAAATAGGTGGGTCAAACTCTGTTTGAGGCTCT
CAGCTCTGAAGGCTGTTAGCTCCCTGATTCCCACTTTGTACTCTATTTCTGTGTCTTTGTCTTAATTCCTCT
AACGCCGCTGGGTTGGGGTCTCCATGACCAAGCTGGTCTTGGCAACGATACTAGCAAACTGAATCCAGTAGC
ACATCAAAAAGTTAATTCACC**GGCCGGGCGCGGTGGCTCACGCCTGTAATCCCAGCACTTTGGGAGGCCGAG
GCGGGCGGATCACGAGGTCAGGAGATCGAGACCATCCCGGCTAAAACGGTGAAACCCCGTCTCTACTAAAAA
TACAAAAAATTAGCCGGGCGTAGTGGCGGGCGCCTGTAGTCCCAGCTACTTGGGAGGCTGAGGCAGGAGAAT
GGCGTGAACCCGGGAGGCGGAGCTTGCAGTGAGCCGAGATCCCGCCACTGCACTCCAGC…[NEOcassett
e]*AAAAAAAAAAAAAAAAAAAAACATTACAAAAAAAAAAAAAAAAAAAAAAAAGAAAAAAAAAAAAA
AAAAAAAAAAAAAAAAAAAAA***AGTTAATTCACCATAATCATGTAAGCTTTATTCTAGGGACGCAACGTTGG
TTCAACATACACAAAAAAATAAAACTAGGCCTAGTTTGTTAGGAGTTTTTATCATGAAGCA

LTR at the 5'; LINE-1 flanking the insertion site.

CLONE 92

[Brown Alu rescue MP#22, colored 26, C32b]

Driver: ORF2
Plasmid: AlurescueA70Du $A_{17}CATTACA_{18}GA_{17}CACACA_{18}$ (T)
Chromosome: 1
5' position: 68060762
Strand: plus
DR: AAGAGAAAGCATAAA
ENDOsite: TCTT/CA

Empty site:

TGCCCTTGACATGTGGGGATTATTACAATTCAAGGTGAGATTTGGGTGGGGACACAGAGCCAAACCATATCA
AGGGGGAATAAAGGCCCATGGCTAATTCCAGAGGCCTGACCGTGAGAGAATGATGGCTGCCTCTCAAATTTT
GGCTCAGATTGAACAACTCACCCTGACCTCTGACGCTGCAGGGTTGCTGCCCAACACCCTTGCAGTGGCTTG
TTTCAACAGACATGGAAGGGACCAGAACCACTGCAGCCTGACTGCAGACAGGCACTGTCTGAGCTAGGATCA
GCTTTCTGTTATGGCAGAGGGAGAGCTTG↑**AAGAGAAAGCATAAA**↓CTAAGAAGCAGCCTCTGGGGTAAAAA
ATTTAGCAGTCTCTTACTGACAGGCCTAAACCAATCTGCTGGTTTAACAACTGTCAGCTTCTCTTGATAGAT
AT

Filled site:

TGCCCTTGACATGTGGGGATTATTACAATTCAAGGTGAGATTTGGGTGGGGACACAGAGCCAAACCATATCA
AGGGGGAATAAAGGCCCATGGCTAATTCCAGAGGCCTGACCGTGAGAGAATGATGGCTGCCTCTCAAATTTT
GGCTCAGATTGAACAACTCACCCTGACCTCTGACGCTGCAGGGTTGCTGCCCAACACCCTTGCAGTGGCTTG
TTTCAACAGACATGGAAGGGACCAGAACCACTGCAGCCTGACTGCAGACAGGCACTGTCTGAGCTAGGATCA
GCTTTCTGTTATGGCAGAGGGAGAGCTTGAAGAGAAAGCATAAA**GCCGGGCGCGGTGGCTCACGCCTGTAAT
CCCAGCACTTTGGGAGGCCGAGGCGGGCGGATCACGAGGTCAGGAGATCGAGACCATCCCGGCTAAAACGGT
GAAACCCCGTCTCTACTAAAAATACAAAAAATTAGCCGGGCGTAGTGGCGGGCGCCTGTAGTCCCAGCTACT
TGGGAGGCTGAGGCAGGAGAATGGCGTGAACCCGGGAGGCGGAGCTTGCAGTGAGCCGAGATCCCGCCACTG
CACTCCAG…[NEOcassette]*AAAAAAAAAAAAAAAAAAAAACATTACAAAAAAAAAAAAAAAAAAAAAA
AAAAAAAAGAAAGAAAAAAAAAAAAAAAAAAAAAAAAAAAAAAAAAACACACAAAAAAAAAAAAAAAAAA
AAAAAAAAAAAAAAAAAAAAAAAAAAA*** GAGAAAGCATAAACTAAGAAGCAGCCTCTGGGGTAAAAAATTTAG
CAGTCTCTTACTGACAGGCCTAAACCAATCTGCTGGTTTAACAACTGTCAGCTTCTCTTGATAGATAT

LTR at the 5'.

CLONE 93

[Brown Alu rescue MP#27, colored 27]

Driver: ORF2
Plasmid: AlurescueA70Du $A_{17}CATTACA_{18}GA_{17}CACACA_{18}$ (T)
Chromosome: 13
5' position: 37536966
Strand: plus
DR: GAGATGTGCT
ENDOsite: TCTC/AA

Empty site:

GGTTTCACCATTGTTGCCCATGCTGGTTTCAAATTCCTGGACTCAAGCAATCCACCTGCCTCAGCCTCCCAA
AGTGCTAGGATGACCACTGTAAGCCACCATCCCCAGCCACTAAGTGAGGTCAATCTTATGTCAATTAACACA
AAATAAATAAAAGGCTTGCATGTAACATTAACACTAAAAAAAGAAAAAAACCCCATTAATGTTCTTTAACTT
AACCCCACAAGAACTGCTGTCTAATACAACAGCCCCTCAGCACATGAGGATACTGAGCACTTGAAATGTGGC
TAGTCTGAATT↑**GAGATGTGCT**↓GTAAATTTGAATATATAGTACAAAGAAAATAATGTAAAATAGTTCAACT
TTTTTCATACTGATTGCATGGTGATATGACAATATCTTGGAAATATATTGG

Filled site:

GGTTTCACCATTGTTGCCCATGCTGGTTTCAAATTCCTGGACTCAAGCAATCCACCTGCCTCAGCCTCCCAA
AGTGCTAGGATGACCACTGTAAGCCACCATCCCCAGCCACTAAGTGAGGTCAATCTTATGTCAATTAACACA
AAATAAATAAAAGGCTTGCATGTAACATTAACACTAAAAAAAGAAAAAAACCCCATTAATGTTCTTTAACTT
AACCCCACAAGAACTGCTGTCTAATACAACAGCCCCTCAGCACATGAGGATACTGAGCACTTGAAATGTGGC
TAGTCTGAATTGAGATGTGCT**GGCCGGGCGCGGTGGCTCACGCCTGTAATCCCAGCACTTTGGGAGGCCGAG**
GCGGGCGGATCACGAGGTCAGGAGATCGAGACCATCCCGGCTAAAACGGTGAAACCCCGTCTCTACTAAAAA
TACAAAAAATTAGCCGGGCGTAGTGGCGGGCGCCTGTAGTCCCAGCTACTTGGGAGGCTGAGGCAGGAGAAT
GGCGTGAACCCGGGAGGCGGAGCTTGCAGTGAGCCGAGATCCCGCCACTGCACTCCAG… [NEOcassette
]*AAAAAAAAAAAAAAAAAAAAAAACATTACAAAAAAAAAAAAAAAAAAAAAAAGAAAAAAAAAAAAAAAAAA*
AAAAAAAAAAAAAAAAAAAAAAAAAAAAAAAAAAAAAAAAAAAAAAAAAAAAAGAGATGTGCTGTAAATTTG
AATATATAGTACAAAGAAAATAATGTAAAATAGTTCAACTTTTTTCATACTGATTGCATGGTGATATGACAA
TATCTTGGAAATATATTGG

Alu at the 5'; hAT-Charlie DNA element flanking insertion site.

CLONE 94

[Green Alu rescue MP#13,15,17,18,29,30, colored 32]

Driver: ORF2
Plasmid: Alu Jo element; AlurescueA70Du $A_{17}CATTACA_{18}GA_{17}CACACA_{18}$ (T)
Chromosome: 3
5’ position: 189,651,214
Strand: minus
DR: AACAGGCTA
ENDOsite: TGTT/GA

Empty site:

TGTTTTCCTATATGCCACACAGCTTGCAACATTCTGGGATAATACAAATTAAATATAACATCCTGAGTTCTT
CATGTTAAAATGAATAAATCCACAATAAACTGCAAGTTATATGAAGCAATTTTATGATACTGTTAGAGCTAC
ATTTTAAAATCCATAAATTACAAGGTAGTTGACACTCATGAACATAGATGCAAAAATCCTCAAGATACTATT
AGCAAATGAAATGCAACAACCTACAAAAAGAATTATACATCATGACCAAGTGGGATTTATTTTAGGATGCA
CGGCTATTTCAGCATTCACTAAATTAATTAATTTAATCCACCACATC↑**AACAGGCTA**↓AAAGCCTGGGCAAC
ATAGGAAGACCCTGTCTCTACAAAAAAAGTTAAAAAAAAAAAGTAGCTGGATACTGTGGCATGTCCCTGTAGT
CCCAGTTATTTGGGAGGCCGAGATGGGGGTGTTTCTTAAGCCCAGGAGTTCTAGGCCGCAGTGAACTGTGAT
CACACCTGGACTCTAGCCTGGGTGACAGAATGAGACCTTGTC

Filled site:

TGTTTTCCTATATGCCACACAGCTTGCAACATTCTGGGATAATACAAATTAAATATAACATCCTGAGTTCTT
CATGTTAAAATGAATAAATCCACAATAAACTGCAAGTTATATGAAGCAATTTTATGATACTGTTAGAGCTAC
ATTTTAAAATCCATAAATTACAAGGTAGTTGACACTCATGAACATAGATGCAAAAATCCTCAAGATACTATT
AGCAAATGAAATGCAACAACCTACAAAAAGAATTATACATCATGACCAAGTGGGATTTATTTTAGGATGCA
CGGCTATTTCAGCATTCACTAAATTAATTAATTTAATCCACCACATCAACAGGCTA**GGCCGGGCGCGGTGGC
TCACGCCTGTAATCCCAGCACTTTGGGAGGCCGAGGCGGGAGGATTGCTTGAGCCCAGGAGTTCGAGACCAG
CCTGGGCAACATAGCGAGACCCCGTCTCTACAAAAAATACAAAAATTAGCCGGGCGTGGTGGCGCGCGCCTG
TAGTCCCAGCTACTCGGGAGGCTGAGGCAGGAGGATCGCTTGAGCCCAGGAGTTCGAGGCTGCAGTGAGCTA
TGATCGCGCCACTGCACTCCAGCCTGGGCGACAGAGCGAGAC… [NEOcassette]** ***AAAAAAAAAAAAA
AAAAAAAAAAAAAAAAAAAAAAAAAAAAAAAAAAACATTACAA***CAGGCTAAAAGCCTGGGCAACATAGGAA
GACCCTGTCTCTACAAAAAAAGTTAAAAAAAAAAAGTAGCTGGATACTGTGGCATGTCCCTGTAGTCCCAGTT
ATTTGGG

LINE-1 flanking the insertion site; Alu at the 3’.

CLONE 95

[Green MP#85, 86, 93, colored 40]

Driver: ORF2
Plasmid: AlurescueA70Du $A_{17}CATTACA_{18}GA_{17}CACACA_{18}$ (T)
Chromosome: 10
5’ position: 80,221,124
Strand: minus
DR: AAAGCAGGCTTCT
ENDOsite: CTTT/AC

Empty site:

CCATTGAGACTAAAGGGCCATAGCGTAGGAGCAATAGGCAGCTGGGCCGGAGTGGCCTCAGTGAGTCAATGC
AGGGATATTCACTGAGCATGTAGCTTGGGCCAGGGACTGCTTAAAAGGAAGCTGCTGGGGGAACACCGACCA
TGGTCCTCAGCAGAGCAGGAAGCTGGGATCCCAGGAGCCATGGCAAGCTCCAGATGAGAAGTGAGTACAAAC
TTGCAGAACAGACAAAGAGTGAGGAAGTGGAGGCTAGGTCAAAAAGCGGAGAGT↑**AAAGCAGGCTTCT**↓GTC
CTTCAAGGCCCAGCCCAGAAGACCCTATGCCCAGTGATATAAGATCCTCATCACTCTGCCCCAGGAACAACT
GGTATGATCCATGAGCAGTCCTGGG

Filled site:

CCATTGAGACTAAAGGGCCATAGCGTAGGAGCAATAGGCAGCTGGGCCGGAGTGGCCTCAGTGAGTCAATGC
AGGGATATTCACTGAGCATGTAGCTTGGGCCAGGGACTGCTTAAAAGGAAGCTGCTGGGGGAACACCGACCA
TGGTCCTCAGCAGAGCAGGAAGCTGGGATCCCAGGAGCCATGGCAAGCTCCAGATGAGAAGTGAGTACAAAC
TTGCAGAACAGACAAAGAGTGAGGAAGTGGAGGCTAGGTCAAAAAGCGGAGAGTAAAGCAGGCTTCTGGCCG
GGCGCGGTGGCTCACGCCTGTAATCCCAGCACTTTGGGAGGCCGAGGCGGGCGGATCACGAGGTCAGGAGAT
CGAGACCATCCCGGCTAAAACGGTGAAACCCCGTCTCTACTAAAAATACAAAAAATTAGCCGGGCGTAGTGG
CGGGCGCCTGTAGTCCCAGCTACTTGGGAGGCTGAGGCAGGAGAATGGCGTGAACCCGGGAGGCGGAGCTTG
CAGTGAGCCGAGATCCCGCCACTGCACTCCAGCCTGGGCGACAGAGCGAGACGTCTC… **[NEOcassette]**
AAAAAAAAAAAAAAAAAAAAAAAAAAAAAAAAAAAAAAAAAAAAAAAAAAAAAAAAAAAAAAAAAAAAAAAA
AAAAACATTACAAAAAAAAAAAAAAAAAAAAAAAAAAAAAAAGAAAAAAAAAAAAAAAAAAAAAAAAAAAAA
AAAAAAAAAAAAAAAAAAAAAAAAAAAAAAAAAAAAAAAAAAAAAAAAAAAAAAAAAAAAAAAAAAAAAAAA
AAAAAAAAAAAAAAAAAAAAAAAAAAAAAAAAAAAGCAGGCTTCTGTCCTTCAAGGCCCAGCCCAGAAGACC
CTATGCCCAGTGATATAAGATCCTCATCACTCTGCCCCAGGAACAACTGGTATGATCCATGAGCAGTCCTGG
G

No repetitive sequences in the immediate proximity.

CLONE 96

[Green Alu rescue MP#87 81 "Green 87," colored 16]

Driver: ORF2
Plasmid: AlurescueA70Du $A_{17}CATTACA_{18}GA_{17}CACACA_{18}$ (T)
Chromosome: 2
5' position: 133,685,841
Strand: minus
DR: AAGAGCTTCAGGAA
ENDOsite: TCTT/AA

Empty site:

TTGCCCACTTGAGTTTTTCAGCAAGTAATAAATAATTACAAAGCAACTTACAAGATAGGTATATGTGTCATA
GCTATTATTTATCTTTCTGCTTATCTCAAAGAAGAGATAGAGTTTTTCACAGTTTAGCAAAGACACAAACCA
TGTCTTTGTTAAGGAAACCTCATCAATTAATGAGTAATATTAATTATTGTAGGTGCAAAGAAACATTTGGTC
ATTCAGCAAGCATTTTATCTTAATTTATCATACGTAGTGCTTACCACATGCCAGATGTTGTTTT↑**AAGAGCT
TCAGGAA**↓TAATTCATTTAATTCTCCCTGTAACCTTATAAGATAATTGTATTATTGTAACCATTTTACAGGT
GGAGATACTGAGGCACAGAGGAGTTAACTAACTTATTCAAGTCCAGTTAGTTAATAAGTGGTAAAGCTGACA
TTGGATTCCCAAGTAGTCTGACCTCTTAAACACTATCCTGTTCTATTTCCTACTATATTCAAAA

Filled site:

TTGCCCACTTGAGTTTTTCAGCAAGTAATAAATAATTACAAAGCAACTTACAAGATAGGTATATGTGTCATA
GCTATTATTTATCTTTCTGCTTATCTCAAAGAAGAGATAGAGTTTTTCACAGTTTAGCAAAGACACAAACCA
TGTCTTTGTTAAGGAAACCTCATCAATTAATGAGTAATATTAATTATTGTAGGTGCAAAGAAACATTTGGTC
ATTCAGCAAGCATTTTATCTTAATTTATCATACGTAGTGCTTACCACATGCCAGATGTTGTTTTAAGAGCTT
CAGGAA**GGCCGGGCGCGGTGGCTCACGCCTGTAATCCCAGCACTTTGGGAGGCCGAGGCGGGCGGATCACGA
GGTCAGGAGATCGAGACCATCCCGGCTAAAACGGTGAAACCCCGTCTCTACTAAAAATACAAAAAATTAGCC
GGGCGTAGTGGCGGGCGCCTGTAGTCCCAGCTACTTGGGAGGCTGAGGCAGGAGAATGGCGTGAACCCGGGA
GGCGGAGCTTGCAGTGAGCCGAGATCCCGCCACTGC... [NEOcassette]** ***AAAAAAAAAAAAAAAAAAAAA
AAAAAAAAAAAAAAAAAAAAAAAAACATTACAAAAAAAAAAAAAAAAAAAAAAAAAAAAAAAAAAAAAAAAA
AAAAAAAAAAAAAAAAAAAAAAAGAAAAAAAAAAAAA***GAGCTTCAGGAATAATTCATTTAATTCTCCCTGTA
ACCTTATAAGATAATTGTATTATTGTAACCATTTTACAGGTGGAGATACTGAGGCACAGAGGAGTTAACTAA
CTTAT

MIR flanking the insertion site.

CLONE 97

[Blue Alu rescue MP#25 Red36, colored 22]

Driver: ORF2
Plasmid: AlurescueA70Du $A_{17}CATTACA_{18}GA_{17}CACACA_{18}$ (T)
Chromosome: 1
5' position: 15646423
Strand: plus
DR: AATGAAAATGAACTTGC
ENDOsite: CAGTT/GA

Empty site:

GTAAAATATAATATGGGGAAAATGGACATGGTAGAAATGAAAACTACAAAATAAAACACAGACAGACAGACC
TGTGATTGGTAAATATTTGATAGGGTCCAGAAAAACTTATGGATGAATCAAATCATAATTGTATAATTTGCC
TACAAAAGAACTGATCCAGATCAAAATAATTTCAGGAGACTAAAGTGAAAATGGAAACATTTGGAAATCTGT
TAAACAACTGGCTTAATGAACTTTGCTCTAGAAAATACCCTCTC↑**AATGAAAATGAACTTGC**↓TATGGTATA
TTTTTCTTTTAAATAGTTGTAGTCATGAACATGGAGTCAAAATGCTCTCTGGGCTATCAATTTTTCTCTAAA
CAAGGCTTTGGCTGCATTC

Filled site:

GTAAAATATAATATGGGGAAAATGGACATGGTAGAAATGAAAACTACAAAATAAAACACAGACAGACAGACC
TGTGATTGGTAAATATTTGATAGGGTCCAGAAAAACTTATGGATGAATCAAATCATAATTGTATAATTTGCC
TACAAAAGAACTGATCCAGATCAAAATAATTTCAGGAGACTAAAGTGAAAATGGAAACATTTGGAAATCTGT
TAAACAACTGGCTTAATGAACTTTGCTCTAGAAAATACCCTCTC<u>AATGAAAATGAACTTGC</u>**GGCCGGGCGCG**
GTGGCTCACGCCTGTAATCCCAGCACTTTGGGAGGCCGAGGCGGGCGGATCACGAGGTCAGGAGATCGAGAC
CATCCCGGCTAAAACGGTGAAACCCCGTCTCTACTAAAAATACAAAAAATTAGCCGGGCGTAGTGGCGGGCG
CCTGTAGTCCCAGCTACTTGGGAGGCTGAGGCAGGAGAATGGCGTGAACCCGGGAGGCGGAGCTTGCAGTGA
GCCGAGATCCCGCCACTGCACTCCAG…[NEOcassette]*AAAAAAAAAAAAAAAAAAACATTACAAA*
AAAAAAAAAAAAAAAAAAAAAAGGAAAAAAAAAAAAAAAAAAAAAAAAAAAAAAAAAAAAAAAAAA
AAAAAAAAAAAAAAAAAAAAAAAAAAA<u>AA</u><u>TGAAAATGAACTTGC</u>TATGGTATATTTTTCTTTTAAATAGT
TGTAGTCATGAACATGGAGTCAAAATGCTCTCTGGGCTATCAATTTTTCTCTAAACAAGGCTTTGGCTGCAT
TC

No repetitive sequences in the immediate proximity.

CLONE 98

[Black Alu rescue MP#2, Black2, purple #7, colored 37]

Driver: ORF2
Plasmid: AlurescueA70Du $A_{17}CATTACA_{18}GA_{17}CACACA_{18}$ (T)
Chromosome: 1
5' position: 218,884,781
Strand: minus
DR: AAGTGACCT
ENDOsite: ACTT/GC

Empty site:

TATAACACAATGCTGATCATATAGTCAGAGTATAAATATATATATTAATTTTTTAATAAGTGAATGAACATA
TAACACAAGATGTTTACAAGATAAGATGCCTTTTAAGTATGGACTAAGTACTTTCAATTTTTTCTGCAATAC
TATATGGAAGTATTAGCCTCTTCGCACCTGTCTGCAGGCTACTGGAAGACCCACTAAACTGGTTCATAGAAG
CGTCACAAGGTTTAGGACAATCCACAGGGATGGCTGTGGTGCTTGCCTTTCACCCTGCCCAGTAGGCATATC
TGAGAGGGTTGTATGGTCCTATGACCAGGCTGAAGC↑**AAGTGACCT**↓ATGAGTGGCACCATGGATGACATGG
GAATGACAAAGCCCACAAGGAAGCCCCTGCTGTCACTCTTTCACCTTGCTGGGTTTCTAACTTTCGAGATCT
GAG

Filled site:

TATAACACAATGCTGATCATATAGTCAGAGTATAAATATATATATTAATTTTTTAATAAGTGAATGAACATA
TAACACAAGATGTTTACAAGATAAGATGCCTTTTAAGTATGGACTAAGTACTTTCAATTTTTTCTGCAATAC
TATATGGAAGTATTAGCCTCTTCGCACCTGTCTGCAGGCTACTGGAAGACCCACTAAACTGGTTCATAGAAG
CGTCACAAGGTTTAGGACAATCCACAGGGATGGCTGTGGTGCTTGCCTTTCACCCTGCCCAGTAGGCATATC
TGAGAGGGTTGTATGGTCCTATGACCAGGCTGAAGCAAGTGACCT **[noCleanAluSequenceRead]**…
[NEOcassette]***AAAAAAAAAAAAAAAAAAAAAAAAAAAAAAAAAACATTACAAAAAAAAAAAAA***
AAAAAAAAAAAAAAAAAAAAAAAAAAAAAAAAAAAAAAAAAAAAAAAAAAAAAAAAAAAAAAAAAA
AAAAAAAAAAAAAAAAAAAAAAAAAAAAAAAAAAAAAAAAAAAAAAAAAAAAAAAAAAAAAAAAGTGA
CCTATGAGTGGCACCATGGATGACATGGGAATGACAAAGCCCACAAGGAAGCCCCTGCTGTCACTCTTTCAC
CTTGCTGGGTTTCTAACTTTCGAGATCTGAG

No repetitive sequences in the immediate proximity.

CLONE 99

[Brown Alu rescue MP#21, colored 44]

Driver: ORF2
Plasmid: AlurescueA70Du $A_{17}CATTACA_{18}GA_{17}CACACA_{18}$ (T)
Chromosome: 12
5' position: 13131825
Strand: minus
DR: TAAAAGGCAGAATG
ENDOsite: TTTA/AT

Empty site:

TTGGGGCATTCTGGACACAGATGCAGTCCTGTTTTTTCCTGCACCCTTCCCCCAGTGCTTCAGCCTTTTCAG
GACAGATGCATACTGTCATTTGTGCTCTGAAGCCTCAGTATTCTGCATGGTGCCTGATCCAGAGCACCTGCC
AGGTATTTATTGAATCAGTCTGTTTCCATTTGAAAATGCCCATGGTAAGAAAAATGTAGATGCCACTGGAAT
CTGTTGTCTTCAAGGCCTTTTGGTTAAAAGGGACAGAAACCCATCAGCTAAAGCAGAAAAGGGAGATTTAT↑
TAAAAGGCAGAATG↓GGCTGGGTGGGGTGGCTCACGCCTGTAATCCCACTACTTTGGGAGTCCAAGGTGGAA
GGATCACTTGAGGTCAGGAGTTCTAGATCAGCCTGGCCAACAG

Filled site:

TTGGGGCATTCTGGACACAGATGCAGTCCTGTTTTTTCCTGCACCCTTCCCCCAGTGCTTCAGCCTTTTCAG
GACAGATGCATACTGTCATTTGTGCTCTGAAGCCTCAGTATTCTGCATGGTGCCTGATCCAGAGCACCTGCC
AGGTATTTATTGAATCAGTCTGTTTCCATTTGAAAATGCCCATGGTAAGAAAAATGTAGATGCCACTGGAAT
CTGTTGTCTTCAAGGCCTTTTGGTTAAAAGGGACAGAAACCCATCAGCTAAAGCAGAAAAGGGAGATTTATT
AAAAGGCAGAATG**GGCCGGGCGCGGTGGCTCACGCCTGTAATCCCAGCACTTTGGGAGGCCGAGGCGGGCGG**
ATCACGAGGTCAGGAGATCGAGACCATCCCGGCTAAAACGGTGAAACCCCGTCTCTACTAAAAATACAAAAA
ATTAGCCGGGCGTAGTGGCGGGCGCCTGTAGTCCCAGCTACTTGGGAGGCTGAGGCAGGAGAATGGCGTGAA
CCCGGGAGGCGGAGCTTGCAGTGAGCCGAGATCCCGCCAC... [NEOcassette] ***AAAAAAAAAAAAAAA***
AAAAAAAAAAAAAAAAAAAAAAAAAAAAAAAAAAAAAAAAAAAAAAAAAAAAAAAAAAAAAAAAAAAAAAAA
AAAAAAAAAAAAAAAAAAAAAAAAAAAAAAAAAAAAAAAAAAAAAAAAAACATTACAAAAAAAAAAAAAAAA
AAAAAAAAAAAAAAAAAAAAAAAAAAAAAAAAAAAAAAAATAAAAGGCAGAATGGGCTGGGTGGGGTGGCTCA
CGCCTGTAATCCCACTACTTTGGGAGTCCAAGGTGGAAGGATCACTTGAGGTCAGGAGTTCTAGATCAGCCT
GGCCAACAG

LTR at the 5'; Alu at the 3'.

CLONE 100
[L12]

Driver: L1
Plasmid: AlurescueA70D $A_{17}CATTACA_{18}GA_{17}CACACA_{18}$ (T)
Chromosome: 8
5’ position: 121,131,336
Strand: plus
DR: AATATATTATATATT
ENDOsite: TATT/AT

Empty site:

AAATTAGCCAAGCATCGTGGCGGGCACCTGTAATCCGAGCTACTCAGGAGGCTGAGTCAGGAGAATGGCTGG
AACCTGGGCAGTGGAGGTTGCAGTAAGCCAAGATCGCGCCACTGCACTCCAGCCTGGATGACAGAGTGAGAC
TCTCTCTCTCTCTCTCTCTCTATATATATTATATATTATATATTATATATATTATATTATATATTATATATT
ATATATTATATATTATATTATATATTATATATATTATATAATGTATTATATATTATATTTAATATATTATAT
TTTATATATATTATATTTAATATATTATATATTATATATATTATATAACGTATTATATATTATATATAATAT
ATTATATATTACATATAATATAT↑**AATATATTATATATT**↓ACTGATTAATAGACACATGGCAGTACATGCGT
GTTACTTAGAAGTATGGAAATTTTGAAAGTCTCAAAAAAAACTGCTAAGAGAGAAAAAAATACTTCTG

Filled site:

AAATTAGCCAAGCATCGTGGCGGGCACCTGTAATCCGAGCTACTCAGGAGGCTGAGTCAGGAGAATGGCTGG
AACCTGGGCAGTGGAGGTTGCAGTAAGCCAAGATCGCGCCACTGCACTCCAGCCTGGATGACAGAGTGAGAC
TCTCTCTCTCTCTCTCTCTCTATATATATTATATATTATATATTATATATATTATATTATATATTATATATT
ATATATTATATATTATATTATATATTATATATATTATATAATGTATTATATATTATATTTAATATATTATAT
TTTATATATATTATATTTAATATATTATATATTATATATATTATATAACGTATTATATATTATATATAATAT
ATTATATATTACATATAATATATAATATATTATATATT**GGCCGGGCGCGGTGGCTCACGCCTGTAATCCCAG**
CACTTTGGGAGGCCGAGGCGGGCGGATCACGAGGTCAGGAGATCGAGACCATCCTGGCTAACACGGTGAAAC
CCCGTCTCTACTAAAAAAAAAAAAAATACAAAAAATTAGCCGGGCGTGGTAGCGGGCGCCTGTAGTCCCAGC
TACTCGGGAGGCTGAGGCAGGGGAATGGCGTGAACCCGGGAGGCGGAGCTTGCAGTGAGCCGAGATCCCGCC
ACTGCACTCCA…[NEOcassette]*AAAAAAAAAAAAAAAAAAAAAAAAAAAAAACATTACAAAAAAAAA*
AAAAAAAAAAAAAAAAAAAAAAAAAAAAAAAAAAAAAAAAAAAAAAAAAAAAAAAAAAAAAAAAAAAAAAAT
ATATTATATATTACTGATTAATAGACACATGGCAGTACATGCGTGTTACTTAGAAGTATGGAAATTTTGAAA
GTCTCAAAAAAAACTGCTAAGAGAGAAAAAAATACTTCTG

Alu at the 3’.

There are several differences relative to the reference sequence in the 3’ low complexity region.

CLONE 101

[I4, I6]

Driver: L1
Plasmid: AlurescueA70D $A_{17}CATTACA_{18}GA_{17}CACACA_{18}$ (T)
Chromosome: 14
5' position: 73,813,205
Strand: plus
DR: AAAAAAAAAAAAAAAAAAAAAAA
ENDOsite: TTTT/GA

Empty site:

AGAACTCTTGAAAAAGTTCTGGGATCTCACTTTGAGAAGCACTAGATTAAGTTTCCTATGGGTAATATTTCA
TTTGTTACATTAAAATCCCTTCTCCATTAAAAAAATTATCTCTGTAGGCTGGGTGCGGTGGCTCATGCCTGT
AACCCAAGAACTTTGGGAGGCCGAGGTGAGCGGATCACCTGAGGTCGGGAGTTCGAGACCAGCTTGACCAAC
ATGGAGAAACCCCCGTCTCTACTAAAAATACAAAATTAGCTGGGTGTGGTGACACATGTCTGTAATCCCAAC
TACTCGGGAGGCTGAGGCAGGAGAATTGCTTGAACCCAGGAAGCAGAGGCTGCGGTGAACCGAGACTGCACT
ATTGCACTCCAGCCTGGGCAACAAGAGTGAAACTCTGTCTC↑**AAAAAAAAAAAAAAAAAAAAAAA**↓TTTATC
TCTGTTATACCAGCAGATAGAATTGCAAGACGAGCTCTGAAACCTACTTTAATTAATCATTACTGTTACCAA
TTATATAGATACTAATATATTT

Filled site:

AGAACTCTTGAAAAAGTTCTGGGATCTCACTTTGAGAAGCACTAGATTAAGTTTCCTATGGGTAATATTTCA
TTTGTTACATTAAAATCCCTTCTCCATTAAAAAAATTATCTCTGTAGGCTGGGTGCGGTGGCTCATGCCTGT
AACCCAAGAACTTTGGGAGGCCGAGGTGAGCGGATCACCTGAGGTCGGGAGTTCGAGACCAGCTTGACCAAC
ATGGAGAAACCCCCGTCTCTACTAAAAATACAAAATTAGCTGGGTGTGGTGACACATGTCTGTAATCCCAAC
TACTCGGGAGGCTGAGGCAGGAGAATTGCTTGAACCCAGGAAGCAGAGGCTGCGGTGAACCGAGACTGCACT
ATTGCACTCCAGCCTGGGCAACAAGAGTGAAACTCTGTCTCAAAAAAAAAAAAAAAAAAAAAAA**GGCCGGGC
GCGGTGGCTCACGCCTGTAATCCCAGCACTTTGGGAGGCCGAGGCGGGCGGATCACGAGGTCAGGAGATCGA
GACCATCCTGGCTAACACGGTGAAACCCCGTCTCTACTAAAAAAAAATACAAAAAATTAGCCGGGCGTGGTA
GCGGGCGCCTGTAGTCCCAGCTACTCGGGAGGCTGAGGCAGGGGAATGGCGTGAACCCGGGAGGCGGAGCTT
GCAGTGAGCCGAGATCCCGCCACTGCACTCCAGC... [NEOcassette]** ***AAAAAAAAAAAAAAAAAAAAAAA
AAAAAAAAAAAAAAAAACATTACAAAAAAAAAAAAAAAAAAAAAAAAAAAAAAAA***TTTATCTCTGTTATACCAGCAG
ATAGAATTGCAAGACGAGCTCTGAAACCTACTTTAATTAATCATTACTGTTACCAATTATATAGATACTAAT
ATATTT

Alu at the 5'.

CLONE 103

[N54, N16]

Driver: L1
Plasmid: AlurescueA70D $A_{17}CATTACA_{18}GA_{17}CACACA_{18}$ (T)
Chromosome: 3
5’ position: 99,590,784
Strand: minus
DR: AATAAAAAAGCAAAGGA
ENDOsite: TATT/GC

Empty site:

TGGAGGTCATCTGATCCAAATGCCTCACTTTACAGAAGAAACTGATGTCCACAGTGTGACTTGCCCCAGAGA
CACAGCTATGCAGAGGTAGGGCCAGGGCCCCCTGCATTCTTGGGCTACAGCTCACCAGTAGTTTTTCCTTAA
TTAGTGCTAGGAAGAGAGGAGAACAGAAGCCCCAGAAAAAGCCTCAGTACTGTAGGTGTTAATTTAGATATA
AAATCCATTAAAAAAAGAAAGTAGAAAGGTTCAAAGTTCTTAAGTATAAGC↑**AATAAAAAAGCAAAGGA**↓TA
AGGAAGCATTGTGAAAATCAATACAATTTAATCAGGCTACAACAACAGAAGGGAGGTGATAAAGGGAGGAAA
GAGAAAATAAAAAATAAGTGTATTTA

Filled site:

TGGAGGTCATCTGATCCAAATGCCTCACTTTACAGAAGAAACTGATGTCCACAGTGTGACTTGCCCCAGAGA
CACAGCTATGCAGAGGTAGGGCCAGGGCCCCCTGCATTCTTGGGCTACAGCTCACCAGTAGTTTTTCCTTAA
TTAGTGCTAGGAAGAGAGGAGAACAGAAGCCCCAGAAAAAGCCTCAGTACTGTAGGTGTTAATTTAGATATA
AAATCCATTAAAAAAAGAAAGTAGAAAGGTTCAAAGTTCTTAAGTATAAGCAATAAAAAAGCAAAGGA**GGCC**
GGGCGCGGTGGCTCACGCCTGTAATCCCAGCACTTTGGGAGGCCGAGGCGGGCGGATCACGAGGTCAGGAGA
TCGAGACCATCCTGGCTAACACGGTGAAACCCCGTCTCTACTAAAAAAAAAAAAAATACAAAAAATTAGCCGG
GCGTGGTAGCGGGCGCCTGTAGTCCCAGCTACTCGGGAGGCTGAGGCAGGGGAATGGCGTGAACCCGGGAGG
CGGAGCTTGCAGTGAGCCGAGATCCC… [NEOcassette] ***AAAAAAAAAAAAAAAAAAAAACATTACAA***
AAAAAAAAAAAAAAAAAAAAAAAAAAAAAAAAAAAAGAAATAAAAAAAGCAAAGGAT
AAGGAAGCATTGTGAAAATCAATACAATTTAATCAGGCTACAACAACAGAAGGGAGGTGATAAAGGGAGGAA
AGAGAAAATAAAAAATAAGTGTATTTA

MIRs at the 5’.

CLONE 105

[N36]

Driver: L1
Plasmid: AlurescueA70D $A_{17}CATTACA_{18}GA_{17}CACACA_{18}$ (T)
Chromosome: 12
5' position: 110,701,857
Strand: plus
DR: AAAAAAAAAAAAAAAGG
ENDOsite: TTTT/GA

Empty site:

AAAAGTCTGACAATTGACCGGCTGCAGTGGCTCACGCCTGTAATCCCAGCACTTTGGGAGGCCGAGGCGGGC
AGATCACTTGAGGTCAGGAGTTCGAGACCAGCCTGGCCAACATGGTGAAACCCCGTCTCTACTAAAAATACA
AAAATTAGGTGGGCTTGGTGGCGGTGCCTGTAATCCCAGCTACTTGGGAGGCTGAGGCAGGAGAATTGCTTG
AACCTGGGAGGCGGAGGTTGCAGTGAGCTGAGATTGCACCACGGCACTCCATCCTGGGCGTCAGAGTGAGAC
TCGGTCTC↑**AAAAAAAAAAAAAAAGG**↓AAAGGTAGTATGAATGTCTGGGCATTCATACCATTCTATCTTTAA
GGTAACAGAAATACTCCAAATGGGGTTTCGGGAAGAGGAGTGGCTGAGTGAAAAA

Filled site:

AAAAGTCTGACAATTGACCGGCTGCAGTGGCTCACGCCTGTAATCCCAGCACTTTGGGAGGCCGAGGCGGGC
AGATCACTTGAGGTCAGGAGTTCGAGACCAGCCTGGCCAACATGGTGAAACCCCGTCTCTACTAAAAATACA
AAAATTAGGTGGGCTTGGTGGCGGTGCCTGTAATCCCAGCTACTTGGGAGGCTGAGGCAGGAGAATTGCTTG
AACCTGGGAGGCGGAGGTTGCAGTGAGCTGAGATTGCACCACGGCACTCCATCCTGGGCGTCAGAGTGAGAC
TCGGTCTCAAAAAAAAAAAAAAAGG**GGCCGGGCGCGGTGGCTCACGCCTGTAATCCCAGCACTTTGGGAGGC**
CGAGGCGGGCGGATCACGAGGTCAGGAGATCGAGACCATCCTGGCTAACACGGTGAAACCCCGTCTCTACTA
AAAAAAAATACAAAAAATTAGCCGGGCGTGGTAGCGGGCGCCTGTAGTCCCAGCTACTCGGGAGGCTGAGGC
AGGGGAATGGCGTGAACCCGGGAGGCGGAGCTTGCAGTGAGCCGAGATCCCGCC…[NEOcassette]*AAA*
AAAAAAAAAAAAAAAAAAAAACATTACAAAAAAAAAAAAAAAAAAAAAAGAAAAAAAAAAAAAAAA
AAAAAAAAAAAAAAAAAAAAAAACACACAAAAAAAAAAAAAAAAAAAAAAAAAAAAAAAAAAAAAA
AAAAAAAAAAAAAAAAAAAAGGAAAGGTAGTATGAATGTCTGGGCATTCATACCATTCTATCTTTAAGGTAA
CAGAAATACTCCAAATGGGGTTTCGGGAAGAGGAGTGGCTGAGTGAAAAA

Alu at the 5'of the insertion site.

CLONE 106

[M13]

Driver: L1
Plasmid: AlurescueA70D $A_{17}CATTACA_{18}GA_{17}CACACA_{18}$ (T)
Chromosome: 17
5' position: 38,552,227
Strand: plus
DR: AATATTTTTAAGA
ENDOsite: TATT/AT

Empty site:

TTTGGGAGGCCGTGGTGGGCAGATCTCTTGAGGTCAGGAGTTCGAGACCAGCCTGGCCAAGATGGTGAAACC
CCATCTCTACTAAAAATACAAAAATTAGCTGGGTGTGGTGATGTGCCCCCAACTACTTGGGAGGCTGAGGCA
CGAGAATCGCTTGAATACGGGAGGCGGAGGTTGCTGTGAGCTGAGATCCTGCCATTGCACTCCAGCCTGGGT
AACACAGCGAGACTCCATCTCAACAACAACAACAAAAAAATTTTATACATTTTTTTTTCAATTTTAAGTGGA
CATATGCATCAATATTTTTATGCAT↑**AATATTTTTAAGA**↓GTATTACCTCTTTTTTTTTTTTGAGATGGAGTT
TTGCTCTGTTGGCCAGGCTGGAGTGCAGTGGTGTGACCTTGGCTCACTGCAACCTCTGCCTCCCGGG

Filled site:

TTTGGGAGGCCGTGGTGGGCAGATCTCTTGAGGTCAGGAGTTCGAGACCAGCCTGGCCAAGATGGTGAAACC
CCATCTCTACTAAAAATACAAAAATTAGCTGGGTGTGGTGATGTGCCCCCAACTACTTGGGAGGCTGAGGCA
CGAGAATCGCTTGAATACGGGAGGCGGAGGTTGCTGTGAGCTGAGATCCTGCCATTGCACTCCAGCCTGGGT
AACACAGCGAGACTCCATCTCAACAACAACAACAAAAAAATTTTATACATTTTTTTTTCAATTTTAAGTGGA
CATATGCATCAATATTTTTATGCATAATATTTTTAAGA**GGCCGGGCGCGGTGGCTCACGCCTGTAATCCCAGC**
ACTTTGGGAGGCCGAGGCGGGCGGATCACGAGGTCAGGAGATCGAGACCATCCTGGCTAACACGGTGAAACC
CCGTCTCTACTAAAAAAAAAAAATACAAAAAATTAGCCGGGCGTGGTAGCGGGCGCCTGTAGTCCCAGCTACT
CGGGAGGCTGAGGCAGGGGAATGGCGTGAACCCGGGAGGCGGAGCTTGCAGTGAGCCGAGATCCCGCCACTG
CACTCCAGC… [NEOcassette] ***AAAAAAAAAAAAAAAAAAAAAAAAAAAAAAAAAAAAAAAAAAAAAAA***
AAAAAAAAAAAAAAAAAAAAAAAAAAAAAAAAAAAAAAAAATATTTTTAAGAGTATTACCTCTTTTTTTTTT
TTGAGATGGAGTTTTGCTCTGTTGGCCAGGCTGGAGTGCAGTGGTGTGACCTTGGCTCACTGCAACCTCTGC
CTCCCGGG

Alus at the 5' and 3'.

CLONE 107

[R28, R38]

Driver: ORF2
Plasmid: AlurescueA70D $A_{17}CATTACA_{18}GA_{17}CACACA_{18}$ (T)
Chromosome: 6
5' position: 146,090,957
Strand: minus
DR: AAATTTTTTATTGTC
ENDOsite: ATTT/CT

Empty site:

CAATGATAGACTGGATTAAGAAAATGTGGCACATATACACCATGGAATACTATGCAGCCATAAAAAATGATG
AGTTCATGTCCTTTGTAGGGACATGGATGAAATTGGAAACCATCATTCTCTAG↑**AAATTTTTTATTGTC**↓GT
TTTTGTTTTTGAGAAAGAGTCTCACTCTGTCACCCAGGCTGGAGTGCAGTGGTGCAATTTCAGCTCACCACA
ATCTCTGCCTTCTGGGTTCAAGTGATTCCCTTGCCTCGGCCTCCCGAATAGCTGGGACTACAGGTGCATGCC
ACCATGCCCAGCTAATTTTTATATTTTTAGTGGAGACGGGGTTTTTCCACGTTG

Filled site:

CAATGATAGACTGGATTAAGAAAATGTGGCACATATACACCATGGAATACTATGCAGCCATAAAAAATGATG
AGTTCATGTCCTTTGTAGGGACATGGATGAAATTGGAAACCATCATTCTCTAGAAATTTTTTATTGTC**GGCC
GGGCGCGGTGGCTCACGCCTGTAATCCCAGCACTTTGGGAGGCCGAGGCGGGCGGATCACGAGGTCAGGAGA
TCGAGACCATCCTGGCTAACACGGTGAAACCCCGTCTCTACTAAAAAAAAAATACAAAAAATTAGCCGGGCG
TGGTAGCGGGCGCCTGTAGTCCCAGCTACTCGGGAGGCTGAGGCAGGGGAATGGCGTGAACCCGGGAGGCGG
AGCTTGCAGTGAGCCGAGATCCC...[NEOcassette]*AAAAAAAAAAAAAAAAAACATTACAAAAAAA
AAAAAAAAAAAAAAGAAAAAAAAAAAAAAAAAAAAAAAAAAAAAAAAAAAAAAAAAA*** TTTTTTATTGT
CGTTTTTGTTTTTGAGAAAGAGTCTCACTCTGTCACCCAGGCTGGAGTGCAGTGGTGCAATTTCAGCTCACC
ACAATCTCTGCCTTCTGGGTTCAAGTGAT

LINE-1 at the 5'; Alu at the 3'.

CLONE 108
[Brown MP#20, 24; Green MP#84, colored 14, C5, N8, colored 14]

Driver: ORF2
Plasmid: AlurescueA70Du $A_{17}CATTACA_{18}GA_{17}CACACA_{18}$ (T)
Chromosome: 6
5' position: 96,943,242 - 96,943,247
Strand: plus
DR: none
ENDOsite: n/a

Empty 5' site 96,943,077 - 96,943,247 plus strand:

GGTTGTTTGTTTTTTTCTTGTAAATTTGTTTAAGTTCTTTGTAGATTCTGGATATTAGCCCTTTGTTGGATG
GGTACTATGCAAAAATTTTCTGCCATTCTATAGGTGGCCTGTTCACTCTGATGGTAGTTTCTTTTGTTGTGC
AGAAGCTCTTTAGGTTAATTAG↑ATCCC↓

Empty 3' site 96,942,936 - 96,942,806 minus strand:

ATGAAGATCATTAAAAAGTCAGGAAACAACAGATGCTGGAGGGGATGTGGAGAAATAGGAACGCTTTTGCAC
TGTTGGTGGGAGTGTTAATTAGTTCAATCATTGTGGAAGGCAGTATGGTGATTCCTCAA

Filled site:

GGTTGTTTGTTTTTTTCTTGTAAATTTGTTTAAGTTCTTTGTAGATTCTGGATATTAGCCCTTTGTTGGATG
GGTACTATGCAAAAATTTTCTGCCATTCTATAGGTGGCCTGTTCACTCTGATGGTAGTTTCTTTTGTTGTGC
AGAAGCTCTTTAGGTTAATTAGATCCC**GGCTAAAACGGTGAAACCCCGTCTCTACTAAAAATACAAAAAATT**
AGCCGGGCGTAGTGGCGGGCGCCTGTAGTCCCAGCTACTTGGGAGGCTGAGGCAGGAGAATGGCGTGAACCC
GGGAGGCGGAGCTTGCAGTGAGCCGAGATCCCGCC... **[NEOcassette]** ***AAAAAAAAAAA***TGATGAAGATC
ATTAAAAAGTCAGGAAACAACAGATGCTGGAGGGGATGTGGAGAAATAGGAACGCTTTTGCACTGTTGGTGG
GAGTGTTAATTAGTTCAATCATTGTGGAAGGCAGTATGGTGATTCCTCAA

LINE-1 sequence flanking both the 5' and 3' regions of the insertion site.

These 5 bases are identical between the vector and reference sequence.

Alu insert is truncated, missing between 85-89 5' bp.

This 3' sequence is 311 bp away and on the opposite strand relative to the 5' reference sequence position.

Clone 108 is problematic as all of the recovered flanking genomic sequence consists of repetitive sequence and L1. The sequences flanking either side of clone 108 are on opposite strands according to the best match obtained from the current reference genomic assembly. The best match to the 5' and 3' flank both map to separate but relatively nearby locations on chromosome 6. This would support that the insertion of clone 108 is associated with an inversion. Although the recovered clone was fully sequenced, all flanking sequence recovered contained L1 sequence. Thus, we were unable to determine if this insertion may have occurred during the Alu insertion event or if it represents an existing inversion in an unannotated L1 element (not present in the human reference sequence).

CLONE 109

[I19, I25]

Driver: L1
Plasmid: AlurescueA70D $A_{17}CATTACA_{18}GA_{17}CACACA_{18}$ (T)
Chromosome: 7
5' position: 44,970,630
Strand: minus
DR: AAAAAAAAAAAAAGAAAAA
ENDOsite: TTTT/GA

Empty site:

CTGCTTTCTCAGTAAATGTTATTGAGCCAAATTGCTAGGCATCTGAGAAAATGTTTCTACTTCACACCACAT
ACCAAAATTAATCTAGGTTGATTAAAATTTTTAATGTAAAAGAAAACAGTTGGCAGGGCACAGTGGCTCGTC
TCTACTAAAAATACAAAAATTAGCCGGGCTTGGTGGCAATGCCTGTAATCCCAGCTACTCAAGAGGCTGAGG
CAGGAGAATTGCTTGAAGCCAGGAGGCGGAAGTTGTGTTTAGCCAAGATCGGCTCATCGCACTCCAGCCTGG
GCAACAAGGGCAAAACTCCGTCTC↑**AAAAAAAAAAAAAGAAAAA**↓GAAAAAGAAAACAGTTAAATAATAGAT
ATCTAAAACATCTTTATAAATGGAAGTTTTTTTAAGCATAGAGCAGGGCATAAAATAGAAAAATGCATGTGT
G

Filled site:

CTGCTTTCTCAGTAAATGTTATTGAGCCAAATTGCTAGGCATCTGAGAAAATGTTTCTACTTCACACCACAT
ACCAAAATTAATCTAGGTTGATTAAAATTTTTAATGTAAAAGAAAACAGTTGGCAGGGCACAGTGGCTCGTC
TCTACTAAAAATACAAAAATTAGCCGGGCTTGGTGGCAATGCCTGTAATCCCAGCTACTCAAGAGGCTGAGG
CAGGAGAATTGCTTGAAGCCAGGAGGCGGAAGTTGTGTTTAGCCAAGATCGGCTCATCGCACTCCAGCCTGG
GCAACAAGGGCAAAACTCCGTCTCAAAAAAAAAAAAAGAAAAA**GGCCGGGCGCGGTGGCTCACGCCTGTAAT
CCCAGCACTTTGGGAGGCCGAGGCGGGCGGATCACGAGGTCAGGAGATCGAGACCATCCTGGCTAACACGGT
GAAACCCCGTCTCTACTAAAAAAAAATACAAAAAATTAGCCGGGCGTGGTAGCGGGCGCCTGTAGTCCCAGC
TACTCGGGAGGCTGAGGCAGGGGAATGGCGTGAACCCGGGAGGCGGAGCTTGCAGTGAGCCGAGATCCCGCC
AC... [NEOcassette] *AAAAAAAAAAAAAAAAAAAAACATTACAAAAAAAAAAAAAA***GAAAAAGAAAAA
GAAAACAGTTAAATAATAGATATCTAAAACATCTTTATAAATGGAAGTTTTTTTAAGCATAGAGCAGGGCAT
AAAATAGAAAAATGCATGTGTG

LINE-1 and Alu at the 5'of the insertion site.

CLONE 110

[Q13, R30]

Driver: L1
Plasmid: AlurescueA70D $A_{17}CATTACA_{18}GA_{17}CACACA_{18}$ (T)
Chromosome: 22
5' position: 31,879,121
Strand: plus
DR: AACAGAATAGCAGGTAC
ENDOsite: TGTT/AG

Empty site:

CAGAGCACTTTAGGAGGCCAAGGTGGGTGGATCATGAGATCAGGAGATCGAGACCATCCTGGCCAACATGGT
GAAACCCCATCTCTACTAAAATGTAAAAAAAATTAGCTGGGCATGGAGGCACGCGCCTGTAGTCCCAGCTAC
TCAAGAGGCTGAGGTAGGGAAATTGCTTGAACCCGGGAGGCAGAGGTTGCAGTGAGCCGACATAGGGCCACT
GCACTCCAGCCTGGCGACAGACAGAGACTCTGTCTCAAAACAAAAAAACAGAAAAGAAAACCACATGGATCT
GAATAGTGGGATGGCCTAAAACATTTCTATTTTGTATTTCAAGCAGAAGATGGCATCACT↑**AACAGAATAGC**
AGGTAC↓TATATTCCAGAACTGGTGAACAAGGGCTTCTGGTGAAGAAAAAGTTGAATGAGCACCACTGCCAG
GAGACGATCTTTTTTTTTTTTTTTTTTTTTTGAGAACAGGGTCTCTTTTTGTTGCACAGGCTGTAGTGAAAT
GGCGCGATGATGGCTCACTGCAGCCTCAAACGATTCTCTCACCTCAGTCCCCTGTGTAGCTGAG

Filled site:

CAGAGCACTTTAGGAGGCCAAGGTGGGTGGATCATGAGATCAGGAGATCGAGACCATCCTGGCCAACATGGT
GAAACCCCATCTCTACTAAAATGTAAAAAAAATTAGCTGGGCATGGAGGCACGCGCCTGTAGTCCCAGCTAC
TCAAGAGGCTGAGGTAGGGAAATTGCTTGAACCCGGGAGGCAGAGGTTGCAGTGAGCCGACATAGGGCCACT
GCACTCCAGCCTGGCGACAGACAGAGACTCTGTCTCAAAACAAAAAAACAGAAAAGAAAACCACATGGATCT
GAATAGTGGGATGGCCTAAAACATTTCTATTTTGTATTTCAAGCAGAAGATGGCATCACTAACAGAATAGCA
GGTAC**GGCCGGGCGCGGTGGCTCACGCCTGTAATCCCAGCACTTTGGGAGGCCGAGGCGGGCGGATCACGAG**
GTCAGGAGATCGAGACCATCCTGGCTAACACGGTGAAACCCCGTCTCTACTAAAAAAAAATACAAAAAATTA
GCCGGGCGTGGTAGCGGGCGCCTGTAGTCCCAGCTACTCGGGAGGCTGAGGCAGGGGAATGGCGTGAACCCG
GGAGGCGGAGCTTGCAGTGAGCCGAGATCCC…[NEOcassette]***AAAAAAAAAAAAAAAAACATTAC***
AAAAAAAAAAAAAAAAAAAAAGAAAAAAAAAAAAAAAAAAAAAAAAAAAAAAAAAAAAAAAAAAAAAAAAAAAA
AAAAAAAAAAAAAAAAAAAAAAAACAGAATAGCAGGTACTATATTCCAGAACTGGTGAACAAGGGCTTCTGG
TGAAGAAAAAGTTGAATGAGCACCACTGCCAGGAGACGATCTTTTTTTTTTTTTTTTTTTTTTGAGA

Two different Alus on each side.

CLONE 119

[JK2,JK3,JK5,JK13,JK18,JK26, K8, K38, P54]

Driver: ORF2
Plasmid: AlurescueA70D $A_{17}CATTACA_{18}GA_{17}CACACA_{18}$ (T)
Chromosome: 12
5' position: 20,058,768
Strand: plus
DR: AAGAAGAGAGAT
ENDOsite: TCTT/GA

Empty site:

TTATAATCCAGTTGGGGATAGCAGATATATAGAAATGATATAATCTGAACATGACATAAAAAGAAGGTAATA
CAGGTAATCACAAGATCATGAACAATTAATGGCCAAATATATTATTTAAACAATAAGTGCTATGAAGGCAAC
ACAAGGAAGTGTCACTGAATTCTTAGTAATACAGAAAACAAAAAAAAAATTAAGAAAGAGTATGAGCTGGGC
ATATTACTGGTTGTTCAATGACTGCCTTTTTTGTTAAAAAGGAGGAGAAAGAGAGAAAGTGGAAATGTATAG
TTATC↑**AAGAAGAGAGAT**↓AAGCAGAACCTCACTTGTTCCCTTAACCAGGCTATGAGTATCTCTAGGGCAGA
GACAGTATTATATTCATAGCTCATTTACAAGTAGTTAGAAAGCATAT

Filled site:

TTATAATCCAGTTGGGGATAGCAGATATATAGAAATGATATAATCTGAACATGACATAAAAAGAAGGTAATA
CAGGTAATCACAAGATCATGAACAATTAATGGCCAAATATATTATTTAAACAATAAGTGCTATGAAGGCAAC
ACAAGGAAGTGTCACTGAATTCTTAGTAATACAGAAAACAAAAAAAAAATTAAGAAAGAGTATGAGCTGGGC
ATATTACTGGTTGTTCAATGACTGCCTTTTTTGTTAAAAAGGAGGAGAAAGAGAGAAAGTGGAAATGTATAG
TTATCAAGAAGAGAGAT**GGCCGGGCGCGGTGGCTCACGCCTGTAATCCCAGCACTTTGGGAGGCCGAGGCGG**
GCGGATCACGAGGTCAGGAGATCGAGACCATCCTGGCTAACACGGTGAAACCCCGTCTCTACTAAAAAAAAA
AATACAAAAAATTAGCCGGGCGTGGTAGCGGGCGCCTGTAGTCCCAGCTACTCGGGAGGCTGAGGCAGGGGA
ATGGCGTGAACCCGGGAGGCGGAGCTTGCAGTGAGCCGAGATCCCGCCACTGCACTCCAGCCT… [NEOcas
sette]*AAAAAAAAAAAAAAAAACATTACAAAAAAAAAAAAAAAAAAGAAAAAAAAAAAAAA*
AAAAAAAAAAACACACAAAAAAAAAAAAAAAAAAAGAAGAGAGATAAGCAGAACCTCACTTG
TTCCCTTAACCAGGCTATGAGTATCTCTAGGGCAGAGACAGTATTATATTCATAGCTCATTTACAAGTAGTT
AGAAAGCATAT

LINE-1 at the 3'.

CLONE 120

[Q53, Q39b]

Driver: L1
Plasmid: AlurescueA70D $A_{17}CATTACA_{18}GA_{17}CACACA_{18}$ (T)
Chromosome: 5
5’ position: 26,453,190
Strand: minus
DR: AAAACATTTTTTA
ENDOsite: TTTT/AT

Empty site:

ATTTTTGGGCACTTGCTTCAGTTGCAAAATAAAATAACAAAATATTCCTGTGATGTATATTCAAGAAAACTG
AAACCTTAAGTTAACTTAAGCATTTAATTCTATAAAACTACCATTGTACAAAAGACAACAAATCGATAAATG
ATGTGGACAGCTTTTGTTGCAGATATCTGATTTCAAAATGCTTCAGCAAAAATAAAATGTATTTAATTTTGT
ATTTATATAAATAAATCTATCCAATTGGCAAAATGACACTTCCAAATTATGTAGAT↑**AAAACATTTTTTA**↓A
AGAAATATTTGGCCTTAGCAATATCCCAGGATTTTATGAATCTGTTTAATAGTAAAGAATCATGATATGATA
TACAGTAACAGGCTGTGGATGAACAGT

Filled site: …**[NEOcassette]**

ATTTTTGGGCACTTGCTTCAGTTGCAAAATAAAATAACAAAATATTCCTGTGATGTATATTCAAGAAAACTG
AAACCTTAAGTTAACTTAAGCATTTAATTCTATAAAACTACCATTGTACAAAAGACAACAAATCGATAAATG
ATGTGGACAGCTTTTGTTGCAGATATCTGATTTCAAAATGCTTCAGCAAAAATAAAATGTATTTAATTTTGT
ATTTATATAAATAAATCTATCCAATTGGCAAAATGACACTTCCAAATTATGTAGATAAAACATTTTTTA**GGC**
CGGGCGCGGTGGCTCACGCCTGTAATCCCAGCACTTTGGGAGGCCGAGGCGGGCGGATCACGAGGTCAGGAG
ATCGAGACCATCCTGGCTAACACGGTGAAACCCCGTCTCTACTAAAAAAAAAAAAATACAAAAAATTAGCCGG
GCGTGGTAGCGGGCGCCTGTAGTCCCAGCTACTCGGGAGGCTGAGGCAGGGGAATGGCGTGAACCCGGGAGG
CGGAGCTTGCAGTGAGCCGAGATCCCGCCACTGCACTCCAGCCT…[NEOcassette]***AAAAAAAAAAAA***
AAAAAAAAAAAAAAAACATTACAAAAAAAAAAAAAAAAAAAAAAAAAAAAAAAAAAAAAAGAAAAAAAAAAA
AAAAAAAAAAAAAAAAAAAAAAAAAAAAAAAAAAAAACATTTTTTAAAGAAATATTTGGCCTTAGCAATATC
CCAGGATTTTATGAATCTGTTTAATAGTAAAGAATCATGATATGATATACAGTAACAGGCTGTGGATGAACA
GT

No repetitive sequences in the immediate proximity.

CLONE 121

[V1, V6, V9]

Driver: L1
Plasmid: Alurescue A30D $A_{10}CTA_{10}TACA_{10}$
Chromosome: 9
5' position: 137,949,426
Strand: plus
DR: AAAAAATAAAAACA
ENDOsite: TTTT/AA

Empty site:

ACCCTAAACTTACCCAGAAAAAAAATGAGGAAGAACAAAAATGTCAATATCAGGAATAAAAGAATGAATGTGA
TTTCAGATCTTATAGATGTTAAAAAGATGAGGAATTATTATGAATAACAATTTGCAAACAAATTCATTGACA
TTAAAAATATAAGAGAATATCATGAACACCTTTATGCCAACAAATGTGACAACTTTGTTAAAAGGAGGAAAA
GCCTTGAAAAATGCAACCTACCAAAATTTACATTATGTGAAAGAGAAAATCTGAATATCTATATAGCTATTT
TAAAAATGTTTTTTT↑**AAAAAATAAAAACA**↓AATTTCGCCAAGAAAATTTCAGGCCTGGATGGTTTCACTTG
TGAATTCCGAGCACATTTCAAGAAGAAATAACACCGAATATACACAAACCCTTTCAGAA

Filled site:

ACCCTAAACTTACCCAGAAAAAAAATGAGGAAGAACAAAAATGTCAATATCAGGAATAAAAGAATGAATGTGA
TTTCAGATCTTATAGATGTTAAAAAGATGAGGAATTATTATGAATAACAATTTGCAAACAAATTCATTGACA
TTAAAAATATAAGAGAATATCATGAACACCTTTATGCCAACAAATGTGACAACTTTGTTAAAAGGAGGAAAA
GCCTTGAAAAATGCAACCTACCAAAATTTACATTATGTGAAAGAGAAAATCTGAATATCTATATAGCTATTT
TAAAAATGTTTTTTTAAAAAATAAAAACA**GGCCGGGCGCGGTGGCTCACGCCTGTAATCCCAGCACTTTGGG**
AGGCCGAGGCGGGCGGATCACGAGGTCAGGAGATCGAGACCATCCTGGCTAACACGGTGAAACCCCGTCTCT
ACTAAAAAAAAATACAAAAAATTAGCCGGGCGTGGTAGCGGGCGCCTGTAGTCCCAGCTACTCGGGAGGCTG
AGGCAGGGGAATGGCGTGAACCCGGGAGGCGGAGCTTGCAGTGAGCCGAGATCCCGCCACTGCACTCCAGCC
TG... [NEOcassette] ***AAAAAAAAAAACTAAAAAAAAAAATACAAAAAAAAAAAAAAAAAAA***TAAAAAC
AAATTTCGCCAAGAAAATTTCAGGCCTGGATGGTTTCACTTGTGAATTCCGAGCACATTTCAAGAAGAAATA
ACACCGAATATACACAAACCCTTTCAGAA

LINE-1 flanking the insertion site.

CLONE 122

[V10]

Driver: L1
Plasmid: Alurescue A30D $A_{10}CTA_{10}TACA_{10}$
Chromosome: 15
5' position: 35,143,814
Strand: minus
DR: AAGAATATTTAACAC
ENDOsite: TCTT/AT

Empty site:

ACATTTTAATTTTAGACTCCAGAATTTCACACTAAGTTTTTCTTCTCATTTCTTATGCAGTAAAAGAAGAGT
TTGTATTTCCAATGTGTCTGGCTTTCTGTTCTTTAGTTTTTGTTTCCTTAAAAAGTTTAAACTCCAAAAAGA
GAAAAGGATTACTTAGCATAACCAATAGAGGTGGTCTGGAAATAATAAAAGAAAATGAAGGCAAATATAAAA
CATGAAGACAGTTTCCTCGCAT↑**AAGAATATTTAACAC**↓ACAAGGACCCCAGCAGTTACTTTGCATTAAGCA
TACTGAATTCTTTCTTGATCTCCAGAATAACTGTCCCCACAATCCTCTTCCCCTCTACCTTTGATTT

Filled site:

ACATTTTAATTTTAGACTCCAGAATTTCACACTAAGTTTTTCTTCTCATTTCTTATGCAGTAAAAGAAGAGT
TTGTATTTCCAATGTGTCTGGCTTTCTGTTCTTTAGTTTTTGTTTCCTTAAAAAGTTTAAACTCCAAAAAGA
GAAAAGGATTACTTAGCATAACCAATAGAGGTGGTCTGGAAATAATAAAAGAAAATGAAGGCAAATATAAAA
CATGAAGACAGTTTCCTCGCATAAGAATATTTAACAC**GGCCGGGCGCGGTGGCTCACGCCTGTAATCCCAGC**
ACTTTGGGAGGCCGAGGCGGGCGGATCACGAGGTCAGGAGATCGAGACCATCCTGGCTAACACGGTGAAACC
CCGTCTCTACTAAAAAAAAAAATACAAAAAATTAGCCGGGCGTGGTAGCGGGCGCCTGTAGTCCCAGCTACTC
GGGAGGCTGAGGCAGGGGAATGGCGTGAACCCGGGAGGCGGAGCTTGCAGTGAGCCGAGATCCCGCCACT... [
NEOcassette]***AAAAAAAAAAAACTAAAAAAAAAAATACAAAAAAAAAAAAAATTAAAAAAAAAAAA***
AAAAAAAAAAAAAGAATATTTAACACACAAGGACCCCAGCAGTTACTTTGCATTAAGCATACTGAATTCTT
TCTTGATCTCCAGAATAACTGTCCCCACAATCCTCTTCCCCTCTACCTTTGATTT

No repetitive sequences in the immediate proximity.

CLONE 123

[W1]

Driver: ORF2
Plasmid: Alurescue A30D $A_{10}CTA_{10}TACA_{10}$
Chromosome: 10
5’ position: 46,986,668
Strand: minus
DR: AGAAATCAGCCATCAT
ENDOsite: TTCT/GC

Empty site:

TCAGACCTAAAAAAAAAAACTATTGCTTGTTAAAAACTCCATATTTGATAGCTTGATTCTTATATCTTGAAA
GATTTTTAAAGTAGAGTGTAAATATAACTGATGTTATGAAATTCTGTTTCATGTTCTGTAGAGCAGTTATAT
ATCATAATGTAGGCAGAAATCAGCCATCATATCATATCATATCATATCATATCATATCATATCATATCACAT
CATAATGTAGGCAGAAATCCGCCATCATATAATTGTGAGC↑**AGAAATCAGCCATCAT**↓ATAATTGTGAGCAG
AAAAATACCTAATTGGATTCTGTAAATGGGTATATTTTGAATTATGCTGTACAGTGCTTGCCCAAAAAAATA
CTGTGCCTGAAAAT

Filled site:

TCAGACCTAAAAAAAAAAACTATTGCTTGTTAAAAACTCCATATTTGATAGCTTGATTCTTATATCTTGAAA
GATTTTTAAAGTAGAGTGTAAATATAACTGATGTTATGAAATTCTGTTTCATGTTCTGTAGAGCAGTTATAT
ATCATAATGTAGGCAGAAATCAGCCATCATATCATATCATATCATATCATATCATATCATATCATATCACAT
CATAATGTAGGCAGAAATCCGCCATCATATAATTGTGAGCAGAAATCAGCCATCAT**GGCCGGGCGCGGTGGC**
TCACGCCTGTAATCCCAGCACTTTGGGAGGCCGAGGCGGGCGGATCACGAGGTCAGGAGATCGAGACCATCC
TGGCTAACACGGTGAAACCCCGTCTCTACTAAAAAAAAAAATACAAAAAATTAGCCGGGCGTGGTAGCGGGC
GCCTGTAGTCCCAGCTACTCGGGAGGCTGAGGCAGGGGAATGGCGTGAACCCGGGAGGCGGAGCTTGCAGTG
AGCCGAGATCCCGCCACT… [NEOcassette] ***AAAAAAAAAAAAAAAAAAAAAAAAAAAAAAAAAAA***
AAAAAAAAAAAAAAAAAAAAAAAAAAAAAAAAAAAAAAAAAAAAAAAAAAAAAAAAAAAAAAAAAAAAAAGA
AATCAGCCATCATATAATTGTGAGCAGAAAAATACCTAATTGGATTCTGTAAATGGGTATATTTTGAATTAT
GCTGTACAGTGCTTGCCCAAAAAAATACTGTGCCTGAAAAT

No repetitive sequences in the immediate proximity.

CLONE 124

[X5, X6, X7]

Driver: ORF2
Plasmid: Alurescue A30D $A_{10}CTA_{10}TACA_{10}$
Chromosome: 15
5' position: 31,259,673
Strand: plus
DR: AAACTCAGTATTCT
ENDOsite: GTTT/CT

Empty site:

ACAATGGGGCTAACAGGAGACACGGCTCTTTGTTTTTCTCTATCAAGATATTTTTAAAAATTGTTTATTAAA
ATTACATTTGTACGAAACAGAATGTACAGAATTGCATGATCATCTGGAATCTTCCCTGGTCATGTCATGACT
GTCAGATATATAGACAAAACTATTTACTTTCACAAAAGCTGTCTAGAATATACTTGTCCTTGGAAAAATGCT
CATCTTCCTTTCATCAGTATTACTCAGAAATAG↑**AAACTCAGTATTCT**↓GAGAATCTTAATGAAGTCCACAA
AAAGTTTTTAATCACTTTAGCTTTTGCATGAAATCACACCTTGCCAGAAAAGATTATGCTTTTAATATATCT
TTTAG

Filled site:

ACAATGGGGCTAACAGGAGACACGGCTCTTTGTTTTTCTCTATCAAGATATTTTTAAAAATTGTTTATTAAA
ATTACATTTGTACGAAACAGAATGTACAGAATTGCATGATCATCTGGAATCTTCCCTGGTCATGTCATGACT
GTCAGATATATAGACAAAACTATTTACTTTCACAAAAGCTGTCTAGAATATACTTGTCCTTGGAAAAATGCT
CATCTTCCTTTCATCAGTATTACTCAGAAATAGAAACTCAGTATTCT**GGCCGGGCGCGGTGGCTCACGCCTG**
TAATCCCAGCACTTTGGGAGGCCGAGGCGGGCGGATCACGAGGTCAGGAGATCGAGACCATCCTGGCTAACA
CGGTGAAACCCCGTCTCTACTAAAAAAAAATACAAAAAATTAGCCGGGCGTGGTAGCGGGCGCCTGTAGTCC
CAGCTACTCGGGAGGCTGAGGCAGGGGAATGGCGTGAACCCGGGAGGCGGAGCTTGCAGTGAGCCGAGATCC
CGCCACTGCACTCCAGCCTG... [NEOcassette] ***AAAAAAAAAACTAAAAAAAAAAAAATACAAAAAAA***
AAAAAAAAAAAAAAAAAAAAAAAAAAAAAAAAAAAAAAAACTCAGTATTCTGAGAATCTTAATGAAGTCCAC
AAAAAGTTTTTAATCACTTTAGCTTTTGCATGAAATCACACCTTGCCAGAAAAGATTATGCTTTTAATATAT
CTTTTAG

No repetitive sequences in the immediate proximity.

CLONE 125

[X1]

Driver: ORF2
Plasmid: Alurescue A30D $A_{10}CTA_{10}TACA_{10}$
Chromosome: 10
5' position: 121,635,083
Strand: plus
DR: AAGAAATGGGAGCCTTC
ENDOsite: TCTT/GT

Empty site:

TTCATGGCCAGTAACAGGCAAATTAAATGTATGATATGGAAATGTCTGCTCTTAAATCCCTGTAAATTCAAA
TTTACTTTATCTAAATTGCAGCTTGATACCAATTCAGCAGTGGCTAGTGTAAGGGAGGAAAAATATCTTTTC
CGTCTACCCTTCTAGATTCTACGTTGGACCCCCTGTAACAAAAGATAGATTAACAAGAGAAAAGCATGCACT
TTATTTCATACACGTTTTAC↑**AAGAAATGGGAGCCTTC**↓ATAAGGAAATGAAGATCCAAAGAAGCGTTAAAA
CCAAGTGTTTTTATGCTAGGTTTGATGAAGAGAGTCATAGAAGAATGTGATAGGACAAAGGTTGTGA

Filled site:

TTCATGGCCAGTAACAGGCAAATTAAATGTATGATATGGAAATGTCTGCTCTTAAATCCCTGTAAATTCAAA
TTTACTTTATCTAAATTGCAGCTTGATACCAATTCAGCAGTGGCTAGTGTAAGGGAGGAAAAATATCTTTTC
CGTCTACCCTTCTAGATTCTACGTTGGACCCCCTGTAACAAAAGATAGATTAACAAGAGAAAAGCATGCACT
TTATTTCATACACGTTTTACAAGAAATGGGAGCCTTC**GGCCGGGCGCGGTGGCTCACGCCTGTAATCCCAGC**
ACTTTGGGAGGCCGAGGCGGGCGGATCACGAGGTCAGGAGATCGAGACCATCCTGGCTAACACGGTGAAACC
CCGTCTCTACTAAAAAAAAAATACAAAAAATTAGCCGGGCGTGGTAGCGGGCGCCTGTAGTCCCAGCTACTC
GGGAGGCTGAGGCAGGGGAATGGCGTGAACCCGGGAGGCGGAGCTTGCAGTGAGCCGAGATCCCGCCACTGC
ACTCCAGCCTG...[NEOcassette]*AAAAAAAAAAACTAAAAAAAAAAAAAAAAAAAAAAAAAAAAAA*
AAAAAAAAAAAAAAAAAAAAAAAAAAAAAAAAAAAAAAAAAAAAAAAAAAAAAAAAAAAAAAAAAAAAAA
AAAAAAGAAATGGGAGCCTTCATAAGGAAATGAAGATCCAAAGAAGCGTTAAAACCAAGTGTTTTTATGCTA
GGTTTGATGAAGAGAGTCATAGAAGAATGTGATAGGACAAAGGTTGTGA

LTR flanking the insertion site.

CLONE 126

[X4, X15, X16]

Driver: ORF2
Plasmid: Alurescue A30D $A_{10}CTA_{10}TACA_{10}$
Chromosome: 1
5' position: 145,825,741
Strand: minus
DR: AAAAACTTGTTT
ENDOsite: TTTT/GA

Empty site:

TGGCCAATATTTGTGAAGCTCAGACTGCCTGTTGACCTTCTTCCCCCCATCTTTCCAATAAAGATTTTGTGT
GTGTGTGGTTGTTTTTACTTGTTCTTTCTCTCAAACGGCAAATAACAGAAGCAGTTACCTTTGGGGGCTTAA
AGTGAACTAAATTTGTGTTCTTTGATTCCGTTTTTGTATTGCTTCATCCTAATACTCGTCTCCGTAGAGCGG
AGCATTGTCCTTTTCTCTTC↑**AAAAACTTGTTT**↓ACAATATTTAAGAAAATAAGAAAGAAGGAAAAGCCATC
CATAATCCTACCACTCTAATGCAATAGTTTTTTCATTTTTAAAATTTACCACCTTTTCTATA

Filled site:

TGGCCAATATTTGTGAAGCTCAGACTGCCTGTTGACCTTCTTCCCCCCATCTTTCCAATAAAGATTTTGTGT
GTGTGTGGTTGTTTTTACTTGTTCTTTCTCTCAAACGGCAAATAACAGAAGCAGTTACCTTTGGGGGCTTAA
AGTGAACTAAATTTGTGTTCTTTGATTCCGTTTTTGTATTGCTTCATCCTAATACTCGTCTCCGTAGAGCGG
AGCATTGTCCTTTTCTCTTCAAAAACTTGTTT**GGCCGGGCGCGGTGGCTCACGCCTGTAATCCCAGCACTTT**
GGGAGGCCGAGGCGGGCGGATCACGAGGTCAGGAGATCGAGACCATCCTGGCTAACACGGTGAAACCCCGTC
TCTACTAAAAAAAAAAATACAAAAAATTAGCCGGGCGTGGTAGCGGGCGCCTGTAGTCCCAGCTACTCGGGAG
GCTGAGGCAGGGGAATGGCGTGAACCCGGGAGGCGGAGCTTGCAGTGAGCCGAGATCCCGCCACTGCACTCC
AGCCTG... [NEOcassette] ***AAAAAAAAAACTAAAAAAAAAAAATACAAAAAAAAAAAAAAAAAAAAAA***
AAAAAAAAAAAAAAAAAAAAAAAAAAAAAAAAAAAAAACTTGTTTACAATATTTAAGAAAATAAGAAAGAAG
GAAAAGCCATCCATAATCCTACCACTCTAATGCAATAGTTTTTTCATTTTTAAAATTTACCACCTTTTCTAT
A

No repetitive sequences in the immediate proximity.

CLONE 127

[Y2, Y12, Y16]

Driver: L1
Plasmid: Alurescue A30D $A_{10}CTA_{10}TACA_{10}$
Chromosome: 16
5' position: 88,733,379
Strand: minus
DR: AAAAAAAAAAAAAAAAA
ENDOsite: TTTT/AA

Empty site:

TTTTTTTGTAGGCTGGATGCGGTGGCTCATGTCTGTAATCCCAGCACTTTGGGAGGCCTAGGCGGGTGGATC
ACGAGGTCAGGAGATCGAGACCATGACCATCCTGGCTAACATGGTGAAACCCCGTCTCTACTAAAAATACAA
AAAAATTAGCTGGGTGTGGTGGTACACACCTGGAGTCCCAACTACTTGGGAGGCTGAGGCAGGAGAATCACT
TGAGCCTGGGAGGCGGAGGTTGCAGTGAGCCTGGGAGGCGGACGTTGCAGTGAGCCTGGGAGGCGGAGGTTG
CAGTGAGCCGAGATTGCACCACTGCACTCCAGCCTGGGCAATGGAACAAGACTGTGTCTT↑**AAAAAAAAAAA**
AAAAAA↓AAATTTTTTTTTTGTGGATATGGTGGAGTCTCGCTATGTTGCCCAGACTGGTCTTGAACTCCAGG
CCTCAAGCAGTCCTCCCAAATCCCAAAGTGCTGCTGGGCTTACAGGCATGGACCACCATGCTTGGCCTATTT
TCGACACACATACAGGCTGAGGTTCAGGAGAGCTGGGCTACAGGCGTCAGGGTGGGGATCGAT

Filled site:

TTTTTTTGTAGGCTGGATGCGGTGGCTCATGTCTGTAATCCCAGCACTTTGGGAGGCCTAGGCGGGTGGATC
ACGAGGTCAGGAGATCGAGACCATGACCATCCTGGCTAACATGGTGAAACCCCGTCTCTACTAAAAATACAA
AAAAATTAGCTGGGTGTGGTGGTACACACCTGGAGTCCCAACTACTTGGGAGGCTGAGGCAGGAGAATCACT
TGAGCCTGGGAGGCGGAGGTTGCAGTGAGCCTGGGAGGCGGACGTTGCAGTGAGCCTGGGAGGCGGAGGTTG
CAGTGAGCCGAGATTGCACCACTGCACTCCAGCCTGGGCAATGGAACAAGACTGTGTCTTAAAAAAAAAAAA
AAAAA**GGCCGGGCGCGGTGGCTCACGCCTGTAATCCCAGCACTTTGGGAGGCCGAGGCGGGCGGATCACGAG**
GTCAGGAGATCGAGACCATCCTGGCTAACACGGTGAAACCCCGTCTCTACTAAAAAAAAAATACAAAAAATT
AGCCGGGCGTGGTAGCGGGCGCCTGTAGTCCCAGCTACTCGGGAGGCTGAGGCAGGGGAATGGCGTGAACCC
GGGAGGCGGAGCTTGCAGTGAGCCGAGATCCCGCCACTGCACTCCAGCCTG... [NEOcassette]***AAAAA***
AAAAAAAAAACTAAAAAAAAAATACAAAAAAAAAAAAAAAAAAAAAAAAAAAAAAAAAAAAAAAA
AAAAAAAAAAAAAAAAAAAAAATTTTTTTTTTGTGGATATGGTGGAGTCTCGCTATGTTGCCCAGACTGGTC
TTGAACTCCAGGCCTCAAGCAGTCCTCCCAAATCCCAAAGTGCTGCT

Alu FLAMC at the 3' of the insertion site and two Alus at the 5'the insertion site.

CLONE 128

[Y11, Y20]

Driver: L1
Plasmid: Alurescue A30D $A_{10}CTA_{10}TACA_{10}$
Chromosome: 7
5’ position: 123,903,007
Strand: plus
DR: AAAAATGATAGTA
ENDOsite: TTTT/AA

Empty site:

AAAGGTGCTGTGACATCATAAACCTTGCAATAAGAAGGATAGTATTGAAATTTTAAAGTTTTTGTTCTTTTA
TTTCATTAATCCCCTATAATACAATATTATTCACATTTTTACTTGGAAAAAAGGAATCTGAAAGTTAAATGT
CCACAGGATCGGTTTTCAGATGATATGAGTTTTTCAGTTAATGTACTAACCTCAATTATGAAGGTTTATTAT
TATTATTATTGCTGCTAAAGTTAGAATAAAAGAAAAGCTAAATTTTCGACACAGAGGAGGGGAAAAAAGGTG
TTTCAAAAAGTTTCATT↑**AAAAATGATAGTA**↓ATGTTCTTTTAAAATTATTTTTAAAACAAAAGCAGCATGG
CATTTGGAAACAAAAGGCAATATTGATGATATTACAATCTGCCTTAGATATTTGGCAGAC

Filled site:

AAAGGTGCTGTGACATCATAAACCTTGCAATAAGAAGGATAGTATTGAAATTTTAAAGTTTTTGTTCTTTTA
TTTCATTAATCCCCTATAATACAATATTATTCACATTTTTACTTGGAAAAAAGGAATCTGAAAGTTAAATGT
CCACAGGATCGGTTTTCAGATGATATGAGTTTTTCAGTTAATGTACTAACCTCAATTATGAAGGTTTATTAT
TATTATTATTGCTGCTAAAGTTAGAATAAAAGAAAAGCTAAATTTTCGACACAGAGGAGGGGAAAAAAGGTG
TTTCAAAAAGTTTCATTAAAAATGATAGTA**GGCCGGGCGCGGTGGCTCACGCCTGTAATCCCAGCACTTTGG
GAGGCCGAGGCGGGCGGATCACGAGGTCAGGAGATCGAGACCATCCTGGCTAACACGGTGAAACCCCGTCTC
TACTAAAAAAAAATACAAAAAATTAGCCGGGCGTGGTAGCGGGCGCCTGTAGTCCCAGCTACTCGGGAGGCT
GAGGCAGGGGAATGGCGTGAACCCGGGAGGCGGAGCTTGCAGTGAGCCGAGATCCCGCCACTGCACTCCAGC
CT... [NEOcassette]** ***AAAAAAAAAAACTAAAAAAAAAAAAAAAAAAAAAAAATACAAAAAAAAAAA
AAAAA***TGATAGTAATGTTCTTTTAAAATTATTTTTAAAACAAAAGCAGCATGGCATTTGGAAACAAAAGGCA
ATATTGATGATATTACAATCTGCCTTAGATATTTGGCAGAC

No repetitive sequences in the immediate proximity.

CLONE 129

[Z1]

Driver: ORF2
Plasmid: AlurescueA70D $A_{17}CATTACA_{18}GA_{17}CACACA_{18}$ (T)
Chromosome: 18
5' position: 54,213,642
Strand: plus
DR: AAAACAGCAAAAG
ENDOsite: TTTT/AA

Empty site:

AAAATTCTGATGAACAAAAATTTAGCAAATACCTCTTCATAGATATATAACATAGATATCTATTTACAAATA
TATAGTATGTGCAAAATTTTAAACTAGCATACTTTGGACTCAGTAATTCCACTTATGGAAATTTAAACTACA
AATATACCACATATGTACAAAATTAGATATCTAAGTAGATACTCACTGCAACATGGTTT↑**AAAACAGCAAAA**
G↓ACTGGAAACCACTTAAGAAAGCCATCCATGGGGCCCGTAAATAATAAATTTAAAGAATTTATTTAGATAA
TATGCTTTTGAAACCATTCCAATATAACTT

Filled site:

AAAATTCTGATGAACAAAAATTTAGCAAATACCTCTTCATAGATATATAACATAGATATCTATTTACAAATA
TATAGTATGTGCAAAATTTTAAACTAGCATACTTTGGACTCAGTAATTCCACTTATGGAAATTTAAACTACA
AATATACCACATATGTACAAAATTAGATATCTAAGTAGATACTCACTGCAACATGGTTTAAAACAGCAAAAG
GGCCGGGCGCGGTGGCTCACGCCTGTAATCCCAGCACTTTGGGAGGCCGAGGCGGGCGGATCACGAGGTCAG
GAGATCGAGACCATCCTGGCTAACACGGTGAAACCCCGTCTCTACTAAAAAAAAAATACAAAAAATTAGCCG
GGCGTGGTAGCGGGCGCCTGTAGTCCCAGCTACTCGGGAGGCTGAGGCAGGAAATGGCGTGAACCCGGGAG
GCGGAGCTTGCAGTGAGCCGAGATCCCGCCACTGCACTCCAGCCT…[NEOcassette]*AAAAAAAAAAA*
AAAAAAAAAAAAAAAACATTACAAAAAAAAAAAAAAAAAAAAAAAGAAAAAAAAAAAAAAAAAAAAAAAA
AAAAAAAAAAAAAAAAAAACAGCAAAAGACTGGAAACCACTTAAGAAAGCCATCCATGGGGCCCGTAAATAA
TAAATTTAAAGAATTTATTTAGATAATATGCTTTTGAAACCATTCCAATATAACTT

LINE-1 flanking the insertion site.

CLONE 130

[BB4]

Driver: ORF2
Plasmid: AlurescueA70Du $A_{17}CATTACA_{18}GA_{17}CACACA_{18}$ (T)
Chromosome: 1
5' position: 212,590,765
Strand: plus
DR: AGAAAAGCCGCAAACTC
ENDOsite: TTCT/AA

Empty site:

TGAGCCAAGATCACACCATTGCACTCCAGCCTGGGCGACAATAGCAAAATTCTGTCTCAAAAAAAAAAAATTG
CTTAAAATGTTTTTCAGATCACAAATTCCAGTGAAACAGCTGACACCAACAAGTTTGAAGACACCAACAGAG
GAATGGAATCAGCATGAGAATACAGCTGCTTCTCTCTCTGTCCAATGATTTCACCCTGTTCCCTTTGGCCAA
TCAGCAGTCTCCACACTTCAGCCTACTCCTTAAAAACCCTT↑**AGAAAAGCCGCAAACTC**↓CTTGAGAGAGGG
TTTGAGGTTCCCCTCCATCTCCTCGATTGGAGGCCCTATTGGTAAGCCTCTTTTTCTACTGCAACTCCGTCT
TGGCATATTGACTTGC

Filled site:

TGAGCCAAGATCACACCATTGCACTCCAGCCTGGGCGACAATAGCAAAATTCTGTCTCAAAAAAAAAAAATTG
CTTAAAATGTTTTTCAGATCACAAATTCCAGTGAAACAGCTGACACCAACAAGTTTGAAGACACCAACAGAG
GAATGGAATCAGCATGAGAATACAGCTGCTTCTCTCTCTGTCCAATGATTTCACCCTGTTCCCTTTGGCCAA
TCAGCAGTCTCCACACTTCAGCCTACTCCTTAAAAACCCTTAGAAAAGCCGCAAACTC**GGCCGGGCGCGGTG
GCTCACGCCTGTAATCCCAGCACTTTGGGAGGCCGAGGCGGGCGGATCACGAGGTCAGGAGATCGAGACCAT
CCTGGCTAACACGGTGAAACCCCGTCTCTACTAAAAAAAAAATACAAAAAATTAGCCGGGCGTGGTAGCGGGC
GCCTGTAGTCCCAGCTACTCGGGAGGCTGAGGCAGGGGAATGGCGTGAACCCGGGAGGCGGAGCTTGCAGTG
AGCCGAGATCCCGCCACTGCACTCCA... [NEOcassette]** ***AAAAAAAAAAAAAAAAAACATTACAAAA
AAAAAAAAAAAAAAAAAAAAAGAAAAAAAAAAAAAAAAAAAAAAAAAAA***GAAAAGCCGCAAACTCCTTGAGA
GAGGGTTTGAGGTTCCCCTCCATCTCCTCGATTGGAGGCCCTATTGGTAAGCCTCTTTTTCTACTGCAACTC
CGTCTTGGCATATTGACTTGC

Alu at the 5'; LTR flanking the insertion site.

CLONE 131

[CC2]

Driver: L1
Plasmid: AlurescueA70Du $A_{17}CATTACA_{18}GA_{17}CACACA_{18}$ (T)
Chromosome: 1
5’ position: 156,439,894
Strand: plus
DR: undetermined
ENDOsite: undetermined

Empty site:

ATCATGTGTAGGTATCACTTTCATAACAATTTAAAAAAATAAAAAGATGGAAACAGATAAGTAGAGAAGGTC
TATGTCTTGGCCTCATGCCCAAGGGGCAGAGGGGCTGCAAGGGTACCATGCCGGGAGCACAGCTGGCCAAGG
TTCCAGCAGATCTCCCCATGAGGGGGTGTTCATTACGTCATAAAATGTCTACCTTAAAGAAGAAACTG↑↓AT
GGTTGCACTGTCACAGAATAAATAGAATCACTGAATGTTTCATGATTTACACAGCACTATTGTTTCATGCTG
TATCCTGTTTGGGACAGTTTAGAAAA

Filled site:

ATCATGTGTAGGTATCACTTTCATAACAATTTAAAAAAATAAAAAGATGGAAACAGATAAGTAGAGAAGGTC
TATGTCTTGGCCTCATGCCCAAGGGGCAGAGGGGCTGCAAGGGTACCATGCCGGGAGCACAGCTGGCCAAGG
TTCCAGCAGATCTCCCCATGAGGGGGTGTTCATTACGTCATAAAATGTCTACCTTAAAGAAGAAACT**GGGCC**
GGGCGCGGTGGCTCACGCCTGTAATCCCAGCACTTTGGGAGGCCGAGGCGGGCGGATCACGAGGTCAGGAGA
TCGAGACCATCCTGGCTAACACGGTGAAACCCCGTCTCTACTAAAAAAAAATACAAAAAATTAGCCGGGCGT
GGTAGCGGGCGCCTGTAGTCCCAGCTACTCGGGAGGCTGAGGCAGGGGAATGGCGTGAACCCGGGAGGCGGA
GCTTGCAGTGAGCCGAGATCCCGCCACTGCACTCCA…[NEOcassette]*AAAAAAAAAAAAAAAAAAAA*
AAAAACATTACAAAAAAAAAAAAAAAAAAAAAAAAAAAAAAAAAAAAAAAAAAAAAAAAAAAAAAAN
***AAAAAAA*[DR?][No3’SequenceReads]**

No repetitive sequences in the immediate proximity.

There are no sequence reads 3’ of the A-tail.

CLONE 132

[CC3]

Driver: L1
Plasmid: AlurescueA70Du $A_{17}CATTACA_{18}GA_{17}CACACA_{18}$ (T)
Chromosome: 4
5’ position: 173,162,876
Strand: minus
DR: AAGAAGGAGAAATCA
ENDOsite: TCTT/AA

Empty site:

TTTTCATTCAACTTTTATTAATAATCTGAATGAAGATTTTAAAAATGCTAATCTATTTTGTGATAATGTACA
GCTGAGAGGTGCAGCCAAACTGTTAAAAGATAAAATACAGTGTCAATAAATTCTCACAAAATATAAAACTAG
TTAAATGAACTATAATGGTATTACACATAGAAAATATAAGAAAAACAATAATGTTTAAATATGACTACGTTA
GTGGCTACTCAT↑**AAGAAGGAGAAATCA**↓GAGTTAATTGGATAAAAGCATAACTCTTAACTGATGTTAGCAT
ATGCCTTAATGCCTTAACATTTGGTTTACCTCTCTTTGGAAGCCTTCCTGGTCCTGC

Filled site:

TTTTCATTCAACTTTTATTAATAATCTGAATGAAGATTTTAAAAATGCTAATCTATTTTGTGATAATGTACA
GCTGAGAGGTGCAGCCAAACTGTTAAAAGATAAAATACAGTGTCAATAAATTCTCACAAAATATAAAACTAG
TTAAATGAACTATAATGGTATTACACATAGAAAATATAAGAAAAACAATAATGTTTAAATATGACTACGTTA
GTGGCTACTCATAAGAAGGAGAAATCA**GGCCGGGCGCGGTGGCTCACGCCTGTAATCCCAGCACTTTGGGAG
GCCGAGGCGGGCGGATCACGAGGTCAGGAGATCGAGACCATCCTGGCTAACACGGTGAAACCCCGTCTCTAC
TAAAAAAAAATACAAAAAATTAGCCGGGCGTGGTAGCGGGCGCCTGTAGTCCCAGCTACTCGGGAGGCTGAG
GCAGGGGAATGGCGTGAACCCGGGAGGCGGAGCTTGCAGTGAGCCGAGATCCCGCCACTGCACTCCA... [NEO
cassette]** ***AAAAAAAAAAAAAAAAAAAAAAAAAAAAAAAAACATTACAAAAAAAAAAAAAAAAAAAAAAA
AAAAAAAAAAAGAAAAAAAAAAAAAAAAAAAACACACAAAAAAAAAAAAAAAAAAAAAAAAAAAAAAAAAAA
AA***GAAGGAGAAATCAGAGTTAATTGGATAAAAGCATAACTCTTAACTGATGTTAGCATATGCCTTAATGCCT
TAACATTTGGTTTACCTCTCTTTGGAAGCCTTCCTGGTCCTGC

L3/CR1 at the 5’.

CLONE 133

[Alurescue2,6,7,10, Black-MP#1and#12, colored 33]

Driver: L1
Plasmid: AlurescueA70Du $A_{17}CATTACA_{18}GA_{17}CACACA_{18}$ (T)
Chromosome: 4
5' position: 145,551,973
Strand: plus
DR: AGAAATACTATAAAAAT
ENDOsite: TTCT/TT

Empty site:

TAGCTGTGTAGGCTTTCAATTCTGTGAATTTATAATGTCTTCCATATTTTATTGTTTACATAGTTAAGGTAT
TATACTTCCTTATAACATTTACATAGAGTTGAGATACAAATAAGTTAATTTAATTGATGTAATCATTAATTA
CTGAATCATCTCAGTTGTGATCGTTCTGTTTTATGAGGTAATTTCACGATGTAGAGACTGTTTTCCATTATT
TTATAATTAAGTACCATTTCTGCACACCCTCTCCTCCCATTAGCTCACCGTTGAAATTCCATGGAAGGAAAA
TGTATTAATAAATTACACCACTTGCAGCTGATTCAGGTATTACAGAAAATTGACGCTTACTAA↑**AGAAATAC
TATAAAAAT**↓AACAGTTAAGCCATTACCACTAAATAAAGCTATGCTTTAACTGAGTTTGGTTTGACAAGATG
CAATTCCAAGAGACATAACTTTCAGGCATCATAAGAAA

Filled site:

TAGCTGTGTAGGCTTTCAATTCTGTGAATTTATAATGTCTTCCATATTTTATTGTTTACATAGTTAAGGTAT
TATACTTCCTTATAACATTTACATAGAGTTGAGATACAAATAAGTTAATTTAATTGATGTAATCATTAATTA
CTGAATCATCTCAGTTGTGATCGTTCTGTTTTATGAGGTAATTTCACGATGTAGAGACTGTTTTCCATTATT
TTATAATTAAGTACCATTTCTGCACACCCTCTCCTCCCATTAGCTCACCGTTGAAATTCCATGGAAGGAAAA
TGTATTAATAAATTACACCACTTGCAGCTGATTCAGGTATTACAGAAAATTGACGCTTACTAAAGAAATACT
ATAAAAAT**GGCCGGGCGCGGTGGCTCACGCCTGTAATCCCAGCACTTTGGGAGGCCGAGGCGGGCGGATCAC
GAGGTCAGGAGATCGAGACCATCCTGGCTAACACGGTGAAACCCCGTCTCTACTAAAAAAAAAAATACAAAAA
ATTAGCCGGGCGTGGTAGCGGGCGCCTGTAGTCCCAGCTACTCGGGAGGCTGAGGCAGGGGAATGGCGTGAA
CCCGGGAGGCGGAGCTTGCAGTGAGCCGAGATCCCGCCACT…[NEOcassette]*AAAAAAAAAAAAAAA
AAAAAAAAAAAAAAAAAAAAAAAAAAAAAAAAAAAAAAAAAAAAAAAAAAAAAAAAAAAAAAAAAAAAAAAA
AAAAAAAAAAAACATTACAAAAAAAAAAAAAAAAAAAAAAAAAAAAAAAAAAAAAAAAAAAAAAAAAAAAAA
AAAAAAAAAAGAAAAAAAAAAGA*** GAAATACTATAAAAATAACAGTTAAGCCATTACCACTAAATAAAGCTAT
GCTTTAACTGAGTTTGGTTTGACAAGATGCAATTCCAAGAGACATAACTTTCAGGCATCATAAGAAA

No repetitive sequences in the immediate proximity.

CLONE 134

[Alurescue4,8,9, colored 35]

Driver: L1
Plasmid: AlurescueA70Du $A_{17}CATTACA_{18}GA_{17}CACACA_{18}$ (T)
Chromosome: 3
5’ position: 123,311,449
Strand: minus
DR: AAAAATTATAAATGCA
ENDOsite: TTTT/AA

Empty site:

ATGACATTCTGGAAAAAGCAAGACTGTAGAGACAGTAAAAAAATCAATGGTTACTATGGGTGTGGGAGGAGG
CTGGTTTGGGATGAATAGGTAAAGCACAGATTTTTAGGGCAGTGAAACTATTATGTACAATGTGCCATGACA
GATACATGGTATTATATATTCATAAAAATTCATATAGGCAAGGAACAAGGGATTATTAAAAATGACCAGACA
GATTTGAAAGGTTTATGTATTGTTTAGGAAGATGATAAAGTACTGATTAATATTAGACTTTAATGAGTTAAT
GATCCATGCTGTAATTTCTATGGTAACCAGTAAAAGAAGAGAAGCAAGATGTACAAATTCCAGTAAGTAGAG
GGGGAAGAATAAGTGATATTTT↑**AAAAATTATAAATGCA**↓AAAAAGGAGTGATACGGTTTAGCTGTGTGTCT
CCAACCAAATCTCATCTCAAATTGTAATCATCACGTGTTGAGGGAGGGACTGGTGGGAGGTGATTGGA

Filled site:

ATGACATTCTGGAAAAAGCAAGACTGTAGAGACAGTAAAAAAATCAATGGTTACTATGGGTGTGGGAGGAGG
CTGGTTTGGGATGAATAGGTAAAGCACAGATTTTTAGGGCAGTGAAACTATTATGTACAATGTGCCATGACA
GATACATGGTATTATATATTCATAAAAATTCATATAGGCAAGGAACAAGGGATTATTAAAAATGACCAGACA
GATTTGAAAGGTTTATGTATTGTTTAGGAAGATGATAAAGTACTGATTAATATTAGACTTTAATGAGTTAAT
GATCCATGCTGTAATTTCTATGGTAACCAGTAAAAGAAGAGAAGCAAGATGTACAAATTCCAGTAAGTAGAG
GGGGAAGAATAAGTGATATTTTAAAAATTATAAATGCA**GGCCGGGCGCGGTGGCTCACGCCTGTAATCCCAG**
CACTTTGGGAGGCCGAGGCGGGCGGATCACGAGGTCAGGAGATCGAGACCATCCTGGCTAACACGGTGAAAC
CCCGTCTCTACTAAAAAAAAAAATACAAAAAATTAGCCGGGCGTGGTAGCGGGCGCCTGTAGTCCCAGCTACT
CGGGAGGCTGAGGCAGGGGAATGGCGTGAACCCGGGAGGCGGAGCTTGCAGTGAGCCGAGATCCCGCCACTG
CACTCCA… [NEOcassette] ***AAAAAAAAAAAAAAAAAAAAAAAAAAAAAAAAAAAAAAAAAAAAAA***
AAAAAAAAAAATTATAAATGCAAAAAAGGAGTGATACGGTTTAGCTGTGTGTCTCCAACCAAATCTCATCTC
AAATTGTAATCATCACGTGTTGAGGGAGGGACTGGTGGGAGGTGATTGGA

LINE-1 flanking the insertion site; LTR at the 3’.

CLONE 135

[Black Alu rescue MP#1, purple MP#6 and purple MP#9 colored 36]

Driver: ORF2
Plasmid: AlurescueA70Du $A_{17}CATTACA_{18}GA_{17}CACACA_{18}$ (T)
Chromosome: 4
5' position: 151,058,540
Strand: plus
DR: undetermined
ENDOsite: undetermined

Empty site:

GTTTCACTCTGCCACCCAGGCTGTGCAGTGGTGCAGTCATAGCTCACTGCAGCCTTGAGCTCCTGGGCTCAA
GGGATCCTCCCACCTCAGCTTCCCAACTCATTGAGACTATGCGTGGGCCACCATGCCCAGTCTATTTATTAT
CCTTTAAATGGTGGCTGCAGCAGCTCTGCTGTTCTCAGCTGAGCTGCAAGATAGTAGGGAGTAGGGTGGAGT
TAGCTGGGACAGGTTGTCTGGGGCAGCTTCCCTCCACATGTCTCTCATCCTGCTTCCACCAGCAGGCTGGCT
CAGGCCTTCTCATGGTAAAGGCAGAAGCAGGAGGGAGCAAATGGAAACACATATGGCCTCTGGAAGCCCAGG
CTCAAAACTGGCACATTG↑↓TTATTTCTGCCTCATTCTGTTGGTTAAAATGAGACAAATGGTCAAACTCTTG
AGACAAGGGTTGATGAAATCCCTACAAAGGACATGTATACAGGGAGGG

Filled site: ...**[NEOcassette]**

GTTTCACTCTGCCACCCAGGCTGTGCAGTGGTGCAGTCATAGCTCACTGCAGCCTTGAGCTCCTGGGCTCAA
GGGATCCTCCCACCTCAGCTTCCCAACTCATTGAGACTATGCGTGGGCCACCATGCCCAGTCTATTTATTAT
CCTTTAAATGGTGGCTGCAGCAGCTCTGCTGTTCTCAGCTGAGCTGCAAGATAGTAGGGAGTAGGGTGGAGT
TAGCTGGGACAGGTTGTCTGGGGCAGCTTCCCTCCACATGTCTCTCATCCTGCTTCCACCAGCAGGCTGGCT
CAGGCCTTCTCATGGTAAAGGCAGAAGCAGGAGGGAGCAAATGGAAACACATATGGCCTCTGGAAGCCCAGG
CTCAAAACTGGCACATTG**GGCCGGGCGCGGTGGCTCACGCCTGTAATCCCAGCACTTTGGGAGGCCGAGGCG**
GGCGGATCACGAGGTCAGGAGATCGAGACCATCCTGGCTAACACGGTGAAACCCCGTCTCTACTAAAAAAAA
AATACAAAAAATTAGCCGGGCGTGGTAGCGGGCGCCTGTAGTCCCAGCTACTCGGGAGGCTGAGGCAGGGGA
ATGGCGTGAACCCGGGAGGCGGAGCTTGCAGTGA...[NEOcassette][No3'SequenceReads]

Alu at the 5'; LTR flanking the insertion site.

There are no 3' sequence reads to complete the A-tail and 3' flanking sequence.

CLONE 137

[Brown MP#26, (purple MP#3, 30, 31), Aluresuce15, Alurescue20, colored 34]

Driver: ORF2
Plasmid: AlurescueA70Du $A_{17}CATTACA_{18}GA_{17}CACACA_{18}$ (T)
Chromosome: 6
5' position: 106,930,681
Strand: plus
DR: AAAAAAGTAAAAATAA
ENDOsite: TTTT/AT

Empty site:

AGAATTCTTCTGGCCAGGCATGGTGGCTCACGCCTATAATCCCAGCACTTTGGGAGGCCGAGGGGGGTGAAT
CACAAGGTCAGGAGATCGAGACCATCCTGGCTAACACCGTGAAACCCCGTCTCTACTAAAAATACAAAAAAT
TAGCTGGGCGTGGTGGCGGGCACCTGTATTCCCAGCTACTTGGGAGGCTGAGGCAGTAGAATGGCATGAACC
GGGGAGACAGAGGTTGCAGTGAGCTGAAATCGTGCCACTGCACTGCAGCCTGGGCGACAGAGCGAGACTCCA
TCTCAAAAAATAATAATCAT↑**AAAAAAGTAAAAATAA**↓AAAAAGAATTCTTCCTACCACAAATATTTTTGGG
CCAACAGTCTTTAAATTTTTTTAATTGACATCTTGTAATTGTACATATTTATGGGGTATAATTTGA

Filled site:

AGAATTCTTCTGGCCAGGCATGGTGGCTCACGCCTATAATCCCAGCACTTTGGGAGGCCGAGGGGGGTGAAT
CACAAGGTCAGGAGATCGAGACCATCCTGGCTAACACCGTGAAACCCCGTCTCTACTAAAAATACAAAAAAT
TAGCTGGGCGTGGTGGCGGGCACCTGTATTCCCAGCTACTTGGGAGGCTGAGGCAGTAGAATGGCATGAACC
GGGGAGACAGAGGTTGCAGTGAGCTGAAATCGTGCCACTGCACTGCAGCCTGGGCGACAGAGCGAGACTCCA
TCTCAAAAAATAATAATCATAAAAAAGTAAAAATAA**GGCCGGGCGCGGTGGCTCACGCCTGTAATCCCAGCA**
CTTTGGGAGGCCGAGGCGGGCGGATCACGAGGTCAGGAGATCGAGACCATCCTGGCTAACACGGTGAAACCC
CGTCTCTACTAAAAAAAATACAAAAAATTAGCCGGGCGTGGTAGCGGGCGCCTGTAGTCCCAGCTACTCGGG
AGGCTGAGGCAGGGGAATGGCGTGAACCCGGGAGGCGGAGCTTGCAGTGAGCCGAGATCCCGCCACTGCACT
CCAGCC... [NEOcassette] ***AAAAAAAAAAAAAAAAAAAAAAAAAAAACATTACAAAAAAAAAAAAAAAA***
AAAAAAAAAAAAAAAAAAAAAAAAAAAAAGAAAAAAAAAAAAAAAAAAAAAAGTAAAAATAAAAAAAGAATT
CTTCCTACCACAAATATTTTTGGGCCAACAGTCTTTAAATTTTTTTAATTGACATCTTGTAATTGTACATAT
TTATGGGGTATAATTTGA

Alu at the 5' of the insertion site; LINE-1 at the 3'.

CLONE 138

[Black Alu rescue MP#3 colored 38]

Driver: ORF2
Plasmid: AlurescueA70Du $A_{17}CATTACA_{18}GA_{17}CACACA_{18}$ (T)
Chromosome: 14
5' position: 50329568
Strand: minus
DR: undetermined
ENDOsite: undetermined

Empty site:

CTGTAAGGCAGAACGGCTGCCCAGCTCGGATGACGCCACACTAACGTAGCCTCCAGACCGCCCAGTGTGGGT
GTGTCCAAGCTCACGTCGCCCGGCGTGGCCCCCCCGCTCCCCAATGACGTAACTGCCCTGCAGCTTCTAGTA
GCTTTTCGCAGCGTCTCCGACC↑↓GCCGGGCGCGGTGGCGCGTGCCTGTAGTCCCAGCTACTCGGGAGGCTG
AGGTGGGAGGATCGCTTGAGCCCAGGAGTTCTGGGCTGTAGTGCGCTATGCC

Filled site:

CTGTAAGGCAGAACGGCTGCCCAGCTCGGATGACGCCACACTAACGTAGCCTCCAGACCGCCCAGTGTGGGT
GTGTCCAAGCTCACGTCGCCCGGCGTGGCCCCCCCGCTCCCCAATGACGTAACTGCCCTGCAGCTTCTAGTA
GCTTTTCGCAGCGTCTCCGACC**GGCCGGGCGCGGTGGCTCACGCCTGTAATCCCAGCACTTTGGGAGGCCGA**
GGCGGGCGGATCACGAGGTCAGGAGATCGAGACCATCCTGGCTAACACGGTGAAACCCCGTCTCTACTAAAA
AAAAAATACAAAAAATTAGCCGGGCGTGGTAGCGGGCGCCTGTAGTCCCAGCTACTCGGGAGGCTGAGGCAG
GGGAATGGCGTGAACCCGGGAGGCGGAGCTTGCAGTGAGCCGAGATCCCGCCACTGCACTCCAGCCTG... [NEOcassette][No3' SequenceReads]

No repetitive sequences in the immediate proximity.

There are no 3' sequence reads to complete the A-tail and 3' flanking sequence.

CLONE 139

[D13, D39]

Driver: ORF2
Plasmid: AlurescueA70Du $A_{17}CATTACA_{18}GA_{17}CACACA_{18}$ (T)
Chromosome: 1
5’ position: 16,216,890
Strand: minus
DR: AAGAATACAAAAATTAG
ENDOsite: TCTT/AG

Empty site:

ATGCTGTAAATGTATTAAAAAGCATTAACTGTCCACTTAAATGGGTGAATTTTACAGTGGAAGTTGCAGTGA
GCAGAGATTGCACTCCAGCCTGGGAAGCAGAGCAAGACCCAGTCTTAAAAAAAAAAAAAAAAAAAAAACCTG
AGTCAAAATCTAAAAATATCAACCAGGCCGGGCACGGTGGCTCATGCCTATAATCCCAGCACTTTGGGAGGT
CGGGGTGGGCGGATCATAAGGTCAAGAGTTCGAGACCAGCCTGGCCAACATGGTGAAACTCAGTCTCTACT↑
AAGAATACAAAAATTAG↓CTGGGTGTGGTGGTGCGTGCCTGTAATCCCAGCTACTCGGGAGGCTGAGGCAGG
AAAATCGCTTGAACCCGGGAGGCAGAGGTTGCAGTGAGCCTAGATCACATGAAAAATAAAAATAAAAATGTC
AACCTGATGTTATTGAACTGTATATAATGTGTTCCCACCTATTTGTAAGAAAATGTTTTACTGCCCTCATCT
TA

Filled site:

ATGCTGTAAATGTATTAAAAAGCATTAACTGTCCACTTAAATGGGTGAATTTTACAGTGGAAGTTGCAGTGA
GCAGAGATTGCACTCCAGCCTGGGAAGCAGAGCAAGACCCAGTCTTAAAAAAAAAAAAAAAAAAAAAACCTG
AGTCAAAATCTAAAAATATCAACCAGGCCGGGCACGGTGGCTCATGCCTATAATCCCAGCACTTTGGGAGGT
CGGGGTGGGCGGATCATAAGGTCAAGAGTTCGAGACCAGCCTGGCCAACATGGTGAAACTCAGTCTCTACTA
AGAATACAAAAATTAG**GGCCGGGCGCGGTGGCTCACGCCTGTAATCCCAGCACTTTGGGAGGCCGAGGCGGG
CGGATCACGAGGTCAGGAGATCGAGACCATCCTGGCTAACACGGTGAAACCCCGTCTCTACTAAAAAAAAAA
ATACAAAAAATTAGCCGGGCGTGGTAGCGGGCGCCTGTAGTCCCAGCTACTCGGGAGGCTGAGGCAGGGGAA
TGGCGTGAACCCGGGAGGCGGAGCTTGCAGTGAGCCGAGATCCCGCCACTGCACTCCAGCCT…[NEOcass
ette]**_**AAAAAAAAAAAAAAAAAAAAAACATTACAAAAAAAAAAAAAAAAAAAAAAAAAAGAAAAAAAAAAAA
AAAAAAAAAAAAAAAAAAAAAAAAAAAAAAAAAAAAAAAAAAAAAAAAAAAAAAAAAAAAAA**_GAATACAAAA
ATTAGCTGGGTGTGGTGGTGCGTGCCTGTAATCCCAGCTACTCGGGAGGCTGAGGCAGGAAAATCGCTTGAA
CCCGGGAGGCAGAGGTTGCAGTGAGCCTAGATC

LINE-1 at the 5’; Alu flanking the insertion site.

CLONE 140

[D127]

Driver: ORF2
Plasmid: AlurescueA70Du $A_{17}CATTACA_{18}GA_{17}CACACA_{18}$ (T)
Chromosome: 17
5' position: 37,132,159
Strand: minus
DR: AAAATTGA
ENDOsite: TTTT/TA

Empty site:

AATTCCAGCTACTCAGGAGGCTGAAGCAGGAGAATCACTTGAACCCAGGAGGCGGAGGTTGCAGTGTGCTTG
AGATTGCACCACTGCACTCCAGCCTGGGCAATAGAGTGAGACTTCATCTCAAAAAAAAAAAAAAAAAGTAAC
TCTCCACTGCCCTCTCCCTCCAGGCCGAGGCAATCACCATTCTACTTTCTGTGTCTATGATTTTGACTACTC
TAACTTCCTCATA↑**AAAATTGA**↓ATCCTGGGCCGGGCGCAGTGGCTCACGCCTGTAATCCCAGCACTTTGGG
AGGCCGAGGCAGGCGGATCACGAGGTCAGGAGATCGAGACCATCCTGGCTA

Filled site:

AATTCCAGCTACTCAGGAGGCTGAAGCAGGAGAATCACTTGAACCCAGGAGGCGGAGGTTGCAGTGTGCTTG
AGATTGCACCACTGCACTCCAGCCTGGGCAATAGAGTGAGACTTCATCTCAAAAAAAAAAAAAAAAAGTAAC
TCTCCACTGCCCTCTCCCTCCAGGCCGAGGCAATCACCATTCTACTTTCTGTGTCTATGATTTTGACTACTC
TAACTTCCTCATAAAAATTGA**GGCCGGGCGCGGTGGCTCACGCCTGTAATCCCAGCACTTTGGGAGGCCGAG**
GCGGGCGGATCACGAGGTCAGGAGATCGAGACCATCCTGGCTAACACGGTGAAACCCCGTCTCTACTAAAAA
AAAATACAAAAAATTAGCCGGGCGTGGTAGCGGGCGCCTGTAGTCCCAGCTACTCGGGAGGCTGAGGCAGGG
GAATGGCGTGAACCCGGGAGGCGGAGCTTGCAGTGAGCCGAGATCCCGCCACTGCACTCCAGCCT... [NEOca
ssette]*AAAAAAAAAAAAAAAAAAAAAAAACATTACAAAAAAAAAAAAAAAAAAAAAAAAAAAAGAAAAA*
AAAAAAAAAAAAAAAAAAAAAAAAAAAAAAAAAAAAAAAAAAAAAAAAAAAAAAAAAAAAAAAAAAAAAAAA
AAACACACAAAATTGAATCCTGGGCCGGGCGCAGTGGCTCACGCCTGTAATCCCAGCACTTTGGGAGGCCG
AGGCAGGCGGATCACGAGGTCAGGAGATCGAGACCATCCTGGCTA

Alu 5' and 3'; LINE-1 flanking the insertion site.

CLONE 141

[S1, S2]

Driver: L1
Plasmid: AlurescueA70Du $A_{17}CATTACA_{18}GA_{17}CACACA_{18}$ (T)
Chromosome: 16
5' position: 57,139,354
Strand: minus
DR: AAAAA
ENDOsite: TTTT/AA

Empty site:

GGCACTGATTCATTGGTTCATTCAACAAATACTCCCTGAGCACCCCGGGGCTATGCAGTTAATAAAACACAC
AGGAGCCAGTGGTGGCTCACACCTGTAATCTCAGCACTTTGGGAGGCTGAGGTGGAAGAATGGCTTGGGCCC
AGGAGTTTGGGGCTGCGGTGAGCTGTGATTGTGCCACTGCACTCCAACCTGGGCGATAGAGCAAAACCCTGT
CTTAAAAGTT↑**AAAAA**↓AAAAAAAAAAACCGGACACATAGGATTCAGCCCTCACAGGGTCCACTGACCCCAG
CGAGGAGGCATCTTGCCCTTAGGCAGGACAGGCTTGGACATTGGT

Filled site:

GGCACTGATTCATTGGTTCATTCAACAAATACTCCCTGAGCACCCCGGGGCTATGCAGTTAATAAAACACAC
AGGAGCCAGTGGTGGCTCACACCTGTAATCTCAGCACTTTGGGAGGCTGAGGTGGAAGAATGGCTTGGGCCC
AGGAGTTTGGGGCTGCGGTGAGCTGTGATTGTGCCACTGCACTCCAACCTGGGCGATAGAGCAAAACCCTGT
CTTAAAAGTTAAAAA**GGCCGGGCGCGGTGGCTCACGCCTGTAATCCCAGCACTTTGGGAGGCCGAGGCGGGC
GGATCACGAGGTCAGGAGATCGAGACCATCCTGGCTAACACGGTGAAACCCCGTCTCTACTAAAAAAAAATA
CAAAAAATTAGCCGGGCGTGGTAGCGGGCGCCTGTAGTCCCAGCTACTCGGGAGGCTGAGGCAGGGGAATGG
CGTGAACCCGGGAGGCGGAGCTTGCAGTGAGCCGAGATCCCGCCACTGCACTCCAGCCT…[NEOcassett
e]*AAAAAAAAAAAAAAAAAAAAAAAAAAAAAAAAAAAAAAAAAAAAAA***AAAAAAAAAAAACCGGACACATA
GGATTCAGCCCTCACAGGGTCCACTGACCCCAGCGAGGAGGCATCTTGCCCTTAGGCAGGACAGGCTTGGAC
ATTGGT

Alu 5' of the insertion site.

CLONE 142

[S31]

Driver: L1
Plasmid: AlurescueA70D $A_{17}CATTACA_{18}GA_{17}CACACA_{18}$ (T)
Chromosome: X
5’ position: 13,030,507
Strand: minus
DR: AAGGACTCTTGTG
ENDOsite: CCTT/AA

Empty site:

GAGCAAGGTGTCGGCGGGACTGCATTCCTGTCTGTAGGCTCTTGGTGAGAATCCATTTCCTCACCTTCTCCA
GCTTCTAGAGGTTTCTACATTCCTTGGCTTAGGACTCCCTCCCTCTATCTTCAAAGCCAGCAGTGCCCGGCC
AAGTCCTTCTCAGGCTGCCATCCCGCTGGTCCTCCCTTGCATAGTCCCATCTTCCTTTTTTGCTTTT↑**AAGG**
ACTCTTGTG↓ATTACATTAAGTCCATCCCAAAACTTCAGGATAGTCACCCTATTTTAAAGTCATCTGCTTAG
CAACCTTAATTCCATCTGCCACCTAAATTCCCCTTTCC

Filled site:

GAGCAAGGTGTCGGCGGGACTGCATTCCTGTCTGTAGGCTCTTGGTGAGAATCCATTTCCTCACCTTCTCCA
GCTTCTAGAGGTTTCTACATTCCTTGGCTTAGGACTCCCTCCCTCTATCTTCAAAGCCAGCAGTGCCCGGCC
AAGTCCTTCTCAGGCTGCCATCCCGCTGGTCCTCCCTTGCATAGTCCCATCTTCCTTTTTTGCTTTTAAGGA
CTCTTGTG**GGCCGGGCGCGGTGGCTCACGCCTGTAATCCCAGCACTTTGGGAGGCCGAGGCGGGCGGATCAC**
GAGGTCAGGAGATCGAGACCATCCTGGCTAACACGGTGAAACCCCGTCTCTACTAAAAAAAAATACAAAAAA
TTAGCCGGGCGTGGTAGCGGGCGCCTGTAGTCCCAGCTACTCGGGAGGCTGAGGCAGGGGAATGGCGTGAAC
CCGGGAGGCGGAGCTTGCAGTGAGCCGAGAT…[NEOcassette]*AAAAAAAAAAAAAAAAAAAAAAAAA*
AAAAAAAAAAAAAAAAAAAAAAAAAAAAAAAAAAAAAAAAAAAAAAAAAAAAAAAAAAAAAAAAAAAAAAAA
AAAAAAAAAAAAAAAAAAAAAAAAAAAAAAAAAAAAAAAAAAAAAAAAAAAAAAAAAAGGACTCTTGTGATT
ACATTAAGTCCATCCCAAAACTTCAGGATAGTCACCCTATTTTAAAGTCATCTGCTTAGCAACCTTAATTCC
ATCTGCCACCTAAATTCCCCTTTCC

LTR flanking the insertion site.

CLONE 143

[Green Alu rescue MP#82, colored 41]

Driver: ORF2
Plasmid: AlurescueA70Du $A_{17}CATTACA_{18}GA_{17}CACACA_{18}$ (T)
Chromosome: 18
5' position: 71,783,287
Strand: plus
DR: ATTTCT
ENDOsite: AAAT/GC

Empty site:

TGTACACAAAAATAATTTGACAGAAAAATCTGGAACTGCAAAAGATGTGAAAGATGTTTTGCTCCACTCTCC
TGGGTATCTATTTCTTTAGTGATACTGTACTTTCTTGGTCACTGTAGTTTCAGAGTGGTTAACATCTGCTAA
GCCAGCCCCCTCTCAATTCATTTTCAAAACTTAAGACCTGGATGGTGATCACACAGGTGTTTTCTTTACAAT
TATCTGTCTTATGC↑**ATTTCT**↓CTGTGTGTCATTTCACAATACAAAGATGTTTAATTACCAAAAAAAAGGAA
AAGCAAGGGAACAATTAACACAACAGACAGAATGGTGGTTCCCTCTGGCA

Filled site:

TGTACACAAAAATAATTTGACAGAAAAATCTGGAACTGCAAAAGATGTGAAAGATGTTTTGCTCCACTCTCC
TGGGTATCTATTTCTTTAGTGATACTGTACTTTCTTGGTCACTGTAGTTTCAGAGTGGTTAACATCTGCTAA
GCCAGCCCCCTCTCAATTCATTTTCAAAACTTAAGACCTGGATGGTGATCACACAGGTGTTTTCTTTACAAT
TATCTGTCTTATGCATTTCT**CAGTGAGCCGAGATCCCGCCACTGCACTCCAG**…**[NEOcassette]*AAAA***
AAAAAAAAAAAAAAAAAAAACATTACAAAAAAAAAAAAAAAAAAAAAAAAAAAAAAAAAAAAAAAAAA
AAAAAGAAAAAAAAATTTCT**[No3'SequenceReads]**

LINE-1 at the 3'.

Alu truncation missing 226 5' bp.

CLONE 144

[T13]

Driver: ORF2
Plasmid: Alurescue A30D $A_{10}CTA_{10}TACA_{10}$
Chromosome: 8
5' position: 119,981,513
Strand: plus
DR: AAGACTTATTTTG
ENDOsite: TCTT/AT

Empty site:

TACAAGGATCTCTATAAGAGGAAGGCAAGAGGATCAGTCACTAGAAGGAATTGTGACAATGGAAGTAGAGTT
TGGAGATATGGGGTCAAGAGCCAAGAAACGCCAACAACCTCTGGAAGCGAATAAAGGAGAGAACAGATTTTC
CCCTGAAGTCTCCAGAAGGAACCAGCACTGACAGTACTTTGTAGCCCCAT↑**AAGACTTATTTTG**↓AACTTGT
GGCTTCCAGAATGATAGTAAATTCATGTTTCTTTAAGCCACTAGATGTGTGATACTATTGCAGCAAACATAG
GCAACTAATGGAGACTCATGG

Filled site:

TACAAGGATCTCTATAAGAGGAAGGCAAGAGGATCAGTCACTAGAAGGAATTGTGACAATGGAAGTAGAGTT
TGGAGATATGGGGTCAAGAGCCAAGAAACGCCAACAACCTCTGGAAGCGAATAAAGGAGAGAACAGATTTTC
CCCTGAAGTCTCCAGAAGGAACCAGCACTGACAGTACTTTGTAGCCCCATAAGACTTATTTTG**GGCCGGGCG**
CGGTGGCTCACGCCTGTAATCCCAGCACTTTGGGAGGCCGAGGCGGGCGGATCACGAGGTCAGGAGATCGAG
ACCATCCTGGCTAACACGGTGAAACCCCGTCTCTACTAAAAAAAAAAATACAAAAAATTAGCCGGGCGTGGTA
GCGGGCGCCTGTAGTCCCAGCTACTCGGGAGGCTGAGGCAGGGGAATGGCGTGAACCCGGGAGGCGGAGCTT
GCAGTGAGCCGACATCCCGCCACTGCACTCCAGCCTG… [NEOcassette] ***AAAAAAAAAACTAAAAAA***
AAAAATACAAAAAAAAAAAAAAAAAAAAAAAAAAAAAAAAAAAAAAAAAAAAAAAAAAAAAAAAAAAAAG
ACTTATTTTGAACTTGTGGCTTCCAGAATGATAGTAAATTCATGTTTCTTTAAGCCACTAGATGTGTGATAC
TATTGCAGCAAACATAGGCAACTAATGGAGACTCATGG

LTR flanking the insertion site.

CLONE 150

[LZ71]

Driver: ORF2
Plasmid: AlurescueA70Du $A_{17}CATTACA_{18}GA_{17}CACACA_{18}$ (T)
Chromosome: 1
5’ position: 1,329,515
Strand: plus
DR: AAAAAAAAAA
ENDOsite: TTTT/GG

Empty site:

CTATAATCCCAGCACTTTGGGAGGCTGAGGCGGGCAGATCACCTGAGGTCAGGAGTTCGAGAACAGCCTGAC
CAATATGGAGAAACCCAGTCTCTACTAAAAATACAAAATTAGTCAGGCATGGTGGTGCACGCCTGTAATCCC
AGGTACTCAGGAGGCTGAGGCAGGAGAATCGCTTGAAACCAGGAGGCGGAGGCTGCATGAGCTGAGGTTGCG
CCATTGCACTCCAGCCTGGACAACAGAGCGAGATTCCGTCTCC↑**AAAAAAAAAA**↓AAAAAGCACTTCAATCT
GTGGTGGGCAATTTGTGGATAATTTGAACAGATGGCAGAAAGCCGAACAAGCAGATGAGGAAACCCACCAAT
AAAAAGGAGAT

Filled site:

CTATAATCCCAGCACTTTGGGAGGCTGAGGCGGGCAGATCACCTGAGGTCAGGAGTTCGAGAACAGCCTGAC
CAATATGGAGAAACCCAGTCTCTACTAAAAATACAAAATTAGTCAGGCATGGTGGTGCACGCCTGTAATCCC
AGGTACTCAGGAGGCTGAGGCAGGAGAATCGCTTGAAACCAGGAGGCGGAGGCTGCATGAGCTGAGGTTGCG
CCATTGCACTCCAGCCTGGACAACAGAGCGAGATTCCGTCTCCAAAAAAAAAA**GGCCGGGCGCGGTGGCTCA
CGCCTGTAATCCCAGCACTTTGGGAGGCCGAGGCGGGCGGATCACGAGGTCAGGAGATCGAGACCATCCTGG
CTAACACGGTGAAACCCCGTCTCTACTAAAAAAAAATACAAAAAATTAGCCGGGCGTGGTAGCGGGCGCCTG
TAGTCCCAGCTACTCGGGAGGCTGAGGCAGGGGAATGGCGTGAACCCGGGAGGCGGAGCTTGCAGTGAGCCG
AGATCCCGCCACTGCA... [NEOcassette] [Atail]**AAAAAAAAAAAAAAAGCACTTCAATCTGTGG
TGGGCAATTTGTGGATAATTTGAACAGATGGCAGAAAGCCGAACAAGCAGATGAGGAAACCCACCA
ATAAAAAGGAGATGGAACCAAAGATGGAAACAATTTTTTGGGAAAACAAGTGCTTTGTTTCCATGT
GACACAGAAGGCACCCACAACGCTGGGAGTGAGGGACCGTGTCACGG

Alu flanking the insertion site.

CLONE 151

[GG29]

Driver: ORF2
Plasmid: AlurescueA70Du $A_{17}CATTACA_{18}GA_{17}CACACA_{18}$ (T)
Chromosome: 1
5’ position: 8,215,946
Strand: minus
DR: AAAAACAAAAAAGGA
ENDOsite: TTTT/GT

Empty site:

GTAAGGCGAACCAAGAGAAAGGCAAGGAGAAGCGGCCACTCCGAAGGCAGCAGGGACGCAAGTAGGAGTGAC
CAAGAAAATTAGCCAGGTGCTGTGGCGCACACCTGTGGTCCCAGCTACTGGGGAGGCTAAGGTGAGAGAATG
GCCTGCGCCCAGGAGGTTGAGGCTGCAGTGTGCTGTGATTGTACCACAGCACTCCAGCCTGGGCAACAGGGT
GAGACCCTGTCTCAAAAAC↑**AAAAACAAAAAAGGA**↓GATGACCTAAAAGAGGAGCACTCCGTGGCTCCTCAG
CCCTTGGAGAAGGAAGCTGCTTTGCTCTGCCGTGCTCCACCGGCCTCCTGCGTCCCCCTCCTCCCCTTCCTC
CACCTCCAAACCACATTCCAAGAGAGGCGGGGACTGAAACCAATTGTTCCAGCCTGGGATTAGGCAAACGTG
GGCCCTGTACCTTCAGCTCC

Filled site:

GTAAGGCGAACCAAGAGAAAGGCAAGGAGAAGCGGCCACTCCGAAGGCAGCAGGGACGCAAGTAGGAGTGAC
CAAGAAAATTAGCCAGGTGCTGTGGCGCACACCTGTGGTCCCAGCTACTGGGGAGGCTAAGGTGAGAGAATG
GCCTGCGCCCAGGAGGTTGAGGCTGCAGTGTGCTGTGATTGTACCACAGCACTCCAGCCTGGGCAACAGGGT
GAGACCCTGTCTCAAAAACAAAAACAAAAAAGGA**GGCCGGGCGCGGTGGCTCACGCCTGTAATCCCAGCACT
TTGGGAGGCCGAGGCGGGCGGATCACGAGGTCAGGAGATCGAGACCATCCTGGCTAACACGGTGAAACCCCG
TCTCTACTAAAAAAAAATACAAAAAATTAGCCGGGCGTGGTAGCGGGCGCCTGTAGTCCCAGCTACTCGGGA
GGCTGAGGCAGGGGAATGGCGTGAACCCGGGAGGCGGAGCTTGCAGTGAGCCGAGATCCCGCCACTGCACTC
C…[NEOcassette][Atail]**AAAAACAAAAAAGGAGATGACCTAAAAGAGGAGCACTCCGTGGCTCCT
CAGCCCTTGGAGAAGGAAGCTGCTTTGCTCTGCCGTGCTCCACCGGCCTCCTGCGTCCCCCTCCTCCCCTTC
CTCCACCTCCAAACCACATTCCAAGAGAGGCGGGGACTGAAACCAATTGTTCCAGCCTGGGATTAGGCAAAC
GTGGGCCCTGTACCTTCAGCTCC

Alu at the 5'.

CLONE 152

[LZ62]

Driver: ORF2
Plasmid: AlurescueA70Du $A_{17}CATTACA_{18}GA_{17}CACACA_{18}$ (T)
Chromosome: 1
5' position: 8,937,306
Strand: minus
DR: N/A
ENDOsite: N/A

Empty site:

AAAGCTTCCTGTTCAATGTTCCCCTGCTCTGTGCGCTTCTCTGGGCGGTAGGGTCCATTGCTTCACTTGCCT
TTCAGGCCCACCCGGGCCCTGACTGCTCCATTCATTTACCGTGTCCCTAATTCGGGGTTCCATCCAGGCAGC
TAAAAAAGCTGTGAATGGTCCGGGCGCGGTGGCTCACGCCTGTAATCCGAGCACTTTGGGATGCCGAGGTGG
GCCTCCTCAGGATCACCTGAGGTCGGGAGTTAGAGACCAGCCTGACCAACGTGGTGAAACCCCGTCTCTACT
GAAAATACAAAAATCGACCA↑GGCGCGGTGGCTCACGCCTGTAATCC↓TAGCACTTTGGGAGGCCGAGGTGG
GTGGCTCACGAGGTCAGGAGATCGAGACCATCCTACTCTACTAAACCCCATCTCTACTAAAAATACAAAAAA
TTAGCCGGGTGTGGTGGTGGGCGCCTGTAGTCCCAGCTACTCTGGAGGCGGAGCCTGCAGTGAGTCGAGATC
GTGTCA

Filled site:

AAAGCTTCCTGTTCAATGTTCCCCTGCTCTGTGCGCTTCTCTGGGCGGTAGGGTCCATTGCTTCACTTGCCT
TTCAGGCCCACCCGGGCCCTGACTGCTCCATTCATTTACCGTGTCCCTAATTCGGGGTTCCATCCAGGCAGC
TAAAAAAGCTGTGAATGGTCCGGGCGCGGTGGCTCACGCCTGTAATCCGAGCACTTTGGGATGCCGAGGTGG
GCCTCCTCAGGATCACCTGAGGTCGGGAGTTAGAGACCAGCCTGACCAACGTGGTGAAACCCCGTCTCTACT
GAAAATACAAAAATCGACCA**GGCGCGGTGGCTCACGCCTGTAATCCCAGCACTTTGGGAGGCCGAGGCGGGC**
GGATCACGAGGTCAGGAGATCGAGACCATCCTGGCTAACACGGTGAAACCCCGTCTCTACTAAAAAAAAATA
CAAAAAATTAGCCGGGCGTGGTAGCGGGCGCCTGTAGTCCCAGCTACTCGGGAGGCTGAGGCAGGGGAATGG
CGTGAACCCGGGAGGCGGAGCTTGCAGTGAGCCGAGATCCCGCC… [NEOcassette][Atail and 3' unsequenced]

Alu flanking the insertion site.

Inserted by recombination with a pre-existing truncated Alu: missing between 5-31 bp of 5' Alu sequence.

Sequence is shared between the Alu rescue vector and the genomic pre-insertion site.

CLONE 153

[LZ12]

Driver: ORF2
Plasmid: AlurescueA70Du $A_{17}CATTACA_{18}GA_{17}CACACA_{18}$ (T)
Chromosome: 1
5' position: 21,737,472
Strand: minus
DR: AAAAATCAGAAATG
ENDOsite: TTTT/AT

Empty site:

GAATTCCTCTTAGGGATATATTAGTAAAGTAACAGTATATATGGTATAGTATAGTATAGTAGTAATACTGGA
ATCAGACTGCCTAGCTCTGATGCTTATACCCATTCAACCTTCTTAACCTTTCTGAGCCTTTGTCCTTACCTT
AACAATAGATAAAATCAACGAAACCAAAGTTGTTTCTTTGAAAAGATTGACAGAATTGACAAACTTTTAGCT
AGACTGGCCAGTGAAAAAAGGGAGAAGACTCAAATT↑**AAAAATCAGAAATG**↓AAAACGGGAACATTACCACC
AACCTTACAGAAATAAAAAGGATTATAGGAGAATACTATGAACAATTGTATACTAACAAATTCGATAACATA
GATGAAATGGATAAATTTCTCAAAAGACAAACTGCCAAAACTGAATCAAAAAGAGAAAAAAAAAAATCTAAAT
AGACCTATGACAAGTAAAGAGATTCAATCAGTAGTC

Filled site:

GAATTCCTCTTAGGGATATATTAGTAAAGTAACAGTATATATGGTATAGTATAGTATAGTAGTAATACTGGA
ATCAGACTGCCTAGCTCTGATGCTTATACCCATTCAACCTTCTTAACCTTTCTGAGCCTTTGTCCTTACCTT
AACAATAGATAAAATCAACGAAACCAAAGTTGTTTCTTTGAAAAGATTGACAGAATTGACAAACTTTTAGCT
AGACTGGCCAGTGAAAAAAGGGAGAAGACTCAAATTAAAAATCAGAAATG**GGCCGGGCGCGGTGGCTCACGC
CTGTAATCCCAGCACTTTGGGAGGCCGAGGCGGGCGGATCACGAGGTCAGGAGATCGAGACCATCCTGGCTA
ACACGGTGAAACCCCGTCTCTACTAAAAAAAAAAAATACAAAAAATTAGCCGGGCGTGGTAGCGGGCGCCTGT
AGTCCCAGCTACTCGGGAGGCTGAGGCAGGGGAATGGCGTGAACCCGGGAGGCGGAGCTTGCAGTGAGCCGA
GATCCCGCC**…**[NEOcassette][Atail]**AAAAATCAGAAATGAAAACGGGAACATTACCACCAACCTT
ACAGAAATAAAAAGGATTATAGGAGAATACTATGAACAATTGTATACTAACAAATTCGATAACATAGATGAA
ATGGATAAATTTCTCAAAAGACAAACTGCCAAAACTGAATCAAAAAGAGAAAAAAAAAAATCTAAATAGACCT
ATGACAAGTAAAGAGATTCAATCAGTAGTC

LINE-1 flanking the insertion site.

CLONE 154

[LZ60]

Driver: ORF2
Plasmid: AlurescueA70Du $A_{17}CATTACA_{18}GA_{17}CACACA_{18}$ (T)
Chromosome: 1
5’ position: 54,191,570
Strand: plus
DR: AAGAACTTCCC
ENDOsite: TCTT/GA

Empty site:

CCGACCCTCCAGGCTGCGAGCTCCGAGGAGGCAAGGGCTCCATCTGTTGGTTCTCCATGGTGTCCCAGGGCA
GAACCCAACACTGGGAGCACAGCGGGCATCCGATAAATATGGATAAACCAACCAACCACCAGCCAAACACAG
GTACAGCCATTCCAGTTTTCAGGCTAATTCTACATACTTCATCCCATCTGCCCCTAACAATTGGCCAGACAG
GGGTCATTCCCCCATTTTAAAAGGGGGAACTGAGTCTCAAAAATTGAGAGCCACTCTGAGTTGCTGTACTGA
AAATCTGCTTC↑**AAGAACTTCCC**↓AAGAAGCCCCACCTTGACTGGAGAGGGGCAGATTCATCCTGCCAGGGT
GCTGTGTTCTTTCTATCCTTTGCTCTATCCTCTATCAGCAAGTCAGAGATGCGTTCATGACATATCCTGCTT
CCAAGAAGCCCACAGTTTCATGGGGAAGGGAAAAATGAACGCAAGCATCTGGAACACCTGACTCTTTCATGA
GAGATAAA

Filled site:

CCGACCCTCCAGGCTGCGAGCTCCGAGGAGGCAAGGGCTCCATCTGTTGGTTCTCCATGGTGTCCCAGGGCA
GAACCCAACACTGGGAGCACAGCGGGCATCCGATAAATATGGATAAACCAACCAACCACCAGCCAAACACAG
GTACAGCCATTCCAGTTTTCAGGCTAATTCTACATACTTCATCCCATCTGCCCCTAACAATTGGCCAGACAG
GGGTCATTCCCCCATTTTAAAAGGGGGAACTGAGTCTCAAAAATTGAGAGCCACTCTGAGTTGCTGTACTGA
AAATCTGCTTCAAGAACTTCCC**GGCCGGGCGCGGTGGCTCACGCCTGTAATCCCAGCACTTTGGGAGGCCGA
GGCGGGCGGATCACGAGGTCAGGAGATCGAGACCATCCTGGCTAACACGGTGAAACCCCGTCTCTACTAAAA
AAAAAATACAAAAAAAAATTAGCCGGGCGTGGTAGCGGGCGCCTGTAGTCCCAGCTACTCGGGAGGCTGAGG
CAGGGGAATGGCGTGAACCCGGGAGGCGGAGCTTGCAGTGAGCCGAGATCCCGCCACTGCACTC… [NEOca
ssette] [Atail]**AAGAACTTCCCAAGAAGCCCCACCTTGACTGGAGAGGGGCAGATTCATCCTGCCAGG
GTGCTGTGTTCTTTCTATCCTTTGCTCTATCCTCTATCAGCAAGTCAGAGATGCGTTCATGACATATCCTGC
TTCCAAGAAGCCCACAGTTTCATGGGGAAGGGAAAAATGAACGCAAGCATCTGGAACACCTGACTCTTTCAT
GAGAGATAAA

L2 and MIR at the 5'.

CLONE 155

[LZ34]

Driver: ORF2
Plasmid: AlurescueA70Du $A_{17}CATTACA_{18}GA_{17}CACACA_{18}$ (T)
Chromosome: 1
5' position: 65,860,631
Strand: minus
DR: AAAAGAACCCAGCAA
ENDOsite: TTTT/CT

Empty site:

CATAAAATAATCATATGTCCTACAATCAAGGGTCATTTATGATTCTGTCCATGTAGCTCTAATGCAAAAAAG
GATACCATAGTCAAATAGGAAAGCAGAGCAAAACAAAGTTAAGCAGGTTTCTGTCCTGTAAGACAGCCCAGA
GATTTTGATATGTTAAGGTGAGTTGTTAATTTCAAAAACAGAGATGTGTGAGGAAGCTTTTATCAAAAAAGC
ACCCACAGGGTGCTTTTATATATGAGCACTTACTGATATCTTGCAGAAAACTATGTTCCACTGAACACAATA
TAAAATATTTCCTTACCATGTACTGTGGGCGAAGGACTTCTTGTGGGCTGGAGAAAGGGGTCACTGGAAGCA
CTGGATGTGTTCAG↑**AAAAGAACCCAGCAA**↓ATCCTGACCTGATGGTTTAGAAGGTGCTCCAAATGGGTCAA
AGGTGGCACCTAGAAAACAATGGGAAAATATATTTATTTGTCCCATGAGTTCATTCACTAAATGTCACAGAA
TTGTCACTTCCTGTAGGGCTAACAGTGGACTCCAAGAATTTCCTATCTTTGTGGACAAAGGAGTAGTCTGGA
CTTACCTGTTGGATG

Filled site:

CATAAAATAATCATATGTCCTACAATCAAGGGTCATTTATGATTCTGTCCATGTAGCTCTAATGCAAAAAAG
GATACCATAGTCAAATAGGAAAGCAGAGCAAAACAAAGTTAAGCAGGTTTCTGTCCTGTAAGACAGCCCAGA
GATTTTGATATGTTAAGGTGAGTTGTTAATTTCAAAAACAGAGATGTGTGAGGAAGCTTTTATCAAAAAAGC
ACCCACAGGGTGCTTTTATATATGAGCACTTACTGATATCTTGCAGAAAACTATGTTCCACTGAACACAATA
TAAAATATTTCCTTACCATGTACTGTGGGCGAAGGACTTCTTGTGGGCTGGAGAAAGGGGTCACTGGAAGCA
CTGGATGTGTTCAGAAAAGAACCCAGCAA**GGCCGGGCGCGGTGGCTCACGCCTGTAATCCCAGCACTTTGGG
AGGCCGAGGCGGGCGGATCACGAGGTCAGGAGATCGAGACCATCCTGGCTAACACGGTGAAACCCCGTCTCT
ACTAAAAAAAAATACAAAAAATTAGCCGGGCGTGGTAGCGGGCGCCTGTAGTCCCAGCTACTCGGGAGGCTG
AGGCAGGGGAATGGCGTGAACCCGGGAGGCGGAGCTTGCAGTGAGCCGAGATCCCGCCACTGCACTCCA...[N
EOcassette][Atail]**AAAAGAACCCAGCAAATCCTGACCTGATGGTTTAGAAGGTGCTCCAAATGGG
TCAAAGGTGGCACCTAGAAAACAATGGGAAAATATATTTATTTGTCCCATGAGTTCATTCACTAAATGTCAC
AGAATTGTCACTTCCTGTAGGGCTAACAGTGGACTCCAAGAATTTCCTATCTTTGTGGACAAAGGAGTAGTC
TGGACTTACCTGTTGGATG

hAT-Charlie DNA element at the 5'.

CLONE 156

[LZ27]

Driver: ORF2
Plasmid: AlurescueA70Du $A_{17}CATTACA_{18}GA_{17}CACACA_{18}$ (T)
Chromosome: 1
5' position: 66,642,147
Strand: plus
DR: AAATAATGCAGTAA
ENDOsite: ATTT/AA

Empty site:

CCTCTTTGATTGCATAGTTGACTCAACAAAGGCAAGATTTCCCACGCTGAAGACAGATGGGAAAATTCCAAG
GTTGGAGGTCGACTGGCAAGAAGCAGGCAGCTAATCTAACGGATAGTGTCTTGAGAGAACCCCGCCTACAAA
ACCACAGATCTAACACAGCATGGGGGCTAGGCATCGCCTAAAATCTGCAGGTCCATCCACTCTTGGAACATT
CCCTGCCTGAGATCACAGCTACCCAAAGCAAGCTCTGTTGTGCCAATCACAGTGAATTAGTCAGATAGCTAA
GACTTCCCTCTT↑**AAATAATGCAGTAA**↓TTTAATTTTCTTGTGATAATTAGAAGGTTGAAGGAAATAAATCT
CTTAACTATAGCCCTCAATTTATTCAAATATAAGTGCAGATTATAGCACAGATGAAGCTATCATATTAAAAG
ATAATCAAGTTATAAAACTGGTATTTCTATCAAGGCTATTTGTATCAACTAATTAAAAGTTATCTTTTGAAT
TACCATGCCTGC

Filled site:

CCTCTTTGATTGCATAGTTGACTCAACAAAGGCAAGATTTCCCACGCTGAAGACAGATGGGAAAATTCCAAG
GTTGGAGGTCGACTGGCAAGAAGCAGGCAGCTAATCTAACGGATAGTGTCTTGAGAGAACCCCGCCTACAAA
ACCACAGATCTAACACAGCATGGGGGCTAGGCATCGCCTAAAATCTGCAGGTCCATCCACTCTTGGAACATT
CCCTGCCTGAGATCACAGCTACCCAAAGCAAGCTCTGTTGTGCCAATCACAGTGAATTAGTCAGATAGCTAA
GACTTCCCTCTTAAATAATGCAGTAA**GGCCGGGCGCGGTGGCTCACGCCTGTAATCCCAGCACTTTGGGAGG**
CCGAGGCGGGCGGATCACGAGGTCAGGAGATCGAGACCATCCTGGCTAACACGGTGAAACCCCGTCTCTACT
AAAAAAAAATACAAAAAATTAGCCGGGCGTGGTAGCGGGCGCCTGTAGTCCCAGCTACTCGGGAGGCTGAGG
CAGGGGAATGGCGTGAACCCGGGAGGCGGAGCTTGCAGTGAGCCGAGATCCCGCCA…[NEOcassette][
Atail]AAATAATGCAGTAATTTAATTTTCTTGTGATAATTAGAAGGTTGAAGGAAATAAATCTCTTAACTA
TAGCCCTCAATTTATTCAAATATAAGTGCAGATTATAGCACAGATGAAGCTATCATATTAAAAGATAATCAA
GTTATAAAACTGGTATTTCTATCAAGGCTATTTGTATCAACTAATTAAAAGTTATCTTTTGAATTACCATGC
CTGC

No repetitive sequences in the immediate proximity.

CLONE 157

[LZ15]

Driver: ORF2
Plasmid: AlurescueA70Du $A_{17}CATTACA_{18}GA_{17}CACACA_{18}$ (T)
Chromosome: 1
5’ position: 67,441,039
Strand: plus
DR: N/A
ENDOsite: N/A

Empty site:

GAGGATGATTTTTTTTAAAGTCCTGATTGCTGTCAGTCTCAGAGCAATATGGATCAGAGCTGTACAGTGCAA
ACGGGAAGAAAGCAAAATATATGTTGGGTAAGGGAGGGTATCATCAACATTTGTCCTTTAGGTGCAATCAGT
GCTACAGAATATTTACTTCAAGAGCAGAAATTTATGGGATAAAGAAGTATATTGCATCCAGAACACATACAG
ACACTGGATTTCCTTTTAAATGGTCAGTATTGCCGGTGCAGTGGCTCATGCCTGTTATCTCAGTACTTTGGG
AGGTTGAAGCATGCAGATTGCTTGAGCCTGGGAGTTCAAGAACAGCCTAGGCAACATGGCAAAACCCC↑A↓T
CTCTACAAAAAATAGAAAAATTACCTGAGTTTGGTGGCATGCAATTGTGGTCCCAGTTATTGAGGGTGCTGA
GGCAGGAGGATTGCTTGAGCCTGGGAGGGCAAGGCTGCAGTTAGCGGTTTGGTGCCACTGTACTCCAGCCTG
GGCAACAGAGCATGACACTATCTCAAAAAAAAAAGAAAAAGAAAGGAAAAAAGT

Filled site:

GAGGATGATTTTTTTTAAAGTCCTGATTGCTGTCAGTCTCAGAGCAATATGGATCAGAGCTGTACAGTGCAA
ACGGGAAGAAAGCAAAATATATGTTGGGTAAGGGAGGGTATCATCAACATTTGTCCTTTAGGTGCAATCAGT
GCTACAGAATATTTACTTCAAGAGCAGAAATTTATGGGATAAAGAAGTATATTGCATCCAGAACACATACAG
ACACTGGATTTCCTTTTAAATGGTCAGTATTGCCGGTGCAGTGGCTCATGCCTGTTATCTCAGTACTTTGGG
AGGTTGAAGCATGCAGATTGCTTGAGCCTGGGAGTTCAAGAACAGCCTAGGCAACATGGCAAAACCCC**AGCG**
GGCGCCTGTAGTCCCAGCTACTCGGGAGGCTGAGGCAGGGGAATGGCGTGAACCCGGGAGGCGGAGCTTGCA
GTGAGCCGAGATCCCGCC…[NEOcassette][Atail and 3' unsequenced]

Alu flanking the insertion site.

Alu insert truncation: missing between 151-152 bp of 5’ Alu sequence.

Sequence is shared between the Alu rescue vector and the genomic pre-insertion site.

CLONE 158

[GG34]

Driver: ORF2
Plasmid: AlurescueA70Du $A_{17}CATTACA_{18}GA_{17}CACACA_{18}$ (T)
Chromosome: 1
5' position: 85,808,537
Strand: plus
DR: AAAAAATGCAGAT
ENDOsite: TTTT/AA

Empty site:

TTATATTTTCAAGTTTATACAATGTTTAATTCTGGGCAGGAAAAACCTAAGAAGTTTCTAGTTTTCTATGGC
TTTAATTTGAATACTTTCTTTACTTTTACTGCTAATGTAATCGGGATATGTTATTTTGCACCTGTATGACAA
AAACGTTGGCTTCTTCAGGTGCACATTATTCGGGATTGTAAGCTGGGCACTTTTGGAAGTTGCAGATATCCT
TGAAAGCAGTGGCTCTCAACCTTGGCCTGTGCATTAGAATCACCTGCAGAGCTTTT↑**AAAAAATGCAGAT**↓G
CCTGGGTTCCATTCCAGACCCAGAATATCAGAATTTTCTGAGATAAACCCAGGGTAGTATGTTTTCAGAAAG
CTACTCAGATGCCTTTAAACCACTCTCACTAGGAACCACTAATTTAAGGTCACTTATAGACTTTTTCTTTCA
GAAGTTAGAGTCTTGGGTTGAAAGGCCCCTTATAAGGCTATGTGGCCAACCATCC

Filled site:

TTATATTTTCAAGTTTATACAATGTTTAATTCTGGGCAGGAAAAACCTAAGAAGTTTCTAGTTTTCTATGGC
TTTAATTTGAATACTTTCTTTACTTTTACTGCTAATGTAATCGGGATATGTTATTTTGCACCTGTATGACAA
AAACGTTGGCTTCTTCAGGTGCACATTATTCGGGATTGTAAGCTGGGCACTTTTGGAAGTTGCAGATATCCT
TGAAAGCAGTGGCTCTCAACCTTGGCCTGTGCATTAGAATCACCTGCAGAGCTTTTAAAAAATGCAGAT**GGC**
CGGGCGCGGTGGCTCACGCCTGTAATCCCAGCACTTTGGGAGGCCGAGGCGGGCGGATCACGAGGTCAGGAG
ATCGAGACCATCCTGGCTAACACGGTGAAACCCCGTCTCTACTAAAAAAAAAATACAAAAAATTAGCCGGGC
GTGGTAGCGGGCGCCTGTAGTCCCAGCTACTCGGGAGGCTGAGGCAGGGGAATGGCGTGAACCCGGGAGGCG
GAGCTTGCAGTGAGCCGAGATCCCGCCAC... [NEOcassette] [Atail] AAAAAATGCAGATGCCTGGG
TTCCATTCCAGACCCAGAATATCAGAATTTTCTGAGATAAACCCAGGGTAGTATGTTTTCAGAAAGCTACTC
AGATGCCTTTAAACCACTCTCACTAGGAACCACTAATTTAAGGTCACTTATAGACTTTTTCTTTCAGAAGTT
AGAGTCTTGGGTTGAAAGGCCCCTTATAAGGCTATGTGGCCAACCATCC

hAT-Charlie DNA element flanking the insertion site.

CLONE 159

[LZ64]

Driver: ORF2
Plasmid: AlurescueA70Du $A_{17}CATTACA_{18}GA_{17}CACACA_{18}$ (T)
Chromosome: 1
5' position: 117,549,581
Strand: plus
DR: AAAAATTTTCA
ENDOsite: TTTT/AT

Empty site:

TTGTCATAATCTTCCCAATTCTCCAAAATCCATTTCTAATGCCATCTCTTATGCCATCTCCATAAACCTATT
TCCCTCCCCTCAAAATGTAGTCCCTCTTCAAAATTTTTGAAGCACTTTTATTATATGTTTTCCATAGAGAGG
TGACTACTATCCACTTGATTGGATTATGAAATCCAGAAGTCAGAATCTAGCCTAAATTCATCTTCATATCTT
AGTCCCTCAAGCATGAGAAATACTATAAGTGGTTGAATAAATATTTATTGAATTGGCAAATAAAGCAGTAGA
AAAAGAAGTGCGTGTTTTCACAAT↑**AAAAATTTTCA**↓AACGTGGTACCCATCTTGCTGAAAAGTGATGGGAA
ACGTTCGAGAAGTGTTGCCTAGCCACCAAAAATAACATTGTTTGTAGAAACATGCCTTGATATATGTGGGTT
AGTGGTACCTCAAAAGCCACATTAAGAAAATATTTTTATTGTGAAATACAACATAAGAAAATTTTTATTATT
TGTTTAAAATAATTTTAAAAT

Filled site:

TTGTCATAATCTTCCCAATTCTCCAAAATCCATTTCTAATGCCATCTCTTATGCCATCTCCATAAACCTATT
TCCCTCCCCTCAAAATGTAGTCCCTCTTCAAAATTTTTGAAGCACTTTTATTATATGTTTTCCATAGAGAGG
TGACTACTATCCACTTGATTGGATTATGAAATCCAGAAGTCAGAATCTAGCCTAAATTCATCTTCATATCTT
AGTCCCTCAAGCATGAGAAATACTATAAGTGGTTGAATAAATATTTATTGAATTGGCAAATAAAGCAGTAGA
AAAAGAAGTGCGTGTTTTCACAATAAAAATTTTCA**GGCCGGGCGCGGTGGCTCACGCCTGTAATCCCAGCAC**
TTTGGGAGGCCGAGGCGGGCGGATCACGAGGTCAGGAGATCGAGACCATCCTGGCTAACACGGTGAAACCCC
GTCTCTACTAAAAAAAAATACAAAAAATTAGCCGGGCGTGGTAGCGGGCGCCTGTAGTCCCAGCTACTCGGG
AGGCTGAGGCAGGGGAATGGCGTGAACCCGGGAGGCGGAGCTTGCAGTGAGCCGAGATCCCGCCA... [NEOca
ssette] [Atail]AAAAATTTTCAAACGTGGTACCCATCTTGCTGAAAAGTGATGGGAAACGTTCGAGAA
GTGTTGCCTAGCCACCAAAAATAACATTGTTTGTAGAAACATGCCTTGATATATGTGGGTTAGTGGTACCTC
AAAAGCCACATTAAGAAAATATTTTTATTGTGAAATACAACATAAGAAAATTTTTATTATTTGTTTAAAATA
ATTTTAAAAT

No repetitive sequences in the immediate proximity.

CLONE 160

[GG50]

Driver: ORF2
Plasmid: AlurescueA70Du $A_{17}CATTACA_{18}GA_{17}CACACA_{18}$ (T)
Chromosome: 1
5' position: 151,963,026
Strand: minus
DR: AGAATTGTTTTCCA
ENDOsite: TTCT/GG

Empty site:

AGAAACACAAATAACTAGAATTCAGGCCAAATGCTTTAATAGTATTCCATTAGCTGGTGAACTCCTCAAAGG
CAAGCACCATGGAGAGCTTTGTGTGTGTGTGCATGTGTGTATGTGTGTGTGAACACCCTCATGCCTAACACC
ATGTCTGGGACATTCAAAGTAGGTGCTCAGGGAGGGAGAACCAAAAATGGAAAATTGAATTCCTCCTAAGGT
AACTAAATTTGTGGAGTCC↑**AGAATTGTTTTCCA**↓TAGAGAAAACTATGCAGAGAAAGATGTCTCCGCCTCC
CTTCTTAAGACCTGTTGCTCAGACAAACATGTAGAGGGCATGTATAGGCATCAGATTAGAATACTTCAATAA
AAGGTGAGTGGACAGCAAGGCTGTTATTATCCTGTGGAGCTCAGCAGTGAGATAGCAGCACTAGAAAAGCAA
AAAGTAGAAGATGGGATGA

Filled site:

AGAAACACAAATAACTAGAATTCAGGCCAAATGCTTTAATAGTATTCCATTAGCTGGTGAACTCCTCAAAGG
CAAGCACCATGGAGAGCTTTGTGTGTGTGTGCATGTGTGTATGTGTGTGTGAACACCCTCATGCCTAACACC
ATGTCTGGGACATTCAAAGTAGGTGCTCAGGGAGGGAGAACCAAAAATGGAAAATTGAATTCCTCCTAAGGT
AACTAAATTTGTGGAGTCCAGAATTGTTTTCCA**GCCGGGCGCGGTGGCTCACGCCTGTAATCCCAGCACTTT
GGGAGGCCGAGGCGGGCGGATCACGAGGTCAGGAGATCGAGACCATCCTGGCTAACACGGTGAAACCCCGTC
TCTACTAAAAAAAAATACAAAAAATTAGCCGGGCGTGGTAGCGGGCGCCTGTAGTCCCAGCTACTCGGGAGG
CTGAGGCAGGGGAATGGCGTGAACCCGGGAGGCGGAGCTTGCAGTGAGCCGAGATCCC…[NEOcassette
][Atail]**AGAATTGTTTTCCATAGAGAAAACTATGCAGAGAAAGATGTCTCCGCCTCCCTTCTTAAGACC
TGTTGCTCAGACAAACATGTAGAGGGCATGTATAGGCATCAGATTAGAATACTTCAATAAAAGGTGAGTGGA
CAGCAAGGCTGTTATTATCCTGTGGAGCTCAGCAGTGAGATAGCAGCACTAGAAAAGCAAAAAGTAGAAGAT
GGGATGA

No repetitive sequences in the immediate proximity.

CLONE 161

[LZ35]

Driver: ORF2
Plasmid: AlurescueA70Du $A_{17}CATTACA_{18}GA_{17}CACACA_{18}$ (T)
Chromosome: 1
5’ position: 182,982,875
Strand: plus
DR: ACTAAATCTTCCT
ENDOsite: TAGT/AA

Empty site:

TTTGTAGGGTATTTCTATTATGCCTATAAATTATGTTTTTACATTTTAGAAATTCAAAAAAACTTTATAAGA
TATTATTTATTAGCAAAACATCCATTCAAAGATTAATGTTTTGTTATTTTGGACATAGATATCAAACCTAGG
TGAATGTTAATTTGTGACTGATAGTATGACAACATACACATGAACCGATGTTTGACACTTGCTGTGTCTTTG
ATAAGTCAATATAATTTGAGAGGCAGTATTATGTAATGGTGAAGACAGAGACTGCAGCCAGACTGCCCGTGT
CTGAATCTCAGCTCTGCCACTTATTAGCAGTATAATCCTAAGTT↑**ACTAAATCTTCCT**↓ATGCCTCTGTTTT
CTCACTTATAAAATGGGATGATACTAAAACTATATATCTCATAGGATTATTGCAATGATTAAATAAGTTAAC
ATACACTGAGCACTTAAAATGTCATTGCTACAAACTCCAAATAAGTATTTGTTATTGTTGTACATAAGGGGT
AAGAACATTGACTCTAATGTCAGACATCCCCAGTTTGAATCCA

Filled site:

TTTGTAGGGTATTTCTATTATGCCTATAAATTATGTTTTTACATTTTAGAAATTCAAAAAAACTTTATAAGA
TATTATTTATTAGCAAAACATCCATTCAAAGATTAATGTTTTGTTATTTTGGACATAGATATCAAACCTAGG
TGAATGTTAATTTGTGACTGATAGTATGACAACATACACATGAACCGATGTTTGACACTTGCTGTGTCTTTG
ATAAGTCAATATAATTTGAGAGGCAGTATTATGTAATGGTGAAGACAGAGACTGCAGCCAGACTGCCCGTGT
CTGAATCTCAGCTCTGCCACTTATTAGCAGTATAATCCTAAGTTACTAAATCTTCCT**GGCCGGGCGCGGTGG**
CTCACGCCTGTAATCCCAGCACTTTGGGAGGCCGAGGCGGGCGGATCACGAGGTCAGGAGATCGAGACCATC
CTGGCTAACACGGTGAAACCCCGTCTCTACTAAAAAAAAATACAAAAAATTAGCCGGGCGTGGTAGCGGGCG
CCTGTAGTCCCAGCTACTCGGGAGGCTGAGGCAGGGGAATGGCGTGAACCCGGGAGGCGGAGCTTGCAGTGA
GCCGAGATCCCGCC… [NEOcassette] [Atail] ACTAAATCTTCCTATGCCTCTGTTTTCTCACTTAT
AAAATGGGATGATACTAAAACTATATATCTCATAGGATTATTGCAATGATTAAATAAGTTAACATACACTGA
GCACTTAAAATGTCATTGCTACAAACTCCAAATAAGTATTTGTTATTGTTGTACATAAGGGGTAAGAACATT
GACTCTAATGTCAGACATCCCCAGTTTGAATCCA

MIR flanking the insertion site.

CLONE 162

[LZ53]

Driver: ORF2
Plasmid: AlurescueA70Du $A_{17}CATTACA_{18}GA_{17}CACACA_{18}$ (T)
Chromosome: 1
5’ position: 186,433,913
Strand: plus
DR: AGAATTTTATTATCTC
ENDOsite: TTCT/GA

Empty site:

TGTAAAGAGGAGACATATCTTCCTTCATTGTCTTCTTACTATATGCATAATTTTAAATCTTTTCAAACATAT
TTTTGAAACCTATTTTACTTTGGATTATAGTATTCTTAATTTTATTTTATCATATTTGATTGCATACCCTTT
TTTCTACCTTTTTATAGTATCCTTTAACAAATCATATGGGCATTCACTGAGATGTTACACCATCAGCCACTT
TATCTTTCTCCTTTCCTAAAAATATGTGGAGATAGTTATTGTC↑**AGAATTTTATTATCTC**↓CTGATGTTATT
TATTTTAGAGAGTCTTTGGCAAAAGAATATATAACTTTTTTTTTTAAGAGAAGGGGTCTCATTATGTTAGCC
CAGGCTGGCCTTGAACTGCTGGGGTCAAGTAACTCTCCTGCCTCAGCCTCCCAAGTGGCTGGGATTACAGGC
ACCTGCCTTCTGTAATGCTTATTGCCTTCTATATATCTGTAGCACT

Filled site:

TGTAAAGAGGAGACATATCTTCCTTCATTGTCTTCTTACTATATGCATAATTTTAAATCTTTTCAAACATAT
TTTTGAAACCTATTTTACTTTGGATTATAGTATTCTTAATTTTATTTTATCATATTTGATTGCATACCCTTT
TTTCTACCTTTTTATAGTATCCTTTAACAAATCATATGGGCATTCACTGAGATGTTACACCATCAGCCACTT
TATCTTTCTCCTTTCCTAAAAATATGTGGAGATAGTTATTGTCAGAATTTTATTATCTC**GGCCGGGCGCGGT**
GGCTCACGCCTGTAATCCCAGCACTTTGGGAGGCCGAGGCGGGCGGATCACGAGGTCAGGAGATCGAGACCA
TCCTGGCTAACACGGTGAAACCCCGTCTCTACTAAAAAAAAATACAAAAAATTAGCCGGGCGTGGTAGCGGG
CGCCTGTAGTCCCAGCTACTCGGGAGGCTGAGGCAGGGGAATGGCGTGAACCCGGGAGGCGGAGCTTGCAGT
GAGCCGAGATCCCGCC...**[NEOcassette][Atail]**AGAATTTTATTATCTCCTGATGTTATTTATTTT
AGAGAGTCTTTGGCAAAAGAATATATAACTTTTTTTTTTAAGAGAAGGGGTCTCATTATGTTAGCCCAGGCT
GGCCTTGAACTGCTGGGGTCAAGTAACTCTCCTGCCTCAGCCTCCCAAGTGGCTGGGATTACAGGCACCTGC
CTTCTGTAATGCTTATTGCCTTCTATATATCTGTAGCACT

Alu at the 3'.

CLONE 163

[TZ2, TZ6]

Driver: ORF2
Plasmid: AlurescueA70Du $A_{17}CATTACA_{18}GA_{17}CACACA_{18}$ (T)
Chromosome: 1
5' position: 186,648,182
Strand: minus
DR: N/A
ENDOsite: N/A

Empty site:

CGAGGTGTATGTATGAGTGTGGGATTTGACCAGTATAAGTGCGATTGTACCCGGACAGGATTCTATGGAGAA
AACTGCTCAACACGTAAGTTTGTCCTTTGGTTGCCTCATTAGGAGTGGGGCTGGATACAGTTATCATTGTAT
AGATTTGTGTCTTATAANNAGTCCCATTAATTTCTCCCTCCCTTTCTTCGTCTTCTTGCAGCGGAATTTTTG
ACAAGAATAAAATTATTTCTGAAACCCACTCCAAACACAGTGCACTACATACTTACCCACTTCAAGGGATTT
TGGAACGTTGTGAATAACATTCCCTTCCTTCGAAATGCAATTATGAGTTATGTGTTGACATGTAAGTACGT↑
↓AAGTGTCTTTCTAAGGTTTTTAGCCTTCTCAAAGAAAAATATGCTTTATAATACTGTAAGCCTAATCTAAA
AACATATTTCCAAGCTTATCAAAAAGACTTTAAGATAGCTTTTAAGTTTGCCTTCCATCTTAATCGCCAAAA
ATATTGACATTTAGTCCCATCCAGTTTATACAGTCTGCTCACAACTCTGTATACCTC

Filled site:

CGAGGTGTATGTATGAGTGTGGGATTTGACCAGTATAAGTGCGATTGTACCCGGACAGGATTCTATGGAGAA
AACTGCTCAACACGTAAGTTTGTCCTTTGGTTGCCTCATTAGGAGTGGGGCTGGATACAGTTATCATTGTAT
AGATTTGTGTCTTATAANNAGTCCCATTAATTTCTCCCTCCCTTTCTTCGTCTTCTTGCAGCGGAATTTTTG
ACAAGAATAAAATTATTTCTGAAACCCACTCCAAACACAGTGCACTACATACTTACCCACTTCAAGGGATTT
TGGAACGTTGTGAATAACATTCCCTTCCTTCGAAATGCAATTATGAGTTATGTGTTGACATGTAAGTACGT**G**
GCCGGGCGCGGTGGCTCACGCCTGTAATCCCAGCACTTTGGGAGGCCGAGGCGGGCGGATCACGAGGTCAGG
AGATCGAGACCATCCTGGCTAACACGGTGAAACCCCGTCTCTACTAAAAAAAAATACAAAAAATTAGCCGGG
CGTGGTAGCGGGCGCCTGTAGTCCCAGCTACTCGGGAGGCTGAGGCAGGGGAATGGCGTGAACCCGGGAGGC
GGAGCTTGCAGTGAGCCGAGATCCCGCC…[NEOcassette][Atail and 3' unsequenced]

No repetitive sequences in the immediate proximity.

CLONE 164

[LZ44]

Driver: ORF2
Plasmid: AlurescueA70Du $A_{17}CATTACA_{18}GA_{17}CACACA_{18}$ (T)
Chromosome: 1
5' position: 205,596,728
Strand: plus
DR: AAGGAGGTGAATTT
ENDOsite: CCTT/AG

Empty site:

ACTCAGGAGGCTGAGGGAGGAGAATGGCGTGAACCTGGGAGGCAGAGTTTGCAGTGAGCTGAGATTGCGCCA
CTGCACTCCAGCCTGGGTGACAGAGCGAGACTCCGCCTCAAAAAAAAAAAAGAAAAGAAAATATTATAAACT
TAGATTGTGTTCATGCTTGCACAAAACCCACAGCATATTACACCT↑**AAGGAGGTGAATTT**↓TTTAAAACAAA
CAACACAACAAACACTATAAAAGTTAGGTCTGGGAAGACTAATTCTAACAACATTTAACTCACAGAAGATGG
GATTCTTAAGGGTCAACTATACCTACTTCCAAAAAGGGCAGAGATAAGAAACTGTAGTGGAAAATAGTTGGG
TTTCTATAACTTT

Filled site:

ACTCAGGAGGCTGAGGGAGGAGAATGGCGTGAACCTGGGAGGCAGAGTTTGCAGTGAGCTGAGATTGCGCCA
CTGCACTCCAGCCTGGGTGACAGAGCGAGACTCCGCCTCAAAAAAAAAAAAGAAAAGAAAATATTATAAACT
TAGATTGTGTTCATGCTTGCACAAAACCCACAGCATATTACACCTAAGGAGGTGAATTT**GGCCGGGCGCGGT**
GGCTCACGCCTGTAATCCCAGCACTTTGGGAGGCCGAGGCGGGCGGATCACGAGGTCAGGAGATCGAGACCA
TCCTGGCTAACACGGTGAAACCCCGTCTCTACTAAAAAAAAAATACAAAAAATTAGCCGGGCGTGGTAGCGG
GCGCCTGTAGTCCCAGCTACTCGGGAGGCTGAGGCAGGGGAATGGCGTGAACCCGGGAGGCGGAGCTTGCAG
TGAGCCGAGATCCCGCCACTGCACTCC... [NEOcassette] [Atail] AAGGAGGTGAATTTTTTAAAAC
AAACAACACAACAAACACTATAAAAGTTAGGTCTGGGAAGACTAATTCTAACAACATTTAACTCACAGAAGA
TGGGATTCTTAAGGGTCAACTATACCTACTTCCAAAAAGGGCAGAGATAAGAAACTGTAGTGGAAAATAGTT
GGGTTTCTATAACTTT

Alu at the 5'.

CLONE 165

[TZ4]

Driver: ORF2
Plasmid: AlurescueA70Du $A_{17}CATTACA_{18}GA_{17}CACACA_{18}$ (T)
Chromosome: 1
5’ position: 206,991,724
Strand: plus
DR: N/A
ENDOsite: N/A

Empty site:

CTTAACAATATTAGAATGTTCATCCTTTTTGAGTGCAAACAGAATCTTTACCAAGACAGACCATATTCTGGG
CCGATAAACTGGAAGTAGCTCAAATGTCCATCAGAAGGAGAATGGGCAAACAAATTGCGGCATAGCCATATA
ATGGCTACTATTAAGCATTAGAAAGAAATTAATTGGTCAA↑↓TACATGCAACACCATGGACAAATCTCAAGA
TAATTATGTTCAGTTGAAACCAGACAAAAAAGACTACATACTGTATGATTCTATTTACATAATATACTAGAA
AACCCAAATTAGTCTATACTTTCGGAAAGCAGATTAGTGCTTGCCTGAGCAGGGTTGGAGTGGGGATGTGGG
TGAGAGGGTAGGAGAAAAGGATTAAA

Filled site:

CTTAACAATATTAGAATGTTCATCCTTTTTGAGTGCAAACAGAATCTTTACCAAGACAGACCATATTCTGGG
CCGATAAACTGGAAGTAGCTCAAATGTCCATCAGAAGGAGAATGGGCAAACAAATTGCGGCATAGCCATATA
ATGGCTACTATTAAGCATTAGAAAGAAATTAATTGGTCAA**GGCCGGGCGCGGTGGCTCACGCCTGTAATCCC**
AGCACTTTGGGAGGCCGAGGCGGGCGGATCACGAGGTCAGGAGATCGAGACCATCCTGGCTAACACGGTGAA
ACCCCGTCTCTACTAAAAAAAAAATACAAAAAATTAGCCGGGCGTGGTAGCGGGCGCCTGTAGTCCCAGCTA
CTCGGGAGGCTGAGGCAGGGGAATGGCGTGAACCCGGGAGGCGGAGCTTGCAGTGAGCCGAGATCCCGCCAC
TGCACTCC…[NEOcassette][Atail and 3' unsequenced]

LINE-1 flanking the insertion site.

CLONE 166

[TZ5]

Driver: ORF2
Plasmid: AlurescueA70Du $A_{17}CATTACA_{18}GA_{17}CACACA_{18}$ (T)
Chromosome: 2
5' position: 10,342,071
Strand: minus
DR: N/A
ENDOsite: N/A

Empty site:

GATTTTTTTTTCAGTTGGATAAAATAAAAATGAAGGTTTGGGCTAGTTGTGAAAGGCTGATTTTAGAGGCAG
TTCTGTGTGTAAACATATTGGCTAAAAGGGGTATCCTCCAGTTTTTCTGTAAATTAAATTTTATTAAAATAA
AAGCACAGTGGGTTTCTCTTAGAGCACTAACCTGCTCTTTAACAAAAATTGTAAAGGGCTATAAAAAGTCTA
TAAAAATCTTACCTTGTGGTCAAATATTAAAATTGAGTAAATATGTCTATAAGGATTTATT↑↓AATTCGGTT
TAACATTCATAGTACACTAATGTAAAGGTGAAATTTGGCTTATTTGGTATAAAAATTATACAGAAAATATTG
TCAAATACGAAATGGTGCTTGGCTTTCTTTGGGCTATATTTGTGTAGATATGTTTTTGGTATGTGTTCCAAG
GTTGTGGGAGACTCCTACAATCCTGATATGTTTTAGTGTACATTATC

Filled site:

GATTTTTTTTTCAGTTGGATAAAATAAAAATGAAGGTTTGGGCTAGTTGTGAAAGGCTGATTTTAGAGGCAG
TTCTGTGTGTAAACATATTGGCTAAAAGGGGTATCCTCCAGTTTTTCTGTAAATTAAATTTTATTAAAATAA
AAGCACAGTGGGTTTCTCTTAGAGCACTAACCTGCTCTTTAACAAAAATTGTAAAGGGCTATAAAAAGTCTA
TAAAAATCTTACCTTGTGGTCAAATATTAAAATTGAGTAAATATGTCTATAAGGATTTATT**GGCCGGGCGCG
GTGGCTCACGCCTGTAATCCCAGCACTTTGGGAGGCCGAGGCGGGCGGATCACGAGGTCAGGAGATCGAGAC
CATCCTGGCTAACACGGTGAAACCCCGTCTCTACTAAAAAAAAAAATACAAAAAATTAGCCGGGCGTGGTAG
CGGGCGCCTGTAGTCCCAGCTACTCGGGAGGCTGAGGCAGGGGAATGGCGTGAACCCGGGAGGCGGAGCTTG
CAGTGAGCCGAGATCCCGCCACTGCACTCC…[NEOcassette][Atail and 3'
unsequenced]**

LTR flanking the insertion site.

CLONE 167

[GG9]

Driver: ORF2
Plasmid: AlurescueA70Du $A_{17}CATTACA_{18}GA_{17}CACACA_{18}$ (T)
Chromosome: 2
5' position: 56,240,461
Strand: plus
DR: AAAATTCTTAGTTTT
ENDOsite: TTTT/AG

Empty site:

AAAAGAAAGTAAATATTTTAAAGCCTAAATTTTTTTTAAGTTTAATGTTCAAAATACTGAACTGTACTAAAA
ATCACAATAAATCGCCATTCTTCCAAATAACGTTCCCTTTATGATTATGGGATTTATATAAACTAAATATTT
GCAGAAGAAATTTCTTCAACTATATTGTAGGGAGAATGTCATCTTTTCAGATATGCTGGTTGGGAATAATCA
CTACACTAGTGGAAATGTTTGCACAAGCCAGCATTGAAAAGAGACGAGATTTCCCTTTATCAGCTTCTCAAA
AATGACAACT↑**AAAATTCTTAGTTTT**↓GGGACAGGACAAAGGTCTAAGTCTGTCATTTTTTAAATGCCACAT
TTTTGAAATAACAATCAGGTGTTTCACTTTAAGTGTATTAAATTTAAGTGTATTAATATTTAATATAAATAT
TGAAATATAATTAGTTTATTCAGAAGCAAAACCCCAATCCTAATTTCAGCATATCTTAATAGAAAGCCTGGG
TTTATGAATCC

Filled site:

AAAAGAAAGTAAATATTTTAAAGCCTAAATTTTTTTTAAGTTTAATGTTCAAAATACTGAACTGTACTAAAA
ATCACAATAAATCGCCATTCTTCCAAATAACGTTCCCTTTATGATTATGGGATTTATATAAACTAAATATTT
GCAGAAGAAATTTCTTCAACTATATTGTAGGGAGAATGTCATCTTTTCAGATATGCTGGTTGGGAATAATCA
CTACACTAGTGGAAATGTTTGCACAAGCCAGCATTGAAAAGAGACGAGATTTCCCTTTATCAGCTTCTCAAA
AATGACAACTAAAATTCTTAGTTTT**GGCCGGGCGCGGTGGCTCACGCCTGTAATCCCAGCACTTTGGGAGGC**
CGAGGCGGGCGGATCACGAGGTCAGGAGATCGAGACCATCCTGGCTAACACGGTGAAACCCCGTCTCTACTA
AAAAAAAATACAAAAAATTAGCCGGGCGTGGTAGCGGGCGCCTGTAGTCCCAGCTACTCGGGAGGCTGAGGC
…**[NEOcassette][Atail]**AAAATTCTTAGTTTTGGGACAGGACAAAGGTCTAAGTCTGTCATTTTTT
AAATGCCACATTTTTGAAATAACAATCAGGTGTTTCACTTTAAGTGTATTAAATTTAAGTGTATTAATATTT
AATATAAATATTGAAATATAATTAGTTTATTCAGAAGCAAAACCCCAATCCTAATTTCAGCATATCTTAATA
GAAAGCCTGGGTTTATGAATCC

No repetitive sequences in the immediate proximity.

CLONE 168

[LZ67]

Driver: ORF2
Plasmid: AlurescueA70Du $A_{17}CATTACA_{18}GA_{17}CACACA_{18}$ (T)
Chromosome: 2
5' position: 69,430,365
Strand: plus
DR: AAGAAAAAGGAAG
ENDOsite: TCTT/AA

Empty site:

GCCATTGATTTCAGAGAATTTTAATTACCTCTTAACCTGTAGGAAAAACTTTTTCTCAGAATTATTTTATGT
TCAAACCAGCAATGACCAAAAGAACCAAGTTATCTACATATTGAAGTCTTACATGAAAGTCGTTTTTCTTGC
ACAATAAACAATTAAGTTTACATTAGCTCTTTATGTTGTGTAAATGGACATCTTCAGAATTCCTATCTGACA
CCAGGTATAAGGTCCCAACAGATCTGGAGGGAAAGTCTCCTGACCTTTTATAAATTTTTGTAAGAAACGGAT
CATTTT↑**AAGAAAAAGGAAG**↓AAAAAAGAGCAAATTATAGGAAATGTTCCTAGAGGGTGTGATTTATTCTGG
CACAGCCATCTTCCCTGGATGTTTGAATTCTCTGTAGTATTCATTCAGTTCTCACCCTTGGAAGCCTCTCCT
TTGCTGTCTCATTGGAGGGGGACATGAGTACTTAGAATTTTTGGCTGAGTTAGCTGAATTTAAGGAATAAAA
AGTTC

Filled site:

GCCATTGATTTCAGAGAATTTTAATTACCTCTTAACCTGTAGGAAAAACTTTTTCTCAGAATTATTTTATGT
TCAAACCAGCAATGACCAAAAGAACCAAGTTATCTACATATTGAAGTCTTACATGAAAGTCGTTTTTCTTGC
ACAATAAACAATTAAGTTTACATTAGCTCTTTATGTTGTGTAAATGGACATCTTCAGAATTCCTATCTGACA
CCAGGTATAAGGTCCCAACAGATCTGGAGGGAAAGTCTCCTGACCTTTTATAAATTTTTGTAAGAAACGGAT
CATTTTAAGAAAAAGGAAG**GGCCGGGCGCGGTGGCTCACGCCTGTAATCCCAGCACTTTGGGAGGCCGAGGC**
GGGCGGATCACGAGGTCAGGAGATCGAGACCATCCTGGCTAACACGGTGAAACCCCGTCTCTACTAAAAAAA
AAATACAAAAAATTAGCCGGGCGTGGTAGCGGGCGCCTGTAGTCCCAGCTACTCGGGAGGCTGAGGCAGGGG
AATGGCGTGAACCCGGGAGGCGGAGCTTGCAGTGAGCCGAGATCCCGCCACTGCACTCC… [NEOcassett
e] [Atail] AAGAAAAAGGAAGAAAAAAGAGCAAATTATAGGAAATGTTCCTAGAGGGTGTGATTTATTCT
GGCACAGCCATCTTCCCTGGATGTTTGAATTCTCTGTAGTATTCATTCAGTTCTCACCCTTGGAAGCCTCTC
CTTTGCTGTCTCATTGGAGGGGGACATGAGTACTTAGAATTTTTGGCTGAGTTAGCTGAATTTAAGGAATAA
AAAGTTC

No repetitive sequences in the immediate proximity.

CLONE 169

[LZ21]

Driver: ORF2
Plasmid: AlurescueA70Du $A_{17}CATTACA_{18}GA_{17}CACACA_{18}$ (T)
Chromosome: 2
5' position: 70,318,707
Strand: plus
DR: AGAAGTATATGCA
ENDOsite: TTCT/AT

Empty site:

CAGCAAGGCATAGGACGAATTGCTACTTGGAAAGATGAAAAGCAAGAATAGCAGTTGGCAAACTACCTCAAG
GAAGAAGCAGGTTCTAATATAGGCTGGCAAGTGGTATCAATAGGAATGCAAGGAAAGGAACTAGGCAATGGC
AGAGATATTGAAACTGGGTGTGGAGGCCAGAGGAAGATGTAAAAAGGGGGAGCAATGAATTCAGGCACGGTG
AT↑**AGAAGTATATGCA**↓GACAGAAATAGTCAACAAGTAGCTAGAAATAAGACTGAGGTGAAGTCAAAGCTGC
ATAGGAACAGAGGTAAATGAGGGGAAGGGACTAGAGATGGAGTTTTGGGGTATTTCGTTGGGTTGAAGGACC
TAGTTTTCTTATTCACTGGGTACCCTAGTACCTGTCACCATACCTGACACATAGCAGGGACTCAGTAAATAT
T

Filled site:

CAGCAAGGCATAGGACGAATTGCTACTTGGAAAGATGAAAAGCAAGAATAGCAGTTGGCAAACTACCTCAAG
GAAGAAGCAGGTTCTAATATAGGCTGGCAAGTGGTATCAATAGGAATGCAAGGAAAGGAACTAGGCAATGGC
AGAGATATTGAAACTGGGTGTGGAGGCCAGAGGAAGATGTAAAAAGGGGGAGCAATGAATTCAGGCACGGTG
ATAGAAGTATATGCA**GGCCGGGCGCGGTGGCTCACGCCTGTAATCCCAGCACTTTGGGAGGCCGAGGCGGGC**
GGATCACGAGGTCAGGAGATCGAGACCATCCTGGCTAACACGGTGAAACCCCGTCTCTACTAAAAAAAAAAA
AAAAATACAAAAAATTAGCCGGGCGTGGTAGCGGGCGCCTGTAGTCCCAGCTACTCGGGAGGCTGAGGCAGG
GGAATGGCGTGAACCCGGGAGGCGGAGCTTGCAGTGAGCCGAGATCCCGCC…**[NEOcassette][Atai**
l]AGAAGTATATGCAGACAGAAATAGTCAACAAGTAGCTAGAAATAAGACTGAGGTGAAGTCAAAGCTGCAT
AGGAACAGAGGTAAATGAGGGGAAGGGACTAGAGATGGAGTTTTGGGGTATTTCGTTGGGTTGAAGGACCTA
GTTTTCTTATTCACTGGGTACCCTAGTACCTGTCACCATACCTGACACATAGCAGGGACTCAGTAAATATT

L2 at the 3'.

CLONE 170

[GG1]

Driver: ORF2
Plasmid: AlurescueA70Du $A_{17}CATTACA_{18}GA_{17}CACACA_{18}$ (T)
Chromosome: 2
5’ position: 101,475,178
Strand: minus
DR: AAAAAAATAAAGTAGG
ENDOsite: TTTT/AA

Empty site:

GTGATT is followed by ↑**AAAAAAATAAAGTAGG**↓ in the sequence below.

AACAACTTATATTCTTGAGGCGTTGATTATGCAACCTTACTTAGTTTTACTAAATATCTTATTGAGCAGGAA
AATTACACTATGGAGGGCAAGGGCTGCTATATTTAAATCCAAATAAACAGGACGGGGCGTGCACACTGACGT
GTAACATGATCACCCTTAATGACTTAAACAACTAATGTCAGTAATTAGGACTATTTTCAGAGTACGGAGAAG
GTGATT↑**AAAAAAATAAAGTAGG**↓GAGTAATTAATTTCACATAATAGTCACACTGGGACCTATTGTGAGATC
TGACGTTAATGATGTATTTCTTTTCTGTAATTAATTTTTATGTGCAGTCCTGGGAACTCCCCGCCACCTTCT
GAATTGCTCCTTGCTCAGCACCCCTAACTCTTCCAGCCCCACCACATCAGGCCAGCCTTCCCCATAACGAAA
GGGAACAC

Filled site:

AACAACTTATATTCTTGAGGCGTTGATTATGCAACCTTACTTAGTTTTACTAAATATCTTATTGAGCAGGAA
AATTACACTATGGAGGGCAAGGGCTGCTATATTTAAATCCAAATAAACAGGACGGGGCGTGCACACTGACGT
GTAACATGATCACCCTTAATGACTTAAACAACTAATGTCAGTAATTAGGACTATTTTCAGAGTACGGAGAAG
GTGATTAAAAAAATAAAGTAGG**GGCCGGGCGCGGTGGCTCACGCCTGTAATCCCAGCACTTTGGGAGGCCGA**
GGCGGGCGGATCACGAGGTCAGGAGATCGAGACCATCCTGGCTAACACGGTGAAACCCCGTCTCTACTAAAA
AAAAAAATACAAAAAATTAGCCGGGCGTGGTAGCGGGCGCCTGTAGTCCCAGCTACTCGGGAGGCTGAGGCA
GGGGAATGGCGTGAACCCGGGAGGCGGAGCTTGCAGTGAGCCGAGA... [NEOcassette] [Atail]AAA
AAAATAAAGTAGGGAGTAATTAATTTCACATAATAGTCACACTGGGACCTATTGTGAGATCTGACGTTAATG
ATGTATTTCTTTTCTGTAATTAATTTTTATGTGCAGTCCTGGGAACTCCCCGCCACCTTCTGAATTGCTCCT
TGCTCAGCACCCCTAACTCTTCCAGCCCCACCACATCAGGCCAGCCTTCCCCATAACGAAAGGGAACAC

No repetitive sequences in the immediate proximity.

CLONE 171

[LZ29]

Driver: ORF2
Plasmid: AlurescueA70Du $A_{17}CATTACA_{18}GA_{17}CACACA_{18}$ (T)
Chromosome: 2
5' position: 189,995,636
Strand: plus
DR: AGAAATATAAGTGTT
ENDOsite: TTCT/AA

Empty site:

GCCACCATATTTAAATAGCCACCTTGGCTCTGAAAAGATTTCAAACAGATCTAGCTAGCATCTAGTATCTCT
TCTACACAGATAAGTTTCCATTCCATTTTACAAATGAAATATTAGAGTATAAACCAGTCTACAAACCTACCT
TTTACACAAGTGCCTGTGAAAAATTTAGTTTATAAAGCCCAAGTCACATCACAAATGCTTAATATATTTATT
TTTATTTATTCAACACATTATTGACTACCTTCTATATCTTAGGCACCATATCAATGTT↑**AGAAATATAAGTG
TT**↓AACAGACAGAAAAGATTTAGTCCTTAAAGAGCTTATACTCTAGTGGAAAGAAACAGGAAATAAGCAAAT
ATTATACTTACTAACCATACAATATGTCTAGTAGCAAGAAGTGTTACCAAAAATTCAGCAAGGTAAAGACAA
AGAGCTCCAGGAGAAAAGTGAATTGAGAGGAGAGCGGGGACTGAGCTTAATTTTGAATA

Filled site:

GCCACCATATTTAAATAGCCACCTTGGCTCTGAAAAGATTTCAAACAGATCTAGCTAGCATCTAGTATCTCT
TCTACACAGATAAGTTTCCATTCCATTTTACAAATGAAATATTAGAGTATAAACCAGTCTACAAACCTACCT
TTTACACAAGTGCCTGTGAAAAATTTAGTTTATAAAGCCCAAGTCACATCACAAATGCTTAATATATTTATT
TTTATTTATTCAACACATTATTGACTACCTTCTATATCTTAGGCACCATATCAATGTTAGAAATATAAGTGT
T**GGCCGGGCGCGGTGGCTCACGCCTGTAATCCCAGCACTTTGGGAGGCCGAGGCGGGCGGATCACGAGGTCA
GGAGATCGAGACCATCCTGGCTAACACGGTGAAACCCCGTCTCTACTAAAAAAAAATACAAAAAATTAGCCG
GGCGTGGTAGCGGGCGCCTGTAGTCCCAGCTACTCGGGAGGCTGAGGCAGGGGAATGGCGTGAACCCGGGAG
GCGGAGCTTGCAGTGAGCCGAGATCCCGCCACTGCACTCC…[NEOcassette][Atail]**AGAAATATA
AGTGTTAACAGACAGAAAAGATTTAGTCCTTAAAGAGCTTATACTCTAGTGGAAAGAAACAGGAAATAAGCA
AATATTATACTTACTAACCATACAATATGTCTAGTAGCAAGAAGTGTTACCAAAAATTCAGCAAGGTAAAGA
CAAAGAGCTCCAGGAGAAAAGTGAATTGAGAGGAGAGCGGGGACTGAGCTTAATTTTGAATA

L2 flanking the insertion site.

CLONE 173

[LZ52]

Driver: ORF2
Plasmid: AlurescueA70Du $A_{17}CATTACA_{18}GA_{17}CACACA_{18}$ (T)
Chromosome: 2
5' position: 210,800,221
Strand: minus
DR: AAAAGTGATAGGT
ENDOsite: TTTT/AA

Empty site:

AACAGGGCTTCAAATTGAGTGTCTTCATGGACTGGTAACTGGGTTGGGATATCGCACTCATTCTGTCCATGA
ACTACCAAGGTTTTAGTACCTACAGATGATAAAAATAATAACAAACTGACTTTTACTATAGAAAATTTGTAT
TTTAAAGCATAAAATATTAAAATATTATCAATAAGCTGATGTTTTTACTGTTAATTACAATTCTTAATTCAT
AGTTACATATATATTTTATTTATTTATTTTTAACCTGGAAATTTAAGATTAATATACATATATTTTAAATAT
ATTCACTTATT↑**AAAAGTGATAGGT**↓ATGGAGTTCCCAATACTTTTAAAAAATTTTTATTTTTAGTAGAGAC
GAGGTTTCGCCATGTTGTCCAAGCTGGTCTCAAACTCCTGACCTCAAGCAATCCACCTGCCTCGGCCTCCCA
AAGTGCGGAGATTACAGGAGTGAGCCACCGCGCTAGGCCAAGTTCTCAATACTTCTACCTGTCTTTTACATG
CATAATTGTA

Filled site:

AACAGGGCTTCAAATTGAGTGTCTTCATGGACTGGTAACTGGGTTGGGATATCGCACTCATTCTGTCCATGA
ACTACCAAGGTTTTAGTACCTACAGATGATAAAAATAATAACAAACTGACTTTTACTATAGAAAATTTGTAT
TTTAAAGCATAAAATATTAAAATATTATCAATAAGCTGATGTTTTTACTGTTAATTACAATTCTTAATTCAT
AGTTACATATATATTTTATTTATTTATTTTTAACCTGGAAATTTAAGATTAATATACATATATTTTAAATAT
ATTCACTTATTAAAAGTGATAGGT**GGCCGGGCGCGGTGGCTCACGCCTGTAATCCCAGCACTTTGGGAGGCC**
GAGGCGGGCGGATCACGAGGTCAGGAGATCGAGACCATCCTGGCTAACACGGTGAAACCCCGTCTCTACTAA
AAAAAAATACAAAAAATTAGCCGGGCGTGGTAGCGGGCGCCTGTAGTCCCAGCTACTCGGGAGGCTGAGGCA
GGGGAATGGCGTGAACCCGGGAGGCGGAGCTTGCAGTGAGCCGAGATCCCGCCACTGCACTCC… **[NEOcas**
sette] [Atail] AAAAGTGATAGGTATGGAGTTCCCAATACTTTTAAAAAATTTTTATTTTTAGTAGAGA
CGAGGTTTCGCCATGTTGTCCAAGCTGGTCTCAAACTCCTGACCTCAAGCAATCCACCTGCCTCGGCCTCCC
AAAGTGCGGAGATTACAGGAGTGAGCCACCGCGCTAGGCCAAGTTCTCAATACTTCTACCTGTCTTTTACAT
GCATAATTGTA

Alu flanking the insertion site.

CLONE 174

[GG26]

Driver: ORF2
Plasmid: AlurescueA70Du $A_{17}CATTACA_{18}GA_{17}CACACA_{18}$ (T)
Chromosome: 2
5' position: 213,446,348
Strand: plus
DR: AAAGAAGCACAGCA
ENDOsite: CTTT/AG

Empty site:

TATTCCATCATGTACATATACTACTTGTTAAAAATCCACTCACCTGTTGATGAGCACTTCAGTTGCTTCCAT
ATCTTGGCTCTTTTGAATAATGCTGCAATGGAAAAGAGAGGATAGTGGGAGGGATCTTTAGAGACCCAAACA
AAAATACGTCGACCCTAAAACTCTTGTTAGGCAAATTTGCTATGCTTGTACATGTGAGAGCAAACTATCTGT
TCTAGCATACTGAGAATCTACGTGTGGTAACTTCATAAACTTTAGTCAAAGCTGCTGTCTGGAGAGTAAAAT
AATCTTCT↑**AAAGAAGCACAGCA**↓AAGGAAATTTAAAACATTTAAATCTTTTTGGCAATCCATTTCAATCGT
TTTCTTTGATTCACTGAATTATTGGGATATTAATAGAAATTTAATATTTGTTCTTGACTTTTTGGAAAATCT
TGTGGTAAAGTAAGAATAGAAGAAAATTGCCTTAATATGATTAACAATGATCATTCTCAATAATAATAAAGT
TATACTGA

Filled site:

TATTCCATCATGTACATATACTACTTGTTAAAAATCCACTCACCTGTTGATGAGCACTTCAGTTGCTTCCAT
ATCTTGGCTCTTTTGAATAATGCTGCAATGGAAAAGAGAGGATAGTGGGAGGGATCTTTAGAGACCCAAACA
AAAATACGTCGACCCTAAAACTCTTGTTAGGCAAATTTGCTATGCTTGTACATGTGAGAGCAAACTATCTGT
TCTAGCATACTGAGAATCTACGTGTGGTAACTTCATAAACTTTAGTCAAAGCTGCTGTCTGGAGAGTAAAAT
AATCTTCTAAAGAAGCACAGCA**GGCCGGGCGCGGTGGCTCACGCCTGTAATCCCAGCACTTTGGGAGGCCGA**
GGCGGGCGGATCACGAGGTCAGGAGATCGAGACCATCCTGGCTAACACGGTGAAACCCCGTCTCTACTAAAA
AAAAAAATACAAAAAATTAGCCGGGCGTGGTAGCGGGCGCCTGTAGTCCCAGCTACTCGGGAGGCTGAGGCA
GGGGAATGGCGTGAACCCGGGAGGCGGAGCTTGC...**[NEOcassette][Atail]**AAAGAAGCACAGCAA
AGGAAATTTAAAACATTTAAATCTTTTTGGCAATCCATTTCAATCGTTTTCTTTGATTCACTGAATTATTGG
GATATTAATAGAAATTTAATATTTGTTCTTGACTTTTTGGAAAATCTTGTGGTAAAGTAAGAATAGAAGAAA
ATTGCCTTAATATGATTAACAATGATCATTCTCAATAATAATAAAGTTATACTGA

LINE-1 at the 3'.

CLONE 175

[TZ1]

Driver: ORF2
Plasmid: AlurescueA70Du $A_{17}CATTACA_{18}GA_{17}CACACA_{18}$ (T)
Chromosome: 2
5' position: 230,982,473
Strand: minus
DR: GAACTGGGCAGAGCC
ENDOsite: GTTC/AA

Empty site:

AAAAAGAACTGGGCAGAGCCAACCATAGCTCAGCAAGGACTACTGCCACTCTAGATTCCACCTCGGAGGGCA
GGGCATAGCAAAACAAAAGGCAGCAGACAGCTTCTGCAGACTTAAATGTCCCTGTCTGACAGCTCTGAAGAG
AGCACTGGTTGTTTCAGCACAGCGTTCAAGCTCTGAGAACGGACAGACTGCCTCCTCAAGTGGGTCCCTGAA
CCCCATGTAGCCTGACTGGGAGATACCTCCCAGTAGGGGCCAACAGACACTTCAAACAGGAGGGTGCCTGTC
TGGGACAAAGCTT↑**GAACTGGGCAGAGCC**↓AACCATAGCTCAGCAAGGACTACTGCCACTCTAGATTCCACC
TCGGAGGGCAGGGCATAGCAAAACAAAAGGCAGCAGACAGCTTCTGCAGACTTAAATGTCCCTGTCTGACAG
CTCTGAAGAGAGCACTGGTTGTTTCAGCACAGCGTTCAAGCTCTGAGAACGGACAGACTGCCTCCTCAAGTG
GGTCCCTGAACCCC

Filled site:

AAAAAGAACTGGGCAGAGCCAACCATAGCTCAGCAAGGACTACTGCCACTCTAGATTCCACCTCGGAGGGCA
GGGCATAGCAAAACAAAAGGCAGCAGACAGCTTCTGCAGACTTAAATGTCCCTGTCTGACAGCTCTGAAGAG
AGCACTGGTTGTTTCAGCACAGCGTTCAAGCTCTGAGAACGGACAGACTGCCTCCTCAAGTGGGTCCCTGAA
CCCCATGTAGCCTGACTGGGAGATACCTCCCAGTAGGGGCCAACAGACACTTCAAACAGGAGGGTGCCTGTC
TGGGACAAAGCTTGAACTGGGCAGAGCC**GGCCGGGCGCGGTGGCTCACGCCTGTAATCCCAGCACTTTGGGA
GGCCGAGGCGGGCGGATCACGAGGTCAGGAGATCGAGACCATCCTGGCTAACACGGTGAAACCCCGTCTCTA
CTAAAAAAAAAATACAAAAAATTAGCCGGGCGTGGTAGCGGGCGCCTGTAGTCCCAGCTACTCGGGAGGCTG
AGGCAGGGGAATGGCGTGAACCCGGGAGGCGGAGCTTGCAGTGAGCCGAGATCCCGCC…[NEOcassette
][Atail]**GAACTGGGCAGAGCCAACCATAGCTCAGCAAGGACTACTGCCACTCTAGATTCCACCTCGGAG
GGCAGGGCATAGCAAAACAAAAGGCAGCAGACAGCTTCTGCAGACTTAAATGTCCCTGTCTGACAGCTCTGA
AGAGAGCACTGGTTGTTTCAGCACAGCGTTCAAGCTCTGAGAACGGACAGACTGCCTCCTCAAGTGGGTCCC
TGAACCCC

LINE-1 flanking the insertion site.

CLONE 176

[LZ17]

Driver: ORF2
Plasmid: AlurescueA70Du $A_{17}CATTACA_{18}GA_{17}CACACA_{18}$ (T)
Chromosome: 2
5' position: 238,628,075
Strand: plus
DR: AGAACTGCTGTATT
ENDOsite: TTCT/GA

Empty site:

AAACCGTGTGTGTCTTAGAATCCACAATACATAGTAGAAAGAGTGACATCCTTTTCGGATATTGTGTGGAAA
TGATTTATCTTCTGATTCCCGAAGAGAGTGAGGTTCTGAAAGTAAGGGGAAAAGTAGATTGATTTAAATGGA
TCAGATGATAGAGGTTAGAAAAAAATTAAGACCCAGCTTCTGCCCCAAACACTGTTTTTACAACAAAAATGA
AGTATGCAAATTATTTTTGTGCTTAAGAACCTCCCACCCCAGAAAAAAACATTTTTACCCACTGGTTTAATT
AAAGCCTTTAAATGAAGGCAAAGAAAATATTTATGTGACATTTAGATC↑**AGAACTGCTGTATT**↓ATGGTGCA
GTGGGACTACCTAAGCTTTTTTAAAGCACTTTTCCTGTGTCCATCTGTCTCTAACCTTTTCATGCTGTTTCT
TCATTGTTAGGAAGACAGTGAGCGCTACTCTCGTAGATCCAGAAGAAACACATCGGTTAGTACCGTGTTCAT
TCATTACTTGGGCAATTTGATTGAATTCTAATTTATGCTTGCAGTGGC

Filled site:

AAACCGTGTGTGTCTTAGAATCCACAATACATAGTAGAAAGAGTGACATCCTTTTCGGATATTGTGTGGAAA
TGATTTATCTTCTGATTCCCGAAGAGAGTGAGGTTCTGAAAGTAAGGGGAAAAGTAGATTGATTTAAATGGA
TCAGATGATAGAGGTTAGAAAAAAATTAAGACCCAGCTTCTGCCCCAAACACTGTTTTTACAACAAAAATGA
AGTATGCAAATTATTTTTGTGCTTAAGAACCTCCCACCCCAGAAAAAAACATTTTTACCCACTGGTTTAATT
AAAGCCTTTAAATGAAGGCAAAGAAAATATTTATGTGACATTTAGATCAGAACTGCTGTATT**GGCCGGGCGC**
GGTGGCTCACGCCTGTAATCCCAGCACTTTGGGAGGCCGAGGCGGGCGGATCACGAGGTCAGGAGATCGAGA
CCATCCTGGCTAACACGGTGAAACCCCGTCTCTACTAAAAAAAAAATACAAAAAATTAGCCGGGCGTGGTAG
CGGGCGCCTGTAGTCCCAGCTACTCGGGAGGCTGAGGCAGGGGAATGGCGTGAACCCGGGAGGCGGAGCTTG
CAGTGAGCCGAGATCCCGCCACTGCA…[NEOcassette][Atail]AGAACTGCTGTATTATGGTGCAG
TGGGACTACCTAAGCTTTTTTAAAGCACTTTTCCTGTGTCCATCTGTCTCTAACCTTTTCATGCTGTTTCTT
CATTGTTAGGAAGACAGTGAGCGCTACTCTCGTAGATCCAGAAGAAACACATCGGTTAGTACCGTGTTCATT
CATTACTTGGGCAATTTGATTGAATTCTAATTTATGCTTGCAGTGGC

No repetitive sequences in the immediate proximity.

CLONE 177

[GG12]

Driver: ORF2
Plasmid: AlurescueA70Du $A_{17}CATTACA_{18}GA_{17}CACACA_{18}$ (T)
Chromosome: 2
5' position: 238,634,801
Strand: minus
DR: AAATAAATTTAATA
ENDOsite: ATTT/CA

Empty site:

CCGTGAAAATGTGCATAATAATAAATACATTACAAAGCCATCGTGAGGATTAAATGAGATGAAAATGAGTAT
CTCAAGCACCTAGAACAGTGCCCTCATGAATCACCAGCTGACTGGGTGGATACATGAATCCACATGGAAGGG
GCCTGGCATATGGGAGGTGCTAAACATGGTTCCTTATCCGGGGGACGGGTGCTGTATAGCACATATTTTGCA
GCCATGACATTAAAGTAGAAAAAAAAAAATTGAATTTCCAGACAGATTATAAATCCAGATTCATAAAACTTTG
ACTATCAAACTATATATTTATCTTTGCTGTAACTCAAAACAAATCAAGAAATCAATTTGAAGTTCTGAAACA
AAAGGAGTGTAGAAATCCTTCCATATTAAATTTGAGAACTCTATGAGTAACATTAAAACATTTCTG↑**AAATA**
AATTTAATA↓GTAAAAATCTTTGGAGATTGGCAAGAGTTTTACTCAGACTAATTTCCTTTCATGTCTTCAGC
AACAAGATTATTTGACATGAAAATGCATGTCAAGGACTTGCAAATTTTAGATCTCAATCCCTTGTTATGTCT
TCAGAAACTAGATTCCAGAAGAAATTAATAAAATAATGAAGGCTAATGTTAATCACTTTTAGATGA

Filled site:

CCGTGAAAATGTGCATAATAATAAATACATTACAAAGCCATCGTGAGGATTAAATGAGATGAAAATGAGTAT
CTCAAGCACCTAGAACAGTGCCCTCATGAATCACCAGCTGACTGGGTGGATACATGAATCCACATGGAAGGG
GCCTGGCATATGGGAGGTGCTAAACATGGTTCCTTATCCGGGGGACGGGTGCTGTATAGCACATATTTTGCA
GCCATGACATTAAAGTAGAAAAAAAAAAATTGAATTTCCAGACAGATTATAAATCCAGATTCATAAAACTTTG
ACTATCAAACTATATATTTATCTTTGCTGTAACTCAAAACAAATCAAGAAATCAATTTGAAGTTCTGAAACA
AAAGGAGTGTAGAAATCCTTCCATATTAAATTTGAGAACTCTATGAGTAACATTAAAACATTTCTGAAATAA
ATTTAATA**GGCCGGGCGCGGTGGCTCACGCCTGTAATCCCAGCACTTTGGGAGGCCGAGGCGGGCGGATCAC**
GAGGTCAGGAGATCGAGACCATCCTGGCTAACACGGTGAAACCCCGTCTCTACTAAAAAAAAATACAAAAAA
TTAGCCGGGCGTGGTAGCGGGCGCCTGTAGTCCCAGCTACTCGGGAGGCTGAGGCAGGGGAATGGCGTGAAC
CCGGGAGGCGGAGCTTGCAGTGAGCCGAGATCCCGCCACTGCA... [NEOcassette] [Atail] AAATAA
ATTTAATAGTAAAAATCTTTGGAGATTGGCAAGAGTTTTACTCAGACTAATTTCCTTTCATGTCTTCAGCAA
CAAGATTATTTGACATGAAAATGCATGTCAAGGACTTGCAAATTTTAGATCTCAATCCCTTGTTATGTCTTC
AGAAACTAGATTCCAGAAGAAATTAATAAAATAATGAAGGCTAATGTTAATCACTTTTAGATGA

MIR at the 5'.

CLONE 178

[TZ8]

Driver: ORF2
Plasmid: AlurescueA70Du $A_{17}CATTACA_{18}GA_{17}CACACA_{18}$ (T)
Chromosome: 3
5’ position: 24,844,836
Strand: minus
DR: N/A
ENDOsite: N/A

Empty site:

AAGCTTCTAAAGAGACTGATTCTATTGCAGTAGAGTACATATATACATATATTCCATATACACATAATATAT
AACATCGTAAACATTACTTATGTGTAATCTGATGAAAGATTTGTATTCTGAATATATAAAGAACTCTCAAAT
TCAAATAAAAAGATAAAAAAGCAAATTAAAAAAGCAGATAACAACTATAAACTGAACAAATCCAAATCAACA
ATCTTCAGCCTCTGGAGAGTCAAAAAAGGAAGCAGATTTAGGAGGGGAAATGAAACTTTGAAGAAGCACTTG
GCATAGGATACATTTTTCCATTTTTATAAATTTTGCCTGAGAGCAGACAAGTGTTAAGATATGGCAGGGGAC
ATTTAAAATTCTAGTAGA↑↓AAACCTGCCCCCTTTTTTTTTTTTTTTTTTTCTGAGACAGGGTCTTGCTCTG
TCGCTCAGGCTGGAGTGCAGTGGCATGGTGTGATCATGGCTCACTGCAGCCTGACATCCTGAGCTCAAGCGA
TCCTCCCGCCTCAGCCAATCATGTAGCTGGGAATACAGGCATGTGCCACCAGGCCCAGCTAATTTTCTGTAA
TTTT

Filled site:

AAGCTTCTAAAGAGACTGATTCTATTGCAGTAGAGTACATATATACATATATTCCATATACACATAATATAT
AACATCGTAAACATTACTTATGTGTAATCTGATGAAAGATTTGTATTCTGAATATATAAAGAACTCTCAAAT
TCAAATAAAAAGATAAAAAAGCAAATTAAAAAAGCAGATAACAACTATAAACTGAACAAATCCAAATCAACA
ATCTTCAGCCTCTGGAGAGTCAAAAAAGGAAGCAGATTTAGGAGGGGAAATGAAACTTTGAAGAAGCACTTG
GCATAGGATACATTTTTCCATTTTTATAAATTTTGCCTGAGAGCAGACAAGTGTTAAGATATGGCAGGGGAC
ATTTAAAATTCTAGTAGA**GGCCGGGCGCGGTGGCTCACGCCTGTAATCCCAGCACTTTGGGAGGCCGAGGCG**
GGCGGATCACGAGGTCAGGAGATCGAGACCATCCTGGCTAACACGGTGAAACCCCGTCTCTACTAAAAAAAA
ATACAAAAAATTAGCCGGGCGTGGTAGCGGGCGCCTGTAGTCCCAGCTACTCGGGAGGCTGAGGCAGGGGAA
TGGCGTGAACCCGGGAGGCGGAGCTTGCAGTGAGCCGAGATCCCGCC… [NEOcassette] [Atail and 3' unsequenced]

LINE-1 at the 5'; Alu at the 3'.

CLONE 179

[LZ79]

Driver: ORF2
Plasmid: AlurescueA70Du $A_{17}CATTACA_{18}GA_{17}CACACA_{18}$ (T)
Chromosome: 3
5' position: 33,345,902
Strand: minus
DR: AAGAATATACCAT
ENDOsite: TCTT/AG

Empty site:

ATTTCTTTATAAAGTTAAATATCTGTATAGCCTATAACCCTCAGAAATTCTACTTTCAGAGAAAATTGTGTA
CTTGTGCACCAGGAGACATCTACGAGGATGTCCAAAGCAGCACTGCTCTTACTGAAAAAACACTGGAAACAA
CCAAAATACTCAGTAAAGGGGGAAAAAATAAAATTTTAGTACATTCACACAGTAGAATATTATACAGCAGTA
AAAATGAATGAGCAACAGCCAATCAGAGCAACAAGAATCACCTTTCATAACAGAATTTTGAGTGAAAAACAT
TTCT↑**AAGAATATACCAT**↓ATAAAGACCAATCAGCACTGCATCAGGTAAGATTTAATAAGCGATAAAAGAAA
TCACTCTAGCTATTTCATGCATAAGGGATTTAGTACAGAGAACTAGACACTTACAAAATCATTGGGAGGCTG
AAGAAATGAGCCCAAAGAAGGGCATGGAAGGTGAGATGTGTAATATCTGTGTCTGTCCTCCCCCACATTATG
CTA

Filled site:

ATTTCTTTATAAAGTTAAATATCTGTATAGCCTATAACCCTCAGAAATTCTACTTTCAGAGAAAATTGTGTA
CTTGTGCACCAGGAGACATCTACGAGGATGTCCAAAGCAGCACTGCTCTTACTGAAAAAACACTGGAAACAA
CCAAAATACTCAGTAAAGGGGGAAAAAATAAAATTTTAGTACATTCACACAGTAGAATATTATACAGCAGTA
AAAATGAATGAGCAACAGCCAATCAGAGCAACAAGAATCACCTTTCATAACAGAATTTTGAGTGAAAAACAT
TTCTAAGAATATACCAT**GGCCGGGCGCGGTGGCTCACGCCTGTAATCCCAGCACTTTGGGAGGCCGAGGCGG
GCGGATCACGAGGTCAGGAGATCGAGACCATCCTGGCTAACACGGTGAAACCCCGTCTCTACTAAAAAAAAA
TACAAAAAATTAGCCGGGCGTGGTAGCGGGCGCCTGTAGTCCCAGCTACTCGGGAGGCTGAGGCAGGGGAAT
GGCGTGAACCCGGGAGGCGGAGCTTGCAGTGAGCCGAGATCCCGCCA...[NEOcassette][Atail]**AA
GAATATACCATATAAAGACCAATCAGCACTGCATCAGGTAAGATTTAATAAGCGATAAAAGAAATCACTCTA
GCTATTTCATGCATAAGGGATTTAGTACAGAGAACTAGACACTTACAAAATCATTGGGAGGCTGAAGAAATG
AGCCCAAAGAAGGGCATGGAAGGTGAGATGTGTAATATCTGTGTCTGTCCTCCCCCACATTATGCTA

LINE-1 at the 5'; LTR at the 3'.

CLONE 180

[TZ12]

Driver: ORF2
Plasmid: AlurescueA70Du $A_{17}CATTACA_{18}GA_{17}CACACA_{18}$ (T)
Chromosome: 3
5’ position: 120,085,269
Strand: minus
DR: N/A
ENDOsite: N/A

Empty site:

CACTTGTAGATAGCTTCATCCATGAAATCCACATTTCTTAGTAAAAGTGATAGAGTCCCCTCAGAAATTTCA
GCCTGGTCCACAGATACTCTTCCCTCATACAAGGAATTTTGATCTTCTAGTTGCTCTGTGTTATTGTGAAAC
TGGTAAACCAACTTCGAGAAAACTTCAAAAAAATCAAGGTACTTGAAAAGGAGGTAATATTCTTTATTATCC
TCTACCTCATATGCCTGTTTGATGTCTTCTCTTTCCCAAGAAAACTCAAGATTTTCAGTGCCCTTCACAAAG
GAGAAATGACAGGAGAGGGTAACATCAGTGAATGGGTGGGCCCGCAGCTCCTCTGTAGACACCGAGAATGTC
AAAGAAGAAATGAAGTTA↑↓ATAAAGACCAATCAGCACTGCATCAGGTAAGATTTAATAAGCGATAAAAGAA
ATCACTCTAGCTATTTCATGCATAAGGGATTTAGTACAGAGAACTAGACACTTACAAAATCATTGGGAGGCT
GAAGAAATGAGCCCAAAGAAGGGCATGGAAGGTGAGATGTGTAATATCTGTGTCTGTCCTCCCCCACATTAT
GCTA

Filled site:

CACTTGTAGATAGCTTCATCCATGAAATCCACATTTCTTAGTAAAAGTGATAGAGTCCCCTCAGAAATTTCA
GCCTGGTCCACAGATACTCTTCCCTCATACAAGGAATTTTGATCTTCTAGTTGCTCTGTGTTATTGTGAAAC
TGGTAAACCAACTTCGAGAAAACTTCAAAAAAATCAAGGTACTTGAAAAGGAGGTAATATTCTTTATTATCC
TCTACCTCATATGCCTGTTTGATGTCTTCTCTTTCCCAAGAAAACTCAAGATTTTCAGTGCCCTTCACAAAG
GAGAAATGACAGGAGAGGGTAACATCAGTGAATGGGTGGGCCCGCAGCTCCTCTGTAGACACCGAGAATGTC
AAAGAAGAAATGAAGTTA**GGCCGGGCGCGGTGGCTCACGCCTGTAATCCCAGCACTTTGGGAGGCCGAGGCG
GGCGGATCACGAGGTCAGGAGATCGAGACCATCCTGGCTAACACGGTGAAACCCCGTCTCTACTAAAAAAAA
ATACAAAAAATTAGCCGGGCGTGGTAGCGGGCGCCTGTAGTCCCAGCTACTCGGGAGGCTGAGGCAGGGGAA
TGGCGTGAACCCGGGAGGCGGAGCTTGCAGTGAGCCGAGATCCCGCC… [NEOcassette] [Atail and 3' unsequenced]**

LTR at the 3'.

CLONE 181

[LZ46]

Driver: ORF2
Plasmid: AlurescueA70Du $A_{17}CATTACA_{18}GA_{17}CACACA_{18}$ (T)
Chromosome: 3
5’ position: 124,745,971
Strand: plus
DR: ATGAGTATGT
ENDOsite: TCAT/AA

Empty site:

AAAAAAGAAAAACTATGTTAAACATGAGGCTCAATACTGAGATAAACTGGGTAGGTTGTACCTACTTCCACT
CCAAGTACCTGGCATTAGAGGAATGGCATTTTGGCTTGATGTCTTTTCCCCTTACCCACTATT↑**ATGAGTAT
GT**↓AACTAGCTGGCTATGCACATTGATTTATGTCTACTTTGAGTGACTTCCGAGTGTTAAGCTGGGTATGTG
GCACTCACATGAGGAAAGGTCTAAGAGAGGAGAGGAAGCTGCTGTCCTGTAGAAATGCAGCCAGGAGAGATC
TGGTCCTGAGGAATTTCTCTTCCTAGAAGGCGTGGTCAGCTCTCCCATCTCCAAAGCAG

Filled site:

AAAAAAGAAAAACTATGTTAAACATGAGGCTCAATACTGAGATAAACTGGGTAGGTTGTACCTACTTCCACT
CCAAGTACCTGGCATTAGAGGAATGGCATTTTGGCTTGATGTCTTTTCCCCTTACCCACTATTATGAGTATG
T**GGCCGGGCGCGGTGGCTCACGCCTGTAATCCCAGCACTTTGGGAGGCCGAGGCGGGCGGATCACGAGGTCA
GGAGATCGAGACCATCCTGGCTAACACGGTGAAACCCCGTCTCTACTAAAAAAAAAAATACAAAAAATTAGC
CGGGCGTGGTAGCGGGCGCCTGTAGTCCCAGCTACTCGGGAGGCTGAGGCAGGGGAATGGCGTGAACCCGGG
AGGCGGAGCTTGCAGTGAGCCGAGATCCCGCC…[NEOcassette][Atail]**ATGAGTATGTAACTAGC
TGGCTATGCACATTGATTTATGTCTACTTTGAGTGACTTCCGAGTGTTAAGCTGGGTATGTGGCACTCACAT
GAGGAAAGGTCTAAGAGAGGAGAGGAAGCTGCTGTCCTGTAGAAATGCAGCCAGGAGAGATCTGGTCCTGAG
GAATTTCTCTTCCTAGAAGGCGTGGTCAGCTCTCCCATCTCCAAAGCAG

No repetitive sequences in the immediate proximity.

CLONE 182

[LZ69]

Driver: ORF2
Plasmid: AlurescueA70Du $A_{17}CATTACA_{18}GA_{17}CACACA_{18}$ (T)
Chromosome: 3
5' position: 15,0736,931
Strand: minus
DR: AAGAGAATGTTTACTACA
ENDOsite: TCTT/GC

Empty site:

CAACTCATAGAAGAGCAAATCCAAATTATAAATACATGAAGCATATGAAAAATGCTCACATACTAGTAAGTA
TGAAAATTCAGTAAAGTAACATAGACTAGTATCCATTAAAGAGACTAACAAGACTTATTGGCAGGGGTAAAA
GGGAGAGGATACTCAAACATTGATGATGAAAATGTGAGTTGCTACAGACTTTTTGAAAAATCAATCTGGCAA
TATATAGTAAAATGAAAAATATAAATACTCTGCTTTAGTAATTCTACTCCTGGAAACCTAACCCATGGAAAT
AAAGGATAAAGGATAAAAAATATCTTTGC↑**AAGAGAATGTTTACTACA**↓GATGTGTGCACAACAAAAACAAT
TAAAACACAGCTGGAAACAAAAGGAATACATATTAGTAAAGGAATGGTTGAATAAATGGTAAAATATCCTCT
TCAAGGAAAGTTATGCTGCAAATTTGAAAAAAATAAATTAGAATTAAATCAGATAACAAGGATTTCCATGAA
GTTTTGTTGAATAAGGAAAGGAAAATGCAGAAA

Filled site:

CAACTCATAGAAGAGCAAATCCAAATTATAAATACATGAAGCATATGAAAAATGCTCACATACTAGTAAGTA
TGAAAATTCAGTAAAGTAACATAGACTAGTATCCATTAAAGAGACTAACAAGACTTATTGGCAGGGGTAAAA
GGGAGAGGATACTCAAACATTGATGATGAAAATGTGAGTTGCTACAGACTTTTTGAAAAATCAATCTGGCAA
TATATAGTAAAATGAAAAATATAAATACTCTGCTTTAGTAATTCTACTCCTGGAAACCTAACCCATGGAAAT
AAAGGATAAAGGATAAAAAATATCTTTGCAAGAGAATGTTTACTACA**GGCCGGGCGCGGTGGCTCACGCCTG
TAATCCCAGCACTTTGGGAGGCCGAGGCGGGCGGATCACGAGGTCAGGAGATCGAGACCATCCTGGCTAACA
CGGTGAAACCCCGTCTCTACTAAAAAAAAATACAAAAAATTAGCCGGGCGTGGTAGCGGGCGCCTGTAGTCC
CAGCTACTCGGGAGGCTGAGGCAGGGGAATGGCGTGAACCCGGGAGGCGGAGCTTGCAGTGAGCCGAGATCC
CGCCACTGCACTCC…[NEOcassette][Atail]**AAGAGAATGTTTACTACAGATGTGTGCACAACAAA
AACAATTAAAACACAGCTGGAAACAAAAGGAATACATATTAGTAAAGGAATGGTTGAATAAATGGTAAAATA
TCCTCTTCAAGGAAAGTTATGCTGCAAATTTGAAAAAAATAAATTAGAATTAAATCAGATAACAAGGATTTC
CATGAAGTTTTGTTGAATAAGGAAAGGAAAATGCAGAAA

LINE-1 flanking the insertion site.

CLONE 183

[GG39]

Driver: ORF2
Plasmid: AlurescueA70Du $A_{17}CATTACA_{18}GA_{17}CACACA_{18}$ (T)
Chromosome: 3
5' position: 152,229,496
Strand: plus
DR: AAAAATAATTTAGCAGATCA
ENDOsite: TTTT/AA

Empty site:

TTCAATTTCTTTCATTTTGTTGATAAAGTAAAACTGTATCTTAAAAGAGAAACTAGGCAATGTTATTTTATT
TAGATTATTTTAAAGTGAAGTACTCTGTTTTCAAATTATATTTCATGAAAAGAAAATGGAAGATTGTTTTTC
TAATCTTAGTTTTAATGACTGAGGTGATCTTTAGTGTATTGGAGATAAATTTCAAGTTTAAACTGTCTGGCT
GAAAGTTCATTGTAGAGGGTTTTTGAGGTTTTCTTTGCAAAGAACCCAAATGAAAAAAATATGACACGGATT
↑**AAAAATAATTTAGCAGATCA**↓TATGATATTATGCATCTCTTAAATGGTCTTACAAGTGATTACATAAGCTA
GGTTCTTTTTTGTTTTTATTTAAATTGTAAAATAGGAAAAGTTTATAAGTTTCTTTATTCTAATCTATTCAG
TGGCCTAGAATGGTTCCATAAGGCCAGTAAAACTGTAATCAGTGGGCTGAGCAGAGATTTATTTTTAAAGGA
GAGGAC

Filled site:

TTCAATTTCTTTCATTTTGTTGATAAAGTAAAACTGTATCTTAAAAGAGAAACTAGGCAATGTTATTTTATT
TAGATTATTTTAAAGTGAAGTACTCTGTTTTCAAATTATATTTCATGAAAAGAAAATGGAAGATTGTTTTTC
TAATCTTAGTTTTAATGACTGAGGTGATCTTTAGTGTATTGGAGATAAATTTCAAGTTTAAACTGTCTGGCT
GAAAGTTCATTGTAGAGGGTTTTTGAGGTTTTCTTTGCAAAGAACCCAAATGAAAAAAATATGACACGGATT
AAAAATAATTTAGCAGATCA**GGCCGGGCGCGGTGGCTCACGCCTGTAATCCCAGCACTTTGGGAGGCCGAGG
CGGGCGGATCACGAGGTCAGGAGATCGAGACCATCCTGGCTAACACGGTGAAACCCCGTCTCTACTAAAAAA
AAAATACAAAAAATTAGCCGGGCGTGGTAGCGGGCGCCTGTAGTCCCAGCTACTCGGGAGGCTGAGGCAGGG
GAATGGCGTGAACCCGGGAGGCGGAGCTTGCAGTGAGCCGAGATCCCGCC…[NEOcassette][Atail
]**AAAAATAATTTAGCAGATCATATGATATTATGCATCTCTTAAATGGTCTTACAAGTGATTACATAAGCTAG
GTTCTTTTTTGTTTTTATTTAAATTGTAAAATAGGAAAAGTTTATAAGTTTCTTTATTCTAATCTATTCAGT
GGCCTAGAATGGTTCCATAAGGCCAGTAAAACTGTAATCAGTGGGCTGAGCAGAGATTTATTTTTAAAGGAG
AGGAC

No repetitive sequences in the immediate proximity.

CLONE 184

[LZ16]

Driver: ORF2
Plasmid: AlurescueA70Du $A_{17}CATTACA_{18}GA_{17}CACACA_{18}$ (T)
Chromosome: 3
5’ position: 171,402,897
Strand: minus
DR: AAAAAAAAATTACAATGTT
ENDOsite: TTTT/GC

Empty site:

AACCTAAGCCCAGGGCTCTCATGTCCTGTTTAGCAGATGAGAGTTACAGTCTAGCTCACATGCCTGAATGTG
AGTCACCTGTGGCTTGCAGGTGCTAATGAACCTGAAAAATTATGCTAGACTTTGAGTAATGATTCCCAGCAT
TCTTTCATCTAGGGCAGGGGTGTCCAATCTTTTGGCTTCCGTGGACCACATTGGAAGAAGAATTGTCTTGGG
CCACACATAAAATATACTAACACTAATGATAGCTGATGAGCTAAAAAAAGTTGC↑**AAAAAAAAATTACAATG
TT**↓TAAGAAAGTTTGTGAATTCGTGTTGGGCCACATTCAAAGCCATCCCGGGCCGCATGTGGCCCACAGGCT
GTGGGTTGGACAAGCTTGGTCCAGCTACGCTGCATTTTAAAAAATGCAATAATCAACTACACACAATGAACT
AAAACTTCAGTGTAACTGTTAAATAAATTTGTGTCTATAAATCATAACAGCATCGATAT

Filled site:

AACCTAAGCCCAGGGCTCTCATGTCCTGTTTAGCAGATGAGAGTTACAGTCTAGCTCACATGCCTGAATGTG
AGTCACCTGTGGCTTGCAGGTGCTAATGAACCTGAAAAATTATGCTAGACTTTGAGTAATGATTCCCAGCAT
TCTTTCATCTAGGGCAGGGGTGTCCAATCTTTTGGCTTCCGTGGACCACATTGGAAGAAGAATTGTCTTGGG
CCACACATAAAATATACTAACACTAATGATAGCTGATGAGCTAAAAAAAGTTGCAAAAAAAAATTACAATGT
T**GGCCGGGCGCGGTGGCTCACGCCTGTAATCCCAGCACTTTGGGAGGCCGAGGCGGGCGGATCACGAGGTCA
GGAGATCGAGACCATCCTGGCTAACACGGTGAAACCCCGTCTCTACTAAAAAAAAATACAAAAAATTAGCCG
GGCGTGGTAGCGGGCGCCTGTAGTCCCAGCTACTCGGGAGGCTGAGGCAGGGGAATGGCGTGAACCCGGGAG
GCGGAGCTTGCAGTGAGCCGAGATCCCGCCACTGC…[NEOcassette][Atail]**AAAAAAAAATTACA
ATGTTTAAGAAAGTTTGTGAATTCGTGTTGGGCCACATTCAAAGCCATCCCGGGCCGCATGTGGCCCACAGG
CTGTGGGTTGGACAAGCTTGGTCCAGCTACGCTGCATTTTAAAAAATGCAATAATCAACTACACACAATGAA
CTAAAACTTCAGTGTAACTGTTAAATAAATTTGTGTCTATAAATCATAACAGCATCGATAT

hAT-Charlie DNA element flanking the insertion site.

CLONE 185

[TZ15]

Driver: ORF2
Plasmid: AlurescueA70Du $A_{17}CATTACA_{18}GA_{17}CACACA_{18}$ (T)
Chromosome: 3
5’ position: 181,561,035
Strand: plus
DR: N/A
ENDOsite: N/A

Empty site:

TATATACCGTATGTTCTGGAAAAGGCAAAACTATGGAGACAGTAAAAAGATCAGTGGTTGCCAGGGGTTGAA
AGGGGAAGGGAGGGATAAATAGGCACAGCACAAAGGATTTTCAGAACAGTGAAAGTATTCTGTATGACACTG
TAATAATGAATCATGTCATTATACATCTGTCAAAGCCCATGGAATGTACAACACCAAGAGTGAACCCTAATA
TAAGCTATGGTCTTTGGATGACAATGATGTGTCAATATAGGTTTGTCAATTATAACAAGTGTAGGCCGGGTG
CGATGGCTCAGGCCTGTAATCCCAGCACTTTGGGAGTCCGAGGCGGGCGGATCACCTGAGGTCAGGAGTTCG
AGACCAGGCTGGCCAACATAGTGAAACA↑↓CCGTCTCTACTAAAAATACAAAAATTAGCCAGGCCTGGTGGC
GCGTGCCTATAGTCCCAGTTACTCAGGAGGCTGAGGCAGGAGACTGACTTGAACCCAGGAGGCAGAGTGAGC
TGAGATCGTGCCATTGCACTCCAGCCTGGGTGATAGAGTGAGACTCCGTCTCAAAAAATAATAAATAAATAA
ATAAAACAAACATA

Filled site:

TATATACCGTATGTTCTGGAAAAGGCAAAACTATGGAGACAGTAAAAAGATCAGTGGTTGCCAGGGGTTGAA
AGGGGAAGGGAGGGATAAATAGGCACAGCACAAAGGATTTTCAGAACAGTGAAAGTATTCTGTATGACACTG
TAATAATGAATCATGTCATTATACATCTGTCAAAGCCCATGGAATGTACAACACCAAGAGTGAACCCTAATA
TAAGCTATGGTCTTTGGATGACAATGATGTGTCAATATAGGTTTGTCAATTATAACAAGTGTAGGCCGGGTG
CGATGGCTCAGGCCTGTAATCCCAGCACTTTGGGAGTCCGAGGCGGGCGGATCACCTGAGGTCAGGAGTTCG
AGACCAGGCTGGCCAACATAGTGAAACA**CGGAGCTTGCAGTGAGCCGAGATCCCGCC…[NEOcassette]**
[Atail and 3' unsequenced]

LINE-1 at the 5'.

Alu flanking the insertion site.

Inserted by recombination with a pre-existing truncated Alu: missing 107 bp of 5’ Alu sequence.

CLONE 186
[LZ43]

Driver: ORF2
Plasmid: AlurescueA70Du $A_{17}CATTACA_{18}GA_{17}CACACA_{18}$ (T)
Chromosome: 3
5' position: 184,205,951
Strand: minus
DR: CAAACTAACTATAAGGC
ENDOsite: TTTG/AA

Empty site:

CAGAAGTTCAAGGCTGCTGTGAGCTATGGTGGTGTCACTGCACTCCAGCCTGGGTAACAGAGAAAGACTCTA
CCTCTAGTAATAGTGATAATAATAATATGAAAAGCCCAAATAGCCAAAGCAACGTTAAGCAAAAAGAACAAA
GCCAGAGGCATCATATTACCTGACTT↑**CAAACTAACTATAAGGC**↓TACGGTAACCAAAACATCATGGTACTG
GTACAAAAACAGACATATAGACCAATGGAACAGAATAGAGAATCCAGAAATAAAGCCACACACCTATAACCA
TCAGATCTTCAACAAAGTTAACAAAAATAAGCAATGGGGAAAGGACTTCCTATTGAATAAATGGTGCTTGGA
TGGCCGGTTAGCCATATGCAAAAGAATGA

Filled site:

CAGAAGTTCAAGGCTGCTGTGAGCTATGGTGGTGTCACTGCACTCCAGCCTGGGTAACAGAGAAAGACTCTA
CCTCTAGTAATAGTGATAATAATAATATGAAAAGCCCAAATAGCCAAAGCAACGTTAAGCAAAAAGAACAAA
GCCAGAGGCATCATATTACCTGACTTCAAACTAACTATAAGGC**GGCCGGGCGCGGTGGCTCACGCCTGTAAT
CCCAGCACTTTGGGAGGCCGAGGCGGGCGGATCACGAGGTCAGGAGATCGAGACCATCCTGGCTAACACGGT
GAAACCCCGTCTCTACTAAAAAAAAATACAAAAAATTAGCCGGGCGTGGTAGCGGGCGCCTGTAGTCCCAGC
TACTCGGGAGGCTGAGGCAGGGGAATGGCGTGAACCCGGGAGGCGGAGCTTGCAGTGAGCCGAGATCCCGCC**
…**[NEOcassette][Atail]**CAAACTAACTATAAGGCTACGGTAACCAAAACATCATGGTACTGGTACA
AAAACAGACATATAGACCAATGGAACAGAATAGAGAATCCAGAAATAAAGCCACACACCTATAACCATCAGA
TCTTCAACAAAGTTAACAAAAATAAGCAATGGGGAAAGGACTTCCTATTGAATAAATGGTGCTTGGATGGCC
GGTTAGCCATATGCAAAAGAATGA

Alu at the 5'; LINE-1 flanking the insertion site.

CLONE 187
[LZ36]

Driver: ORF2
Plasmid: AlurescueA70Du $A_{17}CATTACA_{18}GA_{17}CACACA_{18}$ (T)
Chromosome: 3
5’ position: 197,689,496
Strand: minus
DR: AGAAAGGACATCTGTT
ENDOsite: TTCT/AA

Empty site:

TGTCTGATTTCAATATTTATTACAGAGGTACATAATCCAGGCAACTGAATACCCATATGGGGCAAAAAAAAA
AAAAAAGGATATTGATCCCCTATCTCACATCATATATAAAAATTAATTTCAGATGAATCATAATCCTAAAAG
TGAAGGCTTCTAGAACACAGGAGAATATTTTTATGACCTAGGGGGTATTTAAAGATTTCTT↑**AGAAAGGACA**
TCTGTT↓GGTGAGGATATGCAGCAACCATAACTAATATTTTGTTGATGGGGGTGTAAAATGGTAGAGTATTT
TGGAGAAAGGGTTAGTATTTTTTATAAACATTCACCTACCCTTGACCCAGTAATTTCACCCCTAGGTATTTA
TCCAAGAACAATGAAAACATATATCCACAAAAATTACTGTACAAGAATATTGACAGCAGCTTT

Filled site:

TGTCTGATTTCAATATTTATTACAGAGGTACATAATCCAGGCAACTGAATACCCATATGGGGCAAAAAAAAA
AAAAAAGGATATTGATCCCCTATCTCACATCATATATAAAAATTAATTTCAGATGAATCATAATCCTAAAAG
TGAAGGCTTCTAGAACACAGGAGAATATTTTTATGACCTAGGGGGTATTTAAAGATTTCTTAGAAAGGACAT
CTGTT**GGCCGGGCGCGGTGGCTCACGCCTGTAATCCCAGCACTTTGGGAGGCCGAGGCGGGCGGATCACGAG**
GTCAGGAGATCGAGACCATCCTGGCTAACACGGTGAAACCCCGTCTCTACTAAAAAAAAAATACAAAAAATT
AGCCGGGCGTGGTAGCGGGCGCCTGTAGTCCCAGCTACTCGGGAGGCTGAGGCAGGGGAATGGCGTGAACCC
GGGAGGCGGAGCTTGCAGTGAGCCGAGATCCCGCC...**[NEOcassette][Atail]**AGAAAGGACATCTG
TTGGTGAGGATATGCAGCAACCATAACTAATATTTTGTTGATGGGGGTGTAAAATGGTAGAGTATTTTGGAG
AAAGGGTTAGTATTTTTTATAAACATTCACCTACCCTTGACCCAGTAATTTCACCCCTAGGTATTTATCCAA
GAACAATGAAAACATATATCCACAAAAATTACTGTACAAGAATATTGACAGCAGCTTT

LINE-1 flanking the insertion site.

CLONE 188

[LZ76]

Driver: ORF2
Plasmid: AlurescueA70Du $A_{17}CATTACA_{18}GA_{17}CACACA_{18}$ (T)
Chromosome: 4
5’ position: 124,759,370
Strand: minus
DR: AAAAGTTACATCA
ENDOsite: TTTT/AA

Empty site:

GAGAAGTTTTGTTTGGATTTTTTTATAGACCTGTTAACCCTTGATGGATTTTACAGTCCTGCAGCACTGAAT
ATAAACTATGGATTTTTAAATTGGGAGTGGAAAGTGTTGTTGGTGGGCCCACTGTGGATCAAAAGTCTTGCA
TGTATGTGTTTCCTTTGACCCAGAAACTCTAGGTCTGGAAATATAAAGGAAAGAATGGAGAATAAATATATA
ATTATGTGGATCTTCATTGCAGAATGATTTACAAAGGGAAATATTGACCTCAAATAACGTGTTT↑**AAAAGTT
ACATCA**↓AAGTAAAATGTTGTAAAATACAAGATGAATAGACTATTTTGCAGTCCCAATAAAAGTAATATTCT
TAACATATTCATTCACATTGAAAATATTCATGCAATAGTTAGATGAAAAAGTGAGGTACAAAATGGTAGGTT
AAATGTGATCTCATTTTTATTGCAAAGAATATGTGTGTGTGTGTATATATATACATGTATATG

Filled site:

GAGAAGTTTTGTTTGGATTTTTTTATAGACCTGTTAACCCTTGATGGATTTTACAGTCCTGCAGCACTGAAT
ATAAACTATGGATTTTTAAATTGGGAGTGGAAAGTGTTGTTGGTGGGCCCACTGTGGATCAAAAGTCTTGCA
TGTATGTGTTTCCTTTGACCCAGAAACTCTAGGTCTGGAAATATAAAGGAAAGAATGGAGAATAAATATATA
ATTATGTGGATCTTCATTGCAGAATGATTTACAAAGGGAAATATTGACCTCAAATAACGTGTTTAAAAGTTA
CATCA**GGCCGGGCGCGGTGGCTCACGCCTGTAATCCCAGCACTTTGGGAGGCCGAGGCGGGCGGATCACGAG
GTCAGGAGATCGAGACCATCCTGGCTAACACGGTGAAACCCCGTCTCTACTAAAAAAAAATACAAAAAATTA
GCCGGGCGTGGTAGCGGGCGCCTGTAGTCCCAGCTACTCGGGAGGCTGAGGCAGGGGAATGGCGTGAACCCG
GGAGGCGGAGCTTGCAGTGAGCCGAGATCCCGCCACTGCACTCC…[NEOcassette][Atail]**AAAAG
TTACATCAAAGTAAAATGTTGTAAAATACAAGATGAATAGACTATTTTGCAGTCCCAATAAAAGTAATATTC
TTAACATATTCATTCACATTGAAAATATTCATGCAATAGTTAGATGAAAAAGTGAGGTACAAAATGGTAGGT
TAAATGTGATCTCATTTTTATTGCAAAGAATATGTGTGTGTGTGTATATATATACATGTATATG

LINE-1 flanking the insertion site.

CLONE 189

[LZ11,LZ39]

Driver: ORF2
Plasmid: AlurescueA70Du $A_{17}CATTACA_{18}GA_{17}CACACA_{18}$ (T)
Chromosome: 5
5’ position: 19,387,313
Strand: minus
DR: AAATGTTTATTATTT
ENDOsite: ATTT/AA

Empty site:

CCTGTAATCCCGGCTACTCAAGAGGCTGAAGCATAGAATTGCTTGAACCCAGGAGACTGAGGTTGCAGTGAG
CCAAGATTGCGCCACTGCACTCCAGCCTGGATGACAGAGCGAGACTCCATCTTAAAAAAAAAAAAAAAAAAA
AAAGAAAGAAAGAAAATAATGTTTACTTTCAAATATGTTAAAAATAACTCTAAAGCAAAGAAAGATTTGGTA
TCTTTATATTGTTGGATGTTTGGCATTACATGTATTTCTTCAAGTAATTATTCAATGTACTACATTACTTAT
GTTACCATTTAATTGTT↑**AAATGTTTATTATTT**↓ATGCTACTATAGCCAGAATTAGAAGATATTAATAGGTT
TATGTGTATGTTTAGAAAATGGAGGTCAAAATGACTACAAAATCCAGAAATAGTCACATCTTTTAAACATTT
AGCCTTCATCTTTTATTTGGTTTCTTTTGCATTCAATGCTTCAATTATGTTGGGATAAAAATTGATAGGATG
GCCTTCCTCTAATTTCAC

Filled site:

CCTGTAATCCCGGCTACTCAAGAGGCTGAAGCATAGAATTGCTTGAACCCAGGAGACTGAGGTTGCAGTGAG
CCAAGATTGCGCCACTGCACTCCAGCCTGGATGACAGAGCGAGACTCCATCTTAAAAAAAAAAAAAAAAAAA
AAAGAAAGAAAGAAAATAATGTTTACTTTCAAATATGTTAAAAATAACTCTAAAGCAAAGAAAGATTTGGTA
TCTTTATATTGTTGGATGTTTGGCATTACATGTATTTCTTCAAGTAATTATTCAATGTACTACATTACTTAT
GTTACCATTTAATTGTTAAATGTTTATTATTT**GGCCGGGCGCGGTGGCTCACGCCTGTAATCCCAGCACTTT**
GGGAGGCCGAGGCGGGCGGATCACGAGGTCAGGAGATCGAGACCATCCTGGCTAACACGGTGAAACCCCGTC
TCTACTAAAAAAAAATACAAAAAATTAGCCGGGCGTGGTAGCGGGCGCCTGTAGTCCCAGCTACTCGGGAGG
CTGAGGCAGGGGAATGGCGTGAACCCGGGAGGCGGAGCTTGCAGTGAGCCGAGATCCCGCCACTGCACTCC...
[NEOcassette][Atail]AAATGTTTATTATTTATGCTACTATAGCCAGAATTAGAAGATATTAATAG
GTTTATGTGTATGTTTAGAAAATGGAGGTCAAAATGACTACAAAATCCAGAAATAGTCACATCTTTTAAACA
TTTAGCCTTCATCTTTTATTTGGTTTCTTTTGCATTCAATGCTTCAATTATGTTGGGATAAAAATTGATAGG
ATGGCCTTCCTCTAATTTCAC

Alu at the 5'.

CLONE 190

[LZ56, GG24]

Driver: ORF2
Plasmid: AlurescueA70Du $A_{17}CATTACA_{18}GA_{17}CACACA_{18}$ (T)
Chromosome: 5
5’ position: 36,255,339
Strand: plus
DR: AGAGAAAGAGATTTA
ENDOsite: CTCT/AC

Empty site:

GGTTTGACTGGCTCCAGCTTCTGAACATCACTGTGCCTGCAGGATTCAATAACTGCACACCTTAGAACTAAT
CTCTTTCCTTAGAGACTAATCCTTCATCCCGACCCCCATCCCATGTCTGGGATGCCAAGGATAAATTATGGG
ACAAGTATTTTCTTTTAGAAGATCTTTTCACTTCACAAAATAATTTGCATCATATTCTTTCAAATAATAATA
AGATTACTATTTAGGTATGTGCTTGGCTCTGATATAAGTGCTTTACATGCTTTATCTCATTGAAATCTCTCA
GTCACTCAATAAGTCATGAACTACAGTTATTCCCATTTTAAATTGATATAAGTAGAGT↑**AGAGAAAGAGATT**
TA↓AAAACTTATCAAGGTCACTAGTTAGTCACTGCTGGAGCCAGAACTGGAGCCCATGCAGTCTAACAGGAG
AGCCTACTTGAAATGGGAAGCTCCTATAGAAATATTTTCAACAGTCTGATTTTTTGGTGTATTTTATAGCAC
ATCTCCAGAAAATGATGTGTACCTATATTATTATAAGTACACAGGAGAAATCCTGACAA

Filled site:

GGTTTGACTGGCTCCAGCTTCTGAACATCACTGTGCCTGCAGGATTCAATAACTGCACACCTTAGAACTAAT
CTCTTTCCTTAGAGACTAATCCTTCATCCCGACCCCCATCCCATGTCTGGGATGCCAAGGATAAATTATGGG
ACAAGTATTTTCTTTTAGAAGATCTTTTCACTTCACAAAATAATTTGCATCATATTCTTTCAAATAATAATA
AGATTACTATTTAGGTATGTGCTTGGCTCTGATATAAGTGCTTTACATGCTTTATCTCATTGAAATCTCTCA
GTCACTCAATAAGTCATGAACTACAGTTATTCCCATTTTAAATTGATATAAGTAGAGTAGAGAAAGAGATTT
A**GGCCGGGCGCGGTGGCTCACGCCTGTAATCCCAGCACTTTGGGAGGCCGAGGCGGGCGGATCACGAGGTCA**
GGAGATCGAGACCATCCTGGCTAACACGGTGAAACCCCGTCTCTACTAAAAAAAAATACAAAAAATTAGCCG
GGCGTGGTAGCGGGCGCCTGTAGTCCCAGCTACTCGGGAGGCTGAGGCAGGGGAATGGCGTGAACCCGGGAG
GCGGAGCTTGCAGTGAGCCGAGATCCCGCCACTGCA... **[NEOcassette] [Atail]** AGAGAAAGAGATT
TAAAAACTTATCAAGGTCACTAGTTAGTCACTGCTGGAGCCAGAACTGGAGCCCATGCAGTCTAACAGGAGA
GCCTACTTGAAATGGGAAGCTCCTATAGAAATATTTTCAACAGTCTGATTTTTTGGTGTATTTTATAGCACA
TCTCCAGAAAATGATGTGTACCTATATTATTATAAGTACACAGGAGAAATCCTGACAA

MIR flanking the insertion site.

CLONE 191

[GG47]

Driver: ORF2
Plasmid: AlurescueA70Du $A_{17}CATTACA_{18}GA_{17}CACACA_{18}$ (T)
Chromosome: 5
5’ position: 42,284,787
Strand: plus
DR: AAAAATTTAA
ENDOsite: TTTT/AA

Empty site:

TTTTTATTTCTACTGAGACAGAAAGAAAAATCATTGAATGAATTTAATGCAGAAATAGTATTTTTAAAAGCT
TTAGAGACCAAGTCTCAAGCTTAACATTTCAATTTTCATTTCACTGAAGAGTCACAAAAGACAAAAATAATA
AGTTTAATTATTATTAGAATTGTGTAGAAAAGATAAGAATGTACCATTCTTTCAAGTTACCTCTAATTAGAA
AGTTACCTTTCAAATTAAGTACTGGCTACTTAACTTGTGGACCAGTACAAAATAAAAATACAGGATTTTGT
TCAAAAAACCTTATTT↑**AAAAATTTAA**↓GACAGTGACAGCAGAGCATTTAAGAAAGCAAATAGTCCTTCTAA
GCACAGAGCCCTAATGCAAATGCAGAGCTTGCACAACCATGAAGCCAGCTCTGTCTCTAAGCTCACAATTCA
TTTAATCTGTTCTAGCATTGTATTTGATGTTTAACAAAGCAAACACCTAATGGAGGGGAAAATCTAGCTGTA
ACCAAAAAGGCA

Filled site:

TTTTTATTTCTACTGAGACAGAAAGAAAAATCATTGAATGAATTTAATGCAGAAATAGTATTTTTAAAAGCT
TTAGAGACCAAGTCTCAAGCTTAACATTTCAATTTTCATTTCACTGAAGAGTCACAAAAGACAAAAATAATA
AGTTTAATTATTATTAGAATTGTGTAGAAAAGATAAGAATGTACCATTCTTTCAAGTTACCTCTAATTAGAA
AGTTACCTTTCAAATTAAGTACTGGCTACTTAACTTGTGGACCAGTACAAAATAAAAATACAGGATTTTGT
TCAAAAAACCTTATTTAAAAATTTAA**GGCCGGGCGCGGTGGCTCACGCCTGTAATCCCAGCACTTTGGGAGG**
CCGAGGCGGGCGGATCACGAGGTCAGGAGATCGAGACCATCCTGGCTAACACGGTGAAACCCCGTCTCTACT
AAAAAAAAATACAAAAAATTAGCCGGGCGTGGTAGCGGGCGCCTGTAGTCCCAGCTACTCGGGAGGCTGAGG
CAGGGGAATGGCGTGAACCCGGGAGGCGGAGCTGCAGTGAGCCGAGATCCCGCC…[NEOcassette][At
ail]AAAAATTTAAGACAGTGACAGCAGAGCATTTAAGAAAGCAAATAGTCCTTCTAAGCACAGAGCCCTAA
TGCAAATGCAGAGCTTGCACAACCATGAAGCCAGCTCTGTCTCTAAGCTCACAATTCATTTAATCTGTTCTA
GCATTGTATTTGATGTTTAACAAAGCAAACACCTAATGGAGGGGAAAATCTAGCTGTAACCAAAAAGGCA

DNA element flanking the insertion site.

CLONE 192

[LZ75]

Driver: ORF2
Plasmid: AlurescueA70Du $A_{17}CATTACA_{18}GA_{17}CACACA_{18}$ (T)
Chromosome: 5
5’ position: 64,336,200
Strand: plus
DR: AAAAAAACAGATATA
ENDOsite: TTTT/AT

Empty site:

AACAAGAGAAAACTTCTATCTCAAAAAAAAAAGATAAAAATTAATATACTTAAATCATCTCCCCAAATCTCT
GTATGTCTACCATAGAGCTAAAGGAATTAAGTCATAATATTTATAGAACACTTACTATATGACCACTACTTT
ATGTCTATCATCTTATTTAATTATCCTAAGATTCTTGTGAAGCAGTTCTTGTTTTATTTTACAGATAGAAGA
CTGAGAACTTGTTTATTTCCAATAAGTACAAAAGAGTCATAAATAT↑**AAAAAAACAGATATA**↓GCCACCTGA
AAACTTGTTTCCAACAAACACAAAGTCATAAACGTCAAAAATAATAAAGGACTTAGTGCTAGGATGTGCTTC
AGTGGTTGAAATGGAACTCTGAGTTGGAAATATGTTTACATTTACACTTTGTTTAAATTCATTTTTTAACTG
AAAAGATAGTCATCTGGGACTAATATGCATTATTCAAGATATAATGG

Filled site:

AACAAGAGAAAACTTCTATCTCAAAAAAAAAAGATAAAAATTAATATACTTAAATCATCTCCCCAAATCTCT
GTATGTCTACCATAGAGCTAAAGGAATTAAGTCATAATATTTATAGAACACTTACTATATGACCACTACTTT
ATGTCTATCATCTTATTTAATTATCCTAAGATTCTTGTGAAGCAGTTCTTGTTTTATTTTACAGATAGAAGA
CTGAGAACTTGTTTATTTCCAATAAGTACAAAAGAGTCATAAATATAAAAAAACAGATATA**GGCCGGGCGCG**
GTGGCTCACGCCTGTAATCCCAGCACTTTGGGAGGCCGAGGCGGGCGGATCACGAGGTCAGGAGATCGAGAC
CATCCTGGCTAACACGGTGAAACCCCGTCTCTACTAAAAAAAAATACAAAAAATTAGCCGGGCGTGGTAGCG
GGCGCCTGTAGTCCCAGCTACTCGGGAGGCTGAGGCAGGGGAATGGCGTGAACCCGGGAGGCGGAGCTTGCA
GTGAGCCGAGATCCCGCCACTGCA...[NEOcassette][Atail]AAAAAAACAGATATAGCCACCTGAA
AACTTGTTTCCAACAAACACAAAGTCATAAACGTCAAAAATAATAAAGGACTTAGTGCTAGGATGTGCTTCA
GTGGTTGAAATGGAACTCTGAGTTGGAAATATGTTTACATTTACACTTTGTTTAAATTCATTTTTTAACTGA
AAAGATAGTCATCTGGGACTAATATGCATTATTCAAGATATAATGG

MIR at the 5'.

CLONE 193

[LZ30]

Driver: ORF2
Plasmid: AlurescueA70Du $A_{17}CATTACA_{18}GA_{17}CACACA_{18}$ (T)
Chromosome: 5
5’ position: 76,483,382
Strand: minus
DR: AAGAAAAGA
ENDOsite: TCTT/AG

Empty site:

ATATTTACAATGGAGAATATTGGCTGGCCACGTATGCTAGATGGTTGCTTTTTCATTTGTTTATGCCTCAGT
GTAATTGTCTTAATCTTCTAGGGAAAAGTCTAAAAATTCTTTCGAAGATGAAAACCAAGGCTGGAGTCAGAC
AACACCAGGGTCAGGGAATGCACACCTTGGTATGGGTAGGAGAAAGTAGACTGCTTGAGAGATAGAAGCTCT
GGATTCTAGTCCCCTCTTTGCCATTTTTGCCATATTTCATCAAATCTAAAATACCACTAATTGTAGGGAATG
CCATCACTTTATGTATCACT↑**AAGAAAAGA**↓ATGCTGCCCATCAAACAATGACAGTCCTACAAGATATGAAA
ATTGCTCCCACTGGCCGCTCACTGCTTTGATTTTTTTTTTCATCTATAGATTAGGCTATATCTCCTGGCTTC
AGTTTAGTTACTTGACATGTAAGAGGTAACCCCTTTGCACATCTCTCAATATTGAAACATAAATCTAGCTGT
AGGTACTTGTGGGTA

Filled site:

ATATTTACAATGGAGAATATTGGCTGGCCACGTATGCTAGATGGTTGCTTTTTCATTTGTTTATGCCTCAGT
GTAATTGTCTTAATCTTCTAGGGAAAAGTCTAAAAATTCTTTCGAAGATGAAAACCAAGGCTGGAGTCAGAC
AACACCAGGGTCAGGGAATGCACACCTTGGTATGGGTAGGAGAAAGTAGACTGCTTGAGAGATAGAAGCTCT
GGATTCTAGTCCCCTCTTTGCCATTTTTGCCATATTTCATCAAATCTAAAATACCACTAATTGTAGGGAATG
CCATCACTTTATGTATCACTAAGAAAAGA**GGCCGGGCGCGGTGGCTCACGCCTGTAATCCCAGCACTTTGGG**
AGGCCGAGGCGGGCGGATCACGAGGTCAGGAGATCGAGACCATCCTGGCTAACACGGTGAAACCCCGTCTCT
ACTAAAAAAAAATACAAAAAATTAGCCGGGCGTGGTAGCGGGCGCCTGTAGTCCCAGCTACTCGGGAGGCTG
AGGCAGGGGAATGGCGTGAACCCGGGAGGCGGAGCTTGCAGTGAGCCGAGATCCCGCC…[NEOcassette
][Atail]AAGAAAAGAATGCTGCCCATCAAACAATGACAGTCCTACAAGATATGAAAATTGCTCCCACTG
GCCGCTCACTGCTTTGATTTTTTTTTTCATCTATAGATTAGGCTATATCTCCTGGCTTCAGTTTAGTTACTT
GACATGTAAGAGGTAACCCCTTTGCACATCTCTCAATATTGAAACATAAATCTAGCTGTAGGTACTTGTGGG
TA

MIR at the 5'; DNA element flanking the insertion site.

CLONE 194
[TZ18,TZ19]

Driver: ORF2
Plasmid: AlurescueA70Du $A_{17}CATTACA_{18}GA_{17}CACACA_{18}$ (T)
Chromosome: 5
5' position: 131,066,328
Strand: plus
DR: N/A
ENDOsite: N/A

Empty site:

CCATTGATTAGGTATAAAGATCAATTTCCATCAAAACACAATCATCTCATTACCAGTAATTAAAAAGTCAAG
ATTCTATTTTGATACTGAGTAAATATAAAATACTTGTGATTAATGCAAACTTGGATGACACAGATAAATTTA
CATCAGATGACACTTGCTAAGCATGAGACCAAATACTGATCTTATATAAAATGTAATACATTTAGAGAGAAG
TTCCTATGTATTATACTTACTAATACGTTTAATTCCATATTTTACAAGAAGTCACATTACTTTTGAGAAATA
AAACTGTGTATTATGTATACTTCCAATCATACTTTAATTTCATACAACTGAAACTGTATTAGTATATTTATC
TTCATTTTTCAATAATAAAAAAAGTATGTA↑↓AAATAAGAAATTATTCTGATAACCCAATAAATCTGAATTG
GGAAATATAAGGAGAAAATACTCATGCAATCTATGCCTATAGCTTTCTACTTCTCCTATTCTAAGATTTATC
AAAGTTCATAAACATAATTGTTTATCTGTCTCTCCAGCTTCAATGCAAGCTCTAGGAAGATAAGGACTAATT
GCTATTATATCTCCAC

Filled site:

CCATTGATTAGGTATAAAGATCAATTTCCATCAAAACACAATCATCTCATTACCAGTAATTAAAAAGTCAAG
ATTCTATTTTGATACTGAGTAAATATAAAATACTTGTGATTAATGCAAACTTGGATGACACAGATAAATTTA
CATCAGATGACACTTGCTAAGCATGAGACCAAATACTGATCTTATATAAAATGTAATACATTTAGAGAGAAG
TTCCTATGTATTATACTTACTAATACGTTTAATTCCATATTTTACAAGAAGTCACATTACTTTTGAGAAATA
AAACTGTGTATTATGTATACTTCCAATCATACTTTAATTTCATACAACTGAAACTGTATTAGTATATTTATC
TTCATTTTTCAATAATAAAAAAAGTATGTA**GGCCGGGCGCGGTGGCTCACGCCTGTAATCCCAGCACTTTGG
GAGGCCGAGGCGGGCGGATCACGAGGTCAGGAGATCGAGACCATCCTGGCTAACACGGTGAAACCCCGTCTC
TACTAAAAAAAAATACAAAAAATTAGCCGGGCGTGGTAGCGGGCGCCTGTAGTCCCAGCTACTCGGGAGGCT
GAGGCAGGGGAATGGCGTGAACCCGGGAGGCGGAGCTTGCAGTGAGCCGAGATCCCGCC…[NEOcassett
e][Atail and 3' unsequenced]**

L2 at the 3'.

CLONE 195

[LZ78]

Driver: ORF2
Plasmid: AlurescueA70Du $A_{17}CATTACA_{18}GA_{17}CACACA_{18}$ (T)
Chromosome: 5
5' position: 134,356,756
Strand: plus
DR: AGAAATGGTGTGCTTA
ENDOsite: TTCT/AA

Empty site:

CTTAAAAAAAATTGGTCCATTTTGCGTGAAAGCCAAGTTCAAATTACTGTTACTTTCCCCTTTATGTTGGAA
ACTTAAAAAAGGATAGGGGTGGGAGCTGAGGGGGACACAGGGGACAGATACAGGAGGCGAGGCAGAGGAGGG
GGAAAGAGAGAGAACGGTGTATGGATGAAGAAGAAAAAAGCGAGCCTTCTTGTTTCAGTAAAAATGTATTTC
TGGAATGATGTTTCAGTTTACTCAGGCAGAAAAATT↑**AGAAATGGTGTGCTTA**↓TTAAACAGGCTAAGTAAG
CATGACTATTTTTAGTAGTGTAGTAAAACCTTGGTAATTTGGACCTCTTTAATAAAGATTCCATGATCATGG
TGGCTGGCAGGCACACACTGTGTGTGTGTGTGTTGTGTGTGTGTCAGGGCAAGGTAGGGGTGCAGATCGGTT
GCTGAGTTGGAGGCTGGGGAGCGGGGAGGGTGCAAGAA

Filled site:

CTTAAAAAAAATTGGTCCATTTTGCGTGAAAGCCAAGTTCAAATTACTGTTACTTTCCCCTTTATGTTGGAA
ACTTAAAAAAGGATAGGGGTGGGAGCTGAGGGGGACACAGGGGACAGATACAGGAGGCGAGGCAGAGGAGGG
GGAAAGAGAGAGAACGGTGTATGGATGAAGAAGAAAAAAGCGAGCCTTCTTGTTTCAGTAAAAATGTATTTC
TGGAATGATGTTTCAGTTTACTCAGGCAGAAAAATTAGAAATGGTGTGCTTA**GGCCGGGCGCGGTGGCTCAC
GCCTGTAATCCCAGCACTTTGGGAGGCCGAGGCGGGCGGATCACGAGGTCAGGAGATCGAGACCATCCTGGC
TAACACGGTGAAACCCCGTCTCTACTAAAAAAAAATACAAAAAATTAGCCGGGCGTGGTAGCGGGCGCCTGT
AGTCCCAGCTACTCGGGAGGCTGAGGCAGGGGAATGGCGTGAACCCGGGAGGCGGAGCTTGCAGTGAGCCGA
GATCCCGCCACTGCACTCCA...[NEOcassette][Atail]**AGAAATGGTGTGCTTATTAAACAGGCTAA
GTAAGCATGACTATTTTTAGTAGTGTAGTAAAACCTTGGTAATTTGGACCTCTTTAATAAAGATTCCATGAT
CATGGTGGCTGGCAGGCACACACTGTGTGTGTGTGTGTTGTGTGTGTGTCAGGGCAAGGTAGGGGTGCAGAT
CGGTTGCTGAGTTGGAGGCTGGGGAGCGGGGAGGGTGCAAGAA

No repetitive sequences in the immediate proximity.

CLONE 196

[LZ81]

Driver: ORF2
Plasmid: AlurescueA70Du $A_{17}CATTACA_{18}GA_{17}CACACA_{18}$ (T)
Chromosome: 5
5' position: 141,379,654
Strand: minus
DR: AAAAATTAGCCAAGCA
ENDOsite: TTTT/AA

Empty site:

AAAAAGGGTAGGAATGAACAGATGAGCAATAAATTGTCATGTTGGGGCTGTACCTGAGATCCAAACGAGGAT
ACAGCTGTTATAAAGATTAAGGCAGATTTGTGATGAGGGATATTATTGCAAGGTATGCAAAAATACATTTAG
GTTTTTTTTTTTTTTTTAAAGTTAATATAATGGAATATCTTAGGTCAATATAGCCAATTAAGGAGGTAGATA
CAGAAGTTAAAAGAAGGGTTTAATTTTTTTAACTATTTTGAGGCTGGGTGCAGTGGCTCATGCCTATAATCC
CAGTGCTTTGGGAGGCCAAAGCAGGAGGATCACCTGAGATCAGCAGTTCAAAACCAGCCTGGGCAACATAAC
AAGACCCTGTCTCTACGAAATTTTT↑**AAAAATTAGCCAAGCA**↓TGGTGGTGTATGCCTGTAGTCCCAGCCAC
TCAGAAGGCTGAGACAAGAGGATTGCTTGAGCCCAGGAGTTCAAGACTGCTGTGAACTATAATCGCATCACT
GTACCCCAACTCTGGACAACAGAACAAGACCCCATCTCAAAAAAATATATTTTTTTGATATAAATCCAATGA
TTTTTATGTTTATAGAATACTGCACTT

Filled site:

AAAAAGGGTAGGAATGAACAGATGAGCAATAAATTGTCATGTTGGGGCTGTACCTGAGATCCAAACGAGGAT
ACAGCTGTTATAAAGATTAAGGCAGATTTGTGATGAGGGATATTATTGCAAGGTATGCAAAAATACATTTAG
GTTTTTTTTTTTTTTTTAAAGTTAATATAATGGAATATCTTAGGTCAATATAGCCAATTAAGGAGGTAGATA
CAGAAGTTAAAAGAAGGGTTTAATTTTTTTAACTATTTTGAGGCTGGGTGCAGTGGCTCATGCCTATAATCC
CAGTGCTTTGGGAGGCCAAAGCAGGAGGATCACCTGAGATCAGCAGTTCAAAACCAGCCTGGGCAACATAAC
AAGACCCTGTCTCTACGAAATTTTTAAAAATTAGCCAAGCA**GGCCGGGCGCGGTGGCTCACGCCTGTAATCC**
CAGCACTTTGGGAGGCCGAGGCGGGCGGATCACGAGGTCAGGAGATCGAGACCATCCTGGCTAACACGGTGA
AACCCCGTCTCTACTAAAAAAAAAATACAAAAAATTAGCCGGGCGTGGTAGCGGGCGCCTGTAGTCCCAGCT
ACTCGGGAGGCTGAGGCAGGGGAATGGCGTGAACCCGGGAGGCGGAGCTTGCAGTGAGCCGAGATCCCGCCA
CTGCACT…[NEOcassette][Atail]AAAAATTAGCCAAGCATGGTGGTGTATGCCTGTAGTCCCAGC
CACTCAGAAGGCTGAGACAAGAGGATTGCTTGAGCCCAGGAGTTCAAGACTGCTGTGAACTATAATCGCATC
ACTGTACCCCAACTCTGGACAACAGAACAAGACCCCATCTCAAAAAAATATATTTTTTTGATATAAATCCAA
TGATTTTTATGTTTATAGAATACTGCACTT

Alu flanking the insertion site.

CLONE 197

[LZ45]

Driver: ORF2
Plasmid: AlurescueA70Du $A_{17}CATTACA_{18}GA_{17}CACACA_{18}$ (T)
Chromosome: 5
5’ position: 148,459,878
Strand: plus
DR: AAACATTAAGCTG
ENDOsite: GTTT/AA

Empty site:

AAATTGTGACAGCAAAAATATAAATGGGGGGAATATAAAAGTCTAGAGTATTCACATGCAACTAAAGTTAAG
TTGTTATCTGTTTAACACAGTCTATTACAACTACAAGATGTTTTAGATAAGCTCCATGGTAACCACAAAACA
AAAAGCCACAGCAGATACACAAATGAGAAAGAGAAAGAAATAAAAACTTAACACTACAGAAAATCACCAAAT
CACTAAGACAGTAAGAGAGGAAGAAAGAAACAAAGGATCTACAAAACAACCAGAAAACAATTTAAAAAATGG
CAGGAGTAAGTCCTTATTTATCAGTAATAACTTTGAATGTAAATAGATT↑**AAACATTAAGCTG**↓GATGTGGC
GGCGCAAACCCATAATTCCAACTACTCAGGAGACTGATGCGAGAAGATTCCTTGAGCACAGAAGTTTGAGAC
CAGCCTGGACAACACAGTAAGACCCCCATCTCAAGAAAAAATAAGACATTAAGTGGCTGACATAGAGCGGGT
AAAAAAATAAATAAATAAATAAATAAATAAATAAATAAATAAATAAAA

Filled site:

AAATTGTGACAGCAAAAATATAAATGGGGGGAATATAAAAGTCTAGAGTATTCACATGCAACTAAAGTTAAG
TTGTTATCTGTTTAACACAGTCTATTACAACTACAAGATGTTTTAGATAAGCTCCATGGTAACCACAAAACA
AAAAGCCACAGCAGATACACAAATGAGAAAGAGAAAGAAATAAAAACTTAACACTACAGAAAATCACCAAAT
CACTAAGACAGTAAGAGAGGAAGAAAGAAACAAAGGATCTACAAAACAACCAGAAAACAATTTAAAAAATGG
CAGGAGTAAGTCCTTATTTATCAGTAATAACTTTGAATGTAAATAGATTAAACATTAAGCTG**GGCCGGGCGC**
GGTGGCTCACGCCTGTAATCCCAGCACTTTGGGAGGCCGAGGCGGGCGGATCACGAGGTCAGGAGATCGAGA
CCATCCTGGCTAACACGGTGAAACCCCGTCTCTACTAAAAAAAAATACAAAAAATTAGCCGGGCGTGGTAGC
GGGCGCCTGTAGTCCCAGCTACTCGGGAGGCTGAGGCAGGGGAATGGCGTGAACCCGGGAGGCGGAGCTTGC
AGTGAGCCGAGATCCCGCC...[NEOcassette][Atail]AAACATTAAGCTGGATGTGGCGGCGCAAAC
CCATAATTCCAACTACTCAGGAGACTGATGCGAGAAGATTCCTTGAGCACAGAAGTTTGAGACCAGCCTGGA
CAACACAGTAAGACCCCCATCTCAAGAAAAAATAAGACATTAAGTGGCTGACATAGAGCGGGTAAAAAAATA
AATAAATAAATAAATAAATAAATAAATAAATAAATAAAA

Alu at the 5'; LINE-1 flanking the insertion site.

CLONE 198

[LZ48]

Driver: ORF2
Plasmid: AlurescueA70Du $A_{17}CATTACA_{18}GA_{17}CACACA_{18}$ (T)
Chromosome: 5
5’ position: 167,190,715
Strand: minus
DR: TAAAACAAATTCAT
ENDOsite: TTTA/AT

Empty site:

AGTGGCTTGGAAATTCTGACTTGTATTGATTGAGACACCTTCCCACGAAGAAAGATGGGATTAGTAATAAGT
TGCTTGTCAGTTACTCATTGGGTTCTTTGCCAGGCAAGGATGAGAGGGCTAAAGTTGAAAAAATCAAAGAAA
GAAAAAGGAAAAAAAAAAATGCTCACTGTAAATTAATGCTTGTCAAGCTCAACAAGTTGGAGAGCCGGAGGAA
AGCTTAATTAAAGTAAACAGACAATTTAAAGTGAGGGAAGTTCAGCCTACCTAGAAGAAAAATTCTAGGTAT
↑**TAAAACAAATTCAT**↓GAAAAGTTAGAAGCGCGCACTGCCATTTATCATTTATTCAAGACATAAGTGGTAAT
GAAGTTAGCAAACTCTCCCCAGATTTGATGTTAGAATGACTTTGGTTCTTAGACAATGCTGGGCAATTGAAG
ACAATTGAGCTTGTGTCTGTGAACAGCATCTTTTTTTCTTTTCTTTGACTTTTCTATTCCCCGCCCCACCAG

Filled site:

AGTGGCTTGGAAATTCTGACTTGTATTGATTGAGACACCTTCCCACGAAGAAAGATGGGATTAGTAATAAGT
TGCTTGTCAGTTACTCATTGGGTTCTTTGCCAGGCAAGGATGAGAGGGCTAAAGTTGAAAAAATCAAAGAAA
GAAAAAGGAAAAAAAAAAATGCTCACTGTAAATTAATGCTTGTCAAGCTCAACAAGTTGGAGAGCCGGAGGAA
AGCTTAATTAAAGTAAACAGACAATTTAAAGTGAGGGAAGTTCAGCCTACCTAGAAGAAAAATTCTAGGTAT
TAAAACAAATTCAT**GGCCGGGCGCGGTGGCTCACGCCTGTAATCCCAGCACTTTGGGAGGCCGAGGCGGGCG**
GATCACGAGGTCAGGAGATCGAGACCATCCTGGCTAACACGGTGAAACCCCGTCTCTACTAAAAAAAAAATA
CAAAAAATTAGCCGGGCGTGGTAGCGGGCGCCTGTAGTCCCAGCTACTCGGGAGGCTGAGGCAGGGGAATGG
CGTGAACCCGGGAGGCGGAGCTTGCAGTGAGCCGAGATCCCGCC... [NEOcassette] [Atail] TAAAA
CAAATTCATGAAAAGTTAGAAGCGCGCACTGCCATTTATCATTTATTCAAGACATAAGTGGTAATGAAGTTA
GCAAACTCTCCCCAGATTTGATGTTAGAATGACTTTGGTTCTTAGACAATGCTGGGCAATTGAAGACAATTG
AGCTTGTGTCTGTGAACAGCATCTTTTTTTCTTTTCTTTGACTTTTCTATTCCCCGCCCCACCAG

No repetitive sequences in the immediate proximity.

CLONE 199
[GG5]

Driver: ORF2
Plasmid: AlurescueA70Du $A_{17}CATTACA_{18}GA_{17}CACACA_{18}$ (T)
Chromosome: 6
5' position: 16,730,783
Strand: minus
DR: AAGAAATATATTATT
ENDOsite: TCTT/AA

Empty site:

TGACCTGGAATAAATCATGTTTAACTTCTTTATGCTTCAATTAGCTTTTGAAAGATATGTGTGGTTGTGTTT
AGCCAGTGGAAAGGCAATAACTGAAGTTCACAACGAAAGGAGAACTTGAAGGGAAATTTTGTTAGGAAAGAA
CAGTGGGCAAGAATACAATCTTCAGGCAGGGTGCTATTCCTAAAAAGTCTTGGTACACATGGTTCCTAAGGA
AGGTGGGACATTTTCTTATAATATTCTTTCTGTATTTTGTAGAATT↑**AAGAAATATATTATT**↓GTACATAAA
TATTTTATATTACATTATATATGTATAAATACATTTATATATATATATATATATACATACTGTTTTACCCTT
TAACTCCAGAAAGCTTTGCGTTCACACATCTTTTTCCCTAACTTCTACCTATAAAGGGATTTTTAGTGATAA
TCTTGAATTTAAATTGTAATCAAGCAGTAACAAACTTCATTTAATAT

Filled site:

TGACCTGGAATAAATCATGTTTAACTTCTTTATGCTTCAATTAGCTTTTGAAAGATATGTGTGGTTGTGTTT
AGCCAGTGGAAAGGCAATAACTGAAGTTCACAACGAAAGGAGAACTTGAAGGGAAATTTTGTTAGGAAAGAA
CAGTGGGCAAGAATACAATCTTCAGGCAGGGTGCTATTCCTAAAAAGTCTTGGTACACATGGTTCCTAAGGA
AGGTGGGACATTTTCTTATAATATTCTTTCTGTATTTTGTAGAATTAAGAAATATATTATT**GGCCGGGCGCG
GTGGCTCACGCCTGTAATCCCAGCACTTTGGGAGGCCGAGGCGGGCGGATCACGAGGTCAGGAGATCGAGAC
CATCCTGGCTAACACGGTGAAACCCCGTCTCTACTAAAAAAAAATACAAAAAATTAGCCGGGCGTGGTAGCG
GGCGCCTGTAGTCCCAGCTACTCGGGAGGCTGAGGCAGGGGAATGGCGTGAACCCGGGAGGCGGAGCTTGCA
GTGAGCCGAGATCCCGCC...[NEOcassette][Atail]**AAGAAATATATTATTGTACATAAATATTTTA
TATTACATTATATATGTATAAATACATTTATATATATATATATATATACATACTGTTTTACCCTTTAACTCC
AGAAAGCTTTGCGTTCACACATCTTTTTCCCTAACTTCTACCTATAAAGGGATTTTTAGTGATAATCTTGAA
TTTAAATTGTAATCAAGCAGTAACAAACTTCATTTAATAT

No repetitive sequences in the immediate proximity.

CLONE 200

[B2-7]

Driver: ORF2
Plasmid: AlurescueA70Du $A_{17}CATTACA_{18}GA_{17}CACACA_{18}$ (T)
Chromosome: 6
5' position: 36,825,728
Strand: plus
DR: N/A
ENDOsite: N/A

Empty site:

GTAGCACGTGCCTGTAGTCCCAGCTCCTTAGGAGGCTGAGGCAGGAGGACTGCTTTGAGCCCAGGAGTTTGA
GTTTACGGTGAGCTATGATCATGTCAGTATACTCCAGTCTAGGCAAGAGGCAAAACCTTGACTCTAAATAAA
TGAATACAGGGAAAATGAAGAAACATGGGATTGGGAATTATTTGCTAGCAGCATCACCACCAAATGTTATTT
ATATTATGCCCACAAGAGCAGGGTGCTGGGCTGGATACCCTAAGCTCAGGCAGGTTTTGCTCTAAGAGCAAA
AGGATGTAAGTTAGACCA↑↓GGCGCGGTGGCTCATGCCTATAATCCCAGCACGTTGGAAGGCCAAGGCAGGT
GGATCACGAGGTCATGAGTTCGAGACCAGCCTGGCCAACATAGTGAAACCCCCATCTCTACTAAAAATACAA
AAAAAAAATTAGCCAGGCATGGTGGCACACACCTGTAGTCCCAGCTACTTGGGAGGCTGAGGCAGGAGAATT
GCTTTGAACCCAGTAGGTGGAGGTTGCAGTGAGCCGAGATCATGCCATTGCACTCCAGCCTGGGCGACAGAG
CAAGACTGTCTCAAAAAAAAAAAAAAAAAAAAA

Filled site:

GTAGCACGTGCCTGTAGTCCCAGCTCCTTAGGAGGCTGAGGCAGGAGGACTGCTTTGAGCCCAGGAGTTTGA
GTTTACGGTGAGCTATGATCATGTCAGTATACTCCAGTCTAGGCAAGAGGCAAAACCTTGACTCTAAATAAA
TGAATACAGGGAAAATGAAGAAACATGGGATTGGGAATTATTTGCTAGCAGCATCACCACCAAATGTTATTT
ATATTATGCCCACAAGAGCAGGGTGCTGGGCTGGATACCCTAAGCTCAGGCAGGTTTTGCTCTAAGAGCAAA
AGGATGTAAGTTAGACCA**GGCGCGGTGGCTCACGCCTGTAATCCCAGCACTTTGGGAGGCCGAGGCGGGCGG
ATCACGAGGTCAGGAGATCGAGACCATCCTGGCTAACACGGTGAAACCCCGTCTCTACTAAAAAAAAAAAAA
ATACAAAAAATTAGCCGGGCGTGGTAGCGGGCGCCTGTAGTCCCAGCTACTCGGGAGGCTGAGGCAGGGGAA
TGGCGTGAACCCGGGAGGCGGAGCTTGCAGTGAGCCGAGATCCCGCCACTGCACTCCAGCCTG…[NEOcas
sette][Atail and 3' unsequenced]**

Alu flanking the insertion site.

Inserted by recombination with a pre-existing Alu.

CLONE 201

[GG51]

Driver: ORF2
Plasmid: AlurescueA70Du $A_{17}CATTACA_{18}GA_{17}CACACA_{18}$ (T)
Chromosome: 6
5’ position: 64,248,332
Strand: plus
DR: AAAAGATCAATGTTGG
ENDOsite: TTTT/AT

Empty site:

GCTGACATTAAATGTACATGCAAATTTTTAGCAAGGTTGTGCCTAATCACCACCATGAAAACTTCTTTGGAA
TATGAGCCTTGGTGTTTACCAGTAGGGAAATGACAGGTTGTTCAAAAGGAAATGTTTCCCAAGTTCTGGTTG
GAAGCTGGCATCAGAGAAAGAGGGAAATCTAGAACTGTTCTTAGTTTCTCTAGCAACAAGATTTCAAAAAAT
TGTACTGAAAGTTCAGTGAACATTGTTACAGGGATTTCCATTCTTCATAAAAAGAATAAACTTGCAAAAACT
GAGACTGAATGATTTAGCAATTGAATTAT↑**AAAAGATCAATGTTGG**↓ATATTCTGTGTTTTAAAACATAATA
AAAATTAGATTAGTTTAATTTGAATTCGAGACACTAAGTGATAAAGTTACATGTTCAGTATTTGAAGCTGTA
GCTTTGCATAAGCAATAAATAATTAATAGGATTTTGAAACTTGGTCCAAGCATATAAAATAGTGCAAATACC
AAGAAATAGCATGCAAAAAGAAAAAAAGAAG

Filled site:

GCTGACATTAAATGTACATGCAAATTTTTAGCAAGGTTGTGCCTAATCACCACCATGAAAACTTCTTTGGAA
TATGAGCCTTGGTGTTTACCAGTAGGGAAATGACAGGTTGTTCAAAAGGAAATGTTTCCCAAGTTCTGGTTG
GAAGCTGGCATCAGAGAAAGAGGGAAATCTAGAACTGTTCTTAGTTTCTCTAGCAACAAGATTTCAAAAAAT
TGTACTGAAAGTTCAGTGAACATTGTTACAGGGATTTCCATTCTTCATAAAAAGAATAAACTTGCAAAAACT
GAGACTGAATGATTTAGCAATTGAATTATAAAAGATCAATGTTGG**GGCCGGGCGCGGTGGCTCACGCCTGTA**
ATCCCAGCACTTTGGGAGGCCGAGGCGGGCGGATCACGAGGTCAGGAGATCGAGACCATCCTGGCTAACACG
GTGAAACCCCGTCTCTACTAAAAAAAAATACAAAAAATTAGCCGGGCGTGGTAGCGGGCGCCTGTAGTCCCA
GCTACTCGGGAGGCTGAGGCAGGGGAATGGCGTGAACCCGGGAGGCGGAGCTTGCAGTGAGCCGAGATCCCG
CC...[NEOcassette][Atail]AAAAGATCAATGTTGGATATTCTGTGTTTTAAAACATAATAAAAATT
AGATTAGTTTAATTTGAATTCGAGACACTAAGTGATAAAGTTACATGTTCAGTATTTGAAGCTGTAGCTTTG
CATAAGCAATAAATAATTAATAGGATTTTGAAACTTGGTCCAAGCATATAAAATAGTGCAAATACCAAGAAA
TAGCATGCAAAAAGAAAAAAAGAAG

No repetitive sequences in the immediate proximity.

CLONE 202

[TZ11]

Driver: ORF2
Plasmid: AlurescueA70Du $A_{17}CATTACA_{18}GA_{17}CACACA_{18}$ (T)
Chromosome: 6
5’ position: 86,242,220
Strand: plus
DR: N/A
ENDOsite: N/A

Empty site:

AAGCATAAAAATTAAAAATAGTTGTCNTAAAGCAAAAACAAAAGTATATTCCAAACAAAACTAAGTTTGATA
TGGAAATCTGACTCTATTATTGACAATGGTTTAGCTGTCCACCTGTCAGAATGCTGAAAGAACAGCAACTAC
TAAGACTGAACAGCAACTCCAGCAAAATCAGGCAGTATGGTTTAAGACCTGGACAGAAGATGCTGCCTGATA
TTTAATTATGACAGGAAAAATTATAACTAGCTACTCTGGGCATTCTTCCCTCCCAATTCCTATGTCTCATGT
TCTTGAAACAGGATCAACTTCTGAGGAAAATCAGAAAACCTAATACTTTTTGAAAAATAAGTAAGT↑↓AATA
ATTAGCACTGATCTTCACACTACTACTTAGTTATCCTCTCAAAACCACTTTCTAACATTGCCACTTTTTTTC
CTTCTTTCCTCATAACTCCCTCTTCCTGAGCTCAACTTTATTTTCTGCTCATCATTCTCTAAATGTGGTGCT
TTTCGTTACACTCACTGCCCCACTTCTTGGAAAAAGTGCAAAGTGGTGACTA

Filled site:

AAGCATAAAAATTAAAAATAGTTGTCNTAAAGCAAAAACAAAAGTATATTCCAAACAAAACTAAGTTTGATA
TGGAAATCTGACTCTATTATTGACAATGGTTTAGCTGTCCACCTGTCAGAATGCTGAAAGAACAGCAACTAC
TAAGACTGAACAGCAACTCCAGCAAAATCAGGCAGTATGGTTTAAGACCTGGACAGAAGATGCTGCCTGATA
TTTAATTATGACAGGAAAAATTATAACTAGCTACTCTGGGCATTCTTCCCTCCCAATTCCTATGTCTCATGT
TCTTGAAACAGGATCAACTTCTGAGGAAAATCAGAAAACCTAATACTTTTTGAAAAATAAGTAAGT**GGCCGG**
GCGCGGTGGCTCACGCCTGTAATCCCAGCACTTTGGGAGGCCGAGGCGGGCGGATCACGAGGTCAGGAGATC
GAGACCATCCTGGCTAACACGGTGAAACCCCGTCTCTACTAAAAAAAAAATACAAAAAATTAGCCGGGCGTG
GTAGCGGGCGCCTGTGGTCCCAGCTACTCGGGAGGCTGAGGCAGGGGAATGGCGTGAACCCGGGAGGCGGAG
CTTGCAGTGAGCCGAGATCCCGCC…[NEOcassette][Atail and 3' unsequenced]

Inserted by recombination with a pre-existing truncated Alu: missing 6 bp of 5’ Alu sequence.

CLONE 203

[GG33]

Driver: ORF2
Plasmid: AlurescueA70Du $A_{17}CATTACA_{18}GA_{17}CACACA_{18}$ (T)
Chromosome: 6
5' position: 134,633,594
Strand: plus
DR: N/A
ENDOsite: N/A

Empty site:

ACTACCTGAATTTGAATTCTGGCTATACCTCTTCCTAGGTCTACGATTTTTAACAAATCAGTTAACCTCTGA
CCCTCAGTTTCCTTGGCTATAAAATGGGCATAATGAAGGATTGTGAGAGTTACCTGAGTAAAGTGCTTTGAA
CGGTGCTGCTCAATAATGTTATCCCTCTGAGAAGACATCGAAAGTTTCCCATAAGAAAAGGGCCTTTGTTAT
TCCAATTTGGATACTTTAAGCAGGCAAAATAGAACAGTAAAAGGAAAACAAATGTAAAGGGATGCTGTTTAT
GAAATAGTTGTGAAGAATAAGCACTTCAGAGGAGCTTAATTTAACTTCTCAGCTCTG↑CTA↓GAGTAAACAT
TGTAACCAGACTAAAAACCTTTCCTTTGTTCCTGCAGTTATTAGAGAAAGGAGGAGGCCAGAGAAGTCATAT
CATTTCACTTCTCTGAACTTCACCCTGGATTATTTAATGTTGACAGGCATTCGGGATGTATAGAGATTAAGG
ACACAGACTCTGGAGCCTGGTGCTGGGATCCAAACCCTGATCC

Filled site:

ACTACCTGAATTTGAATTCTGGCTATACCTCTTCCTAGGTCTACGATTTTTAACAAATCAGTTAACCTCTGA
CCCTCAGTTTCCTTGGCTATAAAATGGGCATAATGAAGGATTGTGAGAGTTACCTGAGTAAAGTGCTTTGAA
CGGTGCTGCTCAATAATGTTATCCCTCTGAGAAGACATCGAAAGTTTCCCATAAGAAAAGGGCCTTTGTTAT
TCCAATTTGGATACTTTAAGCAGGCAAAATAGAACAGTAAAAGGAAAACAAATGTAAAGGGATGCTGTTTAT
GAAATAGTTGTGAAGAATAAGCACTTCAGAGGAGCTTAATTTAACTTCTCAGCTCTG**CTAAAAAAAAAATACA**
AAAAATTAGCCGGGCGTGGTAGCGGGCGCCTGTAGTCCCAGCTACTCGGGAGGCTGAGGCAGGGGAATGGCG
TGAACCCGGGAGGCGGAGCTTGCAGTGAGCCGAGATCCCGCC…[NEOcassette]*AAAAAAAAAAAAA*
AAAAAAAACATTACAAAAAAAAAAAAAAAAAAAAAAAAAAAAAGAAAAAAAAAAAAAAAAACACAC
AAAAAAAAAAAAAAAAAAAAAAAAAAAAAAAAAAAAAAAAAAAACCTTTCCTTTGTTCCTGCAGTTA
TTAGAGAAAGGAGGAGGCCAGAGAAGTCATATCATTTCACTTCTCTGAACTTCACCCTGGATTATTTAATGT
TGACAGGCATTCGGGATGTATAGAGATTAAGGACACAGACTCTGGAGCCTGGTGCTGGGATCCAAACCCTGA

Alu insert truncation: missing between 116-119 bp of 5' Alu sequence.

Sequence is shared between the Alu rescue vector and the genomic pre-insertion site (microhomology).

Post-insertion site includes a 22 bp deletion of chr. 6 sequence shown in blue.

MIRs at the 5' and the 3'.

Clone 203 is an example of an Alu insertion that contains a 5' truncated Alu with a typical oligo dA-tail. The insertion caused a 22 bp genomic deletion where the 5' junction appears to have undergone repair through non-homologous end joining (NHEJ) as evidence by 3 bp microhomology to

the genomic pre-insertion sequence and lack of the characteristic direct repeat.

CLONE 204

[LZ25]

Driver: ORF2
Plasmid: AlurescueA70Du $A_{17}CATTACA_{18}GA_{17}CACACA_{18}$ (T)
Chromosome: 6
5’ position: 135,233,306
Strand: plus
DR: AAAAAAAAATTATT
ENDOsite: TTTT/AA

Empty site:

AAAACAATACTTTGTAGTTGAAAACTTAGGAGGGAGAGTAGGTACTAATAAATCAATCTAACAAGAAAAAAG
TCAACTTGGGAGTATGGAGTAATATTTTGTAGGAATTTTAAATCAGGAATTTACTGACTCAAGTGTTTTGGA
TTAAACTTATTAAAGCAATGAATTCTTCTCTTATAAAATGAAGCCTTCTAGTAGGATGATCAGACCATTTAA
TGAGGAGAGAACACCCTGGGGCCAGTACTTAATCATGTCATCCACAACCTCTAATTTTTGCTCCTAAAAACT
CTGAAGCCATCTTTTTCTCCTTTTT↑**AAAAAAAAATTATT**↓TCTGTGCTAGTTCTAGAATCATGGCAAATGT
AAAGTTTTGAATTTGATCCATGCTTGACAAACACAAAGTAGATGAACACGTTTTTTATCTTATTTATGTAGA
AGAGGAAAATTTTCCTCTACCCTCTTCAGTTGGGCCTTGTGAATTAAACTGGCAAAAGTCAAATTGACAGGC
GAAAAAGGCATACGAATTTTATTTT

Filled site:

AAAACAATACTTTGTAGTTGAAAACTTAGGAGGGAGAGTAGGTACTAATAAATCAATCTAACAAGAAAAAAG
TCAACTTGGGAGTATGGAGTAATATTTTGTAGGAATTTTAAATCAGGAATTTACTGACTCAAGTGTTTTGGA
TTAAACTTATTAAAGCAATGAATTCTTCTCTTATAAAATGAAGCCTTCTAGTAGGATGATCAGACCATTTAA
TGAGGAGAGAACACCCTGGGGCCAGTACTTAATCATGTCATCCACAACCTCTAATTTTTGCTCCTAAAAACT
CTGAAGCCATCTTTTTCTCCTTTTTAAAAAAAAATTATT**GGCCGGGCGCGGTGGCTCACGCCTGTAATCCCA**
GCACTTTGGGAGGCCGAGGCGGGCGGATCACGAGGTCAGGAGATCGAGACCATCCTGGCTAACACGGTGAAA
CCCCGTCTCTACTAAAAAAAAAAATACAAAAAATTAGCCGGGCGTGGTAGCGGGCGCCTGTAGTCCCAGCTAC
TCGGGAGGCTGAGGCAGGGGAATGGCGTGAACCCGGGAGGCGGAGCTTGCAGTGAGCCGAGATCCCGCC...[N
EOcassette][Atail]AAAAAAAAATTATTTCTGTGCTAGTTCTAGAATCATGGCAAATGTAAAGTTT
TGAATTTGATCCATGCTTGACAAACACAAAGTAGATGAACACGTTTTTTATCTTATTTATGTAGAAGAGGAA
AATTTTCCTCTACCCTCTTCAGTTGGGCCTTGTGAATTAAACTGGCAAAAGTCAAATTGACAGGCGAAAAAG
GCATACGAATTTTATTTT

LTR at the 3'.

CLONE 205

[LZ70]

Driver: ORF2
Plasmid: AlurescueA70Du $A_{17}CATTACA_{18}GA_{17}CACACA_{18}$ (T)
Chromosome: 6
5' position: 137,359,240
Strand: minus
DR: AGAAACTCAGAGTT
ENDOsite: TTCT/AC

Empty site:

GAGGCAGGAGGATCACTTGAGCCCAGGAGTTTGGGGCTGCAGTGAGCTATGATCGCACCACTGCAACTCCAG
CCCGGGCAACAGAGCAAGACTCTGTCTCAAAAAAAAAAAAAAAAAAAAAAAAAAAAGGAGAACAAAAGAACTA
TAGAAAGAAAGTATGCTAGTCAGAGACATTAAAATTAAATACTGCTTTATCTTTCAGAATTCCTTTTGATGT
↑**AGAAACTCAGAGTT**↓GACAGGACCTTAAAAGTCACCCCACCCAGCTGCCACTCTCTTGATTGTCCTCTGCA
GCATCACTTCCAAGTAATGACCTGTCTGCTGTGTGAGCACCTCTCATCACAGGATACTCACTACCCTGCACC
CAGGCAGGCAGGATGTCCTGTCACTGCAGATTTCAACTATTAAAAAGTTCTTTGATAAAATGAGCTGAGCCT

Filled site:

GAGGCAGGAGGATCACTTGAGCCCAGGAGTTTGGGGCTGCAGTGAGCTATGATCGCACCACTGCAACTCCAG
CCCGGGCAACAGAGCAAGACTCTGTCTCAAAAAAAAAAAAAAAAAAAAAAAAAAAAGGAGAACAAAAGAACTA
TAGAAAGAAAGTATGCTAGTCAGAGACATTAAAATTAAATACTGCTTTATCTTTCAGAATTCCTTTTGATGT
AGAAACTCAGAGTT**GGCCGGGCGCGGTGGCTCACGCCTGTAATCCCAGCACTTTGGGAGGCCGAGGCGGGCG
GATCACGAGGTCAGGAGATCGAGACCATCCTGGCTAACACGGTGAAACCCCGTCTCTACTAAAAAAAAATAC
AAAAAATTAGCCGGGCGTGGTAGCGGGCGCCTGTAGTCCCAGCTACTCGGGAGGCTGAGGCAGGGGAATGGC
GTGAACCCGGGAGGCGGAGCTTGCAGTGAGCCGAGATCCCGCCACTGCACTCCA…[NEOcassette][At
ail]**AGAAACTCAGAGTTGACAGGACCTTAAAAGTCACCCCACCCAGCTGCCACTCTCTTGATTGTCCTCTG
CAGCATCACTTCCAAGTAATGACCTGTCTGCTGTGTGAGCACCTCTCATCACAGGATACTCACTACCCTGCA
CCCAGGCAGGCAGGATGTCCTGTCACTGCAGATTTCAACTATTAAAAAGTTCTTTGATAAAATGAGCTGAGC
CT

Alu at the 5'; L3 at the 3'.

CLONE 206

[LZ38]

Driver: ORF2
Plasmid: AlurescueA70Du $A_{17}CATTACA_{18}GA_{17}CACACA_{18}$ (T)
Chromosome: 7
5' position: 1,287,709
Strand: plus
DR: AAAAAAAAAAAAGG
ENDOsite: TTTT/CT

Empty site:

CGCACCCCCCGGACCCTCAGGACCTGGAGCGCGTGGAGGGCGTGGACCACCCCGGATCCCCCACCACCGGGC
GTGTTTGCCAAGGGTGTCATTGGGCGCCGCGCAGAAGGGGCTTCCGAGGACCCGCCCCTCCCCCGCAGCCGT
CAGGGCGCTGACGGAGGCAGCTGCGCCTGTTTAACTGGGAGCCGGTTGCTTGGCAACCCGAGGCGGCTCATT
ACCGCGGCCACAGTGATTAACCAAAAAAGAAAG↑**AAAAAAAAAAAAGG**↓AAAAAAAAAAGAGAGAAAAATAC
AAGGAAGAAAAAAATAGGGGAAAAGCGGAATATGAAAGTAAAATGGATTATTTAATTAGCTAATTGATAAT
CAACTTCTTTTTAATTATTCACTTAATTAAAAAAGTGTAACTACAACACTGCCCTCATCAAGCTGCGGTTGC
ACCAGCAGGGTTTCAAAGCCGCCTCTCCCTCCC

Filled site:

CGCACCCCCCGGACCCTCAGGACCTGGAGCGCGTGGAGGGCGTGGACCACCCCGGATCCCCCACCACCGGGC
GTGTTTGCCAAGGGTGTCATTGGGCGCCGCGCAGAAGGGGCTTCCGAGGACCCGCCCCTCCCCCGCAGCCGT
CAGGGCGCTGACGGAGGCAGCTGCGCCTGTTTAACTGGGAGCCGGTTGCTTGGCAACCCGAGGCGGCTCATT
ACCGCGGCCACAGTGATTAACCAAAAAAGAAAGAAAAAAAAAAAAGG**GGCCGGGCGCGGTGGCTCACGCCTG**
TAATCCCAGCACTTTGGGAGGCCGAGGCGGGCGGATCACGAGGTCAGGAGATCGAGACCATCCTGGCTAACA
CGGTGAAACCCCGTCTCTACTAAAAAAAAAAAATACAAAAAATTAGCCGGGCGTGGTAGCGGGCGCCTGTAG
TCCCAGCTACTCGGGAGGCTGAGGCAGGGGAATGGCGTGAACCCGGGAGGCGGAGCTTGCAGTGAGCCGAGA
TCCCGCCACTGCAC…[NEOcassette][Atail]AAAAAAAAAAAAGGAAAAAAAAAAGAGAGAAAAAT
ACAAGGAAGAAAAAAATAGGGGAAAAGCGGAATATGAAAGTAAAATGGATTATTTAATTAGCTAATTGATA
ATCAACTTCTTTTTAATTATTCACTTAATTAAAAAAGTGTAACTACAACACTGCCCTCATCAAGCTGCGGTT
GCACCAGCAGGGTTTCAAAGCCGCCTCTCCCTCCC

No repetitive sequences in the immediate proximity.

CLONE 207

[TZ21]

Driver: ORF2
Plasmid: AlurescueA70Du $A_{17}CATTACA_{18}GA_{17}CACACA_{18}$ (T)
Chromosome: 7
5' position: 27,741,174
Strand: minus
DR: N/A
ENDOsite: N/A

Empty site:

GTCACTCCATGTGCCTTGCATCTTTCTTACGGCCAATGAGCTAAAACATGGAGGATGTGAATGTCACATGGG
GTGGAAGGAAGAGGAGAGGGCTAAAAGACACATGGCGGCAGGGCATGGTGGCTCACGCCTGTAATCCCAGCA
CTTTGGGAGGCTGAGGCGGGCAGATCACCTGAGGTCAGGAGTTCGAGACCAGCCTGACCAACATGGTGAAAC
CCTGTCTCTACTAAAAATACAAAAATTACCTGGGCCTGGTGGCGGGTGCCTATAATCCCAGCTACTTGGGAG
GCTGAGGCAGAGAATTGTTTGAACCCGGGAGGCAGAGGTTGCAGTGAGCTGAGATTGCACCACTACACTCCA
GCCTGGGTGACAGATTGAGACTCCGTCTTAAAAAAAAAAAAAATA↑↓AAAGACACATGCCTGGTATAGAATGT
TAGTCTGATGAATATGTAATGTGCCAGAGTCAGAAAAATTGTAAACTATCCCTAATCCGCAAAGCTATTCAA
GTTAGCAGGACAAAACTTCGAGGTTAATCAGATGAATGCTGATATTCAACATTCAGCTGATTTCCCTCAAAA
ATCTTCAATGGATCTTTTTCCTACTCTCTG

Filled site:

GTCACTCCATGTGCCTTGCATCTTTCTTACGGCCAATGAGCTAAAACATGGAGGATGTGAATGTCACATGGG
GTGGAAGGAAGAGGAGAGGGCTAAAAGACACATGGCGGCAGGGCATGGTGGCTCACGCCTGTAATCCCAGCA
CTTTGGGAGGCTGAGGCGGGCAGATCACCTGAGGTCAGGAGTTCGAGACCAGCCTGACCAACATGGTGAAAC
CCTGTCTCTACTAAAAATACAAAAATTACCTGGGCCTGGTGGCGGGTGCCTATAATCCCAGCTACTTGGGAG
GCTGAGGCAGAGAATTGTTTGAACCCGGGAGGCAGAGGTTGCAGTGAGCTGAGATTGCACCACTACACTCCA
GCCTGGGTGACAGATTGAGACTCCGTCTTAAAAAAAAAAAAAATA**GGCCGGGCGCGGTGGCTCACGCCTGTAA**
TCCCAGCACTTTGGGAGGCCGAGGCGGGCGGATCACGAGGTCAGGAGATCGAGACCATCCTGGCTAACACGG
TGAAACCCCGTCTCTACTAAAAAAAAAAAAATACAAAAAATTAGCCGGGCGTGGTAGCGGGCGCCTGTAGTCC
CAGCTACTCGGGAGGCTGAGGCAGGGGAATGGCGTGAACCCGGGAGGCGGAGCTTGCAGTGAGCCGAGATCC
CGCC…[NEOcassette][Atail and 3' unsequenced]

Alu flanking the insertion site.

CLONE 208

[TZ9]

Driver: ORF2
Plasmid: AlurescueA70Du $A_{17}CATTACA_{18}GA_{17}CACACA_{18}$ (T)
Chromosome: 7
5' position: 45,867,351
Strand: plus
DR: N/A
ENDOsite: N/A

Empty site:

CAACATAGTAAGACCCCCATCTCTACAAAAAACAAAGATTAGCCAGGTGTGGTGGTGCATGCCTGTAATCCC
AGCGCTTGGGAGGCTGAGGCAAGATGATAGCTTGAGCCCAGAAGGTTGAGGCTGCAGTGAGCCATGATCACG
CCGGTGCACTACAGCCTGGGTGACAGGTGTACCCTGTCTCTAAAAAAATAAAAAAAAGAGGCCAGGCATGGTG
GCTCACATCTGTAATTCCAGCACTTTGGGAGGCCAAGGCCGTTGGATCACCTCAGGTCAGGGGTTTGAGACC
AGCCTGGCTAACATGGTGAAACCCCATCTCTATTAAAAATACAAAAATT↑↓AGCCGGGCATAGTGGGCGGGT
GACTTAGCTGTGCTGGTAGAACGTATGGGGATTCCCACCCCATGCAGCACGCCATCCCATTTGCCGAGTGTG
GTGGCTCACGCCTGTAATCCCAACACTTTGGGAGGCTGAAGCGGGCAGACCACCTGAGGTTGAGAGTTTGAG
ATCAGCCTGGCTAACATGGTGAAACCTCTAGCTTT

Filled site:

CAACATAGTAAGACCCCCATCTCTACAAAAAACAAAGATTAGCCAGGTGTGGTGGTGCATGCCTGTAATCCC
AGCGCTTGGGAGGCTGAGGCAAGATGATAGCTTGAGCCCAGAAGGTTGAGGCTGCAGTGAGCCATGATCACG
CCGGTGCACTACAGCCTGGGTGACAGGTGTACCCTGTCTCTAAAAAAATAAAAAAAAGAGGCCAGGCATGGTG
GCTCACATCTGTAATTCCAGCACTTTGGGAGGCCAAGGCCGTTGGATCACCTCAGGTCAGGGGTTTGAGACC
AGCCTGGCTAACATGGTGAAACCCCATCTCTATTAAAAATACAAAAATT**GGCCGGGCGCGGTGGCTCACGCC**
TGTAATCCCAGCACTTTGGGAGGCCGAGGCGGGCGGATCACGAGGTCAGGAGATCGAGACCATCCTGGCTAA
CACGGTGAAACCCCGTCTCTACTAAAAAAAAATACAAAAAATTAGCCGGGCGTGGTAGCGGGCGCCTGTAGT
CCCAGCTACTCGGGAGGCTGAGGCAGGGGAATGGCGTGAACCCGGGAGGCGGAGCTTGCAGTGAGCCGAGAT
CCCGCCA…[NEOcassette][Atail and 3' unsequenced]

Alus flanking the insertion site.

CLONE 209

[LZ19]

Driver: ORF2
Plasmid: AlurescueA70Du $A_{17}CATTACA_{18}GA_{17}CACACA_{18}$ (T)
Chromosome: 7
5’ position: 65,619,169
Strand: minus
DR: CAAAAAAAAAAAAAAAAAAA
ENDOsite: TTTG/AA

Empty site:

CTCTTCAGATTTATAAAATGACAAAACTTGATAATTACCTGGAAACAATTTATTATTAACTAGGTTAACCCT
AAATCCATCAAGAAAAACCACCTAACATATTCAGTGTTAAAAAGAGACATTACGACTAGCCTGGCCAATATG
GCAAAACCCTGTCTTTACTAAAAATACAAATTTTAGCAGGGTATAGTGGTACGTGCCTGTAAGTCCCAGCTA
CTCAGGAGGCTAAGGCACCAGAATCGCTTGAATCTGGGAGGCAGAGGTTGCAGTGAGCAGAGATCGTGCCAC
TGCACTCCTGGGCGACACAGCGAGACTGTTT↑**CAAAAAAAAAAAAAAAAAAA**↓GAGAGAGACAACAATGAGA
CAGATTGGCACATGTATGCCCTGAGAAATAAATCAACAGCTCCCACTAAAATTCTCAATACGCTAGACAGAT
TTAAAAGTACAATCTGGTACTTTCTCTTTTTTGAGATGGAGTCTCACCCTGTCGGCCAGGCTGGAGTGCAAT
GCACGATCTGGCTCACTGCAACCTCTGCCTCCCCGGT

Filled site:

CTCTTCAGATTTATAAAATGACAAAACTTGATAATTACCTGGAAACAATTTATTATTAACTAGGTTAACCCT
AAATCCATCAAGAAAAACCACCTAACATATTCAGTGTTAAAAAGAGACATTACGACTAGCCTGGCCAATATG
GCAAAACCCTGTCTTTACTAAAAATACAAATTTTAGCAGGGTATAGTGGTACGTGCCTGTAAGTCCCAGCTA
CTCAGGAGGCTAAGGCACCAGAATCGCTTGAATCTGGGAGGCAGAGGTTGCAGTGAGCAGAGATCGTGCCAC
TGCACTCCTGGGCGACACAGCGAGACTGTTTCAAAAAAAAAAAAAAAAAAA**GGCCGGGCGCGGTGGCTCACG**
CCTGTAATCCCAGCACTTTGGGAGGCCGAGGCGGGCGGATCACGAGGTCAGGAGATCGAGACCATCCTGGCT
AACACGGTGAAACCCCGTCTCTACTAAAAAAAAATACAAAAAATTAGCCGGGCGTGGTAGCGGGCGCCTGTA
GTCCCAGCTACTCGGGAGGCTGAGGCAGGGGAATGGCGTGAACCCGGGAGGCGGAGCTTGCAGTGAGCCGAG
ATCCCGCCA…[NEOcassette][Atail]CAAAAAAAAAAAAAAAAAAAGAGAGAGACAACAATGAGAC
AGATTGGCACATGTATGCCCTGAGAAATAAATCAACAGCTCCCACTAAAATTCTCAATACGCTAGACAGATT
TAAAAGTACAATCTGGTACTTTCTCTTTTTTGAGATGGAGTCTCACCCTGTCGGCCAGGCTGGAGTGCAATG
CACGATCTGGCTCACTGCAACCTCTGCCTCCCCGGT

Alu flanking the insertion site.

CLONE 210

[LZ5]

Driver: ORF2
Plasmid: AlurescueA70Du   A$_{17}$CATTACA$_{18}$GA$_{17}$CACACA$_{18}$ (T)
Chromosome: 7
5’ position: 72,232,576
Strand: plus
DR: AAGCTGAGATC
ENDOsite: GCTT/AC

Empty site:

ATCTGCCCAGCAACTGCCAGCCTACCCTCGGACTGCCGTCACCCCTGTTATTGATCTTTGCAGCCAAGGATA
ATTATTTCAAAACAATTACATAATCCTCCTCAATTTTTCCTTTAAAAATCTTTGTCTTTGGCCAGGCACGGT
GGCTCACATCTGTAATCCCAGCACTTTGGGAGACCAAGGTAGGTGGATCACGAGGTCAGGAGATCGAGACCA
TCCTGGCTAACATGGTGAAACCCATCTCTACTAAAAATACAAAAAAAAAAAAATTAGCCGGGTGTGGTGGCGG
GCGCCTGTAGTCCCAGCTACTCAGGAGGCTGAGGCAGGAGAATGGTGTGAACCTGGGAGGCGGAGCCTGCAG
T↑**AAGCTGAGATC**↓ACACCACTGCACTCCAGCCTGGGTGACAGGGCAAGACCCCGTCTCAAAAAAATAATAA
AAAAATAAAAAAATAAAAAAAACTTTGTCTTCATTGACTTATAGTAACTCATAAAAAATTAAAAAGTAAACT
TTGTCTTCCTGTACCTCCCCAAAAACACAGTTTACTGTGGCTTGCATATTCACATTGCAGTGCTCTATTC

Filled site:

ATCTGCCCAGCAACTGCCAGCCTACCCTCGGACTGCCGTCACCCCTGTTATTGATCTTTGCAGCCAAGGATA
ATTATTTCAAAACAATTACATAATCCTCCTCAATTTTTCCTTTAAAAATCTTTGTCTTTGGCCAGGCACGGT
GGCTCACATCTGTAATCCCAGCACTTTGGGAGACCAAGGTAGGTGGATCACGAGGTCAGGAGATCGAGACCA
TCCTGGCTAACATGGTGAAACCCATCTCTACTAAAAATACAAAAAAAAAAAAATTAGCCGGGTGTGGTGGCGG
GCGCCTGTAGTCCCAGCTACTCAGGAGGCTGAGGCAGGAGAATGGTGTGAACCTGGGAGGCGGAGCCTGCAG
TAAGCTGAGATC**GGCCGGGCGCGGTGGCTCACGCCTGTAATCCCAGCACTTTGGGAGGCCGAGGCGGGCGGA**
TCACGAGGTCAGGAGATCGAGACCATCCTGGCTAACACGGTGAAACCCCGTCTCTACTAAAAAAAAAAAATA
CAAAAAATTAGCCGGGCGTGGTAGCGGGCGCCTGTAGTCCCAGCTACTCGGGAGGCTGAGGCAGGGGAATGG
CGTGAACCCGGGAGGCGGAGCTTGCAGTGAGCCGAGATCCCGCCACTGC... [NEOcassette] [Atail]
AAGCTGAGATCACACCACTGCACTCCAGCCTGGGTGACAGGGCAAGACCCCGTCTCAAAAAAATAATAAAAA
AATAAAAAAATAAAAAAAACTTTGTCTTCATTGACTTATAGTAACTCATAAAAAATTAAAAAGTAAACTTTG
TCTTCCTGTACCTCCCCAAAAACACAGTTTACTGTGGCTTGCATATTCACATTGCAGTGCTCTATTC

Alu flanking the insertion site; LTRs 5' and 3' of the insertion site.

CLONE 211

[LZ22]

Driver: ORF2
Plasmid: AlurescueA70Du $A_{17}CATTACA_{18}GA_{17}CACACA_{18}$ (T)
Chromosome: 7
5’ position: 74,666,432
Strand: minus
DR: AAAAATTAAAGAGATCT
ENDOsite: TTTT/AC

Empty site:

GGGAGTTCAAGACCAGCCTGACCAACATGGAGAAAACCCCGTCTCTACTAAAAATACAAAATTGGCCAGGGT
GGTGGCGCATGCCTGTAATCCCAGCTGCTCGGGAGGCTGAGGCAGGAGAATCACTTGAACCCGGGAGACGGA
AGTTGCGGTGAGCCAAGATCGCGCCATTGCACTCCAGCCTGGGCAACAAGAGTGAAACTCCGTCTCAAAAAA
AAAAAAGAAAGAAAGAAAAAAAAAAAGAAAAGAAAAAGGAAATTGGT↑**AAAAATTAAAGAGATCT**↓AACTAAA
GTATGGACTTAAGTTAATTACAATGTATTAATTTCAGTTCATTAACGGTAACAAATATACTATACCAGTGCA
AGATGTTAATAAAAGGGGGAAATGGTACAGGATATATGCGAATCCTACTGTCTTAACAATTTTTCTGTAAAT
TTAAAACCACTGTAAGATGAAGAGTCTATTTTTAGAGTACTGAGTAAAT

Filled site:

GGGAGTTCAAGACCAGCCTGACCAACATGGAGAAAACCCCGTCTCTACTAAAAATACAAAATTGGCCAGGGT
GGTGGCGCATGCCTGTAATCCCAGCTGCTCGGGAGGCTGAGGCAGGAGAATCACTTGAACCCGGGAGACGGA
AGTTGCGGTGAGCCAAGATCGCGCCATTGCACTCCAGCCTGGGCAACAAGAGTGAAACTCCGTCTCAAAAAA
AAAAAAGAAAGAAAGAAAAAAAAAAAGAAAAGAAAAAGGAAATTGGTAAAAATTAAAGAGATCT**GGCCGGGCG
CGGTGGCTCACGCCTGTAATCCCAGCACTTTGGGAGGCCGAGGCGGGCGGATCACGAGGTCAGGAGATCGAG
ACCATCCTGGCTAACACGGTGAAACCCCGTCTCTACTAAAAAAAAAAAATACAAAAAATTAGCCGGGCGTGGT
AGCGGGCGCCTGTAGTCCCAGCTACTCGGGAGGCTGAGGCAGGGGAATGGCGTGAACCCGGGAGGCGGAGCT
TGCAGTGAGCCGAGATCCCGCC... [NEOcassette] [Atail]**AAAAATTAAAGAGATCTAACTAAAGTA
TGGACTTAAGTTAATTACAATGTATTAATTTCAGTTCATTAACGGTAACAAATATACTATACCAGTGCAAGA
TGTTAATAAAAGGGGGAAATGGTACAGGATATATGCGAATCCTACTGTCTTAACAATTTTTCTGTAAATTTA
AAACCACTGTAAGATGAAGAGTCTATTTTTAGAGTACTGAGTAAAT

Alu at the 5'; LINE-1 flanking the insertion site.

CLONE 212

[GG14]

Driver: ORF2
Plasmid: AlurescueA70Du $A_{17}CATTACA_{18}GA_{17}CACACA_{18}$ (T)
Chromosome: 7
5' position: 117,267,531
Strand: minus
DR: AAGAATGGCAGACA
ENDOsite: TCTT/AA

Empty site:

GTTCTCTAATATGGCATTTCCACCTTCTGTGTATTTTGCTGTGAGATCTTTGACAGTCATTTGGCCCCCTGA
GGGCCAGATGTCATCTTTCTTCACGTGTGAATTCTCAATAATCATAACTTTCGAGAGTTGGCCATTCTTGTA
TGGTTTGGTTGACTTGGTAGGTTTACCTTCTGTTGGCATGTCAATGAACTTAAAGACTCGGCTCACAGATCG
CATCTGAAATAAAAATAACAACATTTTTGTTTTT↑**AAGAATGGCAGACA**↓ATTTCACAATTAGTTTGATGCT
TCCTAAAAAATAACTTGTAAAATGCAACACTTGCTATTTGTCACTTGGTTATTTGTCGGGCTTTTCAATGAA
CTAACAGGAAATAATTGGATATAATTAAAAGTTTAACTGAAGAGAAATAACCAGGACTTATATAAATGGGCT
CTGTATAGGCCTGTTTATTTTTGTGATCACCTAA

Filled site:

GTTCTCTAATATGGCATTTCCACCTTCTGTGTATTTTGCTGTGAGATCTTTGACAGTCATTTGGCCCCCTGA
GGGCCAGATGTCATCTTTCTTCACGTGTGAATTCTCAATAATCATAACTTTCGAGAGTTGGCCATTCTTGTA
TGGTTTGGTTGACTTGGTAGGTTTACCTTCTGTTGGCATGTCAATGAACTTAAAGACTCGGCTCACAGATCG
CATCTGAAATAAAAATAACAACATTTTTGTTTTTAAGAATGGCAGACA**GGCCGGGCGCGGTGGCTCACGCCT
GTAATCCCAGCACTTTGGGAGGCCGAGGCGGGCGGATCACGAGGTCAGGAGATCGAGACCATCCTGGCTAAC
ACGGTGAAACCCCGTCTCTACTAAAAAAAAATACAAAAAATTAGCCGGGCGTGGTAGCGGGCGCCTGTAGTC
CCAGCTACTCGGGAGGCTGAGGCAGGGGAATGGCGTGAACCCGGGAGGCGGAGCTTGC…[NEOcassette
][Atail]**AAGAATGGCAGACAATTTCACAATTAGTTTGATGCTTCCTAAAAAATAACTTGTAAAATGCAA
CACTTGCTATTTGTCACTTGGTTATTTGTCGGGCTTTTCAATGAACTAACAGGAAATAATTGGATATAATTA
AAAGTTTAACTGAAGAGAAATAACCAGGACTTATATAAATGGGCTCTGTATAGGCCTGTTTATTTTTGTGAT
CACCTAA

No repetitive sequences in the immediate proximity.

CLONE 213

[LZ63]

Driver: ORF2
Plasmid: AlurescueA70Du $A_{17}CATTACA_{18}GA_{17}CACACA_{18}$ (T)
Chromosome: 7
5’ position: 156,719,757
Strand: minus
DR: AAAAAAAGCTCTTA
ENDOsite: TTTT/GT

Empty site:

GTTAGCCTACATTAAATAATGACGGCCAATGATTGGAAGAAAAACATCCTTATGTTCCCCTGCAGAGTGTGA
GATTGATGAGAATGGATTACCCTGCCAAGGGAACTCAAAAATATTACAACCTTTTATTCCTCTGTGGCTTGG
ACGAGTGTGTATTCATTTCTACGCTATTAC↑**AAAAAAAGCTCTTA**↓CAACGATTCTGAGCTAAAGACACATT
AAATCACCACTTTAATTATATCGAAACAGGACTCTGAGATTCTATCACTAATCTAGTTTAATCTTGGCAACT
CAAGCTCATCTAATATTTTATTTCTATTCAGGTCATATTAGTCAATTGGGCATTAGCAGCAGTACTTATTTA
TCTTATCAGTTTATTTTTGGTAAAGTAGTA

Filled site:

GTTAGCCTACATTAAATAATGACGGCCAATGATTGGAAGAAAAACATCCTTATGTTCCCCTGCAGAGTGTGA
GATTGATGAGAATGGATTACCCTGCCAAGGGAACTCAAAAATATTACAACCTTTTATTCCTCTGTGGCTTGG
ACGAGTGTGTATTCATTTCTACGCTATTACAAAAAAAGCTCTTA**GGCCGGGCGCGGTGGCTCACGCCTGTAA**
TCCCAGCACTTTGGGAGGCCGAGGCGGGCGGATCACGAGGTCAGGAGATCGAGACCATCCTGGCTAACACGG
TGAAACCCCGTCTCTACTAAAAAAAAATACAAAAAATTAGCCGGGCGTGGTAGCGGGCGCCTGTAGTCCCAG
CTACTCGGGAGGCTGAGGCAGGGGAATGGCGTGAACCCGGGAGGCGGAGCTTGCAGTGAGCCGAGATCCCGC
C…[NEOcassette][Atail]AAAAAAAGCTCTTACAACGATTCTGAGCTAAAGACACATTAAATCACC
ACTTTAATTATATCGAAACAGGACTCTGAGATTCTATCACTAATCTAGTTTAATCTTGGCAACTCAAGCTCA
TCTAATATTTTATTTCTATTCAGGTCATATTAGTCAATTGGGCATTAGCAGCAGTACTTATTTATCTTATCA
GTTTATTTTTGGTAAAGTAGTA

No repetitive sequences in the immediate proximity.

CLONE 214

[LZ47]

Driver: ORF2
Plasmid: AlurescueA70Du $A_{17}CATTACA_{18}GA_{17}CACACA_{18}$ (T)
Chromosome: 8
5' position: 97,323,906
Strand: plus
DR: GAAGACTAAGGGCCATTGTCATTTGTTTC
ENDOsite: CTTC/TA

Empty site:

ATCCAGCAAATCTATACATGAAGTTCAATCCCCAAGGTGGCTTAGCTTCAAGACCTAATCGAGGTTCTAAAT
GGAGTATGAGAGTATAAAGAAAAATTGAGGAAAACCCATTATAGAGAAGAAATTACTTTTCACAGATTTTTA
AACAGGACATTCTTCCAATTAAAAAGTAATACATGCACAATGATAAAAATTCAGAAAAGGATCTAGAAGGAA
CTAAAGATCACCCAAAGAAGTCAACCATTTAGAGATAATCATTGTTAATATTTTAATGTCATTCCTTTCCAT
TTTTCTGTGTATGTGTGTGTGTGTTTCCAGCTGTCATCTAGATTCTGAAGGGTGACATAGTTTTAAAGGCTA
AAAATGTTGATACTCCGGTTAGATTTAGGCAGGTTCCTCCCGACCCTTA↑**GAAGACTAAGGGCCATTGTCAT**
TTGTTTC↓TCTTAGGTTCTATGGTGTAAGCTGATTTTCTTACCCAAAGTGGTTTGTTGTAAAAGGTGATAAG
ATGGATTTGGTTTCCTTTGTTCATGATATAGCAGGAG

Filled site:

ATCCAGCAAATCTATACATGAAGTTCAATCCCCAAGGTGGCTTAGCTTCAAGACCTAATCGAGGTTCTAAAT
GGAGTATGAGAGTATAAAGAAAAATTGAGGAAAACCCATTATAGAGAAGAAATTACTTTTCACAGATTTTTA
AACAGGACATTCTTCCAATTAAAAAGTAATACATGCACAATGATAAAAATTCAGAAAAGGATCTAGAAGGAA
CTAAAGATCACCCAAAGAAGTCAACCATTTAGAGATAATCATTGTTAATATTTTAATGTCATTCCTTTCCAT
TTTTCTGTGTATGTGTGTGTGTGTTTCCAGCTGTCATCTAGATTCTGAAGGGTGACATAGTTTTAAAGGCTA
AAAATGTTGATACTCCGGTTAGATTTAGGCAGGTTCCTCCCGACCCTTAGAAGACTAAGGGCCATTGTCATT
TGTTTCCA**CGACAGAGCGAGTCGACGTCTC…[NEOcassette]*AAAAAAAAAAAAAAAAAAAAAAACA***
TTACAAAAAAAAAAAAAAAAAAAAAGAAAAAAAAAAAAAAAAAAAAAAAAAAAAAAAAAAAAGAAGA
CTAAGGGCCATTGTCATTTGTTTCTCTTAGGTTCTATGGTGTAAGCTGATTTTCTTACCCAAAGTGGTTTGT
TGTAAAAGGTGATAAGATGGATTTGGTTTCCTTTGTTCATGATATAGCAGGAGGGAGAATTCACACATCTCC
CAGATTTCTGACAGCGCAGAGGTGGCGCTCCCGCTTCATGTGTGAGGGCCCTGTGTACTGGAAGAAGGGATG
TGGGCCCCT

LINE-1 at the 5'.

Alu insert truncation: missing between 264 bp of 5' Alu sequence.

These 2 bases untemplated bases (absent in both the genomic sequence and the Alu rescue vector).

CLONE 215

[TZ23]

Driver: ORF2
Plasmid: AlurescueA70Du $A_{17}CATTACA_{18}GA_{17}CACACA_{18}$ (T)
Chromosome: 8
5’ position: 102,554,170
Strand: minus
DR: N/A
ENDOsite: N/A

Empty site:

ATGCTAATGATTATTGAAAACTGTTTTATCTAGTTTAATCTATACTTGAAATTACTAGCAAATTATAGACCA
ACCCATGTCTCAATGTAAAAGGCAATGTAAGCTACTTTGCAAACACAGTGTATTCAGGTAATGCTGTGTAAA
ATGATATGGAACAGCAGTAATGAGACGGACTCTATTGTAGATCCTGTGCTACAACAGAATTCTACAATATTT
AGGTGATATAAAAACTACAAGCTAC↑↓AAAGACCATCATGGAAAGTGAGGGAGTCTATACGGGGAATTTACA
GTAGAAACAGAAATGGGTGGGAGTCAATCAATCCTAACCAAGCCAGCCTTGCATAAAGCAGCAATTGACAAA
CAAGGCAATGTCTTCAGGTGAGTATGATGACTCAAGTACCCAAAGACCATGACAGTCCTCAGCATTTCCCCT
GCTGGGTGCTC

Filled site:

ATGCTAATGATTATTGAAAACTGTTTTATCTAGTTTAATCTATACTTGAAATTACTAGCAAATTATAGACCA
ACCCATGTCTCAATGTAAAAGGCAATGTAAGCTACTTTGCAAACACAGTGTATTCAGGTAATGCTGTGTAAA
ATGATATGGAACAGCAGTAATGAGACGGACTCTATTGTAGATCCTGTGCTACAACAGAATTCTACAATATTT
AGGTGATATAAAAACTACAAGCTAC**GGCCGGGCGCGGTGGCTCACGCCTGTAATCCCAGCACTTTGGGAGGC
CGAGGCGGGCGGATCACGAGGTCAGGAGATCGAGACCATCCTGGCTAACACGGTGAAACCCCGTCTCTACTA
AAAAAAAATACAAAAAATTAGCCGGGCGTGGTAGCGGGCGCCTGTAGTCCCAGCTACTCGGGAGGCTGAGGC
AGGGGAATGGCGTGAACCCGGGAGGCGGAGCTTGCAGTGAGCCGAGAT…[NEOcassette][Atail
and 3' unsequenced]**

No repetitive sequences in the immediate proximity.

CLONE 216

[LZ6]

Driver: ORF2
Plasmid: AlurescueA70Du $A_{17}CATTACA_{18}GA_{17}CACACA_{18}$ (T)
Chromosome: 9
5’ position: 22,157,141
Strand: plus
DR: AAAGAATTATTTGTA
ENDOsite: CTTT/AA

Empty site:

AATACTCTAAATTCTTAGAACCCAGCCCAGTACTTGCTAGTCATCATTTAAATACATTGAATGATAGAGAAA
ATTCTTTTGATCACAAACCTTATACCAGCGTTGTCAAAATTATAATGATAAAGTAGCTCATTTTAAGCGCGT
CCTGGGTTGGGAAACTTAACAATTTTAAAACTTGGAATAAATTCAATAAAATGACATAGTATGGTTACAATG
TCTGTCTACAGAGTCAGGGCAAGAACAAAGAAAATTGAAAGAAGATTGTTTTAGTGTAGTAAAATTACTGGA
TAATTTTCATTTCCTTTGCTCTTT↑**AAAGAATTATTTGTA**↓AAGGGTATTTGAAATGAAAACAAGATTGGAG
AAAAACAACTTGGAAGAAAATCATAGGCAAATAACAATAATGATTGAAGAGTTGTGAATAAGGAGAGTTAGG
AATCAAGTGGTTCCAGGCTTGCAGAATCTGAAAGGAGCCTAGAGTGAGGAGGTTGATCTGTGAAGGATGATG
ATGATCTCCATTAAGGACCAGACAG

Filled site:

AATACTCTAAATTCTTAGAACCCAGCCCAGTACTTGCTAGTCATCATTTAAATACATTGAATGATAGAGAAA
ATTCTTTTGATCACAAACCTTATACCAGCGTTGTCAAAATTATAATGATAAAGTAGCTCATTTTAAGCGCGT
CCTGGGTTGGGAAACTTAACAATTTTAAAACTTGGAATAAATTCAATAAAATGACATAGTATGGTTACAATG
TCTGTCTACAGAGTCAGGGCAAGAACAAAGAAAATTGAAAGAAGATTGTTTTAGTGTAGTAAAATTACTGGA
TAATTTTCATTTCCTTTGCTCTTTAAAGAATTATTTGTA**GGCCGGGCGCGGTGGCTCACGCCTGTAATCCCA**
GCACTTTGGGAGGCCGAGGCGGGCGGATCACGAGGTCAGGAGATCGAGACCATCCTGGCTAACACGGTGAAA
CCCCGTCTCTACTAAAAAAAAAATACAAAAAATTAGCCGGGCGTGGTAGCGGGCGCCTGTAGTCCCAGCTAC
TCGGGAGGCTGAGGCAGGGGAATGGCGTGAACCCGGGAGGCGGAGCTTGCAGTGAGCCGAGATCCCGCCACT
GCACT...[NEOcassette][Atail]AAAGAATTATTTGTAAAGGGTATTTGAAATGAAAACAAGATTGG
AGAAAAACAACTTGGAAGAAAATCATAGGCAAATAACAATAATGATTGAAGAGTTGTGAATAAGGAGAGTTA
GGAATCAAGTGGTTCCAGGCTTGCAGAATCTGAAAGGAGCCTAGAGTGAGGAGGTTGATCTGTGAAGGATGA
TGATGATCTCCATTAAGGACCAGACAG

No repetitive sequences in the immediate proximity.

CLONE 217

[GG32,GG44]

Driver: ORF2
Plasmid: AlurescueA70Du $A_{17}CATTACA_{18}GA_{17}CACACA_{18}$ (T)
Chromosome: 9
5' position: 34,817,513
Strand: minus
DR: AAGAGATTGTC
ENDOsite: TCTT/AT

Empty site:

ATTCTCACTTTTAAAAAGAACTTCAATCCTATATCTTGTTCCATACACAAAATTACTAGTCCAATAACAAAA
TGATCCATAAAAGAAAAAAATTGATAAATTCTACCTCATCAGAATTAAGAACTTCTGTTCTTTAAAAGACAT
GGTTGAGAGAATGAAAGGACAAGCCACGCTCTGGTAGAAAATATTTGCAAATCACGTGTCTGATAAAGAACT
TGTATTCAGAATATATAAAGACCTCTCAAAACTCATTAAGTAATTTAAAAAAACAAACAACTCCCACAAAAA
GAAGATAT↑**AAGAGATTGTC**↓CCCAGGACTTTGGGAGGTGAAAGCAGGAGGATCGTTTGAGGCCAAGAGTTT
GAGACCAGCCTGGGCAACATAGTGAGACCCTGTCTCTACAAAAAATAAAAAAATTAGCTGGGCACAGTGATG
TGCACCTGTAGTCCCAGCTACTTGGGAGACTGCGACAGGAAGATGGCTTGACCCCAGGAGTTTGAGGCTGCA
GTGAGCTATGATTGTGCCACTACACTCCAGCC

Filled site:

ATTCTCACTTTTAAAAAGAACTTCAATCCTATATCTTGTTCCATACACAAAATTACTAGTCCAATAACAAAA
TGATCCATAAAAGAAAAAAATTGATAAATTCTACCTCATCAGAATTAAGAACTTCTGTTCTTTAAAAGACAT
GGTTGAGAGAATGAAAGGACAAGCCACGCTCTGGTAGAAAATATTTGCAAATCACGTGTCTGATAAAGAACT
TGTATTCAGAATATATAAAGACCTCTCAAAACTCATTAAGTAATTTAAAAAAACAAACAACTCCCACAAAAA
GAAGATATAAGAGATTGTC**GGCCGGGCGCGGTGGCTCACGCCTGTAATCCCAGCACTTTGGGAGGCCGAGGC
GGGCGGATCACGAGGTCAGGAGATCGAGACCATCCTGGCTAACACGGTGAAACCCCGTCTCTACTAAAAAAA
AATACAAAAAATTAGCCGGGCGTGGTAGCGGGCGCCTGTAGTCCCAGCTACTCGGGAGGCTGAGGCAGGGGA
ATGGCGTGAACCCGGGAGGCGGAGCTTGCAGTGAGCCGAGATCCC**... **[NEOcassette][Atail]**
AAGAGATTGTCCCCAGGACTTTGGGAGGTGAAAGCAGGAGGATCGTTTGAGGCCAAGAGTTTGAGACCAGCC
TGGGCAACATAGTGAGACCCTGTCTCTACAAAAAATAAAAAAATTAGCTGGGCACAGTGATGTGCACCTGTA
GTCCCAGCTACTTGGGAGACTGCGACAGGAAGATGGCTTGACCCCAGGAGTTTGAGGCTGCAGTGAGCTATG
ATTGTGCCACTACACTCCAGCC

LINE-1 at the 5'; Alu at the 3'.

CLONE 218

[GG35,GG36]

Driver: ORF2
Plasmid: AlurescueA70Du $A_{17}CATTACA_{18}GA_{17}CACACA_{18}$ (T)
Chromosome: 9
5’ position: 123,725,907
Strand: minus
DR: AAAAGCCGTATGTG
ENDOsite: TTTT/AA

Empty site:

GACTCAAAGATCTCTTGCAACTCTAAAGTCAGAGATTTTAACCCATACATTGTAGAAAAGCAAATAAATGGA
GACCCAAATTTAAAAATTAAGGTCTACTAAGGAAGAGTGTCCTTTCTTAAAGACTCTTATTCACTTATTTAT
TATATTTTCTAACCTCTTTTTTATTTTAGCTACAAGCCCAGCAGGGAAGAATCATCATCTGGATCCTCTCAT
GCGGTGATGGACATCTCCTTGCCTACTGGAATCAGTGCAAATGAAGAAGACTT↑**AAAAGCCGTATGTG**↓TTT
TCTTTTAAATCGTGTTGTGAATGCTTCATTTTCTTTTCTGAATTATTTTATAGACAATTTTACTTTCTTTCA
TTTGAATATACCACTTTTATAGAATCACCCAGAATATTTTTTTTTTTTTGGAGGTGGATTTTCACTCTTGTT
GCCCAGGCTGGAGTGTGATGCAGTCTTGGCTCATTGCAACCGCACCTCTCGGG

Filled site:

GACTCAAAGATCTCTTGCAACTCTAAAGTCAGAGATTTTAACCCATACATTGTAGAAAAGCAAATAAATGGA
GACCCAAATTTAAAAATTAAGGTCTACTAAGGAAGAGTGTCCTTTCTTAAAGACTCTTATTCACTTATTTAT
TATATTTTCTAACCTCTTTTTTATTTTAGCTACAAGCCCAGCAGGGAAGAATCATCATCTGGATCCTCTCAT
GCGGTGATGGACATCTCCTTGCCTACTGGAATCAGTGCAAATGAAGAAGACTTAAAAGCCGTATGTGA**GGCC**
GGGCGCGGTGGCTCACGCCTGTAATCCCAGCACTTTGGGAGGCCGAGGCGGGCGGATCACGAGGTCAGGAGA
TCGAGACCATCCTGGCTAACACGGTGAAACCCCGTCTCTACTAAAAAAAAATACAAAAAATTAGCCGGGCGT
GGTAGCGGGCGCCTGTAGTCCCAGCTACTCGGGAGGCTGAGGCAGGGGAATGGCGTGAACCCGGGAGGCGGA
GCTTGCAGTGAGCCGAGATCCCGCCACTGCACT…[NEOcassette]*AAAAAAAAAAAAAAAAAAAAAA*
AAAAAAAAAAAAAAAAAAAAGCCGTATGTGTTTTCTTTTAAATCGTGTTGTGAATGCTTCATTTTCTTTTC
TGAATTATTTTATAGACAATTTTACTTTCTTTCATTTGAATATACCACTTTTATAGAATCACCCAGAATATT
TTTTTTTTTTTGGAGGTGGATTTTCACTCTTGTTGCCCAGGCTGGAGTGTGATGCAGTCTTGGCTCATTGCA
ACCGCACCTCTCGGG

Alu at the 3'.

An extra A added immediately 5’ of Alu.

CLONE 219

[GG28,GG46]

Driver: ORF2
Plasmid: AlurescueA70Du $A_{17}CATTACA_{18}GA_{17}CACACA_{18}$ (T)
Chromosome: 9
5’ position: 129,894,237
Strand: minus
DR: AGAAATGCAAATTCCC
ENDOsite: TTCT/AA

Empty site:

TTTCACATTCAGACATCACAATGTGGCTTAAATGCTACATTTTCCCAGGGTCAATGACACTCAGAAGAGTGA
AAGGGCAAGACAGCCAGCTCCCAGGAAATGCCTGAAAAAAGCCAAACCGCATTTTTCCTGCATGATACGTAA
CCAGCATCTGCACTCACCAAACAGCTCTCAGATGCAGGTTATGGTCACTGCCAGCCCAGTCCTGACTCTCCT
TCATGGCAAACTCCTCTATCTCGAGGAGTGGTTCCCACAGCAAGGGCCTCATGTGAACTTGTT↑**AGAAATGC
AAATTCCC**↓GGGCCCCAGATTTACTGAATCAGAAACTGGGGGTGGGGCCCAGCAAACTTTTAACAAGCCCTC
AGTTGATCTTGAAGCGCAAAAGCTCGAGAACCACTGACCTAGGATGGGCTGAAGTGGTGAGCACAAGGACCC
ACCACTGACTGGGTGCCTGCTATGCATATCTCAAGCACACACCTCCAGGAACCTTCACAATAGTT

Filled site:

TTTCACATTCAGACATCACAATGTGGCTTAAATGCTACATTTTCCCAGGGTCAATGACACTCAGAAGAGTGA
AAGGGCAAGACAGCCAGCTCCCAGGAAATGCCTGAAAAAAGCCAAACCGCATTTTTCCTGCATGATACGTAA
CCAGCATCTGCACTCACCAAACAGCTCTCAGATGCAGGTTATGGTCACTGCCAGCCCAGTCCTGACTCTCCT
TCATGGCAAACTCCTCTATCTCGAGGAGTGGTTCCCACAGCAAGGGCCTCATGTGAACTTGTTAGAAATGCA
AATTCCC**GGCCGGGCGCGGTGGCTCACGCCTGTAATCCCAGCACTTTGGGAGGCCGAGGCGGGCGGATCACG
AGGTCAGGAGATCGAGACCATCCTGGCTAACACGGTGAAACCCCGTCTCTACTAAAAAAAAATACAAAAAAT
TAGCCGGGCGTGGTAGCGGGCGCCTGTAGTCCCAGCTACTCGGGAGGCTGAGGCAGGGGAATGGCGTGAACC
CGGGAGGCGGAGCTTGCAGTGAGCCGAGATCCCGCCA…[NEOcassette][Atail]**AGAAATGCAAAT
TCCCGGGCCCCAGATTTACTGAATCAGAAACTGGGGGTGGGGCCCAGCAAACTTTTAACAAGCCCTCAGTTG
ATCTTGAAGCGCAAAAGCTCGAGAACCACTGACCTAGGATGGGCTGAAGTGGTGAGCACAAGGACCCACCAC
TGACTGGGTGCCTGCTATGCATATCTCAAGCACACACCTCCAGGAACCTTCACAATAGTT

hAT-Charlie DNA element flanking the insertion site.

CLONE 220

[LZ58]

Driver: ORF2
Plasmid: AlurescueA70Du $A_{17}CATTACA_{18}GA_{17}CACACA_{18}$ (T)
Chromosome: 9
5' position: 131,304,493
Strand: minus
DR: AGAATGGGTCACTTCA
ENDOsite: TTCT/AT

Empty site:

GACATGACTTCTCCCAAATACAGCTGTCTTCAGTTCCTTTATTATTGCCTCTTTGCAGCAGCGGTGATGGTG
GGTGATGGAGTGACATCAGGAGCGCCAGAAGGAGGAAGTCAGAAAGCCCTTGGGGACAGGAATGTCCTTGTG
TTGCAAACATTTCTGGTCAGGGAAAGATGAAGAAAACAGAATGAGTTTCTGATAACAGTGTTCCCAACACTT
AATGGTTAAAAGAAGCTTTATTATTAATTTTTTTTGGCACCTTAATTAACAATTCAT↑**AGAATGGGTCACTT**
CA↓GGCCACTCAAGAGTACCAGTGAACACTCCCCCACAAACACACCCTGCCACAAGACATTTAGCACAGAGG
AACAGATCCATGGCCACTGCCTCTGCAGTATCAAAGAGAATTAGTCTTTCCACAAAACAAATTTTAACAGCC
AATCTCTGGATTTCTGTAGTGGCTTTAGTCAGGCATATTTATCATCATATTAGCAGTGT

Filled site:

GACATGACTTCTCCCAAATACAGCTGTCTTCAGTTCCTTTATTATTGCCTCTTTGCAGCAGCGGTGATGGTG
GGTGATGGAGTGACATCAGGAGCGCCAGAAGGAGGAAGTCAGAAAGCCCTTGGGGACAGGAATGTCCTTGTG
TTGCAAACATTTCTGGTCAGGGAAAGATGAAGAAAACAGAATGAGTTTCTGATAACAGTGTTCCCAACACTT
AATGGTTAAAAGAAGCTTTATTATTAATTTTTTTTGGCACCTTAATTAACAATTCATAGAATGGGTCACTTC
A**GGCCGGGCGCGGTGGCTCACGCCTGTAATCCCAGCACTTTGGGAGGCCGAGGCGGGCGGATCACGAGGTCA**
GGAGATCGAGACCATCCTGGCTAACACGGTGAAACCCCGTCTCTACTAAAAAAAAATACAAAAAATTAGCCG
GGCGTGGTAGCGGGCGCCTGTAGTCCCAGCTACTCGGGAGGCTGAGGCAGGGGAATGGCGTGAACCCGGGAG
GCGGAGCTTGCAGTGAGCCGAGATCCCGCCACTGCACTCC…[NEOcassette][Atail]AGAATGGGT
CACTTCAGGCCACTCAAGAGTACCAGTGAACACTCCCCCACAAACACACCCTGCCACAAGACATTTAGCACA
GAGGAACAGATCCATGGCCACTGCCTCTGCAGTATCAAAGAGAATTAGTCTTTCCACAAAACAAATTTTAAC
AGCCAATCTCTGGATTTCTGTAGTGGCTTTAGTCAGGCATATTTATCATCATATTAGCAGTGT

No repetitive sequences in the immediate proximity.

CLONE 221

[LZ24]

Driver: ORF2
Plasmid: AlurescueA70Du $A_{17}CATTACA_{18}GA_{17}CACACA_{18}$ (T)
Chromosome: 10
5' position: 73,047,314
Strand: plus
DR: N/A
ENDOsite: N/A

Empty site:

TTCCAGCTGCAGGCAGTCCCAGGCCCCCATCACTCCCAGGGTGACCCTGGGGAAGTGGCAGCCCTTTCTGGA
CCTTCTTTGCCGTCTTACAATGAGGTGGGGCATAGACAGTGACTTCCCGGATCCCCTCTGGTGCTGACGGTT
ACAGCCCAGCTCTATCTGCCAGTTCTGAGTCCCTACACTGTAGGCAAAAGGGGTCTCACTAGGAAGGCCAGG
CTTCAGCAGAGGGGCCTCGTCAGGGTGAGGGACGGGTTTGGGGGAGGGCAGAGGGTT↑↓CCAGAGCCAAGCC
ATCCAGTTGACAGCTGCCGCCTTGCAGTGAATGGCGGCTGGTCCAGCTGGGCAGAGTGGTCACCCTGCTCCA
ACCGCTGTGGCCGAGGCTGGCAGAAGCGCACCCGGACCTGCACCAACCCCGCTCCACTCAACGGAGGGGCCT
TCTGCGAGGGCCAGGCATTCCAGAAGACCGCCTGCACCACCAT

Filled site:

TTCCAGCTGCAGGCAGTCCCAGGCCCCCATCACTCCCAGGGTGACCCTGGGGAAGTGGCAGCCCTTTCTGGA
CCTTCTTTGCCGTCTTACAATGAGGTGGGGCATAGACAGTGACTTCCCGGATCCCCTCTGGTGCTGACGGTT
ACAGCCCAGCTCTATCTGCCAGTTCTGAGTCCCTACACTGTAGGCAAAAGGGGTCTCACTAGGAAGGCCAGG
CTTCAGCAGAGGGGCCTCGTCAGGGTGAGGGACGGGTTTGGGGGAGGGCAGAGGGTT**GGCCGGGCGCGGTGG**
CTCACGCCTGTAATCCCAGCACTTTGGGAGGCCGAGGCGGGCGGATCACGAGGTCAGGAGATCGAGACCATC
CTGGCTAACACGGTGAAACCCCGTCTCTACTAAAAAAAAAAAAATACAAAAAATTAGCCGGGCGTGGTAGCGG
GCGCCTGTAGTCCCAGCTACTCGGGAGGCTGAGGCAGGGGAATGGCGTGAACCCGGGAGGCGGAGCTTGCAG
TGAGCCGAGATCCCGCC…[NEOcassette][Atail and 3' unsequenced]

No repetitive sequences in the immediate proximity.

CLONE 222

[LZ1a,2a,3a,5a]

Driver: ORF2
Plasmid: AlurescueA70Du $A_{17}CATTACA_{18}GA_{17}CACACA_{18}$ (T)
Chromosome: 10
5' position: 91,034,339
Strand: minus
DR: N/A
ENDOsite: N/A

Empty site:

TTGTGTGTTATAGGAGGAAGAAAACTCCAATAAAGTCTGTCTCAACAGAGGTCACCTCCCACCCATCAGACA
CGGAAATTCCAGTACCTGCTGGGAGTTCTGGTGGCCATCCTTATCCCTAGTCTCTTGTCATTGTTCAAGGAT
TAGGGAAAATTCCTCTCAACCTGCTAGATTTCAGCAGCAATAAACGGGATTGTTTATAGGGTCAGTGAGCCC
AGGATACTAAGGGACAGACATCAGTCAAAAGCATTTT↑↓CCAAAAAAATCCTTTATATTTTAATCAAATCAC
TATAAAGACATGATGTGTCCTAGTTTCTCAGATGTTCAACCCAACAGTCTCTTTCTTTAACTTCTGGGCTGC
CTTTCAGAACTGAGCAGAGTTGTATCCAACACAAAAGACTGTCTGGACTCAGGCACCGTACACATACCCCGT
CACTGCTCCTCTCCATTGAGGTA

Filled site:

TTGTGTGTTATAGGAGGAAGAAAACTCCAATAAAGTCTGTCTCAACAGAGGTCACCTCCCACCCATCAGACA
CGGAAATTCCAGTACCTGCTGGGAGTTCTGGTGGCCATCCTTATCCCTAGTCTCTTGTCATTGTTCAAGGAT
TAGGGAAAATTCCTCTCAACCTGCTAGATTTCAGCAGCAATAAACGGGATTGTTTATAGGGTCAGTGAGCCC
AGGATACTAAGGGACAGACATCAGTCAAAAGCATTTT**GGCCGGGCGCGGTGGCTCACGCCTGTAATCCCAGC**
ACTTTGGGAGGCCGAGGCGGGCGGATCACGAGGTCAGGAGATCGAGACCATCCTGGCTAACACGGTGAAACC
CCGTCTCTACTAAAAAAAAATACAAAAAATTAGCCGGGCGTGGTAGCGGGCGCCTGTAGTCCCAGCTACTCG
GGAGGCTGAGGCAGGGGAATGGCGTGAACCCGGGAGGCGGAGCTTGCAGTGAGCCGAGATCCCGCCACTGCA
CTCCAGCCTG…[NEOcassette][Atail and 3' unsequenced]

LTR flanking the insertion site.

CLONE 223

[GG45]

Driver: ORF2
Plasmid: AlurescueA70Du $A_{17}CATTACA_{18}GA_{17}CACACA_{18}$ (T)
Chromosome: 10
5’ position: 91,391,732
Strand: minus
DR: AAAAAAAAAAAAAACA
ENDOsite: TTTT/AA

Empty site:

AATTCAGACTCATGGTTTTTATTCCAAAAGTCACTGTGTTCCACTTGTCCTCTTATTGTCCTGAGTGAGCGT
TTTGGAAAGTGTTGGGATTTTAACAACTTTTTCTGCATTTTAAACATTATACAAAACTAGAGAATATAGCAT
AATGAACTGCCAAGTACTTATCACTCAGCTTCTATAATTATCAAATCATGTCCAATTTTATAATATTGAAGC
AGACCTCAGAAATCATATGTTTAGTATTAAATGTTTTTGTCTTTATTTCCAAAAGAAAAGAACCCTTTT↑**AA**
AAAAAAAAAAAACA↓CACACACACACACACACAATCTGTTATTTGCTGTAAAAGTTAGCAGTGTATTATATC
CAGTTGAGACTATTGTGTTTTATGATGCACAATTAGATAAATTCAGTTATCTAACCACAATTAAAATTGTTA
CATCTCATTTGTAGGTGGATAATTGAAAACCTGATTTGTGTTGCCCCTGGAGAATCCTTTCATTTGCCCAT

Filled site:

AATTCAGACTCATGGTTTTTATTCCAAAAGTCACTGTGTTCCACTTGTCCTCTTATTGTCCTGAGTGAGCGT
TTTGGAAAGTGTTGGGATTTTAACAACTTTTTCTGCATTTTAAACATTATACAAAACTAGAGAATATAGCAT
AATGAACTGCCAAGTACTTATCACTCAGCTTCTATAATTATCAAATCATGTCCAATTTTATAATATTGAAGC
AGACCTCAGAAATCATATGTTTAGTATTAAATGTTTTTGTCTTTATTTCCAAAAGAAAAGAACCCTTTTAAA
AAAAAAAAAAACA**GGCCGGGCGCGGTGGCTCACGCCTGTAATCCCAGCACTTTGGGAGGCCGAGGCGGGCGG**
ATCACGAGGTCAGGAGATCGAGACCATCCTGGCTAACACGGTGAAACCCCGTCTCTACTAAAAAAAAATACA
AAAAATTAGCCGGGCGTGGTAGCGGGCGCCTGTAGTCCCAGCTACTCGGGAGGCTGAGGCAGGGGAATGGCG
TGAACCCGGGAGGCGGAGCTTGCAGTGAGCCGAGATCCCGCCA…[NEOcassette][Atail]AAAAAA
AAAAAAAACACACACACACACACACACAATCTGTTATTTGCTGTAAAAGTTAGCAGTGTATTATATCCAGTT
GAGACTATTGTGTTTTATGATGCACAATTAGATAAATTCAGTTATCTAACCACAATTAAAATTGTTACATCT
CATTTGTAGGTGGATAATTGAAAACCTGATTTGTGTTGCCCCTGGAGAATCCTTTCATTTGCCCAT

LINE-1 at the 5'.

CLONE 224

[LZ37]

Driver: ORF2
Plasmid: AlurescueA70Du $A_{17}CATTACA_{18}GA_{17}CACACA_{18}$ (T)
Chromosome: 10
5’ position: 112,270,251
Strand: plus
DR: AAGACTCATGGAC
ENDOsite: TCTT/AA

Empty site:

GTCTCGCCCAACTTTGGCTTCATGGGCCAGCTCCTGCAGTACGAATCTGAGATCCTGCCCTCCACGCCCAAC
CCCCAGCCTCCCTCCTGCCAAGGGGAGGCAGCAGGCTCTTCACTGATAGGCCATTTGCAGACACTGAGCCCT
GACATGCAGGGTGCCTACTGCACATTCCCTGCCTCGGTGCTGGCACCGGTGCCTACCCACTCAACAGTCTCA
GAGCTCAGCAGAAGCCCTGTGGCAACGGCCACATCCTGCTAAAACTGGGATGGAGGAATCGGCCCAGCCCCA
AGAGCAACTGTGATTTTTTGTTTTT↑**AAGACTCATGGAC**↓ATTTCATACCTGTGCAATACTGAAGACCTCATT
CTGTCATGCTGCCCCAGTGAGATAGTGAGTGGTCACCAGGCTTGCAAATGAACTTCAGACGGACCTCAGGGT
AGGTTCTCGGGACTGAAGGAAGGCCAAGCCATTACGGGAGCACAGCATGTGCTGACTACTGTACTTCCAGAC
CCCTGCCCTCTTGGGACTGCCCA

Filled site:

GTCTCGCCCAACTTTGGCTTCATGGGCCAGCTCCTGCAGTACGAATCTGAGATCCTGCCCTCCACGCCCAAC
CCCCAGCCTCCCTCCTGCCAAGGGGAGGCAGCAGGCTCTTCACTGATAGGCCATTTGCAGACACTGAGCCCT
GACATGCAGGGTGCCTACTGCACATTCCCTGCCTCGGTGCTGGCACCGGTGCCTACCCACTCAACAGTCTCA
GAGCTCAGCAGAAGCCCTGTGGCAACGGCCACATCCTGCTAAAACTGGGATGGAGGAATCGGCCCAGCCCCA
AGAGCAACTGTGATTTTTTGTTTTTAAGACTCATGGAC**GGCCGGGCGCGGTGGCTCACGCCTGTAATCCCAGC**
ACTTTGGGAGGCCGAGGCGGGCGGATCACGAGGTCAGGAGATCGAGACCATCCTGGCTAACACGGTGAAACC
CCGTCTCTACTAAAAAAAAAAAAAAATACAAAAAATTAGCCGGGCGTGGTAGCGGGCGCCTGTAGTCCCAGCT
ACTCGGGAGGCTGAGGCAGGGGAATGGCGTGAACCCGGGAGGCGGAGCTTGCAGTGAGCCGAGATCCCGCCA
CTGCACTC…[NEOcassette][Atail]AAGACTCATGGACATTTCATACCTGTGCAATACTGAAGACC
TCATTCTGTCATGCTGCCCCAGTGAGATAGTGAGTGGTCACCAGGCTTGCAAATGAACTTCAGACGGACCTC
AGGGTAGGTTCTCGGGACTGAAGGAAGGCCAAGCCATTACGGGAGCACAGCATGTGCTGACTACTGTACTTC
CAGACCCCTGCCCTCTTGGGACTGCCCA

No repetitive sequences in the immediate proximity.

CLONE 225

[LZ59]

Driver: ORF2
Plasmid: AlurescueA70Du $A_{17}CATTACA_{18}GA_{17}CACACA_{18}$ (T)
Chromosome: 10
5' position: 117,012,451
Strand: minus
DR: GAAAAACTCATCAT
ENDOsite: TTTC/AT

Empty site:

TCAACTTCTCCCTTTGTCCAATTCTGCATCATCCCCCTACCTCTTGCAAAGGCATTGATTGTAAGGGTACTC
CCAATTAACATCATGAATACTAAACTCTACCTCAGAGTCTGCTTTCCAGAGGACTCAAACTCTGACTTGTTT
CAGGAATGGTATACTAAAGTAAGAAATAAGTTGGAGTTTGAGAGCTGGATCATACATTGCCAGGCTAGCAAT
↑**GAAAAACTCATCAT**↓TGGTGGTAGGTGGAGCACAGATAGCTCTTGGCACAAAGTGGCCATTAAGCAACTGC
AAAAACTTAAACTAGTGATAAAGTAGGAAGGCATGCCAATAGAAGGGAATAGTATAGCAGGAGTGAAGTACT
AGATGCTTGAAAAACATGAGGAAGGGTAACTATTAAGATACTAGAATTGCATGTCTACTGCTTAGCCCCAGT

Filled site:

TCAACTTCTCCCTTTGTCCAATTCTGCATCATCCCCCTACCTCTTGCAAAGGCATTGATTGTAAGGGTACTC
CCAATTAACATCATGAATACTAAACTCTACCTCAGAGTCTGCTTTCCAGAGGACTCAAACTCTGACTTGTTT
CAGGAATGGTATACTAAAGTAAGAAATAAGTTGGAGTTTGAGAGCTGGATCATACATTGCCAGGCTAGCAAT
GAAAAACTCATCATA**GGCCGGGCGCGGTGGCTCACGCCTGTAATCCCAGCACTTTGGGAGGCCGAGGCGGGC
GGATCACGAGGTCAGGAGATCGAGACCATCCTGGCTAACACGGTGAAACCCCGTCTCTACTAAAAAAAAATA
CAAAAAAAAATTAGCCGGGCGTGGTAGCGGGCGCCTGTAGTCCCAGCTACTCGGGAGGCTGAGGCAGGGGAA
TGGCGTGAACCCGGGAGGCGGAGCTTGCAGTGAGCCGAGATCCCGCCACTGCACTCC…[NEOcassette]
[Atail]**GAAAAACTCATCATTGGTGGTAGGTGGAGCACAGATAGCTCTTGGCACAAAGTGGCCATTAAGCA
ACTGCAAAAACTTAAACTAGTGATAAAGTAGGAAGGCATGCCAATAGAAGGGAATAGTATAGCAGGAGTGAA
GTACTAGATGCTTGAAAAACATGAGGAAGGGTAACTATTAAGATACTAGAATTGCATGTCTACTGCTTAGCC
CCAGT

LTR flanking the insertion site.

An extra A added immediately 5' of Alu.

CLONE 226

[BC1-16,31,34]

Driver: ORF2
Plasmid: AlurescueA70Du $A_{17}CATTACA_{18}GA_{17}CACACA_{18}$ (T)
Chromosome: 10
5' position: 127,918,647
Strand: minus
DR: N/A
ENDOsite: N/A

Empty site:

TACAGCATTCGACTCAGCAGTTTCACATCAGGGATTTATCCTCAGGAAAGTATAGTGTATCCCAAGGCATAT
GCAAAAGTATATTCATTGCAGTGTGGTCATGATAGGCATTGAAGATGTTGTATTGAACGTCAGATAAATGCC
AGGAGCAGTGTTAGCTGCAGGAAATATAAGGTGTGAGGGGCGGGTAGAAACAGATCCAGTCCCTGACCCCTC
TACGAGTGTACCCTGCATCTGAGGTACAGATAAGTAATTTGGCCATAAGAGCCCAATGCCATGAGTGCTTTG
GGGGCTGGAAGATCACACAGAAAAGGAACCTTCCCTGGGCTTGGTAAGACAGGAAAGGAGAGTACCACAAGA
AGGCTTCCTG↑↓GAGGAAGTTGCAGCTCAGCCAAAATGGAAGGATCAGTAAGAATTAGCCACATATAAAGGG
CAGGTAGGAATTGGCCAAGCCAAGGATGTGGACTGACACCTTAAGGGCCAAACACTGCTGGAGAAGCACAAC
TCGTTGAGCACCTGCATGTGGGATACTAGAGTGCGGCAGCGGCCAGGTCACAAGGGCCTTGAGAGTCT

Filled site:

TACAGCATTCGACTCAGCAGTTTCACATCAGGGATTTATCCTCAGGAAAGTATAGTGTATCCCAAGGCATAT
GCAAAAGTATATTCATTGCAGTGTGGTCATGATAGGCATTGAAGATGTTGTATTGAACGTCAGATAAATGCC
AGGAGCAGTGTTAGCTGCAGGAAATATAAGGTGTGAGGGGCGGGTAGAAACAGATCCAGTCCCTGACCCCTC
TACGAGTGTACCCTGCATCTGAGGTACAGATAAGTAATTTGGCCATAAGAGCCCAATGCCATGAGTGCTTTG
GGGGCTGGAAGATCACACAGAAAAGGAACCTTCCCTGGGCTTGGTAAGACAGGAAAGGAGAGTACCACAAGA
AGGCTTCCTG**GGCCGGGCGCGGTGGCTCACGCCTGTAATCCCAGCACTTTGGGAGGCCGAGGCGGGCGGATC
ACGAGGTCAGGAGATCGAGACCATCCTGGCTAACACGGTGAAACCCCGTCTCTACTAAAAAAAAATACAAAA
AATTAGCCGGGCGTGGTAGCGGGCGCCTGTAGTCCCAGCTACTCGGGAGGCTGAGGCAGGGGAATGGCGTGA
ACCCGGGAGGCGGAGCTTGCAGTGAGCCGAGATCCCGCCACTGCACTCCAGCCTG...[NEOcassette][A
tail and 3' unsequenced]**

LINE-1 at the 5'; L2 flanking the insertion site.

CLONE 227
[LZ9]

Driver: ORF2
Plasmid: AlurescueA70Du $A_{17}CATTACA_{18}GA_{17}CACACA_{18}$ (T)
Chromosome: 11
5’ position: 28,671,379
Strand: plus
DR: AGGAGTACTTTTC
ENDOsite: TCCT/AT

Empty site:

CTAAACAATGGACAGTAAGAGACAAATTCTAAACAAGGGTTAGTAACAAAATTCGAACCAATTTTGACATTG
TATTTTAGTGTTCCTTACTACTCTAAACTCTGTAAAAGCAGGAACTGTATCTGCCCAGTTTTTATTGCTTTT
TACATTCGTCTTTTCAACTAGGAATGCATTCCACTTCAAATAACAGAATGCCCTACTGGCAGTAGCTTAGCT
AAAT↑**AGGAGTACTTTTC**↓TCTCATAAGTTTGGAGATAGTAGCTGCTGGTCTGGTTCAATTATTCGACAATA
TTAACAAGAACCTGGACAATTCTTTGTTTTTCTGGTCTACCTTTCCTGAGCTTTTCTACCATAGTTTTATCA
TTTATGCTCTCAAGATGGCTGCAACAGATCCAGAAGTCATGACTTCATTCAAAGTCAGAAAGCAAGAGGACT
GCA

Filled site:

CTAAACAATGGACAGTAAGAGACAAATTCTAAACAAGGGTTAGTAACAAAATTCGAACCAATTTTGACATTG
TATTTTAGTGTTCCTTACTACTCTAAACTCTGTAAAAGCAGGAACTGTATCTGCCCAGTTTTTATTGCTTTT
TACATTCGTCTTTTCAACTAGGAATGCATTCCACTTCAAATAACAGAATGCCCTACTGGCAGTAGCTTAGCT
AAATAGGAGTACTTTTC**GGCCGGGCGCGGTGGCTCACGCCTGTAATCCCAGCACTTTGGGAGGCCGAGGCGG**
GCGGATCACGAGGTCAGGAGATCGAGACCATCCTGGCTAACACGGTGAAACCCCGTCTCTACTAAAAAAAAA
ATACAAAAAATTAGCCGGGCGTGGTAGCGGGCGCCTGTAGTCCCAGCTACTCGGGAGGCTGAGGCAGGGGAA
TGGCGTGAACCCGGGAGGCGGAGCTTGCAGTGAGCCGAGATCCCGCCACTGCACTC... **[NEOcassette][**
Atail]AGGAGTACTTTTCTCTCATAAGTTTGGAGATAGTAGCTGCTGGTCTGGTTCAATTATTCGACAATA
TTAACAAGAACCTGGACAATTCTTTGTTTTTCTGGTCTACCTTTCCTGAGCTTTTCTACCATAGTTTTATCA
TTTATGCTCTCAAGATGGCTGCAACAGATCCAGAAGTCATGACTTCATTCAAAGTCAGAAAGCAAGAGGACT
GCA

LTR flanking the insertion site.

CLONE 228

[GG17]

Driver: ORF2
Plasmid: AlurescueA70Du $A_{17}CATTACA_{18}GA_{17}CACACA_{18}$ (T)
Chromosome: 11
5' position: 46,640,505
Strand: plus
DR: TAAAGTGTTGGGGT
ENDOsite: TTTA/GA

Empty site:

```
TGTTTTATGACTGTTAGTCCTTTTCATGTGTGCATGTAGTGGCTCCAGCTCAATTATGAGGTTTTTTTTTTG
TTGTTTTCAACTCTCCATTTATCAGCTCAGGAGAAGTCTTTTAGTTTTTTAGATTTTAAAATTTTTATTATT
ATTATTAGAGATAAGGTATTGCTGTGTTACCCAAGCTGGAGTACAGTGGCGCCTTCATAGCTAACTTTAATC
TCAAACTCCTGGGCTCAGGGAATCCTCCCACCTCAGCCTTC↑TAAAGTGTTGGGGT↓TACATGCATAAGCCA
CAGAGTCCGGCCCAGGAGCTTAATCTCGAAGGAAGTTTACTTGAACTTTAGGTGTGGAATATTCGAAAGTAA
CTTCCAACATGGGGTGTGGAGCTAAGTAAGATGAATTTACATAAACCGTCATTTTTCCTGCATGTGCTTAGT
GGAGGAGAGTGCATAGAGTTGCTGGTCATTGCTTCCTATCA
```

Filled site:

```
TGTTTTATGACTGTTAGTCCTTTTCATGTGTGCATGTAGTGGCTCCAGCTCAATTATGAGGTTTTTTTTTTG
TTGTTTTCAACTCTCCATTTATCAGCTCAGGAGAAGTCTTTTAGTTTTTTAGATTTTAAAATTTTTATTATT
ATTATTAGAGATAAGGTATTGCTGTGTTACCCAAGCTGGAGTACAGTGGCGCCTTCATAGCTAACTTTAATC
TCAAACTCCTGGGCTCAGGGAATCCTCCCACCTCAGCCTTCTAAAGTGTTGGGGTGGCCGGGCGCGGTGGCT
CACGCCTGTAATCCCAGCACTTTGGGAGGCCGAGGCGGGCGGATCACGAGGTCAGGAGATCGAGACCATCCT
GGCTAACACGGTGAAACCCCGTCTCTACTAAAAAAAAAATACAAAAAATTAGCCGGGCGTGGTAGCGGGCGC
CTGTAGTCCCAGCTACTCGGGAGGCTGAGGCAGGGGAATGGCGTGAACCCGGGAGGCGGAGCTTGCAGTGAG
CCGAGATCCCGCCACTGCACTCC...[NEOcassette][Atail]TAAAGTGTTGGGGTTACATGCATAAG
CCACAGAGTCCGGCCCAGGAGCTTAATCTCGAAGGAAGTTTACTTGAACTTTAGGTGTGGAATATTCGAAAG
TAACTTCCAACATGGGGTGTGGAGCTAAGTAAGATGAATTTACATAAACCGTCATTTTTCCTGCATGTGCTT
AGTGGAGGAGAGTGCATAGAGTTGCTGGTCATTGCTTCCTATCA
```

Alu flanking the insertion site.

CLONE 229

[TZ20]

Driver: ORF2
Plasmid: AlurescueA70Du $A_{17}CATTACA_{18}GA_{17}CACACA_{18}$ (T)
Chromosome: 11
5’ position: 57,578,561
Strand: minus
DR: N/A
ENDOsite: N/A

Empty site:

ACTGAATTCACCCTATCTTATGCTCTAATGGGCATAAACCTCTAGGTTCCTATTCCCAAATAGAATCATGAA
AAAGCAACTTATAAGAGTTGAATAAATACCCTAGACAGTGAGACAAACTAGGATATTATGGCTTCTATTTGT
GTTCCAGAACAGGAGATTACAAAGGATCCAAATATTGACACTATCAGAAGAAAATACAGTTTCAGAGAATAG
TAAAAATTCCCTCAAAGGGCTACGGTAGATTTGGGCAAAAGTCAAAAAGGCCATCATC↑↓CTTCATCATCAC
CTGCACCTAAAGTATGTTATGACATAGAGGGATACCTGAGCTGTGTCGGCAACAAATGAATTGTAGACACAT
CATCCTTTTGAGGAGTAAACCAAATCTTTTTTTTTTTCTTTTTTTTTTGAGACAGGGTTTAGCTATGTTGCC
CAGGCTGGAGTGCAGCAGTGTGATGTCGGCTCACTGCAACCTCT

Filled site:

ACTGAATTCACCCTATCTTATGCTCTAATGGGCATAAACCTCTAGGTTCCTATTCCCAAATAGAATCATGAA
AAAGCAACTTATAAGAGTTGAATAAATACCCTAGACAGTGAGACAAACTAGGATATTATGGCTTCTATTTGT
GTTCCAGAACAGGAGATTACAAAGGATCCAAATATTGACACTATCAGAAGAAAATACAGTTTCAGAGAATAG
TAAAAATTCCCTCAAAGGGCTACGGTAGATTTGGGCAAAAGTCAAAAAGGCCATCATC**GGCCGGGCGCGGTG
GCTCACGCCTGTAATCCCAGCACTTTGGGAGGCCGAGGCGGGCGGATCACGAGGTCAGGAGATCGAGACCAT
CCTGGCTAACACGGTGAAACCCCGTCTCTACTAAAAAAAAAATACAAAAAATTAGCCGGGCGTGGTAGCGGG
CGCCTGTAGTCCCAGCTACTCGGGAGGCTGAGGCAGGGAATGGCGTGAACCCGGGAGGCGGAGCTTGCAGT
GAGCCGAGATCCCGCC…[NEOcassette][Atail and 3' unsequenced]**

Alu at the 3'.

CLONE 230

[LZ31]

Driver: ORF2
Plasmid: AlurescueA70Du $A_{17}CATTACA_{18}GA_{17}CACACA_{18}$ (T)
Chromosome: 11
5’ position: 74,484,409
Strand: minus
DR: AAAGAGGTATTAGAGA
ENDOsite: CTTT/AA

Empty site:

AATTTCATCTATCCTGCAGGCATTTTACAACCCTTCTGACCTCATCAGAAAAGTAAACATGTAAGAATATTG
TTGTGCCTGGGATTCAGTAGGTGAAACATCCAGGCAAGCTCAAAAAGAAAACTGGCCTGGTGGTACATTTGC
ACAAATGACCATCTTTCTCCCTCCTTTCATTCGTATTTAGCTTATACAATTATCATCTTATTTTATATTATC
CTAAATGCTATAATCAAGTCATCTAAGCTGATAAACACATATAAGAGGCTTT↑**AAAGAGGTATTAGAGA**↓AA
GAAATGCAACAGAACAGTAAATCTCAGTCCATGAATTGATCAGATTGGTGTTAAAGATTGACTAGTTGAGAA
ATTCTGAATTGAGGCTACAATAAAGTCATTTATCGCACAAATATTTATGAACCTACTATATGCCAGATATTG
CAGCACCATGGAAATCCAGGCCAATCCTACCCCAGGTCTCAGCAAATCGTAATC

Filled site:

AATTTCATCTATCCTGCAGGCATTTTACAACCCTTCTGACCTCATCAGAAAAGTAAACATGTAAGAATATTG
TTGTGCCTGGGATTCAGTAGGTGAAACATCCAGGCAAGCTCAAAAAGAAAACTGGCCTGGTGGTACATTTGC
ACAAATGACCATCTTTCTCCCTCCTTTCATTCGTATTTAGCTTATACAATTATCATCTTATTTTATATTATC
CTAAATGCTATAATCAAGTCATCTAAGCTGATAAACACATATAAGAGGCTTTAAAGAGGTATTAGAGA**GGCC
GGGCGCGGTGGCTCACGCCTGTAATCCCAGCACTTTGGGAGGCCGAGGCGGGCGGATCACGAGGTCAGGAGA
TCGAGACCATCCTGGCTAACACGGTGAAACCCCGTCTCTACTAAAAAAAAAATACAAAAAATTAGCCGGGCG
TGGTAGCGGGCGCCTGTAGTCCCAGCTACTCGGGAGGCTGAGGCAGGGGAATGGCGTGAACCCGGGAGGCGG
AGCTTGCAGTGAGCCGAGATCCCGCC… [NEOcassette] [Atail]** AAAGAGGTATTAGAGAAAGAAAT
GCAACAGAACAGTAAATCTCAGTCCATGAATTGATCAGATTGGTGTTAAAGATTGACTAGTTGAGAAATTCT
GAATTGAGGCTACAATAAAGTCATTTATCGCACAAATATTTATGAACCTACTATATGCCAGATATTGCAGCA
CCATGGAAATCCAGGCCAATCCTACCCCAGGTCTCAGCAAATCGTAATC

TcMar-Tigger DNA element at the 5’; L2 at the 3'.

CLONE 231

[LZ77]

Driver: ORF2
Plasmid: AlurescueA70Du $A_{17}CATTACA_{18}GA_{17}CACACA_{18}$ (T)
Chromosome: 11
5' position: 101,262,810
Strand: minus
DR: AAAACAATAAACT
ENDOsite: TTTT/AC

Empty site:

TATAAAACATAAAAATTATGCTGACAGAGAGGACATGCACCTTGCAGGAGGACCCTCAAAGGGCTGCTGGCA
GAAGATAAATCCTTATGCTATGAGATAACTTCTTTCATTTAAAAAAATTGCTTGAGTTCAACCAAAACCAAT
AAGAGACAGT↑**AAAACAATAAACT**↓ACCATCTGGATTTAAAATTACTCTTAAACTCTCCCTTTGTTGCTCCT
AGCTCTGGACCAGAAGTTTTCCCTTTCCCTAGCCAGCAGCACAGGGTGTAATTAAGCCTATGAACTCGTACC
TCCAAGCTACCAAGACACAAGAAAGGACCAAGGAAGGGCCACAGCCCGGAAATGCAGGGCAGATGGGGCCTC
CAAGGGAAG

Filled site:

TATAAAACATAAAAATTATGCTGACAGAGAGGACATGCACCTTGCAGGAGGACCCTCAAAGGGCTGCTGGCA
GAAGATAAATCCTTATGCTATGAGATAACTTCTTTCATTTAAAAAAATTGCTTGAGTTCAACCAAAACCAAT
AAGAGACAGTAAAACAATAAACT**GGCCGGGCGCGGTGGCTCACGCCTGTAATCCCAGCACTTTGGGAGGCCG
AGGCGGGCGGATCACGAGGTCAGGAGATCGAGACCATCCTGGCTAACACGGTGAAACCCCGTCTCTACTAAA
AAAAAATACAAAAAATTAGCCGGGCGTGGTAGCGGGCGCCTGTAGTCCCAGCTACTCGGGAGGCTGAGGCAG
GGGAATGGCGTGAACCCGGGAGGCGGAGCTTGCAGTGAGCCGAGATCCCGCCACTGCACTCCA**...**[NEOcas
sette][Atail]**AAAACAATAAACTACCATCTGGATTTAAAATTACTCTTAAACTCTCCCTTTGTTGCTC
CTAGCTCTGGACCAGAAGTTTTCCCTTTCCCTAGCCAGCAGCACAGGGTGTAATTAAGCCTATGAACTCGTA
CCTCCAAGCTACCAAGACACAAGAAAGGACCAAGGAAGGGCCACAGCCCGGAAATGCAGGGCAGATGGGGCC
TCCAAGGGAAG

No repetitive sequences in the immediate proximity.

CLONE 232

[GG49]

Driver: ORF2
Plasmid: AlurescueA70Du $A_{17}CATTACA_{18}GA_{17}CACACA_{18}$ (T)
Chromosome: 12
5' position: 6,582,235
Strand: plus
DR: AAAAGAAAAAAGAATGG
ENDOsite: TTTT/CT

Empty site:

CCCAGCAATGTGGGAGGCCAAGGAGGGCGGATCACTTGAAGCCAGGAGTTCAAGACCAACCTGGCTAACGCA
GCAAAATCCCGTCTCTACTAAAAATACAAAAATTAGCTGGGCATGGACGCCTGTAGTCCCAGCTACTCGGGA
GACTGAGGCAGGAGGATCACTTGAACCCGGGAGGCGGAGGCTGCAGTAAGCCGAGATCTTGCCACTGCACTC
CAACCTGGGCAACAGAATGAGACTGTCTCAAAAAAAAAAAAAAAAAGAAAGAAAGAAAGAAAAAGAAAAGAAA
AAAAAAG↑**AAAAGAAAAAAGAATGG**↓GAGGAAATATTTCTGGCAGCATGAATGCCTATTTATTCCCACCAAC
TTTCCCCTGAAAAAAAACTTAAAATCATTGAATGGTGACTCACGCCTATAATCCCAGCATTTTGGGAGGCCA
AAGCAGGAGGATCACTTGAGCCCAGGAGTTCAAGACTAGCCTGGACAACATGGTGAAATCCTGTCTCTACAA
GAAATAGATA

Filled site:

CCCAGCAATGTGGGAGGCCAAGGAGGGCGGATCACTTGAAGCCAGGAGTTCAAGACCAACCTGGCTAACGCA
GCAAAATCCCGTCTCTACTAAAAATACAAAAATTAGCTGGGCATGGACGCCTGTAGTCCCAGCTACTCGGGA
GACTGAGGCAGGAGGATCACTTGAACCCGGGAGGCGGAGGCTGCAGTAAGCCGAGATCTTGCCACTGCACTC
CAACCTGGGCAACAGAATGAGACTGTCTCAAAAAAAAAAAAAAAAAGAAAGAAAGAAAGAAAAAGAAAAGAAA
AAAAAAGAAAAGAAAAAAGAATGG**GGCCGGGCGCGGTGGCTCACGCCTGTAATCCCAGCACTTTGGGAGGCC
GAGGCGGGCGGATCACGAGGTCAGGAGATCGAGACCATCCTGGCTAACACGGTGAAACCCCGTCTCTACTAA
AAAAAAATACAAAAAATTAGCCGGGCGTGGTAGCGGGCGCCTGTAGTCCCAGCTACTCGGGAGGCTGAGGCA
GGGGAATGGCGTGAACCCGGGAGGCGGAGCTTGCAGTGAGCCGAGATCCCGCCACTGCACT…[NEOcasse
tte][Atail]**AAAAGAAAAAAGAATGGGAGGAAATATTTCTGGCAGCATGAATGCCTATTTATTCCCACC
AACTTTCCCCTGAAAAAAAACTTAAAATCATTGAATGGTGACTCACGCCTATAATCCCAGCATTTTGGGAGG
CCAAAGCAGGAGGATCACTTGAGCCCAGGAGTTCAAGACTAGCCTGGACAACATGGTGAAATCCTGTCTCTA
CAAGAAATAGATA

Alu at the 5' and 3'.

CLONE 233

[LZ42]

Driver: ORF2
Plasmid: AlurescueA70Du $A_{17}CATTACA_{18}GA_{17}CACACA_{18}$ (T)
Chromosome: 12
5’ position: 29,807,168
Strand: plus
DR: AAGAAAAATGTT
ENDOsite: TCTT/AA

Empty site:

TCTTAGAAACAACAAAAATAAATTGGACTTCATCAAAATAAAAAACTTACATGCTTCAAAGGACATCATCAA
GTAAGTGAAATGACAATATACAGAAAGAAAGAAAATGTGTTTATAAATCATAAGTCAGATAAGGGACTTGTA
TCTAGAATATATT↑**AAGAAAAATGTT**↓ACAACTTGGAAATAAAAAGACAAACAACCCAATTAAAAAGTGAAC
AAAGGATCTCCATCAACGTAGTTCCAAAGAAGACAGCAAAAGAGTCAGTACATACATAAGGAGATGTTCAAC
ATCATTAGCCACCAGGGAAATATAGGTACATCAAAACCACAATAACATACCATTCCCTACCCATTGGTATGG
CTATAATAAAA

Filled site:

TCTTAGAAACAACAAAAATAAATTGGACTTCATCAAAATAAAAAACTTACATGCTTCAAAGGACATCATCAA
GTAAGTGAAATGACAATATACAGAAAGAAAGAAAATGTGTTTATAAATCATAAGTCAGATAAGGGACTTGTA
TCTAGAATATATTAAGAAAAATGTT**GGCCGGGCGCGGTGGCTCACGCCTGTAATCCCAGCACTTTGGGAGGC
CGAGGCGGGCGGATCACGAGGTCAGGAGATCGAGACCATCCTGGCTAACACGGTGAAACCCCGTCTCTACTA
AAAAAAAATACAAAAAATTAGCCGGGCGTGGTAGCGGGCGCCTGTAGTCCCAGCTACTCGGGAGGCTGAGGC
AGGGGAATGGCGTGAACCCGGGAGGCGGAGCTTGCAGTGAGCCGAGATCCCGCCACTGCACTCC…[NEOca
ssette][Atail]**AAGAAAAATGTTACAACTTGGAAATAAAAAGACAAACAACCCAATTAAAAAGTGAAC
AAAGGATCTCCATCAACGTAGTTCCAAAGAAGACAGCAAAAGAGTCAGTACATACATAAGGAGATGTTCAAC
ATCATTAGCCACCAGGGAAATATAGGTACATCAAAACCACAATAACATACCATTCCCTACCCATTGGTATGG
CTATAATAAAA

LINE-1 flanking the insertion site.

CLONE 234

[LZ54]

Driver: ORF2
Plasmid: AlurescueA70Du $A_{17}CATTACA_{18}GA_{17}CACACA_{18}$ (T)
Chromosome: 12
5' position: 104,616,769
Strand: plus
DR: AAGAAAATTA
ENDOsite: TCTT/AA

Empty site:

ACTCAGGAAGCTGAGGCAGGAGAATCACTTGAACCTGAGAGGCGGAGGTTGCAGTGAGCCGAGATTGCACTG
CTGCACTCCAGCCTGGGTGAAATAGTGAGACTCTGTCTCAAAAAACAAACAAACAAACAAAACTAACTTTTG
GGATTATTGAGCAGAAATGTACGAGTTAACATGAAACTTCAGTGTCTAGGCTGTAATAAAATAGTAAACAGC
AAAGTCAAGTACAGATACACTCTCTT↑**AAGAAAATTA**↓AATGATCCTTGGGTGGCTTGTTAAACATGGTCC
AGTATGACATTGAAAGGACATTTAGTCAACTTCTTTCTTTACTTCCAAGTTTGGACATTTTTCTTAAGATTG
TGGATATTACAGTGTAGCTATGGCTGCCTTGAGATTATTGCTCACTTATATAGTGGCATTTTAAGCTGCTTA
ATCCCCTCAAATGTGCTGCCAA

Filled site:

ACTCAGGAAGCTGAGGCAGGAGAATCACTTGAACCTGAGAGGCGGAGGTTGCAGTGAGCCGAGATTGCACTG
CTGCACTCCAGCCTGGGTGAAATAGTGAGACTCTGTCTCAAAAAACAAACAAACAAACAAAACTAACTTTTG
GGATTATTGAGCAGAAATGTACGAGTTAACATGAAACTTCAGTGTCTAGGCTGTAATAAAATAGTAAACAGC
AAAGTCAAGTACAGATACACTCTCTTAAGAAAATTA**GGCCGGGCGCGGTGGCTCACGCCTGTAATCCCAGCA**
CTTTGGGAGGCCGAGGCGGGCGGATCACGAGGTCAGGAGATCGAGACCATCCTGGCTAACACGGTGAAACCC
CGTCTCTACTAAAAAAAAAAATACAAAAAATTAGCCGGGCGTGGTAGCGGGCGCCTGTAGTCCCAGCTACTC
GGGAGGCTGAGGCAGGGGAATGGCGTGAACCCGGGAGGCGGAGCTTGCAGTGAGCCGAGATCCCGCCACTGC
ACTCCA... [NEOcassette] [Atail] AAGAAAATTAAATGATCCTTGGGTGGCTTGTTAAACATGGTC
CAGTATGACATTGAAAGGACATTTAGTCAACTTCTTTCTTTACTTCCAAGTTTGGACATTTTTCTTAAGATT
GTGGATATTACAGTGTAGCTATGGCTGCCTTGAGATTATTGCTCACTTATATAGTGGCATTTTAAGCTGCTT
AATCCCCTCAAATGTGCTGCCAA

Alu at the 5'; LTR flanking the insertion site.

CLONE 235

[GG30]

Driver: ORF2
Plasmid: AlurescueA70Du $A_{17}CATTACA_{18}GA_{17}CACACA_{18}$ (T)
Chromosome: 12
5' position: 107,312,921
Strand: plus
DR: AGACATTTAAAAAGTC
ENDOsite: GTCT/AA

Empty site:

TGAATGACTAGAGTCTAAAAACAGCATGTGCTGCTACTGCCATCTAGCAGTCCAACTAGGGCTGCATGGATT
TAGCTTTCCTCAGGTATTTTGAAAGACATCAGGGGGCTATTATTCAAAACACAAAAACTTCATTGAATATGC
AAACATCCTACAGCCCCTAAGTGCTTTCTGAGTGGGAGAGAGTATACATATATAAACAAATCTT↑**AGACATT**
TAAAAAGTC↓TGTTCTGAGCCTGTAATGTTTGACTATTGTAATATTTGACTACTGCAGTCAAACATTATACC
ATCATTGCAATTCAGTGGTCTCAAGGGCCTTTGGTGATGTGCCCAGACTGCCCAGTACAGCAGATATTTGTT
GGGTACCCACTATGCATGCAGAGGACTGTGCAGGTGTGTGTGTGGTAGAAAGAGACAGGAATTTGG

Filled site:

TGAATGACTAGAGTCTAAAAACAGCATGTGCTGCTACTGCCATCTAGCAGTCCAACTAGGGCTGCATGGATT
TAGCTTTCCTCAGGTATTTTGAAAGACATCAGGGGGCTATTATTCAAAACACAAAAACTTCATTGAATATGC
AAACATCCTACAGCCCCTAAGTGCTTTCTGAGTGGGAGAGAGTATACATATATAAACAAATCTTAGACATTT
AAAAAGTC**GGCCGGGCGCGGTGGCTCACGCCTGTAATCCCAGCACTTTGGGAGGCCGAGGCGGGCGGATCAC**
GAGGTCAGGAGATCGAGACCATCCTGGCTAACACGGTGAAACCCCGTCTCTACTAAAAAAAAAAAAAAAAATA
CAAAAAATTAGCCGGGCGTGGTAGCGGGCGCCTGTAGTCCCAGCTACTCGGGAGGCTGAGGCAGGGGAATGG
CGTGAACCCGGGAGGCGGAGCTTGCAGTGAGCCGAGATCCCGCC...[NEOcassette][Atail]AGACA
TTTAAAAAGTCTGTTCTGAGCCTGTAATGTTTGACTATTGTAATATTTGACTACTGCAGTCAAACATTATAC
CATCATTGCAATTCAGTGGTCTCAAGGGCCTTTGGTGATGTGCCCAGACTGCCCAGTACAGCAGATATTTGT
TGGGTACCCACTATGCATGCAGAGGACTGTGCAGGTGTGTGTGTGGTAGAAAGAGACAGGAATTTGG

No repetitive sequences in the immediate proximity.

CLONE 236

[LZ7]

Driver: ORF2
Plasmid: AlurescueA70Du $A_{17}CATTACA_{18}GA_{17}CACACA_{18}$ (T)
Chromosome: 12
5’ position: 112,411,757
Strand: plus
DR: AAAAAATCCTACAGT
ENDOsite: TTTT/GT

Empty site:

CAGGAAATAATGAAGATCAAAGCGGAAATCAAGGCCAGAAGTGGTGGCTCCCACCTGTAATCCCAGCACTTT
GGGAGGCCGAGGCTGGAGATCACTTGAGTCCAGGAGTTTGAGACCAGCCTGGCCAACATATAAAATTAGCCA
GGTGTGGTGGCACACACCTGCAATCCCAGCTACTTGGGTGGCTGAGGCAGGAGAATCACTTGAACCCAGGAG
GTGGAGGTTGCAGATCGTGGCCTGCACTCCAAGCTGGGCAACAAAAGCGAGACTTTGTCAAAAAAACCCAAA
AAAACAAAAAAAC↑**AAAAAATCCTACAGT**↓ATATAAAAATAATACATCACAACTAAGTGGGGTTTATCTCAG
GCCTACAGAGTTGTTTCAACATTCAAAAGTAAATCAATTTAAATCATCCTATTAAAATACTAAAAAAATAAA
AACCAGTGATCATCTAAATAGGTACAGAAAATAGCATTTGGAAAAATATAACATCCATTCTCAATTTTAAAA
ACAGAGAAAAAAAA

Filled site:

CAGGAAATAATGAAGATCAAAGCGGAAATCAAGGCCAGAAGTGGTGGCTCCCACCTGTAATCCCAGCACTTT
GGGAGGCCGAGGCTGGAGATCACTTGAGTCCAGGAGTTTGAGACCAGCCTGGCCAACATATAAAATTAGCCA
GGTGTGGTGGCACACACCTGCAATCCCAGCTACTTGGGTGGCTGAGGCAGGAGAATCACTTGAACCCAGGAG
GTGGAGGTTGCAGATCGTGGCCTGCACTCCAAGCTGGGCAACAAAAGCGAGACTTTGTCAAAAAAACCCAAA
AAAACAAAAAAACAAAAAATCCTACAGT**GGCCGGGCGCGGTGGCTCACGCCTGTAATCCCAGCACTTTGGGA**
GGCCGAGGCGGGCGGATCACGAGGTCAGGAGATCGAGACCATCCTGGCTAACACGGTGAAACCCCGTCTCTA
CTAAAAAAAAATACAAAAAATTAGCCGGGCGTGGTAGCGGGCGCCTGTAGTCCCAGCTACTCGGGAGGCTGA
GGCAGGGGAATGGCGTGAACCCGGGAGGCGGAGCTTGCAGTGAGCCGAGATCCCGCCACTGCACTCCA… [NE
Ocassette] [Atail]AAAAAATCCTACAGTATATAAAAATAATACATCACAACTAAGTGGGGTTTATCT
CAGGCCTACAGAGTTGTTTCAACATTCAAAAGTAAATCAATTTAAATCATCCTATTAAAATACTAAAAAAAT
AAAAACCAGTGATCATCTAAATAGGTACAGAAAATAGCATTTGGAAAAATATAACATCCATTCTCAATTTTA
AAAACAGAGAAAAAAAA

Alu at the 5'; LINE-1 at the 3'.

CLONE 237

[GG18,GG19]

Driver: ORF2
Plasmid: AlurescueA70Du $A_{17}CATTACA_{18}GA_{17}CACACA_{18}$ (T)
Chromosome: 12
5' position: 112,787,674
Strand: plus
DR: AGAAAAGAAATAAAACA
ENDOsite: TTCT/AG

Empty site:

AACATCTAGTTTTGCTTCCCTCTTGTTATATGAATCTCAATCTCATGCTGAGAACAGATGGCAAAAAAAAAA
ATTGGCACAGAATGCAACTACGGCAGTACAAATATGGTGGTCAGCAGGAAATACATTATTCTTAATTTCAAC
CTTTTCCTGGAAACAGTGCCTGGAGTGTTATGGAGTAAAAATGGGTGAACAGGTGTAGGAGGATTTTATTTT
TGGCAAGGGCTGAAAGCAAAGGAGAAAAATTAGTTTGATTTAAGGAGCCTACTTAATTCT↑**AGAAAAGAAAT
AAAACA**↓GACTGGGAACACATTGAATAATTTTATATTTCTGTAAATGTTCATATTTTTTGGAAAGGCTATCA
ATAATATGATATTTTTCTTTTTCTTTTTCTTTTTTTTGAGACAGGGCCTCATTCTGTTGCCCAGGTTGGAGT
GCAGTGGCACAAACACAGCTCACTGCAGCCCCAAACTCCTGGACTCAAGTGATTTTCCTGCCT

Filled site:

AACATCTAGTTTTGCTTCCCTCTTGTTATATGAATCTCAATCTCATGCTGAGAACAGATGGCAAAAAAAAAA
ATTGGCACAGAATGCAACTACGGCAGTACAAATATGGTGGTCAGCAGGAAATACATTATTCTTAATTTCAAC
CTTTTCCTGGAAACAGTGCCTGGAGTGTTATGGAGTAAAAATGGGTGAACAGGTGTAGGAGGATTTTATTTT
TGGCAAGGGCTGAAAGCAAAGGAGAAAAATTAGTTTGATTTAAGGAGCCTACTTAATTCTAGAAAAGAAATA
AAACA**GGCCGGGCGCGGTGGCTCACGCCTGTAATCCCAGCACTTTGGGAGGCCGAGGCGGGCGGATCACGAG
GTCAGGAGATCGAGACCATCCTGGCTAACACGGTGAAACCCCGTCTCTACTAAAAAAAAATACAAAAAATTA
GCCGGGCGTGGTAGCGGGCGCCTGTAGTCCCAGCTACTCGGGAGGCTGAGGCAGGGGAATGGCGTGAACCCG
GGAGGCGGAGCTTGCAGTGAGCCGAGATCCCGCCACTGCA...[NEOcassette][Atail]**AGAAAAGAA
ATAAAACAGACTGGGAACACATTGAATAATTTTATATTTCTGTAAATGTTCATATTTTTTGGAAAGGCTATC
AATAATATGATATTTTTCTTTTTCTTTTTCTTTTTTTTGAGACAGGGCCTCATTCTGTTGCCCAGGTTGGAG
TGCAGTGGCACAAACACAGCTCACTGCAGCCCCAAACTCCTGGACTCAAGTGATTTTCCTGCCT

Alu at the 3'.

CLONE 238

[LZ2]

Driver: ORF2
Plasmid: AlurescueA70Du $A_{17}CATTACA_{18}GA_{17}CACACA_{18}$ (T)
Chromosome: 13
5' position: 77,685,020
Strand: minus
DR: AAGAAGTTATCCAGCTC
ENDOsite: TCTT/AG

Empty site:

ACAAGTTGAAAATAGAAAATTACTCATATATCACATTTTATTAAAAAATATTATTAAAGCATTTTCATGATT
TTGCACTCAATTTAGGAAGGATTAGGTCTGCTATATCTCCCACTTTGTTATTCTCTCAAACTTCTGTACCTG
TAGTATATGTCATTACTTAAGCTGAACAAAACGCTCATTTTTTGTAATCAGACTTCTCATCCTTATCATCAG
ATTGTCTTTCCTCCTTTGGTTTATTTAAGAAGTGTGCATCAGCAGCAGCCGTTATGTCTTATGATGGCATAA
TTTCAAGTGTTCTAGGGGCCT↑**AAGAAGTTATCCAGCTC**↓TCATTTTGAGATGAGAGAATTCTTGCTGACTT
GCCCAAAGTCATAGCTGGCAAATCTAGGACTTGAACATGAGAGTCTGTATTGGGGGAACCCGCCCCCAATAT
TTCAACGTAGGTTCTTTCTATTTTCCCTAAGCATTGGCCAGTCTGAGAAAAAAAGAGAAAGAGTACAAAGAG
GAATTTTACAGCTGGGCCTCTGGC

Filled site:

ACAAGTTGAAAATAGAAAATTACTCATATATCACATTTTATTAAAAAATATTATTAAAGCATTTTCATGATT
TTGCACTCAATTTAGGAAGGATTAGGTCTGCTATATCTCCCACTTTGTTATTCTCTCAAACTTCTGTACCTG
TAGTATATGTCATTACTTAAGCTGAACAAAACGCTCATTTTTTGTAATCAGACTTCTCATCCTTATCATCAG
ATTGTCTTTCCTCCTTTGGTTTATTTAAGAAGTGTGCATCAGCAGCAGCCGTTATGTCTTATGATGGCATAA
TTTCAAGTGTTCTAGGGGCCTAAGAAGTTATCCAGCTC**GGCCGGGCGCGGTGGCTCACGCCTGTAATCCCAG**
CACTTTGGGAGGCCGAGGCGGGCGGATCACGAGGTCAGGAGATCGAGACCATCCTGGCTAACACGGTGAAAC
CCCGTCTCTACTAAAAAAAAATACAAAAAATTAGCCGGGCGTGGTAGCGGGCGCCTGTAGTCCCAGCTACTC
GGGAGGCTGAGGCAGGGGAATGGCGTGAACCCGGGAGGCGGAGCTTGCAGTGAGCCGAGATCCCGCCACTGC
ACTCCAGC…[NEOcassette][Atail]AAGAAGTTATCCAGCTCTCATTTTGAGATGAGAGAATTCTT
GCTGACTTGCCCAAAGTCATAGCTGGCAAATCTAGGACTTGAACATGAGAGTCTGTATTGGGGGAACCCGCC
CCCAATATTTCAACGTAGGTTCTTTCTATTTTCCCTAAGCATTGGCCAGTCTGAGAAAAAAAGAGAAAGAGT
ACAAAGAGGAATTTTACAGCTGGGCCTCTGGC

MIR and LTR at the 3'.

CLONE 239

[LZ55]

Driver: ORF2
Plasmid: AlurescueA70Du $A_{17}CATTACA_{18}GA_{17}CACACA_{18}$ (T)
Chromosome: 13
5' position: 79,200,920
Strand: plus
DR: N/A
ENDOsite: N/A

Empty site:

GGTAGAAGAAGAGTAATCAAAAGTACAAGAGGAAAATGCTACCTCTGTCTAGGAAAATTAGGGAGGGCCTCA
CATATAAACCAACAACTGGGCTAGATCTCAAAGGACAGACAGGGATTCACCAGGAAGAATAAAAACAAGAGA
AGCTAATGTGGAGAACCAGGGAAAATACAGTATGATGAATGTGGGCCCAATGAGAAATTCAGCATAGCCAGA
GCAGAGCACAAGAAGCGAGGGGCTAGAGAAGAGGCAAGGATATTAAACTGAAGTTATATAGTAAAGACCCTT
TTATACTAGACCAAGAATATTGG↑↓GGCCAGGCGTGGTGGCTCACACCTGTAATCCCAGACTTTGGGAGGCC
AAGGCAGGCGGATCATGAGGTCAGGAGATCGAGACCATCCTGGCTAACACAGTGAAACCCCATCTCTACTAA
AAATACAAAAAATTAGCCGGGCATGGTGGCGGGCGCCTGTAGTCCCAGCTACTCAGGAGGCTGAGGCAGGAG
AATAGCATGGAACCCAGGGGGCGGAGCTTGCAGTGAACTGAGATGGTGCCACTGCACTCCAGCCTGGGCGAC
AGCACAGGAAAGAAAGGTAAGCAGTAGAAAGGCTGTTA

Filled site:

GGTAGAAGAAGAGTAATCAAAAGTACAAGAGGAAAATGCTACCTCTGTCTAGGAAAATTAGGGAGGGCCTCA
CATATAAACCAACAACTGGGCTAGATCTCAAAGGACAGACAGGGATTCACCAGGAAGAATAAAAACAAGAGA
AGCTAATGTGGAGAACCAGGGAAAATACAGTATGATGAATGTGGGCCCAATGAGAAATTCAGCATAGCCAGA
GCAGAGCACAAGAAGCGAGGGGCTAGAGAAGAGGCAAGGATATTAAACTGAAGTTATATAGTAAAGACCCTT
TTATACTAGACCAAGAATATTGG**GGCCAGGCGTGGTGGCTCACACCTGTAATCCCAGACTTTGGGAGGCCAA**
GGCAGGCGGATCATGAGGTCAGGAGATCGAGACCATCCTGGCTAACACAGTGAAACCCCATCTCTACTAAAA
ATACAAAAAATTAGCCGGGCATGGTGGCGGGCGCCTGTAGTCCCAGCTACTCAGGAGGCTGAGGCAGGAGAA
TAGCATGAACCCAGGGGGCGGAGCTTGCAGTGAACTGAGATGGTGCCACTGCACTCCAGCCTGGGCGACAGC
…**[NEOcassette][Atail and 3' unsequenced]**

Alu at the 3'.

Alu inserted by recombination into an existing Alu (highlighted in gray).

CLONE 240

[LZ65,LZ74]

Driver: ORF2
Plasmid: AlurescueA70Du $A_{17}CATTACA_{18}GA_{17}CACACA_{18}$ (T)
Chromosome: 13
5' position: 92,850,663
Strand: minus
DR: AAGAAAGAAGTATTATTAATT
ENDOsite: TCTT/AA

Empty site:

TATAATTTACTTGCAAAGATTGTACGAATGCCCACTCTGAATCAGAAGTGAAAATGTATCAGGTCAATAAGT
AATCTTCCCATTGGCTTCAAAGGCTAGACAGCTGGTGATTTCCTCTTGACATCAGTCTTGACGGGAGCAATG
TGTCTACATCCTGACTTTACTCACTGCCAGTCTGCCTACATTTAATCCACATCAGTCCACATTAATCCACAT
TCATGATGGAAGCAAGAATAATATTTGAGCTAATTTGGTCATTCAAATATCTGAACTATAAGTCATCAATTT
GGACTTTAGAATGCTGTGGGGAAATCATCATTCATT↑**AAGAAAGAAGTATTATTAATT**↓AATAAGGAGCATA
TCTATGATCTGTCAATGAGGTATCAACCAACCTATCAACAGAAGAAATTTTTATTGGACATCTCTTAGTAAC
AGGCTCCATCCTAGGTTAAAGGATATAGAGGAATGTAGCACCAGTAAGGATGCTGTTCTCATGAAGGTAGCA
TTTTAGTGTGGAGAGACATATCATATACAAATAGGTAATATAT

Filled site:

TATAATTTACTTGCAAAGATTGTACGAATGCCCACTCTGAATCAGAAGTGAAAATGTATCAGGTCAATAAGT
AATCTTCCCATTGGCTTCAAAGGCTAGACAGCTGGTGATTTCCTCTTGACATCAGTCTTGACGGGAGCAATG
TGTCTACATCCTGACTTTACTCACTGCCAGTCTGCCTACATTTAATCCACATCAGTCCACATTAATCCACAT
TCATGATGGAAGCAAGAATAATATTTGAGCTAATTTGGTCATTCAAATATCTGAACTATAAGTCATCAATTT
GGACTTTAGAATGCTGTGGGGAAATCATCATTCATTAAGAAAGAAGTATTATTAATT**GGCCGGGCGCGGTGG**
CTCACGCCTGTAATCCCAGCACTTTGGGAGGCCGAGGCGGGCGGATCACGAGGTCAGGAGATCGAGACCATC
CTGGCTAACACGGTGAAACCCCGTCTCTACTAAAAAAAAAATACAAAAAATTAGCCGGGCGTGGTAGCGGGC
GCCTGTAGTCCCAGCTACTCGGGAGGCTGAGGCAGGGGAATGGCGTGAACCCGGGAGGCGGAGCTTGCAGTG
AGCCGAGATCCCGCCACTGCACTCCA…[NEOcassette][Atail]AAGAAAGAAGTATTATTAATTAA
TAAGGAGCATATCTATGATCTGTCAATGAGGTATCAACCAACCTATCAACAGAAGAAATTTTTATTGGACAT
CTCTTAGTAACAGGCTCCATCCTAGGTTAAAGGATATAGAGGAATGTAGCACCAGTAAGGATGCTGTTCTCA
TGAAGGTAGCATTTTAGTGTGGAGAGACATATCATATACAAATAGGTAATATAT

L2 at the 3'.

CLONE 241

[LZ80]

Driver: ORF2
Plasmid: AlurescueA70Du $A_{17}CATTACA_{18}GA_{17}CACACA_{18}$ (T)
Chromosome: 14
5' position: 56,285,222
Strand: minus
DR: AATAGTTACCTAATT
ENDOsite: TATT/AA

Empty site:

TCTGAGCATAAGCTCCCATAAAGCCTTGTTTAGGAAAACTCTTTTGGCCTCATGTCAATCTCTATTACATTG
AGAGCCCAAGAACCCATGGTTGGTAACAACAATGTAAAGAAAAACTAATACATTTGACTTGAGACAGTCTTT
AGTTTGTGGCATAGAATGCCATTTCCTTCTCTAAATCTTCATTCCTGCTAGTTGGTTTTACTAGGATT↑**AAT**
AGTTACCTAATT↓ATATTATAAATGGTATTTATTGCATTTCAACTTTTTTTTTTTTTTTTGAGACAGGGTCT
CACTCCATTGCCCAGGGTGAAGGGCAGTGGCATGATCATAGCTTACTGCAGACTCGAACTCCCACACTCAAG
CAATCCCCTCACCTCAGCCTCTCAAGTAGGTGGGACCACACGCATGTGCTACCACACCCAGATAATTTTTGT
AGTTTTTGTAGAGACAGGATCTCACTGTGTTGCCCAGGCTGGTCTCAAA

Filled site:

TCTGAGCATAAGCTCCCATAAAGCCTTGTTTAGGAAAACTCTTTTGGCCTCATGTCAATCTCTATTACATTG
AGAGCCCAAGAACCCATGGTTGGTAACAACAATGTAAAGAAAAACTAATACATTTGACTTGAGACAGTCTTT
AGTTTGTGGCATAGAATGCCATTTCCTTCTCTAAATCTTCATTCCTGCTAGTTGGTTTTACTAGGATTAATA
GTTACCTAATT**GGCCGGGCGCGGTGGCTCACGCCTGTAATCCCAGCACTTTGGGAGGCCGAGGCGGGCGGAT**
CACGAGGTCAGGAGATCGAGACCATCCTGGCTAACACGGTGAAACCCCGTCTCTACTAAAAAAAAATACAAA
AAATTAGCCGGGCGTGGTAGCGGGCGCCTGTAGTCCCAGCTACTCGGGAGGCTGAGGCAGGGGAATGGCGTG
AACCCGGGAGGCGGAGCTTGCAGTGAGCCGAGATCCCGCC...[NEOcassette][Atail]AATAGTTAC
CTAATTATATTATAAATGGTATTTATTGCATTTCAACTTTTTTTTTTTTTTTTGAGACAGGGTCTCACTCCA
TTGCCCAGGGTGAAGGGCAGTGGCATGATCATAGCTTACTGCAGACTCGAACTCCCACACTCAAGCAATCCC
CTCACCTCAGCCTCTCAAGTAGGTGGGACCACACGCATGTGCTACCACACCCAGATAATTTTTGTAGTTTTT
GTAGAGACAGGATCTCACTGTGTTGCCCAGGCTGGTCTCAAA

LTR at the 5'; Alu at the 3'; LINE-1 flanking the insertion site.

CLONE 242
[LZ33]

Driver: ORF2
Plasmid: AlurescueA70Du $A_{17}CATTACA_{18}GA_{17}CACACA_{18}$ (T)
Chromosome: 14
5' position: 65,094,075
Strand: plus
DR: GAGAATTAAATGAGCTA
ENDOsite: TCTC/AC

Empty site:

TAAGGGGAAAAAAAGCATTGCAGGAGTTGGGCAGAATCAGAGAGGTTGTTTAGAGAAGTGGATAAAAGCATG
GGTCCTGGCCTCAGGCAGATGAAGTTTTAAATATTTATCTAAGCCTAAATTTTCTTATCTGTAATGAAGGAT
AATCATCATTTCTGTTTATTGTAAAGGTTGTTGT↑**GAGAATTAAATGAGCTA**↓ATGCATGAGGCTCACTTTG
CACACGGCATGGCTTGCACTAAACACAGTAAACACAAAGGGTCCTGATATGGTTTGGCTCTGTTTCTCCACC
CAAATCTCATCTTGAATTGTAATCCTCACATGTCGAGGGAGGGACCTGTAATCCCCATGTGTCAAGGGAGGA
TGATGATTGGATCTTGGGGGTGGTTCCCCCATGCTGT

Filled site:

TAAGGGGAAAAAAAGCATTGCAGGAGTTGGGCAGAATCAGAGAGGTTGTTTAGAGAAGTGGATAAAAGCATG
GGTCCTGGCCTCAGGCAGATGAAGTTTTAAATATTTATCTAAGCCTAAATTTTCTTATCTGTAATGAAGGAT
AATCATCATTTCTGTTTATTGTAAAGGTTGTTGTGAGAATTAAATGAGCTA**GGCCGGGCGCGGTGGCTCACG**
CCTGTAATCCCAGCACTTTGGGAGGCCGAGGCGGGCGGATCACGAGGTCAGGAGATCGAGACCATCCTGGCT
AACACGGTGAAACCCCGTCTCTACTAAAAAAAAATACAAAAAATTAGCCGGGCGTGGTAGCGGGCGCCTGTA
GTCCCAGCTACTCGGGAGGCTGAGGCAGGGGAATGGCGTGAACCCGGGAGGCGGAGCTTGCAGTGAGCCGAG
ATCCCGCCACTGCACTCC...**[NEOcassette][Atail]**GAGAATTAAATGAGCTAATGCATGAGGCTCA
CTTTGCACACGGCATGGCTTGCACTAAACACAGTAAACACAAAGGGTCCTGATATGGTTTGGCTCTGTTTCT
CCACCCAAATCTCATCTTGAATTGTAATCCTCACATGTCGAGGGAGGGACCTGTAATCCCCATGTGTCAAGG
GAGGATGATGATTGGATCTTGGGGGTGGTTCCCCCATGCTGT

MIR at the 5'; LTR at the 3'.

CLONE 243
[LZ4]

Driver: ORF2
Plasmid: AlurescueA70Du $A_{17}CATTACA_{18}GA_{17}CACACA_{18}$ (T)
Chromosome: 14
5’ position: 91,245,526
Strand: plus
DR: N/A
ENDOsite: N/A

Empty site:

GACAGGCTGGACCACCTGTAGCCCCCTCATCTCAGCCAGAAAGTGCTGGCAGCACTCAATGGCCAGCTCATG
ACTGTGCTGGAGGGACGCGCCACCCAGGGCCCTGGTCTCTGCCAGCCACAGTCAGTCACGGAACGTGGTTTG
AACAGCAGATGGCCAGAAGGACAGGAGCCACTCCAGATAGTCCACTTCTTGCCATGGCCTTAAGAAGTCCCA
TTCA↑↓AAGTGTCCCTCTTCCTCTAGGAAGCCTTCCTGGCCCTGCTGGCCTGAAGTGACCTCTCCGTATTAC
CCATCCCTAGCTTTTACTATTTGCTGCCATGTTTGGGGTTGAGATTTTATTTTTTATTTTTTTAATTTTTTT
GAGATGGAGTCTCGCTCTTGTCGCACAGGCTGGAGTGCAATGGCGCGATCTCAGCTCACTGC

Filled site:

GACAGGCTGGACCACCTGTAGCCCCCTCATCTCAGCCAGAAAGTGCTGGCAGCACTCAATGGCCAGCTCATG
ACTGTGCTGGAGGGACGCGCCACCCAGGGCCCTGGTCTCTGCCAGCCACAGTCAGTCACGGAACGTGGTTTG
AACAGCAGATGGCCAGAAGGACAGGAGCCACTCCAGATAGTCCACTTCTTGCCATGGCCTTAAGAAGTCCCA
TTCA**GGCCGGGCGCGGTGGCTCACGCCTGTAATCCCAGCACTTTGGGAGGCCGAGGCGGGCGGATCACGAGG
TCAGGAGATCGAGACCATCCTGGCTAACACGGTGAAACCCCGTCTCTACTAAAAAAAAAAATACAAAAAATT
AGCCGGGCGTGGTAGCGGGCGCCTGTAGTCCCAGCTACTCGGGAGGCTGAGGCAGGGGAATGGCGTGAACCC
GGGAGGCGGAGCTTGCAGTGAGCCGAGATCCCGCCACTGCACTCCAG…[NEOcassette][Atail
and 3' unsequenced]**

Alu at the 3'.

CLONE 244

[LZ1]

Driver: ORF2
Plasmid: AlurescueA70Du $A_{17}CATTACA_{18}GA_{17}CACACA_{18}$ (T)
Chromosome: 14
5' position: 92,352,545
Strand: plus
DR: TGAAAGCACA
ENDOsite: TTCA/AA

Empty site:

CACGGAGGGAACTGAAATCTAGGAAGAAAGTGGCTTTTCATGCCTGAGGGTATTTTATTTCTGTTTCTGGGC
CTTGTATTTAATCAGCCCAAGGCTCTCTGGAACTTCCTGCTTGTGGCTCTGACTGTCCTGGCCCTAGGGAGA
GGTGTTCATCTGTTTCATGGGACGCTGTCCCCAGAACACTTCAGTTGTCAGTGAGACTATCACACAAGTTCC
ATTCATGACCTGGGTCAGTTCCTGTTACAATAAAACCCGGATTAGGAAACTTTCCATTCTGCAGGACAGAAA
GATTTTTCACGCCCTAGTGTATGGCCCACTTATTT↑**TGAAAGCACA**↓GTGAAGCATTATTAATTTAGACTTT
GCAAATTCACAACTGCTGATGATGCCAGAAGTGGCTGAAATGGAGTGTACTTTAATCTAACCTTTACAAAAA
ACAAGGCAAGAAAGGAAAAGAGAGAGAAAGAAAGAAAAGGAGTTGTGTGTGGCCCTATGTGGGATGCTACAG
AGATAAATGTGAGTAAGAGGTGGTCCCTATT

Filled site:

CACGGAGGGAACTGAAATCTAGGAAGAAAGTGGCTTTTCATGCCTGAGGGTATTTTATTTCTGTTTCTGGGC
CTTGTATTTAATCAGCCCAAGGCTCTCTGGAACTTCCTGCTTGTGGCTCTGACTGTCCTGGCCCTAGGGAGA
GGTGTTCATCTGTTTCATGGGACGCTGTCCCCAGAACACTTCAGTTGTCAGTGAGACTATCACACAAGTTCC
ATTCATGACCTGGGTCAGTTCCTGTTACAATAAAACCCGGATTAGGAAACTTTCCATTCTGCAGGACAGAAA
GATTTTTCACGCCCTAGTGTATGGCCCACTTATTTTGAAAGCACA**GGCCGGGCGCGGTGGCTCACGCCTGTA**
ATCCCAGCACTTTGGGAGGCCGAGGCGGGCGGATCACGAGGTCAGGAGATCGAGACCATCCTGGCTAACACG
GTGAAACCCCGTCTCTACTAAAAAAAAATACAAAAAATTAGCCGGGCGTGGTAGCGGGCGCCTGTAGTCCCA
GCTACTCGGGAGGCTGAGGCAGGGGAATGGCGTGAACCCGGGAGGCGGAGCTTGCAGTGAGCCGAGATCCCG
CCACTGCA…[NEOcassette][Atail]TGAAAGCACAGTGAAGCATTATTAATTTAGACTTTGCAAAT
TCACAACTGCTGATGATGCCAGAAGTGGCTGAAATGGAGTGTACTTTAATCTAACCTTTACAAAAAACAAGG
CAAGAAAGGAAAAGAGAGAGAAAGAAAGAAAAGGAGTTGTGTGTGGCCCTATGTGGGATGCTACAGAGATAA
ATGTGAGTAAGAGGTGGTCCCTATT

No repetitive sequences in the immediate proximity.

CLONE 245

[GG11,25,41]

Driver: ORF2
Plasmid: AlurescueA70Du $A_{17}CATTACA_{18}GA_{17}CACACA_{18}$ (T)
Chromosome: 15
5’ position: 37,024,052
Strand: minus
DR: AAGAAATAATTGA
ENDOsite: TCTT/AT

Empty site:

CAAACTTCTCAAATTTATTTTTTGGCTATAGAGGGGCAAAGGAAGTCCTGGGACATTTCTGCCTACTTCATG
TTCTGAAAGATCTCCATGAAGCCCTTCAGCAAGTCTACCTGTCACATTAAGGAGTCTAAAACTGTGATAATC
ATTCATAAAATTTTGTGTTTTACAATACTACTGTGACGTAACGAAGTTTGTAAAATAGTTTTTATTTAATAG
CATTAAGATATTTATGCATAAACTAAATGACATCAAATAAGCCAATAAATGTTTAGAAATCCCAAGTTTATA
AATATCTTCCTTAGAATGTAT↑**AAGAAATAATTGA**↓GAATTGTTTTTTAATAATCCTGGGAAAGAAATACAA
TTTGGCATGTCTCTTAACTTTGGCTTTTGCCCCCATTTTTAGGGATCATTTAGCCAGACAGGCAGGTTTTCA
TTAAATTTAATTTGTGTGGAGCTTAAATATTAAGTTGAGTTATTAGGATGGTAAACCAATTGATGGCTCTGT
CTTTTAACATCTTGTGGTGC

Filled site:

CAAACTTCTCAAATTTATTTTTTGGCTATAGAGGGGCAAAGGAAGTCCTGGGACATTTCTGCCTACTTCATG
TTCTGAAAGATCTCCATGAAGCCCTTCAGCAAGTCTACCTGTCACATTAAGGAGTCTAAAACTGTGATAATC
ATTCATAAAATTTTGTGTTTTACAATACTACTGTGACGTAACGAAGTTTGTAAAATAGTTTTTATTTAATAG
CATTAAGATATTTATGCATAAACTAAATGACATCAAATAAGCCAATAAATGTTTAGAAATCCCAAGTTTATA
AATATCTTCCTTAGAATGTATAAGAAATAATTGA**GGCCGGGCGCGGTGGCTCACGCCTGTAATCCCAGCACT**
TTGGGAGGCCGAGGCGGGCGGATCACGAGGTCAGGAGATCGAGACCATCCTGGCTAACACGGTGAAACCCCG
TCTCTACTAAAAAAAAAATACAAAAAATTAGCCGGGCGTGGTAGCGGGCGCCTGTAGTCCCAGCTACTCGGG
AGGCTGAGGCAGGGGAATGGCGTGAACCCGGGAGGCGGAGCTTGCAGTGAGCCGAGATCCCGCCAC…[NEOc
assette][Atail]AAGAAATAATTGAGAATTGTTTTTTAATAATCCTGGGAAAGAAATACAATTTGGCA
TGTCTCTTAACTTTGGCTTTTGCCCCCATTTTTAGGGATCATTTAGCCAGACAGGCAGGTTTTCATTAAATT
TAATTTGTGTGGAGCTTAAATATTAAGTTGAGTTATTAGGATGGTAAACCAATTGATGGCTCTGTCTTTTAA
CATCTTGTGGTGC

No repetitive sequences in the immediate proximity.

CLONE 246

[GG22]

Driver: ORF2
Plasmid: AlurescueA70Du $A_{17}CATTACA_{18}GA_{17}CACACA_{18}$ (T)
Chromosome: 15
5' position: 48,974,167
Strand: plus
DR: AAAAAATTCTACTTTA
ENDOsite: TTTT/AG

Empty site:

GTTCTGTGGGTTCCAGTTAAGTCAGTTTCACAAAGGAGATGGCAAAGGAGAAAGAGGCCCAGAAATGGGCAA
AGGAACCCCAAGCTGAGGGCAATGTGGATCCTCCATGTCCCACAAACAGAGCAGATGCTGTGTTCCTGACCT
GTTTGGAACTGAGCACTGGATGTTAATGAAAAAGTTAAGTTAAAACAGGCAATGGCATGCATCAGTTCT↑**AA
AAAATTCTACTTTA**↓ACCAAAAATATATAATGTAAATTTATTTACCATGATTTTAACTGCTTTATATTCGTT
GTATTTGTTCTTTTTTCCCAAATTTTTTTGCTTTCTCTGGTATTAAGAAACATTTTATGTAATTTCATTTTA
TCTTCTCTTAAATATCAACTATACTTCTTTTTTAAAAAAATTAAATCGTTGACCTAGAGTTTGCAATACTC

Filled site:

GTTCTGTGGGTTCCAGTTAAGTCAGTTTCACAAAGGAGATGGCAAAGGAGAAAGAGGCCCAGAAATGGGCAA
AGGAACCCCAAGCTGAGGGCAATGTGGATCCTCCATGTCCCACAAACAGAGCAGATGCTGTGTTCCTGACCT
GTTTGGAACTGAGCACTGGATGTTAATGAAAAAGTTAAGTTAAAACAGGCAATGGCATGCATCAGTTCTAAA
AAATTCTACTTTA**GGCCGGGCGCGGTGGCTCACGCCTGTAATCCCAGCACTTTGGGAGGCCGAGGCGGGCGG
ATCACGAGGTCAGGAGATCGAGACCATCCTGGCTAACACGGTGAAACCCCGTCTCTACTAAAAAAAAATACA
AAAAATTAGCCGGGCGTGGTAGCGGGCGCCTGTAGTCCCAGCTACTCGGGAGGCTGAGGCAGGGGAATGGCG
TGAACCCGGGAGGCGGAGCTTGCAGTGAGCCGAGATCCCGCCACTGCACT…[NEOcassette][Atail
]**AAAAAATTCTACTTTAACCAAAAATATATAATGTAAATTTATTTACCATGATTTTAACTGCTTTATATTCG
TTGTATTTGTTCTTTTTTCCCAAATTTTTTTGCTTTCTCTGGTATTAAGAAACATTTTATGTAATTTCATTT
TATCTTCTCTTAAATATCAACTATACTTCTTTTTTAAAAAAATTAAATCGTTGACCTAGAGTTTGCAATACT
C

LINE-1 at the 5'.

CLONE 247

[LZ18]

Driver: ORF2
Plasmid: AlurescueA70Du $A_{17}CATTACA_{18}GA_{17}CACACA_{18}$ (T)
Chromosome: 15
5' position: 59,724,193
Strand: minus
DR: AAAGAGATAAATAGGT
ENDOsite: CTTT/AA

Empty site:

TTACACCTTCACCTCAGGTCTGCACTAGCAAAGGAGTGAAGATTCGTGTACTTACCCAGATAAATCCAATCA
AATAATTGTTAGTTTAAAATACACAGAGCCAAACATGTTTTGGGATTTGAGCATCTTCTTAATTAATTAAAC
TGAAACGAGCGCTTTATGAGTACATGCCTGCCAGGTAAAGGCATATAGAGTGATGCAGTGTTTAAAAATTGT
AATTAATAGACAATACATAGCCTGTACTTAAAGTAAAATAAAATTAAAGAAAAAAGAAAAATAACAAAGTAA
GATAAATTAATATTATGCAGCTGTTTTT↑**AAAGAGATAAATAGGT**↓ATAATCGTCTTTGTTTTCCCTCCCAT
ATTTGAATACACTTAAATCATTTTCATGCACACACACAGGTTTCCCAAGGCAATGTGAAAGGGTTGCAGGAT
TCTCTACCAGGCACTGGAGCCCCACATAGTCACGTAGTAGGTTTTCATCACTGTAGACTCAGGAAACAGCTT
TTTCTTCCCCTGGGAAACATAAATAATAAA

Filled site:

TTACACCTTCACCTCAGGTCTGCACTAGCAAAGGAGTGAAGATTCGTGTACTTACCCAGATAAATCCAATCA
AATAATTGTTAGTTTAAAATACACAGAGCCAAACATGTTTTGGGATTTGAGCATCTTCTTAATTAATTAAAC
TGAAACGAGCGCTTTATGAGTACATGCCTGCCAGGTAAAGGCATATAGAGTGATGCAGTGTTTAAAAATTGT
AATTAATAGACAATACATAGCCTGTACTTAAAGTAAAATAAAATTAAAGAAAAAAGAAAAATAACAAAGTAA
GATAAATTAATATTATGCAGCTGTTTTTAAAGAGATAAATAGGT**GGCCGGGCGCGGTGGCTCACGCCTGTAA**
TCCCAGCACTTTGGGAGGCCGAGGCGGGCGGATCACGAGGTCAGGAGATCGAGACCATCCTGGCTAACACGG
TGAAACCCCGTCTCTACTAAAAAAAAATACAAAAAATTAGCCGGGCGTGGTAGCGGGCGCCTGTAGTCCCAG
CTACTCGGGAGGCTGAGGCAGGGGAATGGCGTGAACCCGGGAGGCGGAGCTTGCAGTGAGCCGAGATCCCGC
CACTGCACTCC... [NEOcassette] [Atail] AAAGAGATAAATAGGTATAATCGTCTTTGTTTTCCCTC
CCATATTTGAATACACTTAAATCATTTTCATGCACACACACAGGTTTCCCAAGGCAATGTGAAAGGGTTGCA
GGATTCTCTACCAGGCACTGGAGCCCCACATAGTCACGTAGTAGGTTTTCATCACTGTAGACTCAGGAAACA
GCTTTTTCTTCCCCTGGGAAACATAAATAATAAA

No repetitive sequences in the immediate proximity.

CLONE 248

[LZ28]

Driver: ORF2
Plasmid: AlurescueA70Du $A_{17}CATTACA_{18}GA_{17}CACACA_{18}$ (T)
Chromosome: 15
5' position: 69,480,707
Strand: minus
DR: AAGAAAACTA
ENDOsite: TCTT/AT

Empty site:

ATAACCTGAATAACCTTACATATATTAAAGGAATTGAAATTACAGTTAAAAACCACCCCACAAAGAAAACTT
CCAGGTCCAGCTGACTTCACTGGTCAATTCTAACAAACATTTAAGGAAGAAATAATACTAATTCTAAAAGGC
TTATCCAGAAAATTGAAGAAGAGAGAACACTTCTCAAGTCATTCTAAAAGGGCACCATGACCCTGTATCAAA
ACCAAACTAAGATATTAT↑**AAGAAAACTA**↓TAGACCAATTTTCCTATGAATATAGATGTAAAAATACTTAAT
ATGACATAAATCAAATCCAACAATATGTAAAAAGTACAATACCTCATGACCAAGTTGTGTTTTTTTGTTTTG
TTTTGTTTTTTGTTTTTGTTTTGAGACGGAGTCTCACTCTGTCGCCCAAGCTGGAGTGCAGTGGCAATCTTG
GCAACCTCCACCTC

Filled site:

ATAACCTGAATAACCTTACATATATTAAAGGAATTGAAATTACAGTTAAAAACCACCCCACAAAGAAAACTT
CCAGGTCCAGCTGACTTCACTGGTCAATTCTAACAAACATTTAAGGAAGAAATAATACTAATTCTAAAAGGC
TTATCCAGAAAATTGAAGAAGAGAGAACACTTCTCAAGTCATTCTAAAAGGGCACCATGACCCTGTATCAAA
ACCAAACTAAGATATTATAAGAAAACTA**GGCCGGGCGCGGTGGCTCACGCCTGTAATCCCAGCACTTTGGGA
GGCCGAGGCGGGCGGATCACGAGGTCAGGAGATCGAGACCATCCTGGCTAACACGGTGAAACCCCGTCTCTA
CTAAAAAAAAATACAAAAAATTAGCCGGGCGTGGTAGCGGGCGCCTGTAGTCCCAGCTACTCGGGAGGCTGA
GGCAGGGGAATGGCGTGAACCCGGGAGGCGGAGCTTGCAGTGAGCCGAGATCCCGCC…[NEOcassette]
[Atail]**AAGAAAACTATAGACCAATTTTCCTATGAATATAGATGTAAAAATACTTAATATGACATAAATCA
AATCCAACAATATGTAAAAAGTACAATACCTCATGACCAAGTTGTGTTTTTTTGTTTTGTTTTGTTTTTTGT
TTTTGTTTTGAGACGGAGTCTCACTCTGTCGCCCAAGCTGGAGTGCAGTGGCAATCTTGGCAACCTCCACCT
C

LINE-1 flanking the insertion site; Alu at the 3'.

CLONE 249

[LZ83]

Driver: ORF2
Plasmid: AlurescueA70Du $A_{17}CATTACA_{18}GA_{17}CACACA_{18}$ (T)
Chromosome: 16
5' position: 30,514,516
Strand: minus
DR: GAAACTGGGTG
ENDOsite: TTTC/AA

Empty site:

GCACAGTTGTCCTAAGAGCTTGGAGTGACAGTCAAGATTTGAACCCAAATCTGTCCAAATCCAAAGTCCAGG
ATCTTTCCACCATGTCACACTGCTTTCTTTTCTGGCAAATTTATGTTTAAAATGTCTAAATCTTATAAGAGG
TGGAATGGGAGTAGGCAGAGGGGCACTGAGTAGAGGTTTGAATGAAATAGAGGGTGAGTCGATAATTGTT↑**G**
AAACTGGGTG↓ATGAGGACCTGGGGATTCATTAAACTATTTTTTTTCTAGTTTTGTGTGATTCATATTCTCA
TAATAAAAAGTTTAAAACAGGCTGGGTGCAGTGGCTTACACCTGTAATCCCAGCACTTTGGGATGCCGAGGT
GGGTGGATCACCTGAGGTCAGGAGTTTGAGACCAGCCTGGCCAACATGGTGAAAACCCATCTCTATC

Filled site:

GCACAGTTGTCCTAAGAGCTTGGAGTGACAGTCAAGATTTGAACCCAAATCTGTCCAAATCCAAAGTCCAGG
ATCTTTCCACCATGTCACACTGCTTTCTTTTCTGGCAAATTTATGTTTAAAATGTCTAAATCTTATAAGAGG
TGGAATGGGAGTAGGCAGAGGGGCACTGAGTAGAGGTTTGAATGAAATAGAGGGTGAGTCGATAATTGTTGA
AACTGGGTG**GGCCGGGCGCGGTGGCTCACGCCTGTAATCCCAGCACTTTGGGAGGCCGAGGCGGGCGGATCA**
CGAGGTCAGGAGATCGAGACCATCCTGGCTAACACGGTGAAACCCCGTCTCTACTAAAAAAAAATACAAAAA
ATTAGCCGGGCGTGGTAGCGGGCGCCTGTAGTCCCAGCTACTCGGGAGGCTGAGGCAGGGGAATGGCGTGAA
CCCGGGAGGCGGAGCTTGCAGTGAGCCGAGATCCCGCCACTGCAC…**[NEOcassette][Atail]**GAAA
CTGGGTGATGAGGACCTGGGGATTCATTAAACTATTTTTTTTCTAGTTTTGTGTGATTCATATTCTCATAAT
AAAAAGTTTAAAACAGGCTGGGTGCAGTGGCTTACACCTGTAATCCCAGCACTTTGGGATGCCGAGGTGGGT
GGATCACCTGAGGTCAGGAGTTTGAGACCAGCCTGGCCAACATGGTGAAAACCCATCTCTATC

MIR at the 5'; LINE-1 flanking the insertion site; Alu at the 3'.

CLONE 250
[TZ13]

Driver: ORF2
Plasmid: AlurescueA70Du $A_{17}CATTACA_{18}GA_{17}CACACA_{18}$ (T)
Chromosome: 16
5' position: 69,597,400
Strand: plus
DR: N/A
ENDOsite: N/A

Empty site:

AGTTCAAGACTAGCCTGGGTAATATAGTAAGACCATGTCTCTACAAAAAAATAAAAAATAAAAATAAATAAT
AAAATGAATTAGCCGAGTGTGGTGGCATGTGCCTGTAGTCCCAGCTACTTGGGAGGCTGAGGTGAAGGATCA
CTTTGAGCCCGGGAGGAGTTTCAGTCTGCAGCTGCACTGAGCTATGAAGCTGCCACTACACTCCAGCCTGGG
CAACAGAACAAGACCCTGTCTCAAAAAAATAAACAAACAAAAAAACCCGAAAGAATAGAAAAGAAAAAAAAG
AAAAGAAAAGAATATAGGCCAGGTGCCGTGGCTCAT↑GCCTGTAATCCCAGCACTTTGGGAGGCC↓AAGGCG
GGTGGATCACCTGAGGTCAGGAGTTCGACACCAGCCTGGCCAACATGGTGAAACCCCATCTCTACTAAAAAT
ACAAAAAATTAGCCGGGCATGGTGGTGGGCGCCTGTAACCCCAGCTACTCAGGAGGCTGAAGCAGAAGAATC
ACTTGAACCAGGGAGGCAGAGGTTGCAGTGAGCCGAGATTGCACTGCTGCACTCCAGCCTGGACAACAAGAG
TGAAATTCCATCTTAAAAAAACAAACAAAAAAGAGAATATATCTCTGTAA

Filled site:

AGTTCAAGACTAGCCTGGGTAATATAGTAAGACCATGTCTCTACAAAAAAATAAAAAATAAAAATAAATAAT
AAAATGAATTAGCCGAGTGTGGTGGCATGTGCCTGTAGTCCCAGCTACTTGGGAGGCTGAGGTGAAGGATCA
CTTTGAGCCCGGGAGGAGTTTCAGTCTGCAGCTGCACTGAGCTATGAAGCTGCCACTACACTCCAGCCTGGG
CAACAGAACAAGACCCTGTCTCAAAAAAATAAACAAACAAAAAAACCCGAAAGAATAGAAAAGAAAAAAAAG
AAAAGAAAAGAATATAGGCCAGGTGCCGTGGCTCAT**GCCTGTAATCCCAGCACTTTGGGAGGCCGAGGCGGG**
CGGATCACGAGGTCAGGAGATCGAGACCATCCTGGCTAACACGGTGAAACCCCGTCTCTACTAAAAAAAAAA
AAATACAAAAAATTAGCCGGGCGTGGTAGCGGGCGCCTGTAGTCCCAGCTACTCGGGAGGCTGAGGCAGGGG
AATGGCGTGAACCCGGGAGGCGGAGCTTGCAGTGAGCCGAGATCCCGCC… [NEOcassette] [Atail and 3' unsequenced]

Inserted by recombination with a pre-existing truncated Alu: missing between 20-48 bp of 5' Alu sequence.

Sequence is shared between the Alu rescue vector and the genomic pre-insertion site.

Alu in the 5' and 3'.

CLONE 251

[LZ4]

Driver: ORF2
Plasmid: AlurescueA70Du $A_{17}CATTACA_{18}GA_{17}CACACA_{18}$ (T)
Chromosome: 16
5' position: 89,575,313
Strand: plus
DR: TAAAGTGGAATCCA
ENDOsite: TTTA/AA

Empty site:

GAGCCCGGGAGGCCCCTNCGGCGGGGGAGCCTGTATCTTGTGGGCCCGCGGATCCCCCAGCTGTGGACCTCG
GCGCGGAGCGACTGTTGGGGCCCTGGATCGTGGGCGCTGGGCGGGCCGGGAAGAGGCAGGGCTGGGATCCGC
GCAGTCCTCGGCGTGGACTTTCCCAACCCGTCTGTTGTGTGTGGATGTTCTCCGCCCGTCTTCCTCGCCTTT
↑**TAAAGTGGAATCCA**↓GTAACGGTTTCCGGCGTAGCACTAATTTACAGTCCACGCCTGTGGATCGATTCCGT
CACCAGGAATTCCAGCGCCTTCCAGTTAGGGTGGTTATTGCCGAGTAGGGGGCCCAGAACGTTTCTGTGTCC
GCAAAATACCCTGTAGCTGCATTTTGCTGTTGTCATGTAGCTCGGCACGTTATTGATACAGTCATTGAGCAC

Filled site:

GAGCCCGGGAGGCCCCTNCGGCGGGGGAGCCTGTATCTTGTGGGCCCGCGGATCCCCCAGCTGTGGACCTCG
GCGCGGAGCGACTGTTGGGGCCCTGGATCGTGGGCGCTGGGCGGGCCGGGAAGAGGCAGGGCTGGGATCCGC
GCAGTCCTCGGCGTGGACTTTCCCAACCCGTCTGTTGTGTGTGGATGTTCTCCGCCCGTCTTCCTCGCCTTT
TAAAGTGGAATCCA**GGCCGGGCGCGGTGGCTCACGCCTGTAATCCCAGCACTTTGGGAGGCCGAGGCGGGCG
GATCACGAGGTCAGGAGATCGAGACCA*TCCT*GGCTAACACGGTGAAACCCCGTCTCTACTAAAAAAAATACA
AAAAATTAGCCGGGCGTGGTAGCGGGCGCCTGTAGTCCCAGCTACTCGGGAGGCTGAGGCAGGGGAATGGCG
TGAACCCGGGAGGCGGAGCTTGCAGTGAGCCGAGATCCCGCCACTGCACTCC…[NEOcassette][Ata
il]**TAAAGTGGAATCCAGTAACGGTTTCCGGCGTAGCACTAATTTACAGTCCACGCCTGTGGATCGATTCCG
TCACCAGGAATTCCAGCGCCTTCCAGTTAGGGTGGTTATTGCCGAGTAGGGGGCCCAGAACGTTTCTGTGTC
CGCAAAATACCCTGTAGCTGCATTTTGCTGTTGTCATGTAGCTCGGCACGTTATTGATACAGTCATTGAGCA
C

No repetitive sequences in the immediate proximity.

CLONE 252

[LZ3,50]

Driver: ORF2
Plasmid: AlurescueA70Du $A_{17}CATTACA_{18}GA_{17}CACACA_{18}$ (T)
Chromosome: 17
5' position: 39,958,396
Strand: minus
DR: N/A
ENDOsite: N/A

Empty site:

CCATCTTCTCCTGCAGCTAAGCCAGGGCAAGGCACTAGAGACCCACATCCTTCCCATGCCACCAACTCGTCA
GGTCCCACCAAGCAAGCCACTCACCCTACAGCCCAGCAACCATGGCTTGCCTCTCGTGCCACTGCACTCCAG
CCTGGGCGACAGAGTGAGACTCCATCTCAAAAAAAAAAAAAAAAAACTTAAGATGGACACAGCTGACTGGAC
CCCCATCCTGCCTCACCCATGGGTGCTGCACCCCAGACCCATCCTGCCACTTCTATGTCTCTGGACCACAGG
ATGGTGGTGGCATTGCAGGTTGGCAAGTGGGCTGAT↑↓GGGGTCCGCCCTCCTCACTGCTGAGCTCCTCACC
TGGACAGTCTCCTGGACAAGGAGTTTCCAGCTGCTGGCTGGAGTCTCAGGCCAAATTGCAGAGGGTCCTCCA
GGGTCCTGAAGAGCACTGGACTAAGAGTCTAGTGGTTCCAGGGCCCTGACCAGTAGGTGCTCAATAAATGTT
TGTTGTTGAATGAGTCATAGTA

Filled site:

CCATCTTCTCCTGCAGCTAAGCCAGGGCAAGGCACTAGAGACCCACATCCTTCCCATGCCACCAACTCGTCA
GGTCCCACCAAGCAAGCCACTCACCCTACAGCCCAGCAACCATGGCTTGCCTCTCGTGCCACTGCACTCCAG
CCTGGGCGACAGAGTGAGACTCCATCTCAAAAAAAAAAAAAAAAAACTTAAGATGGACACAGCTGACTGGAC
CCCCATCCTGCCTCACCCATGGGTGCTGCACCCCAGACCCATCCTGCCACTTCTATGTCTCTGGACCACAGG
ATGGTGGTGGCATTGCAGGTTGGCAAGTGGGCTGAT**CTCACGCCTGTAATCCCAGCACTTTGGGAGGCCGAG
GCGGGCGGATCACGAGGTCAGGAGATCGAGACCATCCTGGCTAACACGGTGAAACCCCGTCTCTACTAAAAA
AAAATACAAAAAATTAGCCGGGCGTGGTAGCGGGCGCCTGTAGTCCCAGCTACTCGGGAGGCTGAGGCAGGG
GAATGGCGTGAACCCGGGAGGCGGAGCTTGCAGTGAGCCGAGATCCCGCCACTGC…[NEOcassette][A
tail and 3' unsequenced]**

Alu insert truncation: missing 15 bp of 5' Alu sequence.

Alu in the 5'; L2 in the 3'.

CLONE 253

[GG31]

Driver: ORF2
Plasmid: AlurescueA70Du A_{17}CATTACA$_{18}$GA$_{17}$CACACA$_{18}$ (T)
Chromosome: 17
5' position: 48,175,331
Strand: plus
DR: AAGAAGTAAGTTTTC
ENDOsite: TCTT/GT

Empty site:

GGGGTGGGGACAGACAGGAGACACCAAAGCCCCAAGCGCTTGGAGTTGCCTAGGGTCACGTGGGAATCTGAG
GCCAGAGCAGGACTGAGGTCAAGCTCCTAGTCTCTGTGTCTAGCCCTCTTCTTACCACCCACCTTTCTCTGG
AAACACAGGATCCACCTAGGAAAGCATGGTTTTCACAGCCAAAACCCCAATTCAGGCTGATGTCAGCATTAG
CCAGCAGCCCAGACCTGGAACTCCAGTAACTGTTAGGCAGTATTTATTTGGGGGTAACTTTTTGGGAAGCAG
AAAC↑**AAGAAGTAAGTTTTC**↓AAATACATTATATAACCCAAGCTGCAAGACAGACTGTTTTGGATGTTGGAA
AATACTCAGATACACACAAGGATGGCACAGTACCTTCCCGAAGAGCTTTGGAGGGGCAAAGACCAAACGTTT
ATTGCGCACTGCAGCAGGAACAGAGGGAAAGGCTCAGCAGAGTTCACTCAAGAACTCAAGTTAAGTAACTGG
ATGAA

Filled site:

GGGGTGGGGACAGACAGGAGACACCAAAGCCCCAAGCGCTTGGAGTTGCCTAGGGTCACGTGGGAATCTGAG
GCCAGAGCAGGACTGAGGTCAAGCTCCTAGTCTCTGTGTCTAGCCCTCTTCTTACCACCCACCTTTCTCTGG
AAACACAGGATCCACCTAGGAAAGCATGGTTTTCACAGCCAAAACCCCAATTCAGGCTGATGTCAGCATTAG
CCAGCAGCCCAGACCTGGAACTCCAGTAACTGTTAGGCAGTATTTATTTGGGGGTAACTTTTTGGGAAGCAG
AAACAAGAAGTAAGTTTTCT**GGCCGGGCGCGGTGGCTCACGCCTGTAATCCCAGCACTTTGGGAGGCCGAGG**
CGGGCGGATCACGAGGTCAGGAGATCGAGACCATCCTGGCTAACACGGTGAAACCCCGTCTCTACTAAAAAA
AAAATACAAAAAATTAGCCGGGCGTGGTAGCGGGCGCCTGTAGTCCCAGCTACTCGGGAGGCTGAGGCAGGG
GAATGGCGTGAACCCGGGAGGCGGAGCTTGC... **[NEOcassette] [Atail]** AAGAAGTAAGTTTTCAAA
TACATTATATAACCCAAGCTGCAAGACAGACTGTTTTGGATGTTGGAAAATACTCAGATACACACAAGGATG
GCACAGTACCTTCCCGAAGAGCTTTGGAGGGGCAAAGACCAAACGTTTATTGCGCACTGCAGCAGGAACAGA
GGGAAAGGCTCAGCAGAGTTCACTCAAGAACTCAAGTTAAGTAACTGGATGAA

No repetitive sequences in the immediate proximity.

An extra T added immediately 5' of Alu.

CLONE 254

[LZ68]

Driver: ORF2
Plasmid: AlurescueA70Du $A_{17}CATTACA_{18}GA_{17}CACACA_{18}$ (T)
Chromosome: 17
5’ position: 61,238,975
Strand: minus
DR: N/A
ENDOsite: N/A

Empty site:

GTTGCAATTTTAATAGTGTCAATGCAAAGTCTCAATGAAAAGGTGACATTTTAGCAAAGATCTTAAAAAAGG
CAAGGGAACAGGGTAGGGCTAGTATTAAAAATATTTAACAACTGGGACAGCATGAGCACCTACTACTAATCG
GTGGATGTACACTTGAGCAGGGTCTTAGAGGTCATCAAAGTAAATCTGGTGATGACAGCAGAGTCATCAATA
TATACATGTCCTTAAAAGATGTGGGCCTGAATGAGAATACCAACAAAGTGAGCACAGAGAAGATATCTATGG
TCCAGGTCTTAGAGTATTCCAACTTTTAGAAGTGGGAGAAGAAAAGGAAGTAGCA↑↓AAATACACTATGAAA
ATGATGCCCGAGAATTAGGAAGAAAACAAGAATAAGGTGATAACTTGGAGTCACAGAATAAGTGCTTTGGGG
AAGAGGGAGTCAGTAACTGTGTTAAGTGTTGCAGGTAAGTTAAATAAAATGAGAACTGGAAACAAGCATTAG
ATTTAGCAATGCAGAGGGAAGAAAGTGACTGCAACAAATGG

Filled site:

GTTGCAATTTTAATAGTGTCAATGCAAAGTCTCAATGAAAAGGTGACATTTTAGCAAAGATCTTAAAAAAGG
CAAGGGAACAGGGTAGGGCTAGTATTAAAAATATTTAACAACTGGGACAGCATGAGCACCTACTACTAATCG
GTGGATGTACACTTGAGCAGGGTCTTAGAGGTCATCAAAGTAAATCTGGTGATGACAGCAGAGTCATCAATA
TATACATGTCCTTAAAAGATGTGGGCCTGAATGAGAATACCAACAAAGTGAGCACAGAGAAGATATCTATGG
TCCAGGTCTTAGAGTATTCCAACTTTTAGAAGTGGGAGAAGAAAAGGAAGTAGCA**GGCCGGGCGCGGTGGCT
CACGCCTGTAATCCCAGCACTTTGGGAGGCCGAGGCGGGCGGATCACGAGGTCAGGAGATCGAGACCATCCT
GGCTAACACGGTGAAACCCCGTCTCTACTAAAAAAAAAATACAAAAAATTAGCCGGGCGTGGTAGCGGGCGC
CTGTAGTCCCAGCTACTCGGGAGGCTGAGGCAGGGGAATGGCGTGAACCCGGGAGGCGGAGCTTGCAGTGAG
CCGAGATCCCGCC…[NEOcassette][Atail and 3' unsequenced]**

L2 flanking the insertion site; DNA element in the 5'.

CLONE 255

[TZ10]

Driver: ORF2
Plasmid: AlurescueA70Du $A_{17}CATTACA_{18}GA_{17}CACACA_{18}$ (T)
Chromosome: 17
5' position: 62,685,113
Strand: plus
DR: N/A
ENDOsite: N/A

Empty site:

GGAGGATCACATGAGCCCAGGAATTTGAGGCTGAAGTGAGCTATGATCACACCACTGCACTCCAGCCTGGGC
AGCAGGGCCAAACTCCATCTCTAAAAACAAATAGGCCGGGCACAGTGGCTGACACCTGTAATCCCGGCATTT
TGCAGGCCAAGGAGCGAGGATGGGTTGAGCTCAGAAGTTAGAGACCAGCCTGAGCAACATAGTGAGACCTCG
TCTCTATTAAAACTTAGCCAGGTATAGTGATCTGTACCTGTAGTCCCAGCTACTCAGGAGGCTGAGGCAGGA
GGATTGCTTGAGCCAAGGAGGTCAAGTCTGCAGTGAGCTATAATCACCACTGCA↑↓CTCCAACCTTAGTGAC
AGAGTGAAACCCTGTCTCAAAAAAATAAAAATAAAAACAAACAAACAAAAACCCTGTGATGTCACCAAGCCT
CCCTAGCAGGGTGTACCGTTCAGGTTTGTTGTAGAACAAACAACCCCATAATGGAGCAAGTAAAAGCACAAT
AATTTCTTTAGCTCACAATTATATGGCTCAGCAATTTGGG

Filled site:

GGAGGATCACATGAGCCCAGGAATTTGAGGCTGAAGTGAGCTATGATCACACCACTGCACTCCAGCCTGGGC
AGCAGGGCCAAACTCCATCTCTAAAAACAAATAGGCCGGGCACAGTGGCTGACACCTGTAATCCCGGCATTT
TGCAGGCCAAGGAGCGAGGATGGGTTGAGCTCAGAAGTTAGAGACCAGCCTGAGCAACATAGTGAGACCTCG
TCTCTATTAAAACTTAGCCAGGTATAGTGATCTGTACCTGTAGTCCCAGCTACTCAGGAGGCTGAGGCAGGA
GGATTGCTTGAGCCAAGGAGGTCAAGTCTGCAGTGAGCTATAATCACCACTGCA**GGCCGGGCGCGGTGGCTC**
ACGCCTGTAATCCCAGCACTTTGGGAGGCCGAGGCGGGCGGATCACGAGGTCAGGAGATCGAGACCATCCTG
GCTAACACGGTGAAACCCCGTCTCTACTAAAAAAAAATACAAAAAATTAGCCGGGCGTGGTAGCGGGCGCCT
GTAGTCCCAGCTACTCGGGAGGCTGAGGCAGGGGAATGGCGTGAACCCGGGAGGCGGAGCTTGCAGTGAGCC
GAGATCCCGCC…[NEOcassette][Atail and 3' unsequenced]

Alu flanking the insertion site; LTR in the 3'.

CLONE 256
[BC1-18,32,37,38]

Driver: ORF2
Plasmid: AlurescueA70Du $A_{17}CATTACA_{18}GA_{17}CACACA_{18}$ (T)
Chromosome: 17
5' position: 65,857,789
Strand: minus
DR: N/A
ENDOsite: N/A

Empty site:

AGACTTGTATCAAAAAACAAAACAAAACAAAAAAACTATTGGTAGGATGGGTGTTTTAAAAAGTAGTATTTC
ACGATGCAAGAGAAAAAGGGGTTCAGGGAAAGAAAGGGGTGCAGGCAAAGAAATCCCTAGGAAGTAGCATAA
ATAGGCTGAGACCTCAAGAATGAGCTAGTTAGCCAACAGGTTTTTAGGTGCTTTGGTTTGCTTTTTATTTAT
GTTGTCTTAAGTGTTAGAAAGCAAGATAATTAAATGTACAAAGTTAGGGATCTGTGCTAAAGAATCTGAAGT
TTTGGTTAAGGGCAAATGGAAGTAATTGTAATTAAAAGGTTTTTC↑↓AACTTAGGGGAAAAGGAGACTTGAT
CAAAATTGCTGTTTTAAAAATCATCCAGCTATGGCTGGGCATGGTGGCTCACATCTGTAATCCCAGCACATT
TGGAGGCTGAGGTGGGCTGATCACCTGAGGTCAGGAGTTCAATATCAGCCTGGCCAACATGGTGAAACCCTG
TCTCTACTAAAAATACAAAATTAGCCAGGTG

Filled site:

AGACTTGTATCAAAAAACAAAACAAAACAAAAAAACTATTGGTAGGATGGGTGTTTTAAAAAGTAGTATTTC
ACGATGCAAGAGAAAAAGGGGTTCAGGGAAAGAAAGGGGTGCAGGCAAAGAAATCCCTAGGAAGTAGCATAA
ATAGGCTGAGACCTCAAGAATGAGCTAGTTAGCCAACAGGTTTTTAGGTGCTTTGGTTTGCTTTTTATTTAT
GTTGTCTTAAGTGTTAGAAAGCAAGATAATTAAATGTACAAAGTTAGGGATCTGTGCTAAAGAATCTGAAGT
TTTGGTTAAGGGCAAATGGAAGTAATTGTAATTAAAAGGTTTTTC**GGCCGGGCGCGGTGGCTCACGCCTGTA**
ATCCCAGCACTTTGGGAGGCCGAGGCGGGCGGATCACGAGGTCAGGAGATCGAGACCATCCTGGCTAACACG
GTGAAACCCCGTCTCTACTAAAAAAAAATACAAAAAATTAGCCGGGCGTGGTAGCGGGCGCCTGTAGTCCCA
GCTACTCGGGAGGCTGAGGCAGGGGAATGGCGTGAACCCGGGAGGCGGAGCTTGCAGTGAGCCGAGATCCCG
CCACTGCACTCCA…[NEOcassette][Atail and 3' unsequenced]

Alu in the 3'.

CLONE 257

[LZ23]

Driver: ORF2
Plasmid: AlurescueA70Du $A_{17}CATTACA_{18}GA_{17}CACACA_{18}$ (T)
Chromosome: 17
5' position: 68,043,422
Strand: plus
DR: AAAAAGTAATTAATA
ENDOsite: TTTT/AA

Empty site:

TTTAAAAAAAAAAACAAGTAAATAAACCATAAGTTAAACATTCAGTATTGTTCATATCCAATCACCTAAACA
AATAAAATGTAATATCTGTTCCCAAATATGATGGCTATATTTTGTATTATTACAGAAATAGTTGAATTCAGA
GCCAATTTTGATTTCTTGTTTCCTAGTTCAGTTAATAGGAAGCAATTGTGAATAATACAGGCACCTCCTGAA
AGAGGCAATTCTTTAATAGAAATTCTAGTTTGACTTCTTTACTGTTTGGTACAATACTATTTTCTCCAATTT
TT↑**AAAAAGTAATTAATA**↓ATTAATTATTGTTATTGAAAGTTTTGCTGTTGTTTCTACTATGGAGCTACTAT
GACAACTACATTAGCAATTCTGTTAAGGTACTGGTGCATAGTATTTGAAAGTTTGCAAGCCATAGAGCCAGG
CGTGGTGGCCAGTGCCTATAATCCCAGCTAATTGAGAGGCTAAGGAGTTGGGAGGATTGCACGAGCCCAAGA
ATT

Filled site:

TTTAAAAAAAAAAACAAGTAAATAAACCATAAGTTAAACATTCAGTATTGTTCATATCCAATCACCTAAACA
AATAAAATGTAATATCTGTTCCCAAATATGATGGCTATATTTTGTATTATTACAGAAATAGTTGAATTCAGA
GCCAATTTTGATTTCTTGTTTCCTAGTTCAGTTAATAGGAAGCAATTGTGAATAATACAGGCACCTCCTGAA
AGAGGCAATTCTTTAATAGAAATTCTAGTTTGACTTCTTTACTGTTTGGTACAATACTATTTTCTCCAATTT
TTAAAAAGTAATTAATA**GGCCGGGCGCGGTGGCTCACGCCTGTAATCCCAGCACTTTGGGAGGCCGAGGCGG**
GCGGATCACGAGGTCAGGAGATCGAGACCATCCTGGCTAACACGGTGAAACCCCGTCTCTACTAAAAAAAAA
TACAAAAAATTAGCCGGGCGTGGTAGCGGGCGCCTGTAGTCCCAGCTACTCGGGAGGCTGAGGCAGGGGAAT
GGCGTGAACCCGGGAGGCGGAGCTTGCAGTGAGCCGAGATCCCGCC…[NEOcassette][Atail]AAA
AAGTAATTAATAATTAATTATTGTTATTGAAAGTTTTGCTGTTGTTTCTACTATGGAGCTACTATGACAACT
ACATTAGCAATTCTGTTAAGGTACTGGTGCATAGTATTTGAAAGTTTGCAAGCCATAGAGCCAGGCGTGGTG
GCCAGTGCCTATAATCCCAGCTAATTGAGAGGCTAAGGAGTTGGGAGGATTGCACGAGCCCAAGAATT

Alu in the 3'.

CLONE 258

[LZ32]

Driver: ORF2
Plasmid: AlurescueA70Du $A_{17}CATTACA_{18}GA_{17}CACACA_{18}$ (T)
Chromosome: 18
5' position: 32,733,002
Strand: plus
DR: TAAAACAATAAATCC
ENDOsite: TTTA/AT

Empty site:

AAAGCAATTCAATGAGCTAATCTCAGTAAACCTAGAATTAAAGTTCTTGTTTCCATATTTCACTTTCAAAAT
ATTTAGAACTTTGACAGATATGACATTATGTTTTCATTAAAACAGCTGATTTCAAAATGTTGCTCTGTGCAG
AAACGCCATAAACAGTTTATGATGAACTGCCACGCTCTTAAT↑**TAAAACAATAAATCC**↓AGCACACAATATT
TGTGCAGCCTCCGGTCTGACCTTTGCCTCTTGCTATCCACCACCAAGCCGCGGCTGCGTTCCCAACGCCAGA
CGTGGAAGGTTTTAAAACATGATCTGAAAGGCACTAAAGATAAAATAAGAGAAAACGTACCATTAGTCTTCG
CAGTTATGATTAATTCAGTTCATATTTTACAATCTGGTTATGG

Filled site:

AAAGCAATTCAATGAGCTAATCTCAGTAAACCTAGAATTAAAGTTCTTGTTTCCATATTTCACTTTCAAAAT
ATTTAGAACTTTGACAGATATGACATTATGTTTTCATTAAAACAGCTGATTTCAAAATGTTGCTCTGTGCAG
AAACGCCATAAACAGTTTATGATGAACTGCCACGCTCTTAATTAAAACAATAAATCC**TGGCCGGGCGCGGTG**
GCTCACGCCTGTAATCCCAGCACTTTGGGAGGCCGAGGCGGGCGGATCACGAGGTCAGGAGATCGAGACCAT
CCTGGCTAACACGGTGAAACCCCGTCTCTACTAAAAAAAAAATACAAAAAATTAGCCGGGCGTGGTAGCGGG
CGCCTGTAGTCCCAGCTACTCGGGAGGCTGAGGCAGGGGAATGGCGTGAACCCGGGAGGCGGAGCTTGCAGT
GAGCCGAGATCCCGCC…[NEOcassette][Atail]TAAAACAATAAATCCAGCACACAATATTTGTGC
AGCCTCCGGTCTGACCTTTGCCTCTTGCTATCCACCACCAAGCCGCGGCTGCGTTCCCAACGCCAGACGTGG
AAGGTTTTAAAACATGATCTGAAAGGCACTAAAGATAAAATAAGAGAAAACGTACCATTAGTCTTCGCAGTT
ATGATTAATTCAGTTCATATTTTACAATCTGGTTATGG

No repetitive sequences in the immediate proximity.

An extra T added immediately 5' of Alu.

CLONE 259

[LZ51]

Driver: ORF2
Plasmid: AlurescueA70Du $A_{17}CATTACA_{18}GA_{17}CACACA_{18}$ (T)
Chromosome: 19
5' position: 57,679,450
Strand: plus
DR: TAAAAATTCTGTGATCAA
ENDOsite: TTTA/GC

Empty site:

TGCTCCTTGGCTCCCCCAACCCCCTTGTGGGAAAAAAATTCGTCCCAAATCACTGGAACCTGAACAGGGAGA
CAAGCTTGCAATAGTTGCCAATTTTTTAAAAAAGAATATTTTTGAGTACTATTCTGCTTACACATATTTTTC
AATGCTTATTATATACATATAGTCTTTTGTAAATGGGGTTTTTATGCTCTCCACCTATCTTTTGACATAGTG
GCCTCTCCTTATAAATTAAAAGTGTTATTTCTAATTAGTATCACATAAGAGTGAGGCTAGTGAGACAGGAAC
AAAATTTAAGGTGGTGC↑**TAAAAATTCTGTGATCAA**↓GATAAATAGTATTTGAAAGCAATGTATATTTTAAG
TAATGTAAAAAGTCCATCTTAACAAAACATAAAGATTGTAAATAAAAAGAAGATCCCACCCTGCACGTGCAC
GACCCTGCAAGTTATGCAAGTGAGGGTACCTGCGCATTGAAAAAAGATGTCTCACCATTGACCAGTCATTGA
TCAGTCATTGATCAGTCTTGA

Filled site:

TGCTCCTTGGCTCCCCCAACCCCCTTGTGGGAAAAAAATTCGTCCCAAATCACTGGAACCTGAACAGGGAGA
CAAGCTTGCAATAGTTGCCAATTTTTTAAAAAAGAATATTTTTGAGTACTATTCTGCTTACACATATTTTTC
AATGCTTATTATATACATATAGTCTTTTGTAAATGGGGTTTTTATGCTCTCCACCTATCTTTTGACATAGTG
GCCTCTCCTTATAAATTAAAAGTGTTATTTCTAATTAGTATCACATAAGAGTGAGGCTAGTGAGACAGGAAC
AAAATTTAAGGTGGTGCTAAAAATTCTGTGATCAA**GGCCGGGCGCGGTGGCTCACGCCTGTAATCCCAGCAC**
TTTGGGAGGCCGAGGCGGGCGGATCACGAGGTCAGGAGATCGAGACCATCCTGGCTAACACGGTGAAACCCC
GTCTCTACTAAAAAAAAATACAAAAAATTAGCCGGGCGTGGTAGCGGGCGCCTGTAGTCCCAGCTACTCGGG
AGGCTGAGGCAGGGGAATGGCGTGAACCCGGGAGGCGGAGCTTGCAGTGAGCCGAGATCCCGCCACTGCACT
CC…[NEOcassette][Atail]TAAAAATTCTGTGATCAAGATAAATAGTATTTGAAAGCAATGTATAT
TTTAAGTAATGTAAAAAGTCCATCTTAACAAAACATAAAGATTGTAAATAAAAAGAAGATCCCACCCTGCAC
GTGCACGACCCTGCAAGTTATGCAAGTGAGGGTACCTGCGCATTGAAAAAAGATGTCTCACCATTGACCAGT
CATTGATCAGTCATTGATCAGTCTTGA

DNA element flanking the insertion site.

CLONE 260

[LZ82]

Driver: ORF2
Plasmid: AlurescueA70Du $A_{17}CATTACA_{18}GA_{17}CACACA_{18}$ (T)
Chromosome: 20
5' position: 43,781,473
Strand: plus
DR: AAGAAATTCCT
ENDOsite: TCTT/AA

Empty site:

ACATCCAATGCATGGATCACATTCAGAATTATTGCAGAGGGAAGCAATGAAATGGCAGCAAGAAAATTAACA
TCTTTTCAACTCGCCCAAGACATGACTCACAGTTTCTTTTTTCTGAGTTGATCTCCATCAAGTGCATTAATA
ATTTTTCTCCAAGTTCTTTCAGTGAGCAGTTTCTAGGGGATCTTTGGCATAGTTGAAGACAATAGAAGGCAG
AAGAGGAGGAATGAGTAACTTCCACAATCTCCTCCTCAAGTGGTATAAAATTACTCTATCATT↑**AAGAAATT**
CCT↓AAGCAAACTTCACCCTCAACATAACTTGAAAACAAAGAATGCCAAAAATACAAAATACCTGTCAGTGA
AAAGGGAACAAACAACAAATCCCAGTGCTGGGGACTCACACTTACCTGCCCTTTCCAGCCCCACTATAAAGC
TTAAAGCTTTGCAAAATTGACCAAAACACACACACACACACACACACACACACACCCCACTG

Filled site:

ACATCCAATGCATGGATCACATTCAGAATTATTGCAGAGGGAAGCAATGAAATGGCAGCAAGAAAATTAACA
TCTTTTCAACTCGCCCAAGACATGACTCACAGTTTCTTTTTTCTGAGTTGATCTCCATCAAGTGCATTAATA
ATTTTTCTCCAAGTTCTTTCAGTGAGCAGTTTCTAGGGGATCTTTGGCATAGTTGAAGACAATAGAAGGCAG
AAGAGGAGGAATGAGTAACTTCCACAATCTCCTCCTCAAGTGGTATAAAATTACTCTATCATTAAGAAATTC
CT**GGCCGGGCGCGGTGGCTCACGCCTGTAATCCCAGCACTTTGGGAGGCCGAGGCGGGCGGATCACGAGGTC**
AGGAGATCGAGACCATCCTGGCTAACACGGTGAAACCCCGTCTCTACTAAAAAAAAAATACAAAAAATTAGC
CGGGCGTGGTAGCGGGCGCCTGTAGTCCCAGCTACTCGGGAGGCTGAGGCAGGGGAATGGCGTGAACCCGGG
AGGCGGAGCTTGCAGTGAGCCGAGATCCCGCCACTGCACTCC…[NEOcassette][Atail]AAGAAAT
TCCTAAGCAAACTTCACCCTCAACATAACTTGAAAACAAAGAATGCCAAAAATACAAAATACCTGTCAGTGA
AAAGGGAACAAACAACAAATCCCAGTGCTGGGGACTCACACTTACCTGCCCTTTCCAGCCCCACTATAAAGC
TTAAAGCTTTGCAAAATTGACCAAAACACACACACACACACACACACACACACACCCCACTG

LINE-1 in the 3'.

CLONE 261

[LZ10]

Driver: ORF2
Plasmid: AlurescueA70Du $A_{17}CATTACA_{18}GA_{17}CACACA_{18}$ (T)
Chromosome: 20
5' position: 48,437,556
Strand: plus
DR: AAGAGTTCTTTATATAT
ENDOsite: TCTT/AA

Empty site:

CACCATGTTGGCCAGGCTGGTCTTGAACTCCGACCTCAGGTGATCTGCTTGCCTCAGCCTCCCAAAGTGCTG
GGATTCCAGGTGTGAGCCACCGCACCAGGCCCTCACTGTATTTTTTAAAAAATCTTACATGTCCTTCCAGTG
AATCATTGTATTTTGATTTTAGTTTCCCTTGTGACTATTGATGTTGAACATCTTTTGTCTATTCATGTCCTA
TGCCCGTTTAAAAGCTGTGGTGTTTGTCTCTTGGCTGTTGAGTTT↑**AAGAGTTCTTTATATAT**↓TGTGGATA
GTACACCTTTATCAGATATATGATTTGCAAATATTTTCTCCCCTTCTATGGATTGTCTTTTCTGACTTTATT
CTTTTTAATGGCTGAAGGCTGTTACCTGGCATTGATGAGCCTTATATTAACTCTTTCTGTTAATAGTTTCCC
CTTTTGCTCTTAAAGCCGCAAAGAGTTGTTTGATCTATTTATGAGAAT

Filled site:

CACCATGTTGGCCAGGCTGGTCTTGAACTCCGACCTCAGGTGATCTGCTTGCCTCAGCCTCCCAAAGTGCTG
GGATTCCAGGTGTGAGCCACCGCACCAGGCCCTCACTGTATTTTTTAAAAAATCTTACATGTCCTTCCAGTG
AATCATTGTATTTTGATTTTAGTTTCCCTTGTGACTATTGATGTTGAACATCTTTTGTCTATTCATGTCCTA
TGCCCGTTTAAAAGCTGTGGTGTTTGTCTCTTGGCTGTTGAGTTTAAGAGTTCTTTATATAT**GGCCGGGCGC
GGTGGCTCACGCCTGTAATCCCAGCACTTTGGGAGGCCGAGGCGGGCGGATCACGAGGTCAGGAGATCGAGA
CCATCCTGGCTAACACGGTGAAACCCCGTCTCTACTAAAAAAAAATACAAAAAATTAGCCGGGCGTGGTAGC
GGGCGCCTGTAGTCCCAGCTACTCGGGAGGCTGAGGCAGGGGAATGGCGTGAACCCGGGAGGCGGAGCTTGC
AGTGAGCCGAGATCCCGCCACTGCACTCCAG…[NEOcassette][Atail]**AAGAGTTCTTTATATATT
GTGGATAGTACACCTTTATCAGATATATGATTTGCAAATATTTTCTCCCCTTCTATGGATTGTCTTTTCTGA
CTTTATTCTTTTTAATGGCTGAAGGCTGTTACCTGGCATTGATGAGCCTTATATTAACTCTTTCTGTTAATA
GTTTCCCCTTTTGCTCTTAAAGCCGCAAAGAGTTGTTTGATCTATTTATGAGAAT

Alu in the 5'; LINE-1 flanking the insertion site.

CLONE 262

[LZ61,66]

Driver: ORF2
Plasmid: AlurescueA70Du $A_{17}CATTACA_{18}GA_{17}CACACA_{18}$ (T)
Chromosome: 20
5' position: 52,835,539
Strand: minus
DR: AAAAAAAAATATTTTA
ENDOsite: TTTT/CC

Empty site:

CATTTGAAGAAGGAAAATTATCATTTTAAACAATATTCAAGTTTATTAAACAAATAAAAAAAGTATTATAAA
ATGTTTAACTTTCATCAGCTTCAAGGTTTATGTTGCTCCCGAATTTTGCATACAACTGAACTTTCAAATCTG
CTAACACTCGCTGAATTGATTCCACTCTGGATTCTAAGGCGTCAATTTCTTCTTGCAAATTTTTCTGG↑**AAA**
AAAAAATATTTTA↓AATTAATTCTATTCAATAGGATGATTTCTGAGCATCTGTGTCTGAAATAATATTTGGG
AAGAATTTTAGGTCAAGAACCTTCTAAACTTGGACAGGCAGCTAAGTAACACTGATAATTAGATCTAAATGT
CTGAATGTGGGCATTTTTAATTCATGGTCCATATCTGCTTATCACAGAATAAATCAAGTTTGTTTTCTCA

Filled site:

CATTTGAAGAAGGAAAATTATCATTTTAAACAATATTCAAGTTTATTAAACAAATAAAAAAAGTATTATAAA
ATGTTTAACTTTCATCAGCTTCAAGGTTTATGTTGCTCCCGAATTTTGCATACAACTGAACTTTCAAATCTG
CTAACACTCGCTGAATTGATTCCACTCTGGATTCTAAGGCGTCAATTTCTTCTTGCAAATTTTTCTGGAAAA
AAAAATATTTTA**GGCCGGGCGCGGTGGCTCACGCCTGTAATCCCAGCACTTTGGGAGGCCGAGGCGGGCGGA**
TCACGAGGTCAGGAGATCGAGACCATCCTGGCTAACACGGTGAAACCCCGTCTCTACTAAAAAAAAATACAA
AAAATTAGCCGGGCGTGGTAGCGGGCGCCTGTAGTCCCAGCTACTCGGGAGGCTGAGGCAGGGGAATGGCGT
GAACCCGGGAGGCGGAGCTTGCAGTGAGCCGAGATCCCGCC…[NEOcassette][Atail]AAAAAAAA
ATATTTTAAATTAATTCTATTCAATAGGATGATTTCTGAGCATCTGTGTCTGAAATAATATTTGGGAAGAAT
TTTAGGTCAAGAACCTTCTAAACTTGGACAGGCAGCTAAGTAACACTGATAATTAGATCTAAATGTCTGAAT
GTGGGCATTTTTAATTCATGGTCCATATCTGCTTATCACAGAATAAATCAAGTTTGTTTTCTCA

No repetitive sequences in the immediate proximity.

CLONE 263

[LZ8]

Driver: ORF2
Plasmid: AlurescueA70Du $A_{17}CATTACA_{18}GA_{17}CACACA_{18}$ (T)
Chromosome: 20
5' position: 59,087,654
Strand: minus
DR: GAAAATCTCA
ENDOsite: TTTC/AA

Empty site:

GAATTCCAATTATGCTAACTGCCATGAAAAAGTGAGCAGTATTGTGAAAGTTTCCAGGAATATGCGTCGTTT
AAAATGAGACTTAAAGAGTCATTGGAAATTGGTCAGGTTAAAAGAGGGAAGAAAATTATCCTAAGCTGAAGA
AACAATTAAAAAAAAAATCAAACCACGACATACTT↑**GAAAATCTCA**↓AAGGCTGGCATGCCTGGAGCAGAGC
AAGCAATGGAAAAGATGCTGAGAAATGAGGAAATGAGGACAGATCTGCAGTTCTCCACTTTTACTGCATGTG
AAAATCACATGGATCGCTTTGAAAACGTTATCTATGTGCAGGCCCTATTCTAGTTAGCTAAATCAAAAGCTC
CTTCTTGGGGGGAAGAAAAAAGCTCTCTAGT

Filled site:

GAATTCCAATTATGCTAACTGCCATGAAAAAGTGAGCAGTATTGTGAAAGTTTCCAGGAATATGCGTCGTTT
AAAATGAGACTTAAAGAGTCATTGGAAATTGGTCAGGTTAAAAGAGGGAAGAAAATTATCCTAAGCTGAAGA
AACAATTAAAAAAAAAATCAAACCACGACATACTTGAAAATCTCA**GGCCGGGCGCGGTGGCTCACGCCTGTA
ATCCCAGCACTTTGGGAGGCCGAGGCGGGCGGATCACGAGGTCAGGAGATCGAGACCATCCTGGCTAACACG
GTGAAACCCCGTCTCTACTAAAAAAAAAAATACAAAAAATTAGCCGGGCGTGGTAGCGGGCGCCTGTAGTCC
CAGCTACTCGGGAGGCTGAGGCAGGGGAATGGCGTGAACCCGGGAGGCGGAGCTTGCAGTGAGCCGAGATCC
CGCCACTGCA…[NEOcassette][Atail]**GAAAATCTCAAAGGCTGGCATGCCTGGAGCAGAGCAAGC
AATGGAAAAGATGCTGAGAAATGAGGAAATGAGGACAGATCTGCAGTTCTCCACTTTTACTGCATGTGAAAA
TCACATGGATCGCTTTGAAAACGTTATCTATGTGCAGGCCCTATTCTAGTTAGCTAAATCAAAAGCTCCTTC
TTGGGGGGAAGAAAAAAGCTCTCTAGT

L2 flanking the insertion site; hAT-Charlie DNA element in the 3'.

CLONE 264

[LZ49]

Driver: ORF2
Plasmid: AlurescueA70Du $A_{17}CATTACA_{18}GA_{17}CACACA_{18}$ (T)
Chromosome: 21
5' position: 46,280,278
Strand: plus
DR: AAAAATAAATAAATA
ENDOsite: TTTT/GA

Empty site:

CACACCACAAACTGATTTAAAAGGCAAACTACGGGGCTGGGCGCAGTGGCTCACGCCTGTAATCCCAGCACT
TTGGGAGGCTGAGGTGGGCAGATCATGAGGTCAGGAGATCGAGACCATCCTGGCTAACACAGTGAAACCCCG
TCTCTACTAAAAAATACAAGAAATTAGCCGGACGTGGTGGCGGGCACCTGTAGTCCCAGCTACTCAGGAGGC
TGAGGCAGGAGAATGGCGTGAACCTGGGAGGCGGAGCTTGCAGTGAGCCAAGATAGTGCCATTGCACTCCAG
CCCGTCTC↑**AAAAATAAATAAATA**↓AATAAATAAAGGCAAACTAGAAAACAACTTGAATCTCACAAGAGCTG
ATTTCCTTAATTTAAAAACGCCTCCAAAATCAAATATACAAAGATACGCACACAAAAAAAGAGGGCTGTCTA
CGCAAGCAGGGATTCTTAAGCTGGGAAAACTGGAGATTCTAGGGGAATTCACAGGTGAGTTTCAGGGGTGAC
ACATGAACA

Filled site:

CACACCACAAACTGATTTAAAAGGCAAACTACGGGGCTGGGCGCAGTGGCTCACGCCTGTAATCCCAGCACT
TTGGGAGGCTGAGGTGGGCAGATCATGAGGTCAGGAGATCGAGACCATCCTGGCTAACACAGTGAAACCCCG
TCTCTACTAAAAAATACAAGAAATTAGCCGGACGTGGTGGCGGGCACCTGTAGTCCCAGCTACTCAGGAGGC
TGAGGCAGGAGAATGGCGTGAACCTGGGAGGCGGAGCTTGCAGTGAGCCAAGATAGTGCCATTGCACTCCAG
CCCGTCTCAAAAATAAATAAATA**GGCCGGGCGCGGTGGCTCACGCCTGTAATCCCAGCACTTTGGGAGGCCG**
AGGCGGGCGGATCACGAGGTCAGGAGATCGAGACCATCCTGGCTAACACGGTGAAACCCCGTCTCTACTAAA
AAAAAAAAATACAAAAAATTAGCCGGGCGTGGTAGCGGGCGCCTGTAGTCCCAGCTACTCGGGAGGCTGAGG
CAGGGGAATGGCGTGAACCCGGGAGGCGGAGCTTGCAGTGAGCCGAGATCCCGCCACTGCACTC…[NEOca
ssette][Atail]AAAAATAAATAAATAAATAAATAAAGGCAAACTAGAAAACAACTTGAATCTCACAAG
AGCTGATTTCCTTAATTTAAAAACGCCTCCAAAATCAAATATACAAAGATACGCACACAAAAAAAGAGGGCT
GTCTACGCAAGCAGGGATTCTTAAGCTGGGAAAACTGGAGATTCTAGGGGAATTCACAGGTGAGTTTCAGGG
GTGACACATGAACA

Alu in the 5'.

CLONE 265

[LZ72]

Driver: ORF2
Plasmid: AlurescueA70Du $A_{17}CATTACA_{18}GA_{17}CACACA_{18}$ (T)
Chromosome: 22
5' position: 20,311,747
Strand: plus
DR: AAAAAAAAGTGTC
ENDOsite: TTTT/AC

Empty site:

GAAGTCCCAGCTACTTGCCACTTGGGAGACTGAGGTGGGATCACTTGAGCCCAGGAGGTCCAGGCTGCAGTG
AGCTGAGATCACACCACTGTACTGCAGCCTGGGCAACAGAACAAGAATCAAATAATAAATAAGTAAGTAAGT
GAGTAAATCCATCCATCCACCCCAGAGTGAGTCAGCTGGGGCTGCTGCATTGCTCCTCCCTCACCCACAGGA
TTCCCTGGTTCCAGCCAGGAGACGATGGTCAACAGCCACTGCTGTAGTGGTGCCTAGTTAAAACTCTTTGGT
TTCTGTCTCTGT↑**AAAAAAAAGTGTC**↓TAATTGCATTTGGGCAAAAAACTTGCAGTGAAGAGAGAAAAAGAG
ATGAAAGCAACCTGTGAGGGAGGCTCGGGTTGACGTGCGGCATGTGCTGTTTGAGTGTGATGAGTGTGTCGG
TTTGCACTGTAGATGCTCAGGAAGTGAGGATATGTGGAGTGCTGACTGTATACATGATGCCTCCACTGGCTG
GCTGAGTAGAG

Filled site:

GAAGTCCCAGCTACTTGCCACTTGGGAGACTGAGGTGGGATCACTTGAGCCCAGGAGGTCCAGGCTGCAGTG
AGCTGAGATCACACCACTGTACTGCAGCCTGGGCAACAGAACAAGAATCAAATAATAAATAAGTAAGTAAGT
GAGTAAATCCATCCATCCACCCCAGAGTGAGTCAGCTGGGGCTGCTGCATTGCTCCTCCCTCACCCACAGGA
TTCCCTGGTTCCAGCCAGGAGACGATGGTCAACAGCCACTGCTGTAGTGGTGCCTAGTTAAAACTCTTTGGT
TTCTGTCTCTGTAAAAAAAAGTGTC**GGCCGGGCGCGGTGGCTCACGCCTGTAATCCCAGCACTTTGGGAGGC
CGAGGCGGGCGGATCACGAGGTCAGGAGATCGAGACCATCCTGGCTAACACGGTGAAACCCCGTCTCTACTA
AAAAAAAAATACAAAAAATTAGCCGGGCGTGGTAGCGGGCGCCTGTAGTCCCAGCTACTCGGGAGGCTGAGG
CAGGGGAATGGCGTGAACCCGGGAGGCGGAGCTTGCAGTGAGCCGAGATCCCGCCACTGCACTCC… [NEOca
ssette] [Atail]**AAAAAAAAGTGTCTAATTGCATTTGGGCAAAAAACTTGCAGTGAAGAGAGAAAAAGA
GATGAAAGCAACCTGTGAGGGAGGCTCGGGTTGACGTGCGGCATGTGCTGTTTGAGTGTGATGAGTGTGTCG
GTTTGCACTGTAGATGCTCAGGAAGTGAGGATATGTGGAGTGCTGACTGTATACATGATGCCTCCACTGGCT
GGCTGAGTAGAG

Alu in the 5'.

CLONE 266

[LZ73]

Driver: ORF2
Plasmid: AlurescueA70Du $A_{17}CATTACA_{18}GA_{17}CACACA_{18}$ (T)
Chromosome: 22
5' position: 38,002,187
Strand: plus
DR: AAAAGTGACTCC
ENDOsite: TTTT/AA

Empty site:

GTGTAACAGTTACTGTTTTAGGGCTTGGGGATATATTATTAGTGAAGTAACTAAATAAAATCCATTGCCGTG
GTGGAGCTTACATTCTGGTGGGGGAGAGAGACACAAAACACAACAAAGAAATGAAATATGTAGTGGAGATAA
GTGCTAAGGAGAAAAGTTCAGCAGAAAAGGTGGTAGGGAACATGTGGTAGGGGATTAATTTTGCAGACACAC
TTGTTCAAGATCATCCAGAAAGCAGTGAAGTCAGAATTTGCACCTAAATCAAATCTATTTTGTCTTT↑**AAAA**
GTGACTCC↓TGACAGCACAGACATGTCCCTAAATGAATGCAGTAAATGCGATGTGAGTTTTATTTCAGTAAT
AATTCATCTCAGGCTGGGCGTGGTGGCTCACGCCTGTAATCCAAACACTTTGGGTGGTCAAGGCAGGTGGAT
CACCTGAGGTCAGGGGTTCGAGACCCGCCTGGCCAACATGGCGAAACCCTGTCTCTACTAAAAAT

Filled site:

GTGTAACAGTTACTGTTTTAGGGCTTGGGGATATATTATTAGTGAAGTAACTAAATAAAATCCATTGCCGTG
GTGGAGCTTACATTCTGGTGGGGGAGAGAGACACAAAACACAACAAAGAAATGAAATATGTAGTGGAGATAA
GTGCTAAGGAGAAAAGTTCAGCAGAAAAGGTGGTAGGGAACATGTGGTAGGGGATTAATTTTGCAGACACAC
TTGTTCAAGATCATCCAGAAAGCAGTGAAGTCAGAATTTGCACCTAAATCAAATCTATTTTGTCTTTAAAAG
TGACTCCA**GGCCGGGCGCGGTGGCTCACGCCTGTAATCCCAGCACTTTGGGAGGCCGAGGCGGGCGGATCAC**
GAGGTCAGGAGATCGAGACCATCCTGGCTAACACGGTGAAACCCCGTCTCTACTAAAAAAAAATACAAAAAA
TTAGCCGGGCGTGGTAGCGGGCGCCTGTAGTCCCAGCTACTCGGGAGGCTGAGGCAGGGGAATGGCGTGAAC
CCGGGAGGCGGAGCTTGCAGTGAGCCGAGATCCCGCC…[NEOcassette][Atail]AAAAGTGACTCC
TGACAGCACAGACATGTCCCTAAATGAATGCAGTAAATGCGATGTGAGTTTTATTTCAGTAATAATTCATCT
CAGGCTGGGCGTGGTGGCTCACGCCTGTAATCCAAACACTTTGGGTGGTCAAGGCAGGTGGATCACCTGAGG
TCAGGGGTTCGAGACCCGCCTGGCCAACATGGCGAAACCCTGTCTCTACTAAAAAT

L2 in the 5'; Alu in the 3'.

An extra A added immediately 5' of Alu.

CLONE 267

[GG27]

Driver: ORF2
Plasmid: AlurescueA70Du $A_{17}CATTACA_{18}GA_{17}CACACA_{18}$ (T)
Chromosome: X
5’ position: 118,503,604
Strand: minus
DR: N/A
ENDOsite: N/A

Empty site:

TTTAGAGATTGAATGCAATTCCAATTAAAATTCTAAATAGAACCAAGAATAAGCAAGACAATATTAAAGCAG
TACAATATACCCTACCCGATGGCAGCACTTCTTATAAGCTGTAGTAATTAAGGCAGTACAGAGAAAAATAGA
CCAGTGGGACAGAATGCAAAGAGCCCAGAAACCACGTGTAAAGAAAATCTTAATACATGATCAGTGTGGAAA
TGATTTATTTAATAAATGTTGTTGGAACCACTGGTTATTATTTTGG↑↓AAAAAAATAAAATCTCAACCCCAC
ATTGTACACAAAAACAAACTCAAAGTGGTTTTATTTTATTTTATTATTTTTTTGAGATGGAGTCTCACTCTG
TCCCCCAGGCTGGAGTGCAGTGGCGTGATCTCGGCTCACTGCAACCTCTGCCTCCTGGGTTTGAGCGATTCT
CCTGCCTCAGCTTCCTGACTAGCTGGGACTAC

Filled site:

TTTAGAGATTGAATGCAATTCCAATTAAAATTCTAAATAGAACCAAGAATAAGCAAGACAATATTAAAGCAG
TACAATATACCCTACCCGATGGCAGCACTTCTTATAAGCTGTAGTAATTAAGGCAGTACAGAGAAAAATAGA
CCAGTGGGACAGAATGCAAAGAGCCCAGAAACCACGTGTAAAGAAAATCTTAATACATGATCAGTGTGGAAA
TGATTTATTTAATAAATGTTGTTGGAACCACTGGTTATTATTTTGG**GGCCGGGCGCGGTGGCTCACGCCTGT
AATCCCAGCACTTTGGGAGGCCGAGGCGGGCGGATCACGAGGTCAGGAGATCGAGACCATCCTGGCTAACAC
GGTGAAACCCCGTCTCTACTAAAAAAAAATACAAAAAATTAGCCGGGCGTGGTAGCGGGCGCCTGTAGTCCC
AGCTACTCGGGAGGCTGAGGCAGGGGAATGG…[NEOcassette][Atail and 3'
unsequenced]**

LINE-1 flanking the insertion site; Alu in the 3'.

CLONE 268

[GG23]

Driver: ORF2
Plasmid: AlurescueA70Du $A_{17}CATTACA_{18}GA_{17}CACACA_{18}$ (T)
Chromosome: X
5' position: 134,042,388
Strand: minus
DR: AAGATTTACTGCTCCTC
ENDOsite: TCTT/AA

Empty site:

CTGGTCACAAGGTGGTGTGCACACATAAGGTTTTCTTTATAATTTCATTCAGGATTGTTTTTCTGTGCCTTT
GGTACAGGGGAGGAAGACAAGACAGTTTCCTGTATCACCAGATCTCAGCTGCGCTCTCTGCCTGTGAGGTCA
GAAGTGTGTTGGAGCCTCAGATTTCTAAATGCTTGCTGGCACAGTTAGGCACTGAAGAGCCAGGGAAAATCA
GAGGGAAACAGAAGGAGGGGAGGGGAGAAGAGGGGAAGAGAGGTAACTGGGAGTGCTGATTCTCTCTGCTCT
CCTCTTACCCCAACCCATGGTGAGCAGCCACTT↑**AAGATTTACTGCTCCTC**↓TAATTAGGATCCCTTGCTTG
TCTTGTATAGACAAAGTGTGTTCAGCTATGACGGGTGAGTGGTGGGGGGAATGTTCCCCAGTGGAGGAGACA
GTTATTAGTAGACATCTGTAGGATAACTGATAAGCTTTTAGCTATCTGCTAAGTGCCCAGCACTGACCTTGG
GCCTGTGAGGGGTGCAAAAAACAAAAGCATCCCACC

Filled site:

CTGGTCACAAGGTGGTGTGCACACATAAGGTTTTCTTTATAATTTCATTCAGGATTGTTTTTCTGTGCCTTT
GGTACAGGGGAGGAAGACAAGACAGTTTCCTGTATCACCAGATCTCAGCTGCGCTCTCTGCCTGTGAGGTCA
GAAGTGTGTTGGAGCCTCAGATTTCTAAATGCTTGCTGGCACAGTTAGGCACTGAAGAGCCAGGGAAAATCA
GAGGGAAACAGAAGGAGGGGAGGGGAGAAGAGGGGAAGAGAGGTAACTGGGAGTGCTGATTCTCTCTGCTCT
CCTCTTACCCCAACCCATGGTGAGCAGCCACTTAAGATTTACTGCTCCTC**GCTTGCAGTGAGCCGAGATCCC**
GCCACTGCA…[NEOcassette][Atail]AAGATTTACTGCTCCTCTAATTAGGATCCCTTGCTTGTCT
TGTATAGACAAAGTGTGTTCAGCTATGACGGGTGAGTGGTGGGGGGAATGTTCCCCAGTGGAGGAGACAGTT
ATTAGTAGACATCTGTAGGATAACTGATAAGCTTTTAGCTATCTGCTAAGTGCCCAGCACTGACCTTGGGCC
TGTGAGGGGTGCAAAAAACAAAAGCATCCCACC

No repetitive sequences in the immediate proximity.

Alu insert truncation: missing 220 bp of 5' Alu sequence.
