# Supplementary material for: Rescuing Alu: Recovery of New Inserts Shows LINE-1 Preserves Alu Activity through A-Tail Expansion
Source: PLoS Genet. 2012 Aug 9;8(8):e1002842. doi: 10.1371/journal.pgen.1002842 (PMC3415434; doi:10.1371/journal.pgen.1002842)
Supplement: Text S2 — Sequences of the pre-insertion and post-insertion genomic sites of the de novo BC1 or B2 inserts. (PDF) [file pgen.1002842.s009.pdf]

BC1 and B2 RESCUES

GRCh37/hg19 assembly

CLONE 1 BC1

[MP#1]

Driver: ORF2
Plasmid: BC1rescueA70D $A_{17}CATTACA_{18}GA_{17}CACACA_{18}$ (T)
Chromosome: 14
5' position: 50329568
DR: unable to determine

ENDOsite: unable to determine

Empty site:

GAAAACAAACAGTTGCCCCAGGCTTCCTTCGTTCGAATAGTGAAGACTGAGCTGGCCTCGGCACTGTGCAGA
CTCCCAACCTGTGCTACTGTGGGTTATTCTCCTTATAGTGCAGTCACCTCCTAAATGCTTGCTGAAAATAAT
GGAAGACTTCGAGCCAGATGTTTGCCTCTTAAGTAACGTCCCCCAAAAGATGCAGCACATATTTAAGGCCTG
CTGAAGCAAGCAACGCTGACGGACCAGGTTATTGCGTTTTTCTCTCGCTGCGTGTCCGGACGCCACACTATG
AGCATTAAGCGCATACTGACCAATTTCTGTTAAGCAGGAACGTGGTGTTCTGATCAAAACACAGGGAGCCGC
GGCCGCAACCGCGCCGATAAGCTTCCGGGGTCCCAACCCTGTAAGGCAGAACGGCTGCCCAGCTCGGATGAC
GCCACACTAACGTAGCCTCCAGACCGCCCAGTGTGGGTGTGTCCAAGCTCACGTCGCCCGGCGTGGCCCCCC
CGCTCCCCAATGACGTAACTGCCCTGCAGCTTCTAGTAGCTTTTCGCAGCGTCTCCGACC↓GCCGGGCGCGG
TGGCGCGTGCCTGTAGTCCCAGCTACTCGGGAGGCTGAGGTGGGAGGATCGCTTGAGCCCAGGAGTTCTGGG
CTGTAGTGCGCTATGCCGATCGGGTGTCCGCACTAAGTTCGGCATCAATATGGTGACCTCCCGGGAGCGGGG
GACCACCAGGTTGCCTAAGGAGGGGTGAACCGGCCCAGGTCGGAAACGGAGCAGGTCAAAACTCCCGTGCTG
ATCAGTAGTGGGATCGCGCCTGTGAATAGCCACTGCACTCCAGCCTGAGCAACATAGCGAGACCCCGTCTCT
TTTGCCCCCCTCCCTCATTAAGGGTACCTTTGTAAGTAAATAGTGTCTTTGAAGTTAAGAAGTTTGCCTCCT
TTCCGGTTCATACGTATTAAGAAACATACTAATGTGCACATTTAA

Filled site:
GAAAACAAACAGTTGCCCCAGGCTTCCTTCGTTCGAATAGTGAAGACTGAGCTGGCCTCGGCACTGTGCA
GACTCCCAACCTGTGCTACTGTGGGTTATTCTCCTTATAGTGCAGTCACCTCCTAAATGCTTGCTGAAAA
TAATGGAAGACTTCGAGCCAGATGTTTGCCTCTTAAGTAACGTCCCCCAAAAGATGCAGCACATATTTAA
GGCCTGCTGAAGCAAGCAACGCTGACGGACCAGGTTATTGCGTTTTTCTCTCGCTGCGTGTCCGGACGCC
ACACTATGAGCATTAAGCGCATACTGACCAATTTCTGTTAAGCAGGAACGTGGTGTTCTGATCAAAACAC
AGGGAGCCGCGGCCGCAACCGCGCCGATAAGCTTCCGGGGTCCCAACCCTGTAAGGCAGAACGGCTGCCC
AGCTCGGATGACGCCACACTAACGTAGCCTCCAGACCGCCCAGTGTGGGTGTGTCCAAGCTCACGTCGCC
CGGCGTGGCCCCCCCGCTCCCCAATGACGTAACTGCCCTGCAGCTTCTAGTAGCTTTTCGCAGCGTCTCC
GACT**GGGGTTGGGGATTTAGCTCAGTGGTAGAGCGCT…[NEOcassette]AAAAAAAAAAAAAAAAAA**
AAAAAAAAAAAAAAAAAAAAAA

Note: we were unable to recover the 3' flank to verify direct repeats and endonuclease cleavage site.

CLONE 2 BC1

[MP#1, 5, 34, 35, 36, 38]

Driver: ORF2
Plasmid:BC1rescueA70D $A_{17}CATTACA_{18}GA_{17}CACACA_{18}$ (T)
Chromosome: 12
5' position: 96192169
DR: AAGATTACAATGCA
ENDOsite: TCTT/GT

Empty site:

AAATCAAGCTTAAAAAAGCTCACAATCTACTGAAGCAGCAATTTGATTAAAAACATCCCTTGTCCATAGGTG
GGATTTCAAGATATGTAATATAGTAGAATATGTTCAAGATGTTAATTAAGCTAGATATGCTTCTGGTTTGGT
TTTATCTATAGCTCCTAATTACCCTGGACACTTTCCGATCCTAAACCTGTGCTGCAATTTCAAACACAAGTG
CCTTTCTGAATATTTTTATTACAATTGTGCCTTAC↑AAGATTACAATGCA↓ATTTAGAGGGTAAATCTATGA
CACAATCTGCTTCAACCCTAAATTCAGTATAATTGACCTCATCATAGAAGAGGCATTCAGTTATCAGAAACA
TTAGAAAAACTTCATTAACCAGTATTAAAACTGTACCATCTCAGAGCTCAAATTAATGGTTTCAGGATGTTA
ATACATTTTGAAAACCTGTAAATGAATTTTAAGCAAATTGGTTTGGAAGGCCGAGGTGGGTGGATTACATGA
CGTCAGGAGTTTGAGACCTGCCTGGCCAACATGGTGAAACCCCGTCTCTACTAAAAATACAAA

Filled site:

AAATCAAGCTTAAAAAAGCTCACAATCTACTGAAGCAGCAATTTGATTAAAAACATCCCTTGTCCATAGGTG
GGATTTCAAGATATGTAATATAGTAGAATATGTTCAAGATGTTAATTAAGCTAGATATGCTTCTGGTTTGGT
TTTATCTATAGCTCCTAATTACCCTGGACACTTTCCGATCCTAAACCTGTGCTGCAATTTCAAACACAAGTG
CCTTTCTGAATATTTTTATTACAATTGTGCCTTACAAGATTACAATGCA**GGGGTTGGGGATTTAGCTCAGTG**
GTAGAGCGCTTGCCTAGCAAGCGCAAGGCCC…**[NEOcassette]*AAAAAAAAAAAAAAAAAAAAAAAAAAA***
AAAAAAAAAAAAAAAAAAAAAAAAAAAAAAAAAGATTACAATGCAATTTAGAGGGTAAATCTATGACACAAT
CTGCTTCAACCCTAAATTCAGTATAATTGACCTCATCATAGAAGAGGCATTCAGTTATCAGAAACATTAGAA
AAACTTCATTAACCAGTATTAAAACTGTACCATCTCAGAGCTCAAATTAATGGTTTCAGGATGTTAATACAT
TTTGAAAACCTGTAAATGAATTTTAAGCAAATTGGTTTGGAAGGCCGAGGTGGGTGGATTACATGACGTCAG
GAGTTTGAGACCTGCCTGGCCAACATGGTGAAACCCCGTCTCTACTAAAAATACAAA

CLONE 3 B2

[MP# 19; 20]

Driver: ORF2
Plasmid: B2rescueA70D $A_{17}CATTACA_{18}GA_{17}CACACA_{18}$ (T)
Chromosome: 1
5’ position: 151124725
DR: AAAAATACAAAAAATT
ENDOsite: TTTT/AA

Empty site:

CTTTTATCTTTTTATCCCCAGCACTTAATTGTAATGCCTGGCATACAGTAAGTAGAGGAAGAAATAAATGCA
TCAAAGATTAAAAACAAACAAACATCCTATGGCTCAGAGGAAAAACAACAACAACAACAAAAAAACAAAAACA
AAAAAAAAACAAGCCAGGTATGGTGGCTCATTTCTGTAATCCCAGCATTTTGGGAGGCCAAGGCAGGCGAAT
CATGAGGTCAGGAGATCGAGACCATCCTGGCTAACATGGTGAAACCCCGTGTCGATT↑AAAAATACAAAAAA
TT↓AGCTGGGCGTGGTAGCGCGCGCCTGTAATCCCAGCTACTCAGGAGGCTGAGGCAGGAGAATAGCTTGAA
CCTGGGAGGCAGAGGTTGCAGTGAGCCGAGATCGTGCCACTGCACTCCAGCCTGGGCGACAGAGTGAGACTC
CATCTCAAAAAAGTAAATAAATAAAAATAGGACAGGCACGGTGGTTCACACCTGTAATCCCAGCACTTCGGG
AGGCCGAGCTGGGCGAATCATGAGGTCAGGAGTTCAAGACCAGCCTGGCCAACATAGTGAAACCCCGTCTCT
ACTAAAAATACAAAA

Filled site:

CTTTTATCTTTTTATCCCCAGCACTTAATTGTAATGCCTGGCATACAGTAAGTAGAGGAAGAAATAAATGCA
TCAAAGATTAAAAACAAACAAACATCCTATGGCTCAGAGGAAAAACAACAACAACAACAAAAAAACAAAAACA
AAAAAAAAACAAGCCAGGTATGGTGGCTCATTTCTGTAATCCCAGCATTTTGGGAGGCCAAGGCAGGCGAAT
CATGAGGTCAGGAGATCGAGACCATCCTGGCTAACATGGTGAAACCCCGTGTCGATTAAAAATACAAAAAAT
T**GGGCTGGTGAGATGGCTCAGTGGGTTAGAGCACCCGACTGCTCTTCCGAAGGTCCAGAGTTCAAATTCCAG**
CAACCACATGGTGGCTCACAACCATCCGTAACAAGATCTGACTCCCTCTTCTGGAGTGTC…**[NEOcassett**
e]***AAAAAAAAAAAAAAAAAAAAAAAAAAAAAAAAAAAAAAAAAAAAAA***TACAAAAAAATTAGCTGGGCGGGG
TAGCGCGCGCCGGTAATCCCAGCTACTCAGGAGGCTGAGGCAGGAAAATAGCTTGAACCTGGGAGGCAAAGG
TTGCAGTGAGC

CLONE 4 B2

[MP# 48]

Driver: ORF2
Plasmid: B2rescueA70D $A_{17}CATTACA_{18}GA_{17}CACACA_{18}$ (T)
Chromosome: 9
5' position: 20623400
DR: AAGACTCCCGGTT
ENDOsite: TCTT/AA

Empty site:

AAGAAAGGGACAAAGAGGAAAGAAAGAGGGACAAAGAGGAAAGGCAAGGAGAAGAGAAGGAAAAATCAAGGT
TCAGCAAGAAGTTGGAAGGCGAGGGAAAAAGGGGAGTAAAGAGAAGGCAAAAGAGGTCTAAGGGCTAGGAAG
ATGCAAGGATCTTTTTTTAACGAACCAAAGTCACATCCATCTTTCACTTTCAAATCAATCGTCTAGATCTTA
TCCGTTTCCTTTTTAAAGGTTATCTGCGTCTGTAAAGGAAGCCAGTAGTTGATACTCCTCTCCGCGCCCCAC
GCGTGGCCGCGCTGTTCTATCTCCGGCCACTGAGATCAAATACAACGCGAGTGAGAACATTTGCAGAGGCGC
GACTCTGGGAACACTCCTGCGACTGGCTGTCACCCGGGAGTCTAACCGGATTAATACTCAGAGATGGATTAC
GCTGGTTGTCACCAATACGCCTAAAGATTATAATCCAATTTACGCAAATTACAGTCAGTATTAACTTTATCT
GCTATTTT↑AAGACTCCCGGTT↓GCCCCGCGCGTCCTCAGAGGACAATCCTCCACGTTCTGATGCAACTCAG
TAGGCTGTCAGGAGGGGAAGCCCCCCGAAGAAAACAACTCCAAGCCTGGTCCCCGAGGGTCTGTACCCGGCA
CTCCCAGGTGTCTTGCTTTCACCTGGGCACCGCTTTATGAAGCTTCCCAGCCGGGGATTACCAAAGCGGAAG
GCGTTCCCTTGCAAGGAAATAGCATCCTTGTTTCTCCCTTGTTCATTTCATTTTCAAATTTTCGGTGGCGGT
GGCAGAAGGGCGGGGAGTGAGGGTGGCGGGCGGGT

Filled site:

AAGAAAGGGACAAAGAGGAAAGAAAGAGGGACAAAGAGGAAAGGCAAGGAGAAGAGAAGGAAAAATCAAGGT
TCAGCAAGAAGTTGGAAGGCGAGGGAAAAAGGGGAGTAAAGAGAAGGCAAAAGAGGTCTAAGGGCTAGGAAG
ATGCAAGGATCTTTTTTTAACGAACCAAAGTCACATCCATCTTTCACTTTCAAATCAATCGTCTAGATCTTA
TCCGTTTCCTTTTTAAAGGTTATCTGCGTCTGTAAAGGAAGCCAGTAGTTGATACTCCTCTCCGCGCCCCAC
GCGTGGCCGCGCTGTTCTATCTCCGGCCACTGAGATCAAATACAACGCGAGTGAGAACATTTGCAGAGGCGC
GACTCTGGGAACACTCCTGCGACTGGCTGTCACCCGGGAGTCTAACCGGATTAATACTCAGAGATGGATTAC
GCTGGTTGTCACCAATACGCCTAAAGATTATAATCCAATTTACGCAAATTACAGTCAGTATTAACTTTATCT
GCTATTTTAAGACTCCCGGTTG**GGCTGGTGAGATGGCTCAGTGGGTTAGAGCACCCGACTGCTCTTCCGAAG**
GTCCAGAGTTCAAATTCCAGCAACCACATGGTGGCTCACAACCATCCGTAACAAGATCTGACTCCCTCTTCT
GGAGTGTC…[NEOcassette]*AAAAAAAAAAAAAAAAAAAAAAAAAAAAAAAAAAAAAAAAAAAACATTAC*
AAAAAAAAAAAAAAAAAAAAAAAAAAAAAGACTCCCGGTTGCCCCGCGCGTCCTCAGAGGACAATCCTCCAC
GTTCTGATGCAACTCAGTAGGCTGTCAGGAGGGGAAGCCCCCCGAAGAAAACAACTCCAAGCCTGGTCCCCG
AGGGTCTGTACCCGGCACTCCCAGGTGTCTTGCTTTCACCTGGGCACCGCTTTATGAAGCTTCCCAGCCGGG
GATTACCAAAGCGGAAG

CLONE 5 B2

[MP# 11, 41]

Driver: ORF2
Plasmid: B2rescueA70D   A$_{17}$CATTACA$_{18}$GA$_{17}$CACACA$_{18}$ (T)
Chromosome: 3
5' position: 70268177
DR: AAAAAAGAATGTTCA
ENDOsite: TTTT/AA

Empty site:

ATTTTCTTTTGCTTCCTTTCGGTACCCAGGGTGTGGTTGCTGGAACGTTTACAGCCACAGCAGCTGCAGTAG
TGGCCTGAGTAGACTTTTCCTCAGAGTTTCTATAGTGGTAACAGGATTAACAGACTCTATTCTGGAAGTACT
TGGGTGGCATAACACCTCCCAGCAGAACTATTCCTGACAAGGATGTGCAGATGGAAATGCATATTTATGTCA
CTTAAAGCCTACAAGAGACACAAGGATTTGAGCTATAACAATAAATAGAAAAAAAAATTCTTCAGCATTTTT
GGTAACCCATACTCAAGTGCATTGACTGTGCTTGGCGAAAGACCTACAGTTTGCTCTTTGTCCAAGCATAAA
ATATTCAGCATTCAAGCATGTTGAGGTGAACTATTCTGAATATATTTTAGAGTGTTAAAATAAGCTACCACA
AAGTTAGTTTAAAATTTCAGGTCTTAAAAATAACAGGACAATTTTATGAAGTGTTTGGGCCATTTCAAGAAA
AAAAAAGTCCCCTTT↑AAAAAAGAATGTTCA↓TTTGTTCCAATTTTTAGTAAAGGCAGGTTTAAAAAAATTA
TTTCTAAACACCAAATAGTCATTATAGTCAAGGCTTTCAAGTTTAAGGTATTGTCTGCATCACAGTCCTTGA
AACAGCCTCTCCTAGCTTTCTTCTAAAACACCTTTAAAAATATGGAAAAACAAGAATGCTCACATCTGCAAA
TTTGGGTTTGATAGTATTGGGAAAGATTTACACTAAATATCAACCCATTAGAATTGAACTGAATAATCCAAT
CAGTCTAGCACTTACAGAGCTCTGCTGAAAGTAGAATGGAATTA

Filled site:

ATTTTCTTTTGCTTCCTTTCGGTACCCAGGGTGTGGTTGCTGGAACGTTTACAGCCACAGCAGCTGCAGTAG
TGGCCTGAGTAGACTTTTCCTCAGAGTTTCTATAGTGGTAACAGGATTAACAGACTCTATTCTGGAAGTACT
TGGGTGGCATAACACCTCCCAGCAGAACTATTCCTGACAAGGATGTGCAGATGGAAATGCATATTTATGTCA
CTTAAAGCCTACAAGAGACACAAGGATTTGAGCTATAACAATAAATAGAAAAAAAAATTCTTCAGCATTTTT
GGTAACCCATACTCAAGTGCATTGACTGTGCTTGGCGAAAGACCTACAGTTTGCTCTTTGTCCAAGCATAAA
ATATTCAGCATTCAAGCATGTTGAGGTGAACTATTCTGAATATATTTTAGAGTGTTAAAATAAGCTACCACA
AAGTTAGTTTAAAATTTCAGGTCTTAAAAATAACAGGACAATTTTATGAAGTGTTTGGGCCATTTCAAGAAA
AAAAAAGTCCCCTTTAAAAAAGAATGTTCA**GGGCTGGTGAGATGGCTCAGTGGGTTAGAGCACCCGACTGCT**
CTTCCGAAGGTCCAGAGTTCAAATTCCAGCAACCACATGGTGGCTCACAACCATCCGTAACAAGATCTGACT
CCCTCTTCTGGAGTGTC… [NEOcassette] ***AAAAAAAAAAAAAAAAAAAAAAAAAAACATTACAAAAAAAA***
AAAAAAAAAAAAAAAAAAAAAAAAAAAAAAAAAAAAAAAAAAAAAAAAAAAAAAAAAAAAAAAGAATGTTCA
TTTGTTCCAATTTTTAGTAAAGGCAGGTTTAAAAAAATTATTTCTAAACACCAAATAGTCATTATAGTCAAG
GCTTTCAAGTTTAAGGTATTGTCTGCATCACAGTCCTTGA

CLONE 6 B2

[MP# 13]

Driver: ORF2
Plasmid: B2rescueA70D A_{17}CATTACA_{18}GA_{17}CACACA_{18} (T)
Chromosome:7
5' position: 2843712
DR: AAGAGGACGAGATTTCG
ENDOsite: TCTT/AA

Empty site:

AAAAAAAAAAAAAAAAAAGAGGACGATTTCGTTCCCCCTTGTACTAGTGTTTGTCATGTAGTGAAATTTTTA
GTGAAGGGAGATTCCGAGACTTATCCTGACCCCTGGAAGAACAGGAGAGGAAGAAGTTGGTCTGCCCATGGC
CATGGCTGGCGGGTGGGGGTTGGGGAGATGATTCCAGGAGTTTCCAGCCTGCTGGCTTGTGAGTTTTGAAAT
GGAGCCCTGGATTTTCACTTTCCAGTTCAGGAGATCTGGGGAAATCTGGATTGGTTTGGAGTTGTTTTTTTG
TTTGTTTGTTTTGTTTTGTTTTGTTTTGGACTTTTAGGAAAACCTTTTCAAGGCAGCTGAAAAAAGCTAATG
ATGATAATTTGCTTCTCCTATGCCATTCCTAAACCCAGAGTTTTCTCTGCAGTTCCATCTCCTCTTGAGAAG
CTTTTCCCCCTTAGAATGATATTTTGTGTGTTTGTGTTCTCAAGTTTTGCTGATCTTTTCTGTACTT↑AAGA
GGACGATTTCG↓TTCCCCCTTGTACTAGTGTTTGTCATGTAGTGAAATTTTTAGTGAAGGGAGATTCCGAGA
CTTATCCTGACCCCTGGAAGAACAGGAGAGGAAGAAGTTGGTCTGCCCATGGCCATGGCTGGCGGGTGGGGG
TTGGGGAGATGATTCCAGGAGTTTCCAGCCTGCTGGCTTGTGAGTTTTGAAATGGAGCCCTGGATTTTCACT
TTCCAGTTCAGGAGATCTGGGGAAATCTGGATTGGTTTGGAGTTGTTTTTTTGTTTGTTTGTTTTGTTTTGT
TTTGTTTTGGACTTTTAGGAAAAC

Filled site:

AAAAAAAAAAAAAAAAAAGAGGACGATTTCGTTCCCCCTTGTACTAGTGTTTGTCATGTAGTGAAATTTTTA
GTGAAGGGAGATTCCGAGACTTATCCTGACCCCTGGAAGAACAGGAGAGGAAGAAGTTGGTCTGCCCATGGC
CATGGCTGGCGGGTGGGGGTTGGGGAGATGATTCCAGGAGTTTCCAGCCTGCTGGCTTGTGAGTTTTGAAAT
GGAGCCCTGGATTTTCACTTTCCAGTTCAGGAGATCTGGGGAAATCTGGATTGGTTTGGAGTTGTTTTTTTG
TTTGTTTGTTTTGTTTTGTTTTGTTTTGGACTTTTAGGAAAACCTTTTCAAGGCAGCTGAAAAAAGCTAATG
ATGATAATTTGCTTCTCCTATGCCATTCCTAAACCCAGAGTTTTCTCTGCAGTTCCATCTCCTCTTGAGAAG
CTTTTCCCCCTTAGAATGATATTTTGTGTGTTTGTGTTCTCAAGTTTTGCTGATCTTTTCTGTACTTAAGAG
GACGATTTCG**GGGCTGGTGAGATGGCTCAGTGGGTTAGAGCACCCGACTGCTCTTCCGAAGGTCCAGAGTTC**
AAATTCCAGCAACCACATGGTGGCTCACAACCATCCGTAACAAGATCTGACTCCCTCTTCTGGAGTGTC…**[N**
EOcassette]*AAAAAAAAAAAAAAAAAAAAAAAAAACATTACAAAAAAAAAAAAAAAAAAAAAAAAAAAAA*
AAAAAAAAAAAAAAAAAAAAAAAAAAAAAAAAGAGGACGAGATTTCGTTCCCCCTTGTACTAGTGTTTGTCA
TGTAGTGAAATTTTTAGTGAAGGGAGATTCCGAGACTTATCCTGACCCCTGGAAGAACAGGAGAGGAAGAAG
TTGGTCTGCCCATGGCCATGGCTGGCGGGTGGGGGTTGGGGAGATGA

CLONE 7 B2

[MP# 16, 42]

Driver: ORF2
Plasmid: B2rescueA70D $A_{17}CATTACA_{18}GA_{17}CACACA_{18}$ (T)
Chromosome:3
5' position: 133956392
DR: AAGAACCATTAGTT
ENDOsite: CCTT/AA

Empty site:

AGGTAGCATTTTGTCATTAGAATGCACAGAATTTTTAAATTCTGATTTAGGATGAAAATACTTCAGCTGTTC
AGTTACATTAACCAGACTTAAATTTCCCATTGCCAATTCCAGCAAAGGAAAAATGTTGCTCTCTTGTTAGAA
AGCATGGCATTTTTTCAAAATTTTATTTTTAAACAACTCAAATTCAAAGCTAAAAAACCAAAGTTATTCTTG
CAAAACTCTTAAGGCTTATCAAAAAAAAATTCATTTTTCCCTAGACGATTTTAAGTTCAAATTTATTTCAAGG
TCTGTGATCCACATGAACTGGCCTAACTCAAGTTCTAATAATTCAACAGTTCAAAAATTTTTTCCAAATGAT
TTTAAAAGTTGACTATTGGTTTCTCAAAGCTGTACATATGGTTCATTCGTGCTAAATCAGCAAACCATCTCT
CTTTATTTTAAAAGCCACCTTTATAGCACTTAACAAAACATATTTCAGGGAGTTGTTTCAAATGTCCATCTT
CCTATTACCTTGCACATCTCACAAACAAGGCTGGGGTCTTAGTTTACTTGTAACTACTTAAATATTAGATGC
ATGAATGGCTGGCTGTCGATAAAAGTCAACTACATCAAAGATGGGTTTTAAAGAAAATGGGTTTTAAAGAAA
ATGATACTTTAAAGAAAATGTC↑AAGAACCATTAGTT↓AAGGCAAAAAAACAAAAGTGATGTCATAGAAAGA
GAACTAAAGCAGAGTTCACAGTTCTTACTTCCCTTCCCCTGGCTTCACCAGTAGGTAGCTGAATGACCTTTG
GCAACCAATTACTTAACCGCCCTCTCTAGACAACAATTTTCTCAACTACAAAACAAAAAAGTTAAACCAGAT
GATGTTAATGTCCCTCTCCTCAGCCGCAAAATTCTATTACTCTATGATATCATCCATGGTTTCCTAGTGTCA
GCCAAAACACTATTATTTTATTATATCATCCACAGTTTCCATATGCCATT

Filled site:

AGGTAGCATTTTGTCATTAGAATGCACAGAATTTTTAAATTCTGATTTAGGATGAAAATACTTCAGCTGTTC
AGTTACATTAACCAGACTTAAATTTCCCATTGCCAATTCCAGCAAAGGAAAAATGTTGCTCTCTTGTTAGAA
AGCATGGCATTTTTTCAAAATTTTATTTTTAAACAACTCAAATTCAAAGCTAAAAAACCAAAGTTATTCTTG
CAAAACTCTTAAGGCTTATCAAAAAAAAATTCATTTTTCCCTAGACGATTTTAAGTTCAAATTTATTTCAAGG
TCTGTGATCCACATGAACTGGCCTAACTCAAGTTCTAATAATTCAACAGTTCAAAAATTTTTTCCAAATGAT
TTTAAAAGTTGACTATTGGTTTCTCAAAGCTGTACATATGGTTCATTCGTGCTAAATCAGCAAACCATCTCT
CTTTATTTTAAAAGCCACCTTTATAGCACTTAACAAAACATATTTCAGGGAGTTGTTTCAAATGTCCATCTT
CCTATTACCTTGCACATCTCACAAACAAGGCTGGGGTCTTAGTTTACTTGTAACTACTTAAATATTAGATGC
ATGAATGGCTGGCTGTCGATAAAAGTCAACTACATCAAAGATGGGTTTTAAAGAAAATGGGTTTTAAAGAAA
ATGATACTTTAAAGAAAATGTCAAGAACCATTAGTT**GGGCTGGTGAGATGGCTCAGTGGGTTAGAGCACCCG**
ACTGCTCTTCCGAAGGTCCAGAGTTCAAATTCCAGCAACCACATGGTGGCTCACAACCATCCGTAACAAGAT
CTGACTCCCTCTTCTGGAGTGTC…[NEOcassette]*AAAAAAAAAAAAAAAAAAAAAAAAACATTACAAAA*
AAAAAAAAAAAAAAAAAAAGAAAAAAAAAAAAAAAAAAAAAAAAAAAAAAAAAAAAAGAACCATTAGTTAAG
GCAAAAAAACAAAAGTGATGTCATAGAAAGAGAACTAAAGCAGAGTTCACAGTTCTTACTTCCCTTCCCCTG
GCTTCACCAGTAGGTAGCTGAATGACCTTTGGCAACCAAT
